# Health economic and hospital resource use in isolated mitral valve repair in more than 8000 patients: Robotic versus nonrobotic approaches

**DOI:** 10.1016/j.xjon.2026.102067

**Published:** 2026-08-19

**Authors:** Makoto Hashimoto, Masaki Nakamura, Matthew Reifenberger, Shannon M.E. Murphy, Tom C. Nguyen

**Affiliations:** a Florida International University, Herbert Wertheim College of Medicine, Miami, Fla; b Department of CardioThoracic Surgery, Miami Cardiac & Vascular Institute, Baptist Health South Florida, Miami, Fla; c Edwards Lifesciences, Irvine, Calif

**Keywords:** mitral valve repair, robotic surgery, healthcare resource use

## Abstract

**Objective:**

To explore the health economics and hospital resource use in isolated mitral valve repair (MVr) in patients undergoing robotic versus nonrobotic surgical approaches.

**Methods:**

We conducted a retrospective cohort study using the Premier Healthcare Database for patients who underwent isolated MVr surgery from 2019 to 2024. Hospital chargemaster data was text mined for “robotic,” and patient and procedure characteristic and outcomes were stratified by robotic versus traditional surgical approaches. Differences in outcomes (MVr hospitalization cost, intensive care unit [ICU] days, length of stay, operating room time, prolonged ventilation, and 30-day cardiovascular [CV] readmissions) were estimated using generalized linear modeling with adjustment for patient, procedure, and hospital characteristics.

**Results:**

A total of 8025 patients received an isolated MVr (mean age 57.8 ± 17.7 years, 39.4% female). Overall, 780 (9.7%) of the procedures were performed robotically, with robotic use generally increasing each year from 7.7% in 2019 to 14.7% in 2024. Compared with traditional (nonrobotic) surgery, robotic cases cost 12% more ($57,678 robotic vs $51,530 nonrobotic, *P* < .0001). However, patients who underwent robotic surgery spent 14% less time in the ICU (2.4 vs 2.8 days, *P* < .0001) and 16% less time in the hospital overall (5.7 vs 6.8 days, *P* < .0001). Robot use was also associated with lower 30-day CV readmission rates (3.1% vs 4.9%, *P* = .0215). There was no statistical difference in operating room time or prolonged ventilation use.

**Conclusions:**

Robotic use in isolated MVr procedures has increased ∼7% over the last 5 years. This signals ongoing physician and hospital adoption of robotic use in MVr. The use of innovative robotic interventions comes at an increased cost to the hospital but may be associated with procedural efficiencies, including shorter ICU days and shorter overall hospital stays, as well as lower 30-day CV readmission rates, which may have important clinical and economic benefits for patients and health care systems.

**Figure:**
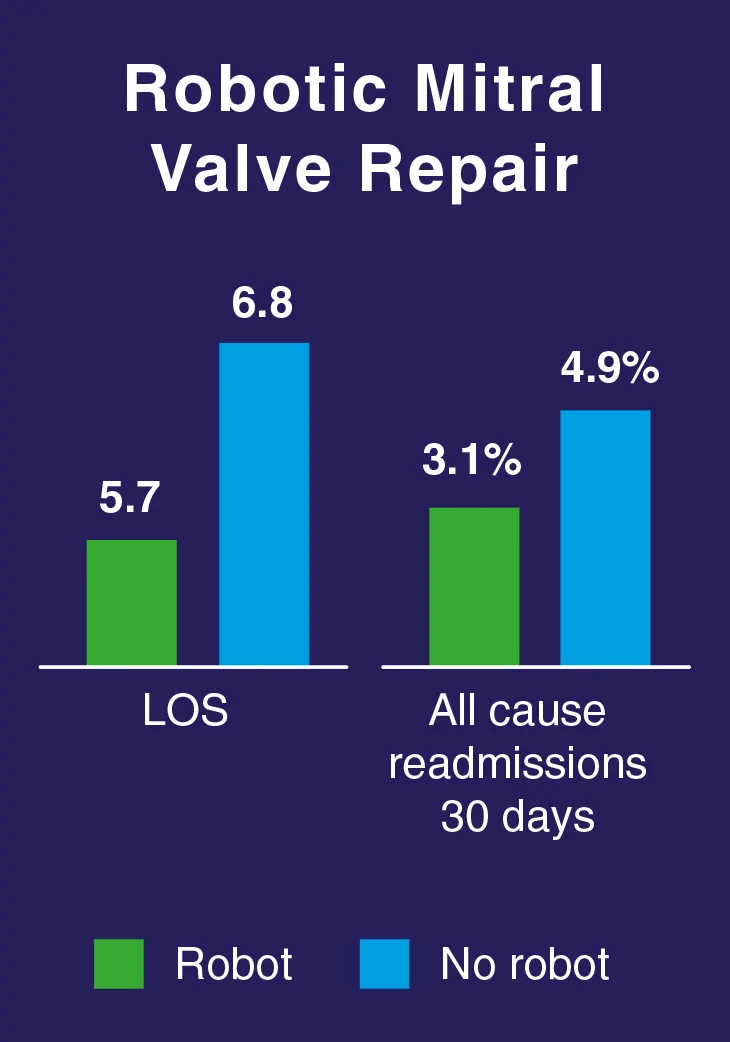
*LOS*, Length of stay.

**Central Message**

Robotic mitral valve repair improves recovery, with shorter stays and fewer readmissions, maintaining comparable mortality; slightly greater costs are driven by operating room expenses.

**Perspective**

Although robotic-assisted mitral valve repair is growing, real-world evidence remains limited. In this large national analysis, robotic surgery was associated with shorter ICU and hospital stays, fewer readmissions, and comparable mortality versus nonrobotic approaches. Although total costs were greater, differences were driven by operating room (OR) expenses, with similar non-OR costs.

Robotic-assisted mitral valve repair (MVr) has emerged as an established minimally invasive approach for the treatment of mitral regurgitation, offering several potential advantages over conventional sternotomy, including smaller incisions, reduced pain, shorter hospital stays, and faster return to functional activity.^1^, ^2^, ^3^ Since the first robotic MVr performed by Carpentier in 1998 and subsequent approval by the Food and Drug Administration of the da Vinci Surgical System (Intuitive Surgical) for intracardiac use in 2002,^4^^,^^5^ adoption of this technology has expanded considerably at specialized centers worldwide. Recent meta-analyses have demonstrated that robotic MVr is associated with significantly shorter intensive care unit (ICU) and hospital lengths of stay, reduced blood transfusion rates, and comparable or lower mortality compared with conventional sternotomy, despite longer operative and cardiopulmonary bypass times.^2^^,^^3^^,^^6^

However, much of the existing literature derives from single-center experiences at high-volume academic institutions with dedicated robotic programs, raising questions about the generalizability of these outcomes to broader clinical practice. A recent national analysis by Hadaya and colleagues^7^ using the Nationwide Readmissions Database found comparable short-term outcomes between robotic and conventional approaches, although costs remained greater for robotic surgery except at the greatest-volume centers. Understanding real-world use patterns and outcomes across diverse hospital settings is essential for informing clinical decision-making and resource allocation. The purpose of this study was to evaluate the outcomes of robotic-assisted versus nonrobotic approaches for isolated MVr using the Premier Healthcare Database (PHD), a large, nationally representative dataset reflecting contemporary US practice patterns.

## Methods

### Data Source

The PHD is a large, US hospital–based, service-level, all-payor database containing information on inpatient discharges from geographically diverse nonprofit, nongovernmental, community, and teaching hospitals and health systems from rural and urban areas. The database includes more than 170 million inpatient visits representing approximately 25% of annual US inpatient admissions and more than 341 million unique patients from more than 1370 contributing hospitals and health care systems.^8^ The PHD contains information on hospital and visit characteristics, patient demographics, disease states, admission and discharge diagnoses (*International Classification of Diseases* [ICD], *Ninth Revision* and *Tenth Revision* [-10]), procedural codes (ICD, Current Procedural Terminology, and Healthcare Common Procedure Coding System), detailed pharmacy data, medical device and supply use, laboratory results, and billing information including costs and charges at the departmental level. Using a unique masked identifier, patients can be tracked within the same hospital across inpatient and hospital-based outpatient settings. All patient-related data in the PHD are deidentified and compliant with the Health Insurance Portability and Accountability Act Privacy Rule. Because this study involved a retrospective analysis of a deidentified database, it was classified as exempt from institutional review board oversight under 45 CFR 46.101(b) (4). Consent was not required.

### Study Population

Patients who underwent MVr between January 2019 and December 2024 were identified in the PHD using ICD-10 procedure codes (Online Data Supplement). Patients undergoing concomitant cardiac procedures were excluded, including heart transplant, coronary artery bypass grafting, cardiac ablation, left atrial appendage closure, left ventricular assist device implantation, maze procedure, and aortic, tricuspid, or pulmonic valve procedures (Online Data Supplement).

### Variables of Interest

Patients were stratified by the use of robotic-assisted surgery for MVr versus nonrobotic approaches. Robotic use was identified using a series of expert rules that incorporated ICD-10/Healthcare Common Procedure Coding System procedure codes (Online Data Supplement) indicating robotic-assisted procedures and text mining of hospital chargemaster data for robotic platform-specific terms. Baseline patient characteristics included age, sex, race (White, Black, Asian, or missing/unknown), ethnicity (Hispanic, non-Hispanic, or missing/unknown), primary payor (commercial, Medicaid, Medicare, or missing/unknown), and admission type (elective or nonelective). Comorbidity burden was assessed using the Elixhauser Comorbidity Index (Online Data Supplement), which measures patient comorbidity on the basis of ICD-10 diagnosis codes across 31 comorbidity categories and has been validated as a predictor of hospital resource use and in-hospital mortality.^9^ Hospital-level characteristics included geographic region (Midwest, Northeast, South, West), teaching status (academic or community), urban versus rural location, and bed size.

Outcomes of interest included clinical outcomes (in-hospital mortality, prolonged mechanical ventilation [Online Data Supplement]), resource use (operating room time, ICU length of stay, total hospital length of stay, 30-day cardiovascular [CV] readmissions), and hospital costs. Hospital costs excluding operating room costs were also evaluated to assess cost differences independent of the capital expenditures and instrumentation costs associated with the robotic surgical system. In addition, 30-day CV readmission costs were evaluated.

### Statistical Analyses

Descriptive statistics were used to summarize patient and hospital characteristics. Continuous variables are reported as means with SDs, and categorical variables are reported as frequencies with percentages. Differences between robotic and nonrobotic groups were assessed using *t* tests for continuous variables and χ^2^ tests for categorical variables.

Multivariable regression models were used to compare outcomes between robotic and nonrobotic MVr groups while adjusting for potential confounders. Adjusted least squares means estimates with 95% CIs were calculated for each outcome. All models were adjusted for patient-level covariates including age, sex, race, ethnicity, year of procedure, admission type, and Elixhauser Comorbidity Index score, as well as hospital-level characteristics including geographic region, teaching status, urban versus rural setting, and bed size.

## Results

From 2019 to 2024, 26,479 patients underwent MVr procedures in the PHD. Of these, 1335 (5.0%) involved robotic-assisted surgery and 25,144 (95.0%) were performed without robotic assistance. Robotic cases were more likely to be isolated MVrs without concomitant procedures (58.4%) compared with nonrobotic cases (28.8%). After we excluded patients who underwent concomitant procedures, the final analytic cohort included 8025 patients with isolated MVr: 780 (9.7%) in the robotic group and 7245 (90.3%) in the nonrobotic group (Figure 1).

**Figure 1:**
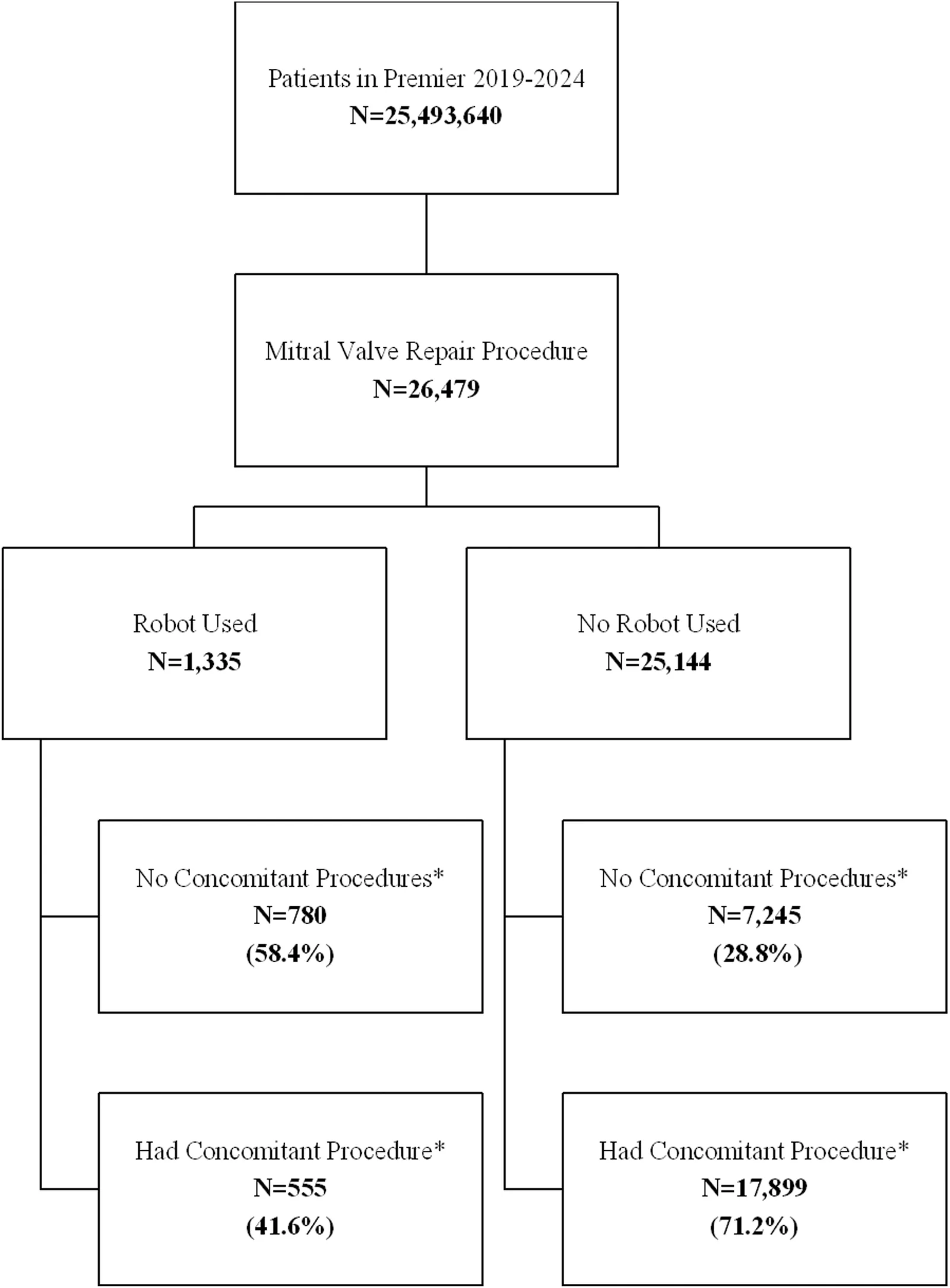
Attrition diagram. ∗Concomitant procedures included heart transplant, coronary artery bypass grafting, cardiac ablation, left atrial appendage closure, left ventricular assist device, maze, and aortic/tricuspid/pulmonic procedures.

Patient characteristics are presented in Table 1. Patients undergoing robotic-assisted repair were slightly older (mean age 59.8 vs 57.2 years) and more likely to be male (66.0% vs 60.0%) compared with those in the nonrobotic group. Racial distribution was similar between groups, with approximately 81% White in both cohorts. Patients in the robotic group were more likely to undergo elective procedures (93.3% vs 82.0%) and less likely to have Medicaid insurance (6.3% vs 11.3%). Comorbidity burden, as measured by the Elixhauser Comorbidity Index, was comparable between groups (4.32 vs 4.49). Although the distribution of robotic procedures across each year of the study is consistent, the proportion performed robotically actually doubled from 2019 to 2024. In 2019, robotic cases represented 7.7% of isolated repairs (136 robotic cases/[136 robotic + 1628 nonrobotic] = 136/1764). By 2024, this proportion had nearly doubled to 14.7% (120 robotic cases/[120 robotic + 699 non-robotic] = 120/819).

**Table 1:** Patient characteristics

Total patient visits	Robot	No robot	*P* value
	780	7245	
Age, y			<.0001
Mean (SD)	59.80 (12.20)	57.21 (17.75)	
Sex			.0011
Female	265 (33.97%)	2900 (40.03%)	
Male	515 (66.03%)	4344 (59.97%)	
Race			.0003
White	632 (81.03%)	5845 (80.68%)	
Black	51 (6.54%)	571 (7.88%)	
Asian	11 (1.41%)	251 (3.46%)	
Missing/unknown	86 (11.03%)	578 (7.98%)	
Year			<.0001
2019	136 (17.44%)	1628 (22.47%)	
2020	121 (15.51%)	1301 (17.96%)	
2021	113 (14.49%)	1239 (17.10%)	
2022	142 (18.21%)	1271 (17.54%)	
2023	148 (18.97%)	1107 (15.28%)	
2024	120 (15.38%)	699 (9.65%)	
Ethnicity			<.0001
Hispanic	25 (3.21%)	506 (6.98%)	
Not Hispanic	605 (77.56%)	6195 (85.51%)	
Missing/unknown	150 (19.23%)	544 (7.51%)	
Payor			.0002
Commercial	385 (49.36%)	3235 (44.65%)	
Medicaid	49 (6.28%)	817 (11.28%)	
Medicare	307 (39.36%)	2825 (38.99%)	
Missing/unknown	39 (5%)	368 (5.08%)	
Admission type			<.0001
Elective	728 (93.33%)	5944 (82.04%)	
Nonelective	42 (5.38%)	1242 (17.14%)	
Missing/unknown	10 (1.28%)	59 (0.81%)	
Elixhauser score			.0240
Mean (SD)	4.32 (1.95)	4.49 (2.15)	

Hospital characteristics differed between groups (Table 2). Robotic procedures were more frequently performed in the South (58.7% vs 35.0%) and less frequently in the Midwest (9.6% vs 25.8%) and Northeast (12.1% vs 23.1%). The majority of procedures were performed at academic centers in both groups, although this was more pronounced in the nonrobotic group (71.6% vs 54.7%). All robotic procedures were performed in urban settings, whereas 2.8% of nonrobotic procedures occurred in rural hospitals. Robotic cases were concentrated in larger hospitals, with 68.0% performed at facilities with 500 or more beds compared with 58.3% for nonrobotic cases.

**Table 2:** Hospital characteristics

Total patients	Robot	No robot	*P* value
	780	7245	
Region			<.0001
Midwest	75 (9.62%)	1872 (25.84%)	
Northeast	94 (12.05%)	1674 (23.11%)	
South	458 (58.72%)	2538 (35.03%)	
West	153 (19.62%)	1161 (16.02%)	
Teaching			<.0001
Academic	427 (54.74%)	5188 (71.61%)	
Community	353 (45.26%)	2057 (28.39%)	
Location			<.0001
Rural	0 (0%)	203 (2.80%)	
Urban	780 (100%)	7042 (97.20%)	
Bed size			<.0001
0-99	0 (0%)	439 (6.06%)	
100-199	103 (13.21%)	241 (3.33%)	
200-299	11 (1.41%)	568 (7.84%)	
300-399	45 (5.77%)	984 (13.58%)	
400-499	91 (11.67%)	786 (10.85%)	
500+	530 (67.95%)	4227 (58.34%)	

Adjusted outcomes are presented in Table 3. Clinical outcomes were similar between groups, with comparable rates of in-hospital mortality (0.3% vs 0.3%; *P* = .89) and prolonged mechanical ventilation (1.7% vs 2.2%; *P* = .34). Regarding resource use, operating room time was similar between groups (6.5 vs 6.3 hours; *P* = .28). However, robotic-assisted repair was associated with significantly shorter ICU length of stay (2.4 vs 2.8 days; *P* < .0001), shorter total hospital length of stay (5.7 vs 6.8 days; *P* < .0001), and lower 30-day CV readmissions (3.1% vs 4.9%; *P* = .02). Total hospital costs were significantly greater in the robotic group ($57,678 vs $51,530; *P* < .0001). However, when operating room costs were excluded, there was no significant difference in costs between groups ($36,791 vs $36,950; *P* = .83).

**Table 3:** Multivariable results

Outcome	Robot	No Robot	*P* value
Clinical outcomes			
In-hospital death	0.3% [0.1%, 0.8%]	0.3% [0.2%, 0.5%]	.8884
Prolonged ventilation	1.7% [1.0%, 2.8%]	2.2% [1.8%, 2.6%]	.3378
Resource use			
OR time	6.5 [6.2, 6.8]	6.3 [6.2, 6.4]	.2835
ICU stay, d	2.4 [2.2, 2.5]	2.8 [2.7, 2.9]	<.0001
LOS	5.7 [5.5, 6.0]	6.8 [6.7, 6.9]	<.0001
CV readmissions at 30 d	3.1% [2.1%, 4.6%]	4.9% [4.5%, 5.5%]	.0215
Hospital costs			
Costs	$57,678 [$55,754, $59,669]	$51,530 [$50,972, $52,093]	<.0001
Costs without OR	$36,791 [$35,428, $38,207]	$36,950 [$36,505, $37,399]	.8326

Values are reported as mean[SD].

*OR*, Operating room; *ICU*, intensive care unit; *LOS*, length of stay; *CV*, cardiovascular.

Examination of cost drivers revealed differences in the distribution of costs between groups (Figure 2). Operating room costs represented a larger proportion of total costs in the robotic group compared with the nonrobotic group (38% vs 27%), whereas room and board costs represented a smaller proportion (24% vs 34%), reflecting the shorter hospital length of stay observed with robotic-assisted surgery. Supply costs were similar between groups (20% vs 17%), as were pharmacy, laboratory, and other ancillary costs. Mean 30-day CV readmission costs, calculated across all patients including those without a readmission, were lower in the robotic group ($571 vs $1335), consistent with the reduced 30-day CV readmission rate.

**Figure 2:**
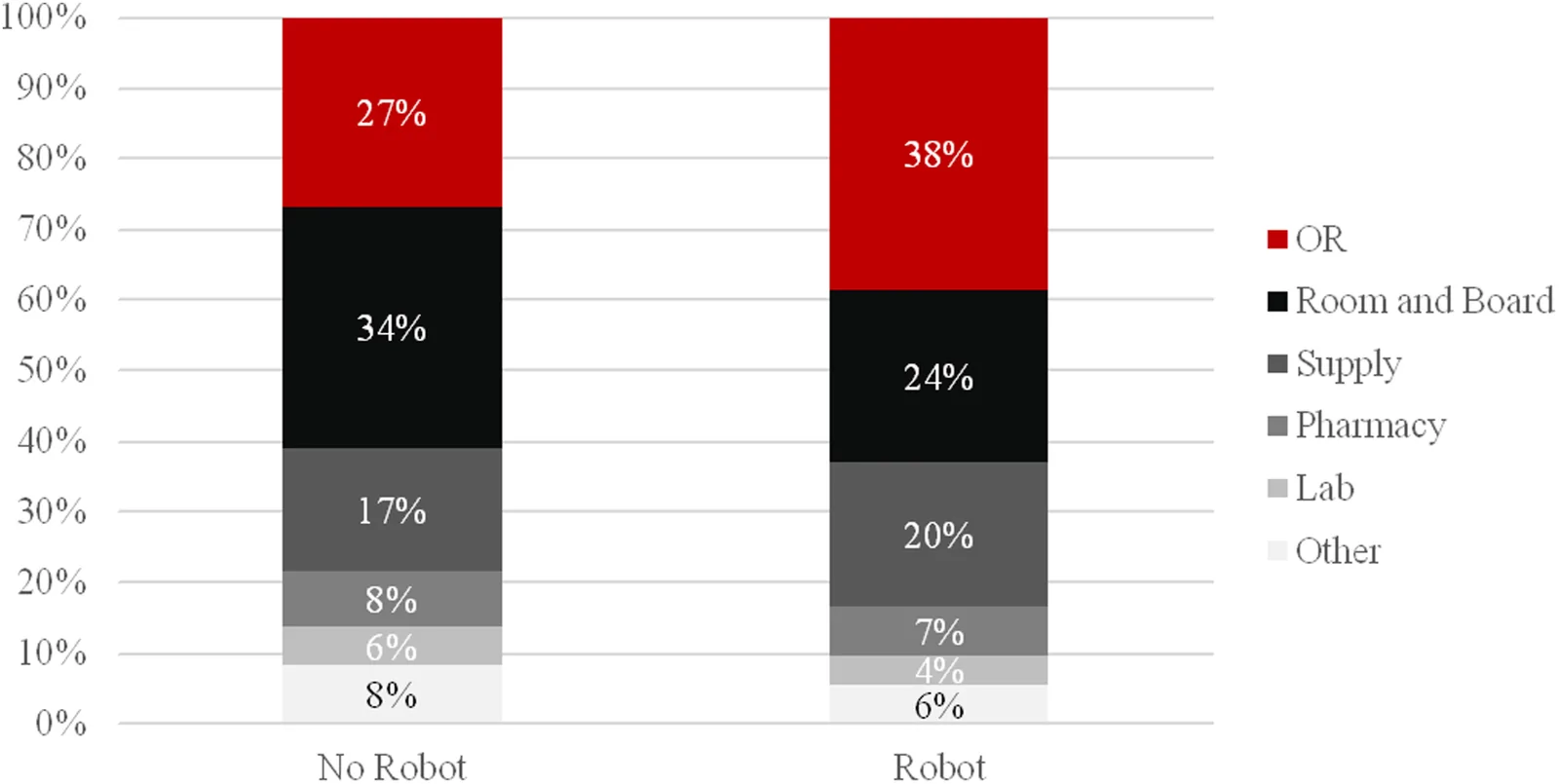
Cost drivers. *OR*, Operating room.

## Discussion

Robotic MVr continues to gain adoption, with our data demonstrating a near doubling of use from 7.7% in 2019 to 14.7% in 2024. This trend is consistent with national data from the Society of Thoracic Surgeons Adult Cardiac Surgery Database, which demonstrated an increase from 10.9% in 2015 to 14.6% in 2021.^10^ In this context, understanding real-world outcomes and costs becomes increasingly important. In this large, real-world analysis of more than 8000 patients undergoing isolated MVr, robotic-assisted surgery was associated with several clinical benefits compared with nonrobotic approaches. Patients in the robotic group experienced significantly shorter ICU length of stay, shorter total hospital length of stay, and lower 30-day CV readmission rates, with comparable rates of prolonged mechanical ventilation and in-hospital mortality. Although total hospital costs were significantly greater in the robotic group, operating room costs represented a greater proportion of total costs (38% vs 27%), reflecting the capital expenditures, maintenance, and disposable instrumentation associated with the robotic surgical system. Notably, room and board costs represented a smaller proportion of total costs in the robotic group (24% vs 34%), directly reflecting the shorter hospital length of stay and offsetting a portion of the increased operating room expenditures.

Our findings are consistent with the existing literature demonstrating the clinical benefits of robotic-assisted mitral valve repair. Recent meta-analyses have reported that robotic surgery is associated with significantly shorter ICU and hospital lengths of stay compared with conventional sternotomy approaches.^2^^,^^3^ Williams and colleagues^2^ reported that robotic mitral valve surgery was associated with a 26-hour reduction in ICU stay and a 2-day reduction in hospital length of stay compared with sternotomy. Similarly, Fu and colleagues^3^ found significantly lower blood transfusion rates and mortality with robotic approaches, despite longer operative and cardiopulmonary bypass times. Our observation of reduced 30-day CV readmissions in the robotic group aligns with previous single-center studies suggesting improved early recovery with minimally invasive techniques.

The cost findings in our study mirror those reported by Hadaya and colleagues^7^ in a recent national analysis using the Nationwide Readmissions Database, which found that robotic-assisted MVr was associated with greater inpatient costs compared with conventional surgery. However, similar to our findings, the authors noted that robotic procedures were associated with shorter lengths of stay and lower rates of discharge to skilled nursing facilities, which may offset some of the upfront cost differential when considering the total episode of care. Importantly, our analysis extends these findings by demonstrating that when operating room costs—which capture the capital and instrumentation costs of the robotic platform—are excluded, hospitalization costs are equivalent between approaches. This suggests that robotic-assisted surgery does not increase the downstream resource use associated with postoperative care. Furthermore, we found mean 30-day CV readmission costs lower in the robotic group, consistent with the reduced 30-day CV readmission rate.

Beyond the hospital setting, the shorter length of stay and reduced readmission rates observed with robotic-assisted surgery may confer broader societal benefits. Shorter hospitalizations allow patients to return to work and daily activities sooner, potentially improving economic productivity. Reduced time in the hospital may also decrease exposure to hospital-acquired infections and limit postoperative opioid requirements. In addition, shorter lengths of stay free hospital capacity for other patients, an increasingly important consideration, given constraints on health care resources.

### Study Limitations

This study has several limitations inherent to retrospective analyses of administrative data. First, despite multivariable adjustment, residual confounding from unmeasured variables may persist, and selection bias may exist as patients undergoing robotic surgery may differ systematically from those who do not. Second, the use of expert rules and Chargemaster text mining to identify robotic use may be subject to misclassification. Third, the PHD captures approximately 25% of US inpatient admissions and may not be fully generalizable to all practice settings. Fourth, administrative data lack granular clinical detail such as echocardiographic outcomes, repair durability, important clinical markers (ie, Society of Thoracic Surgeons Predicted Risk of Mortality score), conversion from robotic to sternotomy, crossclamp and bypass times, and long-term follow-up. Finally, patients cannot be tracked across different hospital systems, limiting our ability to capture readmissions and other clinical outcomes occurring at nonindex hospitals.

## Conclusions

In this real-world analysis of 8025 patients undergoing isolated MVr, robotic-assisted surgery was associated with shorter ICU and hospital lengths of stay, lower 30-day CV readmission rates, readmission costs, and comparable mortality compared with nonrobotic approaches. Although total hospital costs were greater with robotic surgery, this difference was attributable to operating room costs related to the robotic platform; hospitalization costs excluding the operating room were equivalent between groups. These findings support robotic-assisted MVr as a safe and effective approach that may offer meaningful benefits in postoperative recovery and resource use.

### Webcast

You can watch a Webcast of this AATS meeting presentation by going to: https://www.aats.org/resources/isolated-mitral-valve-repair-i-12535.

**Figure:**
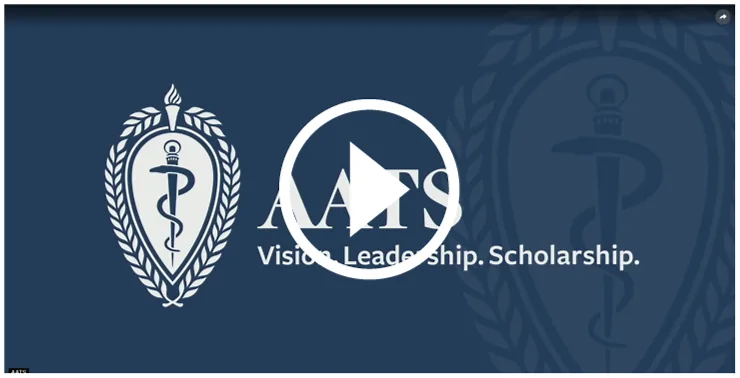

## Conflict of Interest Statement

Dr Hashimoto is a consultant for Intuitive and Edwards Lifesciences. Dr Reifenberger is an employee of Edwards Lifesciences. S. Murphy is an employee of Edwards Lifesciences. Dr Nguyen has performing speaking engagements for Edwards Lifesciences, Abbott, and Medtronic. Dr Nakamura reported no conflicts of interest.

The *Journal* policy requires editors and reviewers to disclose conflicts of interest and to decline handling or reviewing manuscripts for which they may have a conflict of interest. The editors and reviewers of this article have no conflicts of interest.
