# Supplementary material for: Health economic and hospital resource use in isolated mitral valve repair in more than 8000 patients: Robotic versus nonrobotic approaches
Source: JTCVS Open. 2026 Aug 19;33:102067. doi: 10.1016/j.xjon.2026.102067 (PMC13711181; doi:10.1016/j.xjon.2026.102067)
Supplement: Supplementary file 1 [file mmc1.docx]

**APPENDIX A – Mitral Valve Repair**

| **Variable** | **Code** | **Description** |
| --- | --- | --- |
| Mitral Valve Repair | 02QG0ZE | Repair Mitral Valve created from Left Atrioventricular Valve, Open Approach |
| Mitral Valve Repair | 02QG0ZZ | Repair Mitral Valve, Open Approach |
| Mitral Valve Repair | 02QG4ZE | Repair Mitral Valve created from Left Atrioventricular Valve, Percutaneous Endoscopic Approach |
| Mitral Valve Repair | 02QG4ZZ | Repair Mitral Valve, Percutaneous Endoscopic Approach |
| Mitral Valve Repair | 02UG07E | Supplement Mitral Valve created from Left Atrioventricular Valve with Autologous Tissue Substitute, Open Approach |
| Mitral Valve Repair | 02UG07Z | Supplement Mitral Valve with Autologous Tissue Substitute, Open Approach |
| Mitral Valve Repair | 02UG08E | Supplement Mitral Valve created from Left Atrioventricular Valve with Zooplastic Tissue, Open Approach |
| Mitral Valve Repair | 02UG08Z | Supplement Mitral Valve with Zooplastic Tissue, Open Approach |
| Mitral Valve Repair | 02UG0JE | Supplement Mitral Valve created from Left Atrioventricular Valve with Synthetic Substitute, Open Approach |
| Mitral Valve Repair | 02UG0JZ | Supplement Mitral Valve with Synthetic Substitute, Open Approach |
| Mitral Valve Repair | 02UG0KE | Supplement Mitral Valve created from Left Atrioventricular Valve with Nonautologous Tissue Substitute, Open Approach |
| Mitral Valve Repair | 02UG0KZ | Supplement Mitral Valve with Nonautologous Tissue Substitute, Open Approach |
| Mitral Valve Repair | 02UG47E | Supplement Mitral Valve created from Left Atrioventricular Valve with Autologous Tissue Substitute, Percutaneous Endoscopic Approach |
| Mitral Valve Repair | 02UG47Z | Supplement Mitral Valve with Autologous Tissue Substitute, Percutaneous Endoscopic Approach |
| Mitral Valve Repair | 02UG48E | Supplement Mitral Valve created from Left Atrioventricular Valve with Zooplastic Tissue, Percutaneous Endoscopic Approach |
| Mitral Valve Repair | 02UG48Z | Supplement Mitral Valve with Zooplastic Tissue, Percutaneous Endoscopic Approach |
| Mitral Valve Repair | 02UG4JE | Supplement Mitral Valve created from Left Atrioventricular Valve with Synthetic Substitute, Percutaneous Endoscopic Approach |
| Mitral Valve Repair | 02UG4JZ | Supplement Mitral Valve with Synthetic Substitute, Percutaneous Endoscopic Approach |
| Mitral Valve Repair | 02UG4KE | Supplement Mitral Valve created from Left Atrioventricular Valve with Nonautologous Tissue Substitute, Percutaneous Endoscopic Approach |
| Mitral Valve Repair | 02UG4KZ | Supplement Mitral Valve with Nonautologous Tissue Substitute, Percutaneous Endoscopic Approach |
| Mitral Valve Repair | 02WG07Z | Revision of Autologous Tissue Substitute in Mitral Valve, Open Approach |
| Mitral Valve Repair | 02WG08Z | Revision of Zooplastic Tissue in Mitral Valve, Open Approach |
| Mitral Valve Repair | 02WG0JZ | Revision of Synthetic Substitute in Mitral Valve, Open Approach |
| Mitral Valve Repair | 02WG0KZ | Revision of Nonautologous Tissue Substitute in Mitral Valve, Open Approach |
| Mitral Valve Repair | 02WG47Z | Revision of Autologous Tissue Substitute in Mitral Valve, Percutaneous Endoscopic Approach |
| Mitral Valve Repair | 02WG48Z | Revision of Zooplastic Tissue in Mitral Valve, Percutaneous Endoscopic Approach |
| Mitral Valve Repair | 02WG4JZ | Revision of Synthetic Substitute in Mitral Valve, Percutaneous Endoscopic Approach |
| Mitral Valve Repair | 02WG4KZ | Revision of Nonautologous Tissue Substitute in Mitral Valve, Percutaneous Endoscopic Approach |

**Appendix B – Concomitant Procedures**

| **Variable** | **Code** | **Description** |
| --- | --- | --- |
| Heart Transplant | 02YA0Z0 | Transplantation of Heart, Allogeneic, Open Approach |
| Heart Transplant | 02YA0Z1 | Transplantation of Heart, Syngeneic, Open Approach |
| Heart Transplant | 02YA0Z2 | Transplantation of Heart, Zooplastic, Open Approach |
| CABG | 0210088 | Bypass Coronary Artery, One Artery from Right Internal Mammary with Zooplastic Tissue, Open Approach |
| CABG | 0210089 | Bypass Coronary Artery, One Artery from Left Internal Mammary with Zooplastic Tissue, Open Approach |
| CABG | 021008C | Bypass Coronary Artery, One Artery from Thoracic Artery with Zooplastic Tissue, Open Approach |
| CABG | 021008W | Bypass Coronary Artery, One Artery from Aorta with Zooplastic Tissue, Open Approach |
| CABG | 0210093 | Bypass Coronary Artery, One Artery from Coronary Artery with Autologous Venous Tissue, Open Approach |
| CABG | 0210098 | Bypass Coronary Artery, One Artery from Right Internal Mammary with Autologous Venous Tissue, Open Approach |
| CABG | 0210099 | Bypass Coronary Artery, One Artery from Left Internal Mammary with Autologous Venous Tissue, Open Approach |
| CABG | 021009C | Bypass Coronary Artery, One Artery from Thoracic Artery with Autologous Venous Tissue, Open Approach |
| CABG | 021009W | Bypass Coronary Artery, One Artery from Aorta with Autologous Venous Tissue, Open Approach |
| CABG | 02100A3 | Bypass Coronary Artery, One Artery from Coronary Artery with Autologous Arterial Tissue, Open Approach |
| CABG | 02100A8 | Bypass Coronary Artery, One Artery from Right Internal Mammary with Autologous Arterial Tissue, Open Approach |
| CABG | 02100A9 | Bypass Coronary Artery, One Artery from Left Internal Mammary with Autologous Arterial Tissue, Open Approach |
| CABG | 02100AC | Bypass Coronary Artery, One Artery from Thoracic Artery with Autologous Arterial Tissue, Open Approach |
| CABG | 02100AW | Bypass Coronary Artery, One Artery from Aorta with Autologous Arterial Tissue, Open Approach |
| CABG | 02100J3 | Bypass Coronary Artery, One Artery from Coronary Artery with Synthetic Substitute, Open Approach |
| CABG | 02100J8 | Bypass Coronary Artery, One Artery from Right Internal Mammary with Synthetic Substitute, Open Approach |
| CABG | 02100J9 | Bypass Coronary Artery, One Artery from Left Internal Mammary with Synthetic Substitute, Open Approach |
| CABG | 02100JC | Bypass Coronary Artery, One Artery from Thoracic Artery with Synthetic Substitute, Open Approach |
| CABG | 02100JW | Bypass Coronary Artery, One Artery from Aorta with Synthetic Substitute, Open Approach |
| CABG | 02100K3 | Bypass Coronary Artery, One Artery from Coronary Artery with Nonautologous Tissue Substitute, Open Approach |
| CABG | 02100K8 | Bypass Coronary Artery, One Artery from Right Internal Mammary with Nonautologous Tissue Substitute, Open Approach |
| CABG | 02100K9 | Bypass Coronary Artery, One Artery from Left Internal Mammary with Nonautologous Tissue Substitute, Open Approach |
| CABG | 02100KC | Bypass Coronary Artery, One Artery from Thoracic Artery with Nonautologous Tissue Substitute, Open Approach |
| CABG | 02100KW | Bypass Coronary Artery, One Artery from Aorta with Nonautologous Tissue Substitute, Open Approach |
| CABG | 02100Z3 | Bypass Coronary Artery, One Artery from Coronary Artery, Open Approach |
| CABG | 02100Z8 | Bypass Coronary Artery, One Artery from Right Internal Mammary, Open Approach |
| CABG | 02100Z9 | Bypass Coronary Artery, One Artery from Left Internal Mammary, Open Approach |
| CABG | 02100ZC | Bypass Coronary Artery, One Artery from Thoracic Artery, Open Approach |
| CABG | 0210488 | Bypass Coronary Artery, One Artery from Right Internal Mammary with Zooplastic Tissue, Percutaneous Endoscopic Approach |
| CABG | 0210489 | Bypass Coronary Artery, One Artery from Left Internal Mammary with Zooplastic Tissue, Percutaneous Endoscopic Approach |
| CABG | 021048C | Bypass Coronary Artery, One Artery from Thoracic Artery with Zooplastic Tissue, Percutaneous Endoscopic Approach |
| CABG | 021048W | Bypass Coronary Artery, One Artery from Aorta with Zooplastic Tissue, Percutaneous Endoscopic Approach |
| CABG | 0210493 | Bypass Coronary Artery, One Artery from Coronary Artery with Autologous Venous Tissue, Percutaneous Endoscopic Approach |
| CABG | 0210498 | Bypass Coronary Artery, One Artery from Right Internal Mammary with Autologous Venous Tissue, Percutaneous Endoscopic Approach |
| CABG | 0210499 | Bypass Coronary Artery, One Artery from Left Internal Mammary with Autologous Venous Tissue, Percutaneous Endoscopic Approach |
| CABG | 021049C | Bypass Coronary Artery, One Artery from Thoracic Artery with Autologous Venous Tissue, Percutaneous Endoscopic Approach |
| CABG | 021049W | Bypass Coronary Artery, One Artery from Aorta with Autologous Venous Tissue, Percutaneous Endoscopic Approach |
| CABG | 02104A3 | Bypass Coronary Artery, One Artery from Coronary Artery with Autologous Arterial Tissue, Percutaneous Endoscopic Approach |
| CABG | 02104A8 | Bypass Coronary Artery, One Artery from Right Internal Mammary with Autologous Arterial Tissue, Percutaneous Endoscopic Approach |
| CABG | 02104A9 | Bypass Coronary Artery, One Artery from Left Internal Mammary with Autologous Arterial Tissue, Percutaneous Endoscopic Approach |
| CABG | 02104AC | Bypass Coronary Artery, One Artery from Thoracic Artery with Autologous Arterial Tissue, Percutaneous Endoscopic Approach |
| CABG | 02104AW | Bypass Coronary Artery, One Artery from Aorta with Autologous Arterial Tissue, Percutaneous Endoscopic Approach |
| CABG | 02104J3 | Bypass Coronary Artery, One Artery from Coronary Artery with Synthetic Substitute, Percutaneous Endoscopic Approach |
| CABG | 02104J8 | Bypass Coronary Artery, One Artery from Right Internal Mammary with Synthetic Substitute, Percutaneous Endoscopic Approach |
| CABG | 02104J9 | Bypass Coronary Artery, One Artery from Left Internal Mammary with Synthetic Substitute, Percutaneous Endoscopic Approach |
| CABG | 02104JC | Bypass Coronary Artery, One Artery from Thoracic Artery with Synthetic Substitute, Percutaneous Endoscopic Approach |
| CABG | 02104JW | Bypass Coronary Artery, One Artery from Aorta with Synthetic Substitute, Percutaneous Endoscopic Approach |
| CABG | 02104K3 | Bypass Coronary Artery, One Artery from Coronary Artery with Nonautologous Tissue Substitute, Percutaneous Endoscopic Approach |
| CABG | 02104K8 | Bypass Coronary Artery, One Artery from Right Internal Mammary with Nonautologous Tissue Substitute, Percutaneous Endoscopic Approach |
| CABG | 02104K9 | Bypass Coronary Artery, One Artery from Left Internal Mammary with Nonautologous Tissue Substitute, Percutaneous Endoscopic Approach |
| CABG | 02104KC | Bypass Coronary Artery, One Artery from Thoracic Artery with Nonautologous Tissue Substitute, Percutaneous Endoscopic Approach |
| CABG | 02104KW | Bypass Coronary Artery, One Artery from Aorta with Nonautologous Tissue Substitute, Percutaneous Endoscopic Approach |
| CABG | 02104Z3 | Bypass Coronary Artery, One Artery from Coronary Artery, Percutaneous Endoscopic Approach |
| CABG | 02104Z8 | Bypass Coronary Artery, One Artery from Right Internal Mammary, Percutaneous Endoscopic Approach |
| CABG | 02104Z9 | Bypass Coronary Artery, One Artery from Left Internal Mammary, Percutaneous Endoscopic Approach |
| CABG | 02104ZC | Bypass Coronary Artery, One Artery from Thoracic Artery, Percutaneous Endoscopic Approach |
| CABG | 0211088 | Bypass Coronary Artery, Two Arteries from Right Internal Mammary with Zooplastic Tissue, Open Approach |
| CABG | 0211089 | Bypass Coronary Artery, Two Arteries from Left Internal Mammary with Zooplastic Tissue, Open Approach |
| CABG | 021108C | Bypass Coronary Artery, Two Arteries from Thoracic Artery with Zooplastic Tissue, Open Approach |
| CABG | 021108W | Bypass Coronary Artery, Two Arteries from Aorta with Zooplastic Tissue, Open Approach |
| CABG | 0211098 | Bypass Coronary Artery, Two Arteries from Right Internal Mammary with Autologous Venous Tissue, Open Approach |
| CABG | 0211099 | Bypass Coronary Artery, Two Arteries from Left Internal Mammary with Autologous Venous Tissue, Open Approach |
| CABG | 021109C | Bypass Coronary Artery, Two Arteries from Thoracic Artery with Autologous Venous Tissue, Open Approach |
| CABG | 021109W | Bypass Coronary Artery, Two Arteries from Aorta with Autologous Venous Tissue, Open Approach |
| CABG | 02110A8 | Bypass Coronary Artery, Two Arteries from Right Internal Mammary with Autologous Arterial Tissue, Open Approach |
| CABG | 02110A9 | Bypass Coronary Artery, Two Arteries from Left Internal Mammary with Autologous Arterial Tissue, Open Approach |
| CABG | 02110AC | Bypass Coronary Artery, Two Arteries from Thoracic Artery with Autologous Arterial Tissue, Open Approach |
| CABG | 02110AW | Bypass Coronary Artery, Two Arteries from Aorta with Autologous Arterial Tissue, Open Approach |
| CABG | 02110J8 | Bypass Coronary Artery, Two Arteries from Right Internal Mammary with Synthetic Substitute, Open Approach |
| CABG | 02110J9 | Bypass Coronary Artery, Two Arteries from Left Internal Mammary with Synthetic Substitute, Open Approach |
| CABG | 02110JC | Bypass Coronary Artery, Two Arteries from Thoracic Artery with Synthetic Substitute, Open Approach |
| CABG | 02110JW | Bypass Coronary Artery, Two Arteries from Aorta with Synthetic Substitute, Open Approach |
| CABG | 02110K8 | Bypass Coronary Artery, Two Arteries from Right Internal Mammary with Nonautologous Tissue Substitute, Open Approach |
| CABG | 02110K9 | Bypass Coronary Artery, Two Arteries from Left Internal Mammary with Nonautologous Tissue Substitute, Open Approach |
| CABG | 02110KC | Bypass Coronary Artery, Two Arteries from Thoracic Artery with Nonautologous Tissue Substitute, Open Approach |
| CABG | 02110KW | Bypass Coronary Artery, Two Arteries from Aorta with Nonautologous Tissue Substitute, Open Approach |
| CABG | 02110Z8 | Bypass Coronary Artery, Two Arteries from Right Internal Mammary, Open Approach |
| CABG | 02110Z9 | Bypass Coronary Artery, Two Arteries from Left Internal Mammary, Open Approach |
| CABG | 02110ZC | Bypass Coronary Artery, Two Arteries from Thoracic Artery, Open Approach |
| CABG | 0211488 | Bypass Coronary Artery, Two Arteries from Right Internal Mammary with Zooplastic Tissue, Percutaneous Endoscopic Approach |
| CABG | 0211489 | Bypass Coronary Artery, Two Arteries from Left Internal Mammary with Zooplastic Tissue, Percutaneous Endoscopic Approach |
| CABG | 021148C | Bypass Coronary Artery, Two Arteries from Thoracic Artery with Zooplastic Tissue, Percutaneous Endoscopic Approach |
| CABG | 021148W | Bypass Coronary Artery, Two Arteries from Aorta with Zooplastic Tissue, Percutaneous Endoscopic Approach |
| CABG | 0211498 | Bypass Coronary Artery, Two Arteries from Right Internal Mammary with Autologous Venous Tissue, Percutaneous Endoscopic Approach |
| CABG | 0211499 | Bypass Coronary Artery, Two Arteries from Left Internal Mammary with Autologous Venous Tissue, Percutaneous Endoscopic Approach |
| CABG | 021149C | Bypass Coronary Artery, Two Arteries from Thoracic Artery with Autologous Venous Tissue, Percutaneous Endoscopic Approach |
| CABG | 021149W | Bypass Coronary Artery, Two Arteries from Aorta with Autologous Venous Tissue, Percutaneous Endoscopic Approach |
| CABG | 02114A8 | Bypass Coronary Artery, Two Arteries from Right Internal Mammary with Autologous Arterial Tissue, Percutaneous Endoscopic Approach |
| CABG | 02114A9 | Bypass Coronary Artery, Two Arteries from Left Internal Mammary with Autologous Arterial Tissue, Percutaneous Endoscopic Approach |
| CABG | 02114AC | Bypass Coronary Artery, Two Arteries from Thoracic Artery with Autologous Arterial Tissue, Percutaneous Endoscopic Approach |
| CABG | 02114AW | Bypass Coronary Artery, Two Arteries from Aorta with Autologous Arterial Tissue, Percutaneous Endoscopic Approach |
| CABG | 02114J8 | Bypass Coronary Artery, Two Arteries from Right Internal Mammary with Synthetic Substitute, Percutaneous Endoscopic Approach |
| CABG | 02114J9 | Bypass Coronary Artery, Two Arteries from Left Internal Mammary with Synthetic Substitute, Percutaneous Endoscopic Approach |
| CABG | 02114JC | Bypass Coronary Artery, Two Arteries from Thoracic Artery with Synthetic Substitute, Percutaneous Endoscopic Approach |
| CABG | 02114JW | Bypass Coronary Artery, Two Arteries from Aorta with Synthetic Substitute, Percutaneous Endoscopic Approach |
| CABG | 02114K8 | Bypass Coronary Artery, Two Arteries from Right Internal Mammary with Nonautologous Tissue Substitute, Percutaneous Endoscopic Approach |
| CABG | 02114K9 | Bypass Coronary Artery, Two Arteries from Left Internal Mammary with Nonautologous Tissue Substitute, Percutaneous Endoscopic Approach |
| CABG | 02114KC | Bypass Coronary Artery, Two Arteries from Thoracic Artery with Nonautologous Tissue Substitute, Percutaneous Endoscopic Approach |
| CABG | 02114KW | Bypass Coronary Artery, Two Arteries from Aorta with Nonautologous Tissue Substitute, Percutaneous Endoscopic Approach |
| CABG | 02114Z8 | Bypass Coronary Artery, Two Arteries from Right Internal Mammary, Percutaneous Endoscopic Approach |
| CABG | 02114Z9 | Bypass Coronary Artery, Two Arteries from Left Internal Mammary, Percutaneous Endoscopic Approach |
| CABG | 02114ZC | Bypass Coronary Artery, Two Arteries from Thoracic Artery, Percutaneous Endoscopic Approach |
| CABG | 021208C | Bypass Coronary Artery, Three Arteries from Thoracic Artery with Zooplastic Tissue, Open Approach |
| CABG | 021208W | Bypass Coronary Artery, Three Arteries from Aorta with Zooplastic Tissue, Open Approach |
| CABG | 021209C | Bypass Coronary Artery, Three Arteries from Thoracic Artery with Autologous Venous Tissue, Open Approach |
| CABG | 021209W | Bypass Coronary Artery, Three Arteries from Aorta with Autologous Venous Tissue, Open Approach |
| CABG | 02120AC | Bypass Coronary Artery, Three Arteries from Thoracic Artery with Autologous Arterial Tissue, Open Approach |
| CABG | 02120AW | Bypass Coronary Artery, Three Arteries from Aorta with Autologous Arterial Tissue, Open Approach |
| CABG | 02120JC | Bypass Coronary Artery, Three Arteries from Thoracic Artery with Synthetic Substitute, Open Approach |
| CABG | 02120JW | Bypass Coronary Artery, Three Arteries from Aorta with Synthetic Substitute, Open Approach |
| CABG | 02120KC | Bypass Coronary Artery, Three Arteries from Thoracic Artery with Nonautologous Tissue Substitute, Open Approach |
| CABG | 02120KW | Bypass Coronary Artery, Three Arteries from Aorta with Nonautologous Tissue Substitute, Open Approach |
| CABG | 02120ZC | Bypass Coronary Artery, Three Arteries from Thoracic Artery, Open Approach |
| CABG | 021248C | Bypass Coronary Artery, Three Arteries from Thoracic Artery with Zooplastic Tissue, Percutaneous Endoscopic Approach |
| CABG | 021248W | Bypass Coronary Artery, Three Arteries from Aorta with Zooplastic Tissue, Percutaneous Endoscopic Approach |
| CABG | 021249C | Bypass Coronary Artery, Three Arteries from Thoracic Artery with Autologous Venous Tissue, Percutaneous Endoscopic Approach |
| CABG | 021249W | Bypass Coronary Artery, Three Arteries from Aorta with Autologous Venous Tissue, Percutaneous Endoscopic Approach |
| CABG | 02124AC | Bypass Coronary Artery, Three Arteries from Thoracic Artery with Autologous Arterial Tissue, Percutaneous Endoscopic Approach |
| CABG | 02124AW | Bypass Coronary Artery, Three Arteries from Aorta with Autologous Arterial Tissue, Percutaneous Endoscopic Approach |
| CABG | 02124JC | Bypass Coronary Artery, Three Arteries from Thoracic Artery with Synthetic Substitute, Percutaneous Endoscopic Approach |
| CABG | 02124JW | Bypass Coronary Artery, Three Arteries from Aorta with Synthetic Substitute, Percutaneous Endoscopic Approach |
| CABG | 02124KC | Bypass Coronary Artery, Three Arteries from Thoracic Artery with Nonautologous Tissue Substitute, Percutaneous Endoscopic Approach |
| CABG | 02124KW | Bypass Coronary Artery, Three Arteries from Aorta with Nonautologous Tissue Substitute, Percutaneous Endoscopic Approach |
| CABG | 02124ZC | Bypass Coronary Artery, Three Arteries from Thoracic Artery, Percutaneous Endoscopic Approach |
| CABG | 021308C | Bypass Coronary Artery, Four or More Arteries from Thoracic Artery with Zooplastic Tissue, Open Approach |
| CABG | 021308W | Bypass Coronary Artery, Four or More Arteries from Aorta with Zooplastic Tissue, Open Approach |
| CABG | 021309C | Bypass Coronary Artery, Four or More Arteries from Thoracic Artery with Autologous Venous Tissue, Open Approach |
| CABG | 021309W | Bypass Coronary Artery, Four or More Arteries from Aorta with Autologous Venous Tissue, Open Approach |
| CABG | 02130AC | Bypass Coronary Artery, Four or More Arteries from Thoracic Artery with Autologous Arterial Tissue, Open Approach |
| CABG | 02130AW | Bypass Coronary Artery, Four or More Arteries from Aorta with Autologous Arterial Tissue, Open Approach |
| CABG | 02130JC | Bypass Coronary Artery, Four or More Arteries from Thoracic Artery with Synthetic Substitute, Open Approach |
| CABG | 02130JW | Bypass Coronary Artery, Four or More Arteries from Aorta with Synthetic Substitute, Open Approach |
| CABG | 02130KC | Bypass Coronary Artery, Four or More Arteries from Thoracic Artery with Nonautologous Tissue Substitute, Open Approach |
| CABG | 02130KW | Bypass Coronary Artery, Four or More Arteries from Aorta with Nonautologous Tissue Substitute, Open Approach |
| CABG | 02130ZC | Bypass Coronary Artery, Four or More Arteries from Thoracic Artery, Open Approach |
| CABG | 021348C | Bypass Coronary Artery, Four or More Arteries from Thoracic Artery with Zooplastic Tissue, Percutaneous Endoscopic Approach |
| CABG | 021348W | Bypass Coronary Artery, Four or More Arteries from Aorta with Zooplastic Tissue, Percutaneous Endoscopic Approach |
| CABG | 021349C | Bypass Coronary Artery, Four or More Arteries from Thoracic Artery with Autologous Venous Tissue, Percutaneous Endoscopic Approach |
| CABG | 021349W | Bypass Coronary Artery, Four or More Arteries from Aorta with Autologous Venous Tissue, Percutaneous Endoscopic Approach |
| CABG | 02134AC | Bypass Coronary Artery, Four or More Arteries from Thoracic Artery with Autologous Arterial Tissue, Percutaneous Endoscopic Approach |
| CABG | 02134AW | Bypass Coronary Artery, Four or More Arteries from Aorta with Autologous Arterial Tissue, Percutaneous Endoscopic Approach |
| CABG | 02134JC | Bypass Coronary Artery, Four or More Arteries from Thoracic Artery with Synthetic Substitute, Percutaneous Endoscopic Approach |
| CABG | 02134JW | Bypass Coronary Artery, Four or More Arteries from Aorta with Synthetic Substitute, Percutaneous Endoscopic Approach |
| CABG | 02134KC | Bypass Coronary Artery, Four or More Arteries from Thoracic Artery with Nonautologous Tissue Substitute, Percutaneous Endoscopic Approach |
| CABG | 02134KW | Bypass Coronary Artery, Four or More Arteries from Aorta with Nonautologous Tissue Substitute, Percutaneous Endoscopic Approach |
| CABG | 02134ZC | Bypass Coronary Artery, Four or More Arteries from Thoracic Artery, Percutaneous Endoscopic Approach |
| Cardiac Ablation | 02580ZZ | Destruction of Conduction Mechanism, Open Approach |
| Cardiac Ablation | 02583ZZ | Destruction of Conduction Mechanism, Percutaneous Approach |
| Cardiac Ablation | 02584ZZ | Destruction of Conduction Mechanism, Percutaneous Endoscopic Approach |
| LAAC | 02L70CK | Occlusion of Left Atrial Appendage with Extraluminal Device, Open Approach |
| LAAC | 02L70DK | Occlusion of Left Atrial Appendage with Intraluminal Device, Open Approach |
| LAAC | 02L70ZK | Occlusion of Left Atrial Appendage, Open Approach |
| LAAC | 02L73CK | Occlusion of Left Atrial Appendage with Extraluminal Device, Percutaneous Approach |
| LAAC | 02L73DK | Occlusion of Left Atrial Appendage with Intraluminal Device, Percutaneous Approach |
| LAAC | 02L73ZK | Occlusion of Left Atrial Appendage, Percutaneous Approach |
| LAAC | 02L74CK | Occlusion of Left Atrial Appendage with Extraluminal Device, Percutaneous Endoscopic Approach |
| LAAC | 02L74DK | Occlusion of Left Atrial Appendage with Intraluminal Device, Percutaneous Endoscopic Approach |
| LAAC | 02L74ZK | Occlusion of Left Atrial Appendage, Percutaneous Endoscopic Approach |
| LVAD | 02HA0QZ | Insertion of Implantable Heart Assist System into Heart, Open Approach |
| LVAD | 02HA0RJ | Insertion of Short-term External Heart Assist System into Heart, Intraoperative, Open Approach |
| LVAD | 02HA0RS | Insertion of Biventricular Short-term External Heart Assist System into Heart, Open Approach |
| LVAD | 02HA0RZ | Insertion of Short-term External Heart Assist System into Heart, Open Approach |
| LVAD | 02HA3QZ | Insertion of Implantable Heart Assist System into Heart, Percutaneous Approach |
| LVAD | 02HA3RJ | Insertion of Short-term External Heart Assist System into Heart, Intraoperative, Percutaneous Approach |
| LVAD | 02HA3RS | Insertion of Biventricular Short-term External Heart Assist System into Heart, Percutaneous Approach |
| LVAD | 02HA3RZ | Insertion of Short-term External Heart Assist System into Heart, Percutaneous Approach |
| LVAD | 02HA4QZ | Insertion of Implantable Heart Assist System into Heart, Percutaneous Endoscopic Approach |
| LVAD | 02HA4RJ | Insertion of Short-term External Heart Assist System into Heart, Intraoperative, Percutaneous Endoscopic Approach |
| LVAD | 02HA4RS | Insertion of Biventricular Short-term External Heart Assist System into Heart, Percutaneous Endoscopic Approach |
| LVAD | 02HA4RZ | Insertion of Short-term External Heart Assist System into Heart, Percutaneous Endoscopic Approach |
| Maze | 02560ZZ | Destruction of Right Atrium, Open Approach |
| Maze | 02570ZZ | Destruction of Left Atrium, Open Approach |
| Maze | 025K0ZZ | Destruction of Right Ventricle, Open Approach |
| Maze | 025L0ZZ | Destruction of Left Ventricle, Open Approach |
| Maze | 02B60ZZ | Excision of Right Atrium, Open Approach |
| Maze | 02B70ZZ | Excision of Left Atrium, Open Approach |
| Maze | 02BK0ZZ | Excision of Right Ventricle, Open Approach |
| Maze | 02BL0ZZ | Excision of Left Ventricle, Open Approach |
| Maze | 02T80ZZ | Resection of Conduction Mechanism, Open Approach |
| Aortic Valve Procedures | 024F07J | Creation of Aortic Valve from Truncal Valve using Autologous Tissue Substitute, Open Approach |
| Aortic Valve Procedures | 024F08J | Creation of Aortic Valve from Truncal Valve using Zooplastic Tissue, Open Approach |
| Aortic Valve Procedures | 024F0JJ | Creation of Aortic Valve from Truncal Valve using Synthetic Substitute, Open Approach |
| Aortic Valve Procedures | 024F0KJ | Creation of Aortic Valve from Truncal Valve using Nonautologous Tissue Substitute, Open Approach |
| Aortic Valve Procedures | 025F0ZZ | Destruction of Aortic Valve, Open Approach |
| Aortic Valve Procedures | 025F3ZZ | Destruction of Aortic Valve, Percutaneous Approach |
| Aortic Valve Procedures | 025F4ZZ | Destruction of Aortic Valve, Percutaneous Endoscopic Approach |
| Aortic Valve Procedures | 027F04Z | Dilation of Aortic Valve with Drug-eluting Intraluminal Device, Open Approach |
| Aortic Valve Procedures | 027F0DZ | Dilation of Aortic Valve with Intraluminal Device, Open Approach |
| Aortic Valve Procedures | 027F0ZZ | Dilation of Aortic Valve, Open Approach |
| Aortic Valve Procedures | 027F34Z | Dilation of Aortic Valve with Drug-eluting Intraluminal Device, Percutaneous Approach |
| Aortic Valve Procedures | 027F3DZ | Dilation of Aortic Valve with Intraluminal Device, Percutaneous Approach |
| Aortic Valve Procedures | 027F3ZZ | Dilation of Aortic Valve, Percutaneous Approach |
| Aortic Valve Procedures | 027F44Z | Dilation of Aortic Valve with Drug-eluting Intraluminal Device, Percutaneous Endoscopic Approach |
| Aortic Valve Procedures | 027F4DZ | Dilation of Aortic Valve with Intraluminal Device, Percutaneous Endoscopic Approach |
| Aortic Valve Procedures | 027F4ZZ | Dilation of Aortic Valve, Percutaneous Endoscopic Approach |
| Aortic Valve Procedures | 02BF0ZX | Excision of Aortic Valve, Open Approach, Diagnostic |
| Aortic Valve Procedures | 02BF0ZZ | Excision of Aortic Valve, Open Approach |
| Aortic Valve Procedures | 02BF3ZX | Excision of Aortic Valve, Percutaneous Approach, Diagnostic |
| Aortic Valve Procedures | 02BF3ZZ | Excision of Aortic Valve, Percutaneous Approach |
| Aortic Valve Procedures | 02BF4ZX | Excision of Aortic Valve, Percutaneous Endoscopic Approach, Diagnostic |
| Aortic Valve Procedures | 02BF4ZZ | Excision of Aortic Valve, Percutaneous Endoscopic Approach |
| Aortic Valve Procedures | 02CF0ZZ | Extirpation of Matter from Aortic Valve, Open Approach |
| Aortic Valve Procedures | 02CF3ZZ | Extirpation of Matter from Aortic Valve, Percutaneous Approach |
| Aortic Valve Procedures | 02CF4ZZ | Extirpation of Matter from Aortic Valve, Percutaneous Endoscopic Approach |
| Aortic Valve Procedures | 02NF0ZZ | Release Aortic Valve, Open Approach |
| Aortic Valve Procedures | 02NF3ZZ | Release Aortic Valve, Percutaneous Approach |
| Aortic Valve Procedures | 02NF4ZZ | Release Aortic Valve, Percutaneous Endoscopic Approach |
| Aortic Valve Procedures | 02QF0ZJ | Repair Aortic Valve created from Truncal Valve, Open Approach |
| Aortic Valve Procedures | 02QF0ZZ | Repair Aortic Valve, Open Approach |
| Aortic Valve Procedures | 02QF3ZJ | Repair Aortic Valve created from Truncal Valve, Percutaneous Approach |
| Aortic Valve Procedures | 02QF3ZZ | Repair Aortic Valve, Percutaneous Approach |
| Aortic Valve Procedures | 02QF4ZJ | Repair Aortic Valve created from Truncal Valve, Percutaneous Endoscopic Approach |
| Aortic Valve Procedures | 02QF4ZZ | Repair Aortic Valve, Percutaneous Endoscopic Approach |
| Aortic Valve Procedures | 02RF07Z | Replacement of Aortic Valve with Autologous Tissue Substitute, Open Approach |
| Aortic Valve Procedures | 02RF08Z | Replacement of Aortic Valve with Zooplastic Tissue, Open Approach |
| Aortic Valve Procedures | 02RF0JZ | Replacement of Aortic Valve with Synthetic Substitute, Open Approach |
| Aortic Valve Procedures | 02RF0KZ | Replacement of Aortic Valve with Nonautologous Tissue Substitute, Open Approach |
| Aortic Valve Procedures | 02RF37H | Replacement of Aortic Valve with Autologous Tissue Substitute, Transapical, Percutaneous Approach |
| Aortic Valve Procedures | 02RF37Z | Replacement of Aortic Valve with Autologous Tissue Substitute, Percutaneous Approach |
| Aortic Valve Procedures | 02RF38H | Replacement of Aortic Valve with Zooplastic Tissue, Transapical, Percutaneous Approach |
| Aortic Valve Procedures | 02RF38Z | Replacement of Aortic Valve with Zooplastic Tissue, Percutaneous Approach |
| Aortic Valve Procedures | 02RF3JH | Replacement of Aortic Valve with Synthetic Substitute, Transapical, Percutaneous Approach |
| Aortic Valve Procedures | 02RF3JZ | Replacement of Aortic Valve with Synthetic Substitute, Percutaneous Approach |
| Aortic Valve Procedures | 02RF3KH | Replacement of Aortic Valve with Nonautologous Tissue Substitute, Transapical, Percutaneous Approach |
| Aortic Valve Procedures | 02RF3KZ | Replacement of Aortic Valve with Nonautologous Tissue Substitute, Percutaneous Approach |
| Aortic Valve Procedures | 02RF47Z | Replacement of Aortic Valve with Autologous Tissue Substitute, Percutaneous Endoscopic Approach |
| Aortic Valve Procedures | 02RF48Z | Replacement of Aortic Valve with Zooplastic Tissue, Percutaneous Endoscopic Approach |
| Aortic Valve Procedures | 02RF4JZ | Replacement of Aortic Valve with Synthetic Substitute, Percutaneous Endoscopic Approach |
| Aortic Valve Procedures | 02RF4KZ | Replacement of Aortic Valve with Nonautologous Tissue Substitute, Percutaneous Endoscopic Approach |
| Aortic Valve Procedures | 02UF07J | Supplement Aortic Valve created from Truncal Valve with Autologous Tissue Substitute, Open Approach |
| Aortic Valve Procedures | 02UF07Z | Supplement Aortic Valve with Autologous Tissue Substitute, Open Approach |
| Aortic Valve Procedures | 02UF08J | Supplement Aortic Valve created from Truncal Valve with Zooplastic Tissue, Open Approach |
| Aortic Valve Procedures | 02UF08Z | Supplement Aortic Valve with Zooplastic Tissue, Open Approach |
| Aortic Valve Procedures | 02UF0JJ | Supplement Aortic Valve created from Truncal Valve with Synthetic Substitute, Open Approach |
| Aortic Valve Procedures | 02UF0JZ | Supplement Aortic Valve with Synthetic Substitute, Open Approach |
| Aortic Valve Procedures | 02UF0KJ | Supplement Aortic Valve created from Truncal Valve with Nonautologous Tissue Substitute, Open Approach |
| Aortic Valve Procedures | 02UF0KZ | Supplement Aortic Valve with Nonautologous Tissue Substitute, Open Approach |
| Aortic Valve Procedures | 02UF37J | Supplement Aortic Valve created from Truncal Valve with Autologous Tissue Substitute, Percutaneous Approach |
| Aortic Valve Procedures | 02UF37Z | Supplement Aortic Valve with Autologous Tissue Substitute, Percutaneous Approach |
| Aortic Valve Procedures | 02UF38J | Supplement Aortic Valve created from Truncal Valve with Zooplastic Tissue, Percutaneous Approach |
| Aortic Valve Procedures | 02UF38Z | Supplement Aortic Valve with Zooplastic Tissue, Percutaneous Approach |
| Aortic Valve Procedures | 02UF3JJ | Supplement Aortic Valve created from Truncal Valve with Synthetic Substitute, Percutaneous Approach |
| Aortic Valve Procedures | 02UF3JZ | Supplement Aortic Valve with Synthetic Substitute, Percutaneous Approach |
| Aortic Valve Procedures | 02UF3KJ | Supplement Aortic Valve created from Truncal Valve with Nonautologous Tissue Substitute, Percutaneous Approach |
| Aortic Valve Procedures | 02UF3KZ | Supplement Aortic Valve with Nonautologous Tissue Substitute, Percutaneous Approach |
| Aortic Valve Procedures | 02UF47J | Supplement Aortic Valve created from Truncal Valve with Autologous Tissue Substitute, Percutaneous Endoscopic Approach |
| Aortic Valve Procedures | 02UF47Z | Supplement Aortic Valve with Autologous Tissue Substitute, Percutaneous Endoscopic Approach |
| Aortic Valve Procedures | 02UF48J | Supplement Aortic Valve created from Truncal Valve with Zooplastic Tissue, Percutaneous Endoscopic Approach |
| Aortic Valve Procedures | 02UF48Z | Supplement Aortic Valve with Zooplastic Tissue, Percutaneous Endoscopic Approach |
| Aortic Valve Procedures | 02UF4JJ | Supplement Aortic Valve created from Truncal Valve with Synthetic Substitute, Percutaneous Endoscopic Approach |
| Aortic Valve Procedures | 02UF4JZ | Supplement Aortic Valve with Synthetic Substitute, Percutaneous Endoscopic Approach |
| Aortic Valve Procedures | 02UF4KJ | Supplement Aortic Valve created from Truncal Valve with Nonautologous Tissue Substitute, Percutaneous Endoscopic Approach |
| Aortic Valve Procedures | 02UF4KZ | Supplement Aortic Valve with Nonautologous Tissue Substitute, Percutaneous Endoscopic Approach |
| Aortic Valve Procedures | 02WF07Z | Revision of Autologous Tissue Substitute in Aortic Valve, Open Approach |
| Aortic Valve Procedures | 02WF08Z | Revision of Zooplastic Tissue in Aortic Valve, Open Approach |
| Aortic Valve Procedures | 02WF0JZ | Revision of Synthetic Substitute in Aortic Valve, Open Approach |
| Aortic Valve Procedures | 02WF0KZ | Revision of Nonautologous Tissue Substitute in Aortic Valve, Open Approach |
| Aortic Valve Procedures | 02WF37Z | Revision of Autologous Tissue Substitute in Aortic Valve, Percutaneous Approach |
| Aortic Valve Procedures | 02WF38Z | Revision of Zooplastic Tissue in Aortic Valve, Percutaneous Approach |
| Aortic Valve Procedures | 02WF3JZ | Revision of Synthetic Substitute in Aortic Valve, Percutaneous Approach |
| Aortic Valve Procedures | 02WF3KZ | Revision of Nonautologous Tissue Substitute in Aortic Valve, Percutaneous Approach |
| Aortic Valve Procedures | 02WF47Z | Revision of Autologous Tissue Substitute in Aortic Valve, Percutaneous Endoscopic Approach |
| Aortic Valve Procedures | 02WF48Z | Revision of Zooplastic Tissue in Aortic Valve, Percutaneous Endoscopic Approach |
| Aortic Valve Procedures | 02WF4JZ | Revision of Synthetic Substitute in Aortic Valve, Percutaneous Endoscopic Approach |
| Aortic Valve Procedures | 02WF4KZ | Revision of Nonautologous Tissue Substitute in Aortic Valve, Percutaneous Endoscopic Approach |
| Tricuspid Valve Procedures | 027J04Z | Dilation of Tricuspid Valve with Drug-eluting Intraluminal Device, Open Approach |
| Tricuspid Valve Procedures | 027J0DZ | Dilation of Tricuspid Valve with Intraluminal Device, Open Approach |
| Tricuspid Valve Procedures | 027J0ZZ | Dilation of Tricuspid Valve, Open Approach |
| Tricuspid Valve Procedures | 027J34Z | Dilation of Tricuspid Valve with Drug-eluting Intraluminal Device, Percutaneous Approach |
| Tricuspid Valve Procedures | 027J3DZ | Dilation of Tricuspid Valve with Intraluminal Device, Percutaneous Approach |
| Tricuspid Valve Procedures | 027J3ZZ | Dilation of Tricuspid Valve, Percutaneous Approach |
| Tricuspid Valve Procedures | 027J44Z | Dilation of Tricuspid Valve with Drug-eluting Intraluminal Device, Percutaneous Endoscopic Approach |
| Tricuspid Valve Procedures | 027J4DZ | Dilation of Tricuspid Valve with Intraluminal Device, Percutaneous Endoscopic Approach |
| Tricuspid Valve Procedures | 027J4ZZ | Dilation of Tricuspid Valve, Percutaneous Endoscopic Approach |
| Tricuspid Valve Procedures | 02BJ0ZZ | Excision of Tricuspid Valve, Open Approach |
| Tricuspid Valve Procedures | 02BJ3ZZ | Excision of Tricuspid Valve, Percutaneous Approach |
| Tricuspid Valve Procedures | 02BJ4ZZ | Excision of Tricuspid Valve, Percutaneous Endoscopic Approach |
| Tricuspid Valve Procedures | 02CJ0ZZ | Extirpation of Matter from Tricuspid Valve, Open Approach |
| Tricuspid Valve Procedures | 02CJ3ZZ | Extirpation of Matter from Tricuspid Valve, Percutaneous Approach |
| Tricuspid Valve Procedures | 02CJ4ZZ | Extirpation of Matter from Tricuspid Valve, Percutaneous Endoscopic Approach |
| Tricuspid Valve Procedures | 02NJ0ZZ | Release Tricuspid Valve, Open Approach |
| Tricuspid Valve Procedures | 02NJ3ZZ | Release Tricuspid Valve, Percutaneous Approach |
| Tricuspid Valve Procedures | 02NJ4ZZ | Release Tricuspid Valve, Percutaneous Endoscopic Approach |
| Tricuspid Valve Procedures | 02QJ0ZG | Repair Tricuspid Valve created from Right Atrioventricular Valve, Open Approach |
| Tricuspid Valve Procedures | 02QJ0ZZ | Repair Tricuspid Valve, Open Approach |
| Tricuspid Valve Procedures | 02QJ3ZG | Repair Tricuspid Valve created from Right Atrioventricular Valve, Percutaneous Approach |
| Tricuspid Valve Procedures | 02QJ3ZZ | Repair Tricuspid Valve, Percutaneous Approach |
| Tricuspid Valve Procedures | 02QJ4ZG | Repair Tricuspid Valve created from Right Atrioventricular Valve, Percutaneous Endoscopic Approach |
| Tricuspid Valve Procedures | 02QJ4ZZ | Repair Tricuspid Valve, Percutaneous Endoscopic Approach |
| Tricuspid Valve Procedures | 02RJ07Z | Replacement of Tricuspid Valve with Autologous Tissue Substitute, Open Approach |
| Tricuspid Valve Procedures | 02RJ08Z | Replacement of Tricuspid Valve with Zooplastic Tissue, Open Approach |
| Tricuspid Valve Procedures | 02RJ0JZ | Replacement of Tricuspid Valve with Synthetic Substitute, Open Approach |
| Tricuspid Valve Procedures | 02RJ0KZ | Replacement of Tricuspid Valve with Nonautologous Tissue Substitute, Open Approach |
| Tricuspid Valve Procedures | 02RJ37H | Replacement of Tricuspid Valve with Autologous Tissue Substitute, Transapical, Percutaneous Approach |
| Tricuspid Valve Procedures | 02RJ37Z | Replacement of Tricuspid Valve with Autologous Tissue Substitute, Percutaneous Approach |
| Tricuspid Valve Procedures | 02RJ38H | Replacement of Tricuspid Valve with Zooplastic Tissue, Transapical, Percutaneous Approach |
| Tricuspid Valve Procedures | 02RJ38Z | Replacement of Tricuspid Valve with Zooplastic Tissue, Percutaneous Approach |
| Tricuspid Valve Procedures | 02RJ3JH | Replacement of Tricuspid Valve with Synthetic Substitute, Transapical, Percutaneous Approach |
| Tricuspid Valve Procedures | 02RJ3JZ | Replacement of Tricuspid Valve with Synthetic Substitute, Percutaneous Approach |
| Tricuspid Valve Procedures | 02RJ3KH | Replacement of Tricuspid Valve with Nonautologous Tissue Substitute, Transapical, Percutaneous Approach |
| Tricuspid Valve Procedures | 02RJ3KZ | Replacement of Tricuspid Valve with Nonautologous Tissue Substitute, Percutaneous Approach |
| Tricuspid Valve Procedures | 02RJ47Z | Replacement of Tricuspid Valve with Autologous Tissue Substitute, Percutaneous Endoscopic Approach |
| Tricuspid Valve Procedures | 02RJ48Z | Replacement of Tricuspid Valve with Zooplastic Tissue, Percutaneous Endoscopic Approach |
| Tricuspid Valve Procedures | 02RJ4JZ | Replacement of Tricuspid Valve with Synthetic Substitute, Percutaneous Endoscopic Approach |
| Tricuspid Valve Procedures | 02RJ4KZ | Replacement of Tricuspid Valve with Nonautologous Tissue Substitute, Percutaneous Endoscopic Approach |
| Tricuspid Valve Procedures | 02UJ07G | Supplement Tricuspid Valve created from Right Atrioventricular Valve with Autologous Tissue Substitute, Open Approach |
| Tricuspid Valve Procedures | 02UJ07Z | Supplement Tricuspid Valve with Autologous Tissue Substitute, Open Approach |
| Tricuspid Valve Procedures | 02UJ08G | Supplement Tricuspid Valve created from Right Atrioventricular Valve with Zooplastic Tissue, Open Approach |
| Tricuspid Valve Procedures | 02UJ08Z | Supplement Tricuspid Valve with Zooplastic Tissue, Open Approach |
| Tricuspid Valve Procedures | 02UJ0JG | Supplement Tricuspid Valve created from Right Atrioventricular Valve with Synthetic Substitute, Open Approach |
| Tricuspid Valve Procedures | 02UJ0JZ | Supplement Tricuspid Valve with Synthetic Substitute, Open Approach |
| Tricuspid Valve Procedures | 02UJ0KG | Supplement Tricuspid Valve created from Right Atrioventricular Valve with Nonautologous Tissue Substitute, Open Approach |
| Tricuspid Valve Procedures | 02UJ0KZ | Supplement Tricuspid Valve with Nonautologous Tissue Substitute, Open Approach |
| Tricuspid Valve Procedures | 02UJ37G | Supplement Tricuspid Valve created from Right Atrioventricular Valve with Autologous Tissue Substitute, Percutaneous Approach |
| Tricuspid Valve Procedures | 02UJ37Z | Supplement Tricuspid Valve with Autologous Tissue Substitute, Percutaneous Approach |
| Tricuspid Valve Procedures | 02UJ38G | Supplement Tricuspid Valve created from Right Atrioventricular Valve with Zooplastic Tissue, Percutaneous Approach |
| Tricuspid Valve Procedures | 02UJ38Z | Supplement Tricuspid Valve with Zooplastic Tissue, Percutaneous Approach |
| Tricuspid Valve Procedures | 02UJ3JG | Supplement Tricuspid Valve created from Right Atrioventricular Valve with Synthetic Substitute, Percutaneous Approach |
| Tricuspid Valve Procedures | 02UJ3JZ | Supplement Tricuspid Valve with Synthetic Substitute, Percutaneous Approach |
| Tricuspid Valve Procedures | 02UJ3KG | Supplement Tricuspid Valve created from Right Atrioventricular Valve with Nonautologous Tissue Substitute, Percutaneous Approach |
| Tricuspid Valve Procedures | 02UJ3KZ | Supplement Tricuspid Valve with Nonautologous Tissue Substitute, Percutaneous Approach |
| Tricuspid Valve Procedures | 02UJ47G | Supplement Tricuspid Valve created from Right Atrioventricular Valve with Autologous Tissue Substitute, Percutaneous Endoscopic Approach |
| Tricuspid Valve Procedures | 02UJ47Z | Supplement Tricuspid Valve with Autologous Tissue Substitute, Percutaneous Endoscopic Approach |
| Tricuspid Valve Procedures | 02UJ48G | Supplement Tricuspid Valve created from Right Atrioventricular Valve with Zooplastic Tissue, Percutaneous Endoscopic Approach |
| Tricuspid Valve Procedures | 02UJ48Z | Supplement Tricuspid Valve with Zooplastic Tissue, Percutaneous Endoscopic Approach |
| Tricuspid Valve Procedures | 02UJ4JG | Supplement Tricuspid Valve created from Right Atrioventricular Valve with Synthetic Substitute, Percutaneous Endoscopic Approach |
| Tricuspid Valve Procedures | 02UJ4JZ | Supplement Tricuspid Valve with Synthetic Substitute, Percutaneous Endoscopic Approach |
| Tricuspid Valve Procedures | 02UJ4KG | Supplement Tricuspid Valve created from Right Atrioventricular Valve with Nonautologous Tissue Substitute, Percutaneous Endoscopic Approach |
| Tricuspid Valve Procedures | 02UJ4KZ | Supplement Tricuspid Valve with Nonautologous Tissue Substitute, Percutaneous Endoscopic Approach |
| Pulmonic Valve Procedures | 02NH0ZZ | Release Pulmonary Valve, Open Approach |
| Pulmonic Valve Procedures | 02QH0ZZ | Repair Pulmonary Valve, Open Approach |
| Pulmonic Valve Procedures | 02RH07Z | Replacement of Pulmonary Valve with Autologous Tissue Substitute, Open Approach |
| Pulmonic Valve Procedures | 02RH08Z | Replacement of Pulmonary Valve with Zooplastic Tissue, Open Approach |
| Pulmonic Valve Procedures | 02RH0JZ | Replacement of Pulmonary Valve with Synthetic Substitute, Open Approach |
| Pulmonic Valve Procedures | 02RH0KZ | Replacement of Pulmonary Valve with Nonautologous Tissue Substitute, Open Approach |
| Pulmonic Valve Procedures | 02RH47Z | Replacement of Pulmonary Valve with Autologous Tissue Substitute, Percutaneous Endoscopic Approach |
| Pulmonic Valve Procedures | 02RH48Z | Replacement of Pulmonary Valve with Zooplastic Tissue, Percutaneous Endoscopic Approach |
| Pulmonic Valve Procedures | 02RH4JZ | Replacement of Pulmonary Valve with Synthetic Substitute, Percutaneous Endoscopic Approach |
| Pulmonic Valve Procedures | 02RH4KZ | Replacement of Pulmonary Valve with Nonautologous Tissue Substitute, Percutaneous Endoscopic Approach |

**Appendix C – Robotic ICD-10/HCPCS Codes**

| **Variable** | **Code** | **Description** |
| --- | --- | --- |
| Robot | S2900 | Surgical techniques requiring use of robotic surgical system (list separately in addition to code for primary procedure) |
| Robot | 8E090CZ | Robotic Assisted Procedure of Head and Neck Region, Open Approach |
| Robot | 8E093CZ | Robotic Assisted Procedure of Head and Neck Region, Percutaneous Approach |
| Robot | 8E094CZ | Robotic Assisted Procedure of Head and Neck Region, Percutaneous Endoscopic Approach |
| Robot | 8E097CZ | Robotic Assisted Procedure of Head and Neck Region, Via Natural or Artificial Opening |
| Robot | 8E098CZ | Robotic Assisted Procedure of Head and Neck Region, Via Natural or Artificial Opening Endoscopic |
| Robot | 8E09XCZ | Robotic Assisted Procedure of Head and Neck Region |
| Robot | 8E0W0CZ | Robotic Assisted Procedure of Trunk Region, Open Approach |
| Robot | 8E0W3CZ | Robotic Assisted Procedure of Trunk Region, Percutaneous Approach |
| Robot | 8E0W4CZ | Robotic Assisted Procedure of Trunk Region, Percutaneous Endoscopic Approach |
| Robot | 8E0W7CZ | Robotic Assisted Procedure of Trunk Region, Via Natural or Artificial Opening |
| Robot | 8E0W8CZ | Robotic Assisted Procedure of Trunk Region, Via Natural or Artificial Opening Endoscopic |
| Robot | 8E0WXCZ | Robotic Assisted Procedure of Trunk Region |
| Robot | 8E0X0CZ | Robotic Assisted Procedure of Upper Extremity, Open Approach |
| Robot | 8E0X3CZ | Robotic Assisted Procedure of Upper Extremity, Percutaneous Approach |
| Robot | 8E0X4CZ | Robotic Assisted Procedure of Upper Extremity, Percutaneous Endoscopic Approach |
| Robot | 8E0XXCZ | Robotic Assisted Procedure of Upper Extremity |
| Robot | 8E0Y0CZ | Robotic Assisted Procedure of Lower Extremity, Open Approach |
| Robot | 8E0Y3CZ | Robotic Assisted Procedure of Lower Extremity, Percutaneous Approach |
| Robot | 8E0Y4CZ | Robotic Assisted Procedure of Lower Extremity, Percutaneous Endoscopic Approach |
| Robot | 8E0YXCZ | Robotic Assisted Procedure of Lower Extremity |

**Appendix D – Elixhauser Comorbidity Index**

| **Variable** | **Code** | **Description** |
| --- | --- | --- |
| AIDS | B20 | Human immunodeficiency virus [HIV] disease |
| Alcohol Abuse | E52 | Niacin deficiency [pellagra] |
| Alcohol Abuse | F10.10 | Alcohol abuse, uncomplicated |
| Alcohol Abuse | F10.120 | Alcohol abuse with intoxication, uncomplicated |
| Alcohol Abuse | F10.121 | Alcohol abuse with intoxication delirium |
| Alcohol Abuse | F10.129 | Alcohol abuse with intoxication, unspecified |
| Alcohol Abuse | F10.14 | Alcohol abuse with alcohol-induced mood disorder |
| Alcohol Abuse | F10.150 | Alcohol abuse with alcohol-induced psychotic disorder with delusions |
| Alcohol Abuse | F10.151 | Alcohol abuse with alcohol-induced psychotic disorder with hallucinations |
| Alcohol Abuse | F10.159 | Alcohol abuse with alcohol-induced psychotic disorder, unspecified |
| Alcohol Abuse | F10.180 | Alcohol abuse with alcohol-induced anxiety disorder |
| Alcohol Abuse | F10.181 | Alcohol abuse with alcohol-induced sexual dysfunction |
| Alcohol Abuse | F10.182 | Alcohol abuse with alcohol-induced sleep disorder |
| Alcohol Abuse | F10.188 | Alcohol abuse with other alcohol-induced disorder |
| Alcohol Abuse | F10.19 | Alcohol abuse with unspecified alcohol-induced disorder |
| Alcohol Abuse | F10.20 | Alcohol dependence, uncomplicated |
| Alcohol Abuse | F10.21 | Alcohol dependence, in remission |
| Alcohol Abuse | F10.220 | Alcohol dependence with intoxication, uncomplicated |
| Alcohol Abuse | F10.221 | Alcohol dependence with intoxication delirium |
| Alcohol Abuse | F10.229 | Alcohol dependence with intoxication, unspecified |
| Alcohol Abuse | F10.230 | Alcohol dependence with withdrawal, uncomplicated |
| Alcohol Abuse | F10.231 | Alcohol dependence with withdrawal delirium |
| Alcohol Abuse | F10.232 | Alcohol dependence with withdrawal with perceptual disturbance |
| Alcohol Abuse | F10.239 | Alcohol dependence with withdrawal, unspecified |
| Alcohol Abuse | F10.24 | Alcohol dependence with alcohol-induced mood disorder |
| Alcohol Abuse | F10.250 | Alcohol dependence with alcohol-induced psychotic disorder with delusions |
| Alcohol Abuse | F10.251 | Alcohol dependence with alcohol-induced psychotic disorder with hallucinations |
| Alcohol Abuse | F10.259 | Alcohol dependence with alcohol-induced psychotic disorder, unspecified |
| Alcohol Abuse | F10.26 | Alcohol dependence with alcohol-induced persisting amnestic disorder |
| Alcohol Abuse | F10.27 | Alcohol dependence with alcohol-induced persisting dementia |
| Alcohol Abuse | F10.280 | Alcohol dependence with alcohol-induced anxiety disorder |
| Alcohol Abuse | F10.281 | Alcohol dependence with alcohol-induced sexual dysfunction |
| Alcohol Abuse | F10.282 | Alcohol dependence with alcohol-induced sleep disorder |
| Alcohol Abuse | F10.288 | Alcohol dependence with other alcohol-induced disorder |
| Alcohol Abuse | F10.29 | Alcohol dependence with unspecified alcohol-induced disorder |
| Alcohol Abuse | F10.920 | Alcohol use, unspecified with intoxication, uncomplicated |
| Alcohol Abuse | F10.921 | Alcohol use, unspecified with intoxication delirium |
| Alcohol Abuse | F10.929 | Alcohol use, unspecified with intoxication, unspecified |
| Alcohol Abuse | F10.94 | Alcohol use, unspecified with alcohol-induced mood disorder |
| Alcohol Abuse | F10.950 | Alcohol use, unspecified with alcohol-induced psychotic disorder with delusions |
| Alcohol Abuse | F10.951 | Alcohol use, unspecified with alcohol-induced psychotic disorder with hallucinations |
| Alcohol Abuse | F10.959 | Alcohol use, unspecified with alcohol-induced psychotic disorder, unspecified |
| Alcohol Abuse | F10.96 | Alcohol use, unspecified with alcohol-induced persisting amnestic disorder |
| Alcohol Abuse | F10.97 | Alcohol use, unspecified with alcohol-induced persisting dementia |
| Alcohol Abuse | F10.980 | Alcohol use, unspecified with alcohol-induced anxiety disorder |
| Alcohol Abuse | F10.981 | Alcohol use, unspecified with alcohol-induced sexual dysfunction |
| Alcohol Abuse | F10.982 | Alcohol use, unspecified with alcohol-induced sleep disorder |
| Alcohol Abuse | F10.988 | Alcohol use, unspecified with other alcohol-induced disorder |
| Alcohol Abuse | F10.99 | Alcohol use, unspecified with unspecified alcohol-induced disorder |
| Alcohol Abuse | G62.1 | Alcoholic polyneuropathy |
| Alcohol Abuse | I42.6 | Alcoholic cardiomyopathy |
| Alcohol Abuse | K29.20 | Alcoholic gastritis without bleeding |
| Alcohol Abuse | K29.21 | Alcoholic gastritis with bleeding |
| Alcohol Abuse | K70.0 | Alcoholic fatty liver |
| Alcohol Abuse | K70.30 | Alcoholic cirrhosis of liver without ascites |
| Alcohol Abuse | K70.31 | Alcoholic cirrhosis of liver with ascites |
| Alcohol Abuse | K70.9 | Alcoholic liver disease, unspecified |
| Alcohol Abuse | T51.0X1A | Toxic effect of ethanol, accidental (unintentional), initial encounter |
| Alcohol Abuse | T51.0X1D | Toxic effect of ethanol, accidental (unintentional), subsequent encounter |
| Alcohol Abuse | T51.0X1S | Toxic effect of ethanol, accidental (unintentional), sequela |
| Alcohol Abuse | T51.0X2A | Toxic effect of ethanol, intentional self-harm, initial encounter |
| Alcohol Abuse | T51.0X2D | Toxic effect of ethanol, intentional self-harm, subsequent encounter |
| Alcohol Abuse | T51.0X2S | Toxic effect of ethanol, intentional self-harm, sequela |
| Alcohol Abuse | T51.0X3A | Toxic effect of ethanol, assault, initial encounter |
| Alcohol Abuse | T51.0X3D | Toxic effect of ethanol, assault, subsequent encounter |
| Alcohol Abuse | T51.0X3S | Toxic effect of ethanol, assault, sequela |
| Alcohol Abuse | T51.0X4A | Toxic effect of ethanol, undetermined, initial encounter |
| Alcohol Abuse | T51.0X4D | Toxic effect of ethanol, undetermined, subsequent encounter |
| Alcohol Abuse | T51.0X4S | Toxic effect of ethanol, undetermined, sequela |
| Alcohol Abuse | T51.1X1A | Toxic effect of methanol, accidental (unintentional), initial encounter |
| Alcohol Abuse | T51.1X1D | Toxic effect of methanol, accidental (unintentional), subsequent encounter |
| Alcohol Abuse | T51.1X1S | Toxic effect of methanol, accidental (unintentional), sequela |
| Alcohol Abuse | T51.1X2A | Toxic effect of methanol, intentional self-harm, initial encounter |
| Alcohol Abuse | T51.1X2D | Toxic effect of methanol, intentional self-harm, subsequent encounter |
| Alcohol Abuse | T51.1X2S | Toxic effect of methanol, intentional self-harm, sequela |
| Alcohol Abuse | T51.1X3A | Toxic effect of methanol, assault, initial encounter |
| Alcohol Abuse | T51.1X3D | Toxic effect of methanol, assault, subsequent encounter |
| Alcohol Abuse | T51.1X3S | Toxic effect of methanol, assault, sequela |
| Alcohol Abuse | T51.1X4A | Toxic effect of methanol, undetermined, initial encounter |
| Alcohol Abuse | T51.1X4D | Toxic effect of methanol, undetermined, subsequent encounter |
| Alcohol Abuse | T51.1X4S | Toxic effect of methanol, undetermined, sequela |
| Alcohol Abuse | T51.2X1A | Toxic effect of 2-Propanol, accidental (unintentional), initial encounter |
| Alcohol Abuse | T51.2X1D | Toxic effect of 2-Propanol, accidental (unintentional), subsequent encounter |
| Alcohol Abuse | T51.2X1S | Toxic effect of 2-Propanol, accidental (unintentional), sequela |
| Alcohol Abuse | T51.2X2A | Toxic effect of 2-Propanol, intentional self-harm, initial encounter |
| Alcohol Abuse | T51.2X2D | Toxic effect of 2-Propanol, intentional self-harm, subsequent encounter |
| Alcohol Abuse | T51.2X2S | Toxic effect of 2-Propanol, intentional self-harm, sequela |
| Alcohol Abuse | T51.2X3A | Toxic effect of 2-Propanol, assault, initial encounter |
| Alcohol Abuse | T51.2X3D | Toxic effect of 2-Propanol, assault, subsequent encounter |
| Alcohol Abuse | T51.2X3S | Toxic effect of 2-Propanol, assault, sequela |
| Alcohol Abuse | T51.2X4A | Toxic effect of 2-Propanol, undetermined, initial encounter |
| Alcohol Abuse | T51.2X4D | Toxic effect of 2-Propanol, undetermined, subsequent encounter |
| Alcohol Abuse | T51.2X4S | Toxic effect of 2-Propanol, undetermined, sequela |
| Alcohol Abuse | T51.3X1A | Toxic effect of fusel oil, accidental (unintentional), initial encounter |
| Alcohol Abuse | T51.3X1D | Toxic effect of fusel oil, accidental (unintentional), subsequent encounter |
| Alcohol Abuse | T51.3X1S | Toxic effect of fusel oil, accidental (unintentional), sequela |
| Alcohol Abuse | T51.3X2A | Toxic effect of fusel oil, intentional self-harm, initial encounter |
| Alcohol Abuse | T51.3X2D | Toxic effect of fusel oil, intentional self-harm, subsequent encounter |
| Alcohol Abuse | T51.3X2S | Toxic effect of fusel oil, intentional self-harm, sequela |
| Alcohol Abuse | T51.3X3A | Toxic effect of fusel oil, assault, initial encounter |
| Alcohol Abuse | T51.3X3D | Toxic effect of fusel oil, assault, subsequent encounter |
| Alcohol Abuse | T51.3X3S | Toxic effect of fusel oil, assault, sequela |
| Alcohol Abuse | T51.3X4A | Toxic effect of fusel oil, undetermined, initial encounter |
| Alcohol Abuse | T51.3X4D | Toxic effect of fusel oil, undetermined, subsequent encounter |
| Alcohol Abuse | T51.3X4S | Toxic effect of fusel oil, undetermined, sequela |
| Alcohol Abuse | T51.8X1A | Toxic effect of other alcohols, accidental (unintentional), initial encounter |
| Alcohol Abuse | T51.8X1D | Toxic effect of other alcohols, accidental (unintentional), subsequent encounter |
| Alcohol Abuse | T51.8X1S | Toxic effect of other alcohols, accidental (unintentional), sequela |
| Alcohol Abuse | T51.8X2A | Toxic effect of other alcohols, intentional self-harm, initial encounter |
| Alcohol Abuse | T51.8X2D | Toxic effect of other alcohols, intentional self-harm, subsequent encounter |
| Alcohol Abuse | T51.8X2S | Toxic effect of other alcohols, intentional self-harm, sequela |
| Alcohol Abuse | T51.8X3A | Toxic effect of other alcohols, assault, initial encounter |
| Alcohol Abuse | T51.8X3D | Toxic effect of other alcohols, assault, subsequent encounter |
| Alcohol Abuse | T51.8X3S | Toxic effect of other alcohols, assault, sequela |
| Alcohol Abuse | T51.8X4A | Toxic effect of other alcohols, undetermined, initial encounter |
| Alcohol Abuse | T51.8X4D | Toxic effect of other alcohols, undetermined, subsequent encounter |
| Alcohol Abuse | T51.8X4S | Toxic effect of other alcohols, undetermined, sequela |
| Alcohol Abuse | T51.91XA | Toxic effect of unspecified alcohol, accidental (unintentional), initial encounter |
| Alcohol Abuse | T51.91XD | Toxic effect of unspecified alcohol, accidental (unintentional), subsequent encounter |
| Alcohol Abuse | T51.91XS | Toxic effect of unspecified alcohol, accidental (unintentional), sequela |
| Alcohol Abuse | T51.92XA | Toxic effect of unspecified alcohol, intentional self-harm, initial encounter |
| Alcohol Abuse | T51.92XD | Toxic effect of unspecified alcohol, intentional self-harm, subsequent encounter |
| Alcohol Abuse | T51.92XS | Toxic effect of unspecified alcohol, intentional self-harm, sequela |
| Alcohol Abuse | T51.93XA | Toxic effect of unspecified alcohol, assault, initial encounter |
| Alcohol Abuse | T51.93XD | Toxic effect of unspecified alcohol, assault, subsequent encounter |
| Alcohol Abuse | T51.93XS | Toxic effect of unspecified alcohol, assault, sequela |
| Alcohol Abuse | T51.94XA | Toxic effect of unspecified alcohol, undetermined, initial encounter |
| Alcohol Abuse | T51.94XD | Toxic effect of unspecified alcohol, undetermined, subsequent encounter |
| Alcohol Abuse | T51.94XS | Toxic effect of unspecified alcohol, undetermined, sequela |
| Alcohol Abuse | Z71.41 | Alcohol abuse counseling and surveillance of alcoholic |
| Alcohol Abuse | Z71.42 | Counseling for family member of alcoholic |
| Arrhythmias | I44.1 | Atrioventricular block, second degree |
| Arrhythmias | I44.2 | Atrioventricular block, complete |
| Arrhythmias | I44.30 | Unspecified atrioventricular block |
| Arrhythmias | I44.39 | Other atrioventricular block |
| Arrhythmias | I45.6 | Pre-excitation syndrome |
| Arrhythmias | I45.9 | Conduction disorder, unspecified |
| Arrhythmias | I47.0 | Re-entry ventricular arrhythmia |
| Arrhythmias | I47.1 | Supraventricular tachycardia |
| Arrhythmias | I47.2 | Ventricular tachycardia |
| Arrhythmias | I47.9 | Paroxysmal tachycardia, unspecified |
| Arrhythmias | I48.0 | Paroxysmal atrial fibrillation |
| Arrhythmias | I48.1 | Persistent atrial fibrillation |
| Arrhythmias | I48.2 | Chronic atrial fibrillation |
| Arrhythmias | I48.3 | Typical atrial flutter |
| Arrhythmias | I48.4 | Atypical atrial flutter |
| Arrhythmias | I48.91 | Unspecified atrial fibrillation |
| Arrhythmias | I48.92 | Unspecified atrial flutter |
| Arrhythmias | I49.01 | Ventricular fibrillation |
| Arrhythmias | I49.02 | Ventricular flutter |
| Arrhythmias | I49.1 | Atrial premature depolarization |
| Arrhythmias | I49.2 | Junctional premature depolarization |
| Arrhythmias | I49.3 | Ventricular premature depolarization |
| Arrhythmias | I49.40 | Unspecified premature depolarization |
| Arrhythmias | I49.49 | Other premature depolarization |
| Arrhythmias | I49.5 | Sick sinus syndrome |
| Arrhythmias | I49.8 | Other specified cardiac arrhythmias |
| Arrhythmias | I49.9 | Cardiac arrhythmia, unspecified |
| Arrhythmias | R00.0 | Tachycardia, unspecified |
| Arrhythmias | R00.1 | Bradycardia, unspecified |
| Arrhythmias | R00.8 | Other abnormalities of heart beat |
| Arrhythmias | T82.110A | Breakdown (mechanical) of cardiac electrode, initial encounter |
| Arrhythmias | T82.110D | Breakdown (mechanical) of cardiac electrode, subsequent encounter |
| Arrhythmias | T82.110S | Breakdown (mechanical) of cardiac electrode, sequela |
| Arrhythmias | T82.111A | Breakdown (mechanical) of cardiac pulse generator (battery), initial encounter |
| Arrhythmias | T82.111D | Breakdown (mechanical) of cardiac pulse generator (battery), subsequent encounter |
| Arrhythmias | T82.111S | Breakdown (mechanical) of cardiac pulse generator (battery), sequela |
| Arrhythmias | T82.118A | Breakdown (mechanical) of other cardiac electronic device, initial encounter |
| Arrhythmias | T82.118D | Breakdown (mechanical) of other cardiac electronic device, subsequent encounter |
| Arrhythmias | T82.118S | Breakdown (mechanical) of other cardiac electronic device, sequela |
| Arrhythmias | T82.119A | Breakdown (mechanical) of unspecified cardiac electronic device, initial encounter |
| Arrhythmias | T82.119D | Breakdown (mechanical) of unspecified cardiac electronic device, subsequent encounter |
| Arrhythmias | T82.119S | Breakdown (mechanical) of unspecified cardiac electronic device, sequela |
| Arrhythmias | T82.120A | Displacement of cardiac electrode, initial encounter |
| Arrhythmias | T82.120D | Displacement of cardiac electrode, subsequent encounter |
| Arrhythmias | T82.120S | Displacement of cardiac electrode, sequela |
| Arrhythmias | T82.121A | Displacement of cardiac pulse generator (battery), initial encounter |
| Arrhythmias | T82.121D | Displacement of cardiac pulse generator (battery), subsequent encounter |
| Arrhythmias | T82.121S | Displacement of cardiac pulse generator (battery), sequela |
| Arrhythmias | T82.128A | Displacement of other cardiac electronic device, initial encounter |
| Arrhythmias | T82.128D | Displacement of other cardiac electronic device, subsequent encounter |
| Arrhythmias | T82.128S | Displacement of other cardiac electronic device, sequela |
| Arrhythmias | T82.129A | Displacement of unspecified cardiac electronic device, initial encounter |
| Arrhythmias | T82.129D | Displacement of unspecified cardiac electronic device, subsequent encounter |
| Arrhythmias | T82.129S | Displacement of unspecified cardiac electronic device, sequela |
| Arrhythmias | T82.190A | Other mechanical complication of cardiac electrode, initial encounter |
| Arrhythmias | T82.190D | Other mechanical complication of cardiac electrode, subsequent encounter |
| Arrhythmias | T82.190S | Other mechanical complication of cardiac electrode, sequela |
| Arrhythmias | T82.191A | Other mechanical complication of cardiac pulse generator (battery), initial encounter |
| Arrhythmias | T82.191D | Other mechanical complication of cardiac pulse generator (battery), subsequent encounter |
| Arrhythmias | T82.191S | Other mechanical complication of cardiac pulse generator (battery), sequela |
| Arrhythmias | T82.198A | Other mechanical complication of other cardiac electronic device, initial encounter |
| Arrhythmias | T82.198D | Other mechanical complication of other cardiac electronic device, subsequent encounter |
| Arrhythmias | T82.198S | Other mechanical complication of other cardiac electronic device, sequela |
| Arrhythmias | T82.199A | Other mechanical complication of unspecified cardiac device, initial encounter |
| Arrhythmias | T82.199D | Other mechanical complication of unspecified cardiac device, subsequent encounter |
| Arrhythmias | T82.199S | Other mechanical complication of unspecified cardiac device, sequela |
| Arrhythmias | Z45.010 | Encounter for checking and testing of cardiac pacemaker pulse generator [battery] |
| Arrhythmias | Z45.018 | Encounter for adjustment and management of other part of cardiac pacemaker |
| Arrhythmias | Z45.02 | Encounter for adjustment and management of automatic implantable cardiac defibrillator |
| Arrhythmias | Z45.09 | Encounter for adjustment and management of other cardiac device |
| Arrhythmias | Z49.01 | Encounter for fitting and adjustment of extracorporeal dialysis catheter |
| Arrhythmias | Z49.02 | Encounter for fitting and adjustment of peritoneal dialysis catheter |
| Arrhythmias | Z95.0 | Presence of cardiac pacemaker |
| Chronic Pulmonary Disease | I27.81 | Cor pulmonale (chronic) |
| Chronic Pulmonary Disease | I27.82 | Chronic pulmonary embolism |
| Chronic Pulmonary Disease | I27.89 | Other specified pulmonary heart diseases |
| Chronic Pulmonary Disease | I27.9 | Pulmonary heart disease, unspecified |
| Chronic Pulmonary Disease | J40 | Bronchitis, not specified as acute or chronic |
| Chronic Pulmonary Disease | J41.0 | Simple chronic bronchitis |
| Chronic Pulmonary Disease | J41.1 | Mucopurulent chronic bronchitis |
| Chronic Pulmonary Disease | J41.8 | Mixed simple and mucopurulent chronic bronchitis |
| Chronic Pulmonary Disease | J42 | Unspecified chronic bronchitis |
| Chronic Pulmonary Disease | J43.0 | Unilateral pulmonary emphysema [MacLeod's syndrome] |
| Chronic Pulmonary Disease | J43.1 | Panlobular emphysema |
| Chronic Pulmonary Disease | J43.2 | Centrilobular emphysema |
| Chronic Pulmonary Disease | J43.8 | Other emphysema |
| Chronic Pulmonary Disease | J43.9 | Emphysema, unspecified |
| Chronic Pulmonary Disease | J44.0 | Chronic obstructive pulmonary disease with (acute) lower respiratory infection |
| Chronic Pulmonary Disease | J44.1 | Chronic obstructive pulmonary disease with (acute) exacerbation |
| Chronic Pulmonary Disease | J44.9 | Chronic obstructive pulmonary disease, unspecified |
| Chronic Pulmonary Disease | J45.20 | Mild intermittent asthma, uncomplicated |
| Chronic Pulmonary Disease | J45.21 | Mild intermittent asthma with (acute) exacerbation |
| Chronic Pulmonary Disease | J45.22 | Mild intermittent asthma with status asthmaticus |
| Chronic Pulmonary Disease | J45.30 | Mild persistent asthma, uncomplicated |
| Chronic Pulmonary Disease | J45.31 | Mild persistent asthma with (acute) exacerbation |
| Chronic Pulmonary Disease | J45.32 | Mild persistent asthma with status asthmaticus |
| Chronic Pulmonary Disease | J45.40 | Moderate persistent asthma, uncomplicated |
| Chronic Pulmonary Disease | J45.41 | Moderate persistent asthma with (acute) exacerbation |
| Chronic Pulmonary Disease | J45.42 | Moderate persistent asthma with status asthmaticus |
| Chronic Pulmonary Disease | J45.50 | Severe persistent asthma, uncomplicated |
| Chronic Pulmonary Disease | J45.51 | Severe persistent asthma with (acute) exacerbation |
| Chronic Pulmonary Disease | J45.52 | Severe persistent asthma with status asthmaticus |
| Chronic Pulmonary Disease | J45.901 | Unspecified asthma with (acute) exacerbation |
| Chronic Pulmonary Disease | J45.902 | Unspecified asthma with status asthmaticus |
| Chronic Pulmonary Disease | J45.909 | Unspecified asthma, uncomplicated |
| Chronic Pulmonary Disease | J45.990 | Exercise induced bronchospasm |
| Chronic Pulmonary Disease | J45.991 | Cough variant asthma |
| Chronic Pulmonary Disease | J45.998 | Other asthma |
| Chronic Pulmonary Disease | J47.0 | Bronchiectasis with acute lower respiratory infection |
| Chronic Pulmonary Disease | J47.1 | Bronchiectasis with (acute) exacerbation |
| Chronic Pulmonary Disease | J47.9 | Bronchiectasis, uncomplicated |
| Chronic Pulmonary Disease | J60 | Coalworker's pneumoconiosis |
| Chronic Pulmonary Disease | J61 | Pneumoconiosis due to asbestos and other mineral fibers |
| Chronic Pulmonary Disease | J62.0 | Pneumoconiosis due to talc dust |
| Chronic Pulmonary Disease | J62.8 | Pneumoconiosis due to other dust containing silica |
| Chronic Pulmonary Disease | J63.0 | Aluminosis (of lung) |
| Chronic Pulmonary Disease | J63.1 | Bauxite fibrosis (of lung) |
| Chronic Pulmonary Disease | J63.2 | Berylliosis |
| Chronic Pulmonary Disease | J63.3 | Graphite fibrosis (of lung) |
| Chronic Pulmonary Disease | J63.4 | Siderosis |
| Chronic Pulmonary Disease | J63.5 | Stannosis |
| Chronic Pulmonary Disease | J63.6 | Pneumoconiosis due to other specified inorganic dusts |
| Chronic Pulmonary Disease | J64 | Unspecified pneumoconiosis |
| Chronic Pulmonary Disease | J65 | Pneumoconiosis associated with tuberculosis |
| Chronic Pulmonary Disease | J66.0 | Byssinosis |
| Chronic Pulmonary Disease | J66.1 | Flax-dressers' disease |
| Chronic Pulmonary Disease | J66.2 | Cannabinosis |
| Chronic Pulmonary Disease | J66.8 | Airway disease due to other specific organic dusts |
| Chronic Pulmonary Disease | J67.0 | Farmer's lung |
| Chronic Pulmonary Disease | J67.1 | Bagassosis |
| Chronic Pulmonary Disease | J67.2 | Bird fancier's lung |
| Chronic Pulmonary Disease | J67.3 | Suberosis |
| Chronic Pulmonary Disease | J67.4 | Maltworker's lung |
| Chronic Pulmonary Disease | J67.5 | Mushroom-worker's lung |
| Chronic Pulmonary Disease | J67.6 | Maple-bark-stripper's lung |
| Chronic Pulmonary Disease | J67.7 | Air conditioner and humidifier lung |
| Chronic Pulmonary Disease | J67.8 | Hypersensitivity pneumonitis due to other organic dusts |
| Chronic Pulmonary Disease | J67.9 | Hypersensitivity pneumonitis due to unspecified organic dust |
| Chronic Pulmonary Disease | J68.4 | Chronic respiratory conditions due to chemicals, gases, fumes and vapors |
| Chronic Pulmonary Disease | J70.1 | Chronic and other pulmonary manifestations due to radiation |
| Chronic Pulmonary Disease | J70.3 | Chronic drug-induced interstitial lung disorders |
| Coagulopathy | D65 | Disseminated intravascular coagulation [defibrination syndrome] |
| Coagulopathy | D66 | Hereditary factor VIII deficiency |
| Coagulopathy | D67 | Hereditary factor IX deficiency |
| Coagulopathy | D68.0 | Von Willebrand's disease |
| Coagulopathy | D68.1 | Hereditary factor XI deficiency |
| Coagulopathy | D68.2 | Hereditary deficiency of other clotting factors |
| Coagulopathy | D68.311 | Acquired hemophilia |
| Coagulopathy | D68.312 | Antiphospholipid antibody with hemorrhagic disorder |
| Coagulopathy | D68.318 | Other hemorrhagic disorder due to intrinsic circulating anticoagulants, antibodies, or inhibitors |
| Coagulopathy | D68.32 | Hemorrhagic disorder due to extrinsic circulating anticoagulants |
| Coagulopathy | D68.4 | Acquired coagulation factor deficiency |
| Coagulopathy | D68.51 | Activated protein C resistance |
| Coagulopathy | D68.52 | Prothrombin gene mutation |
| Coagulopathy | D68.59 | Other primary thrombophilia |
| Coagulopathy | D68.61 | Antiphospholipid syndrome |
| Coagulopathy | D68.62 | Lupus anticoagulant syndrome |
| Coagulopathy | D68.69 | Other thrombophilia |
| Coagulopathy | D68.8 | Other specified coagulation defects |
| Coagulopathy | D68.9 | Coagulation defect, unspecified |
| Coagulopathy | D69.1 | Qualitative platelet defects |
| Coagulopathy | D69.3 | Immune thrombocytopenic purpura |
| Coagulopathy | D69.41 | Evans syndrome |
| Coagulopathy | D69.42 | Congenital and hereditary thrombocytopenia purpura |
| Coagulopathy | D69.49 | Other primary thrombocytopenia |
| Coagulopathy | D69.51 | Posttransfusion purpura |
| Coagulopathy | D69.59 | Other secondary thrombocytopenia |
| Coagulopathy | D69.6 | Thrombocytopenia, unspecified |
| Congestive Heart Failure | I09.1 | Rheumatic diseases of endocardium, valve unspecified |
| Congestive Heart Failure | I09.9 | Rheumatic heart disease, unspecified |
| Congestive Heart Failure | I11.0 | Hypertensive heart disease with heart failure |
| Congestive Heart Failure | I13.0 | Hypertensive heart and chronic kidney disease with heart failure and stage 1 through stage 4 chronic kidney disease, or unspecified chronic kidney disease |
| Congestive Heart Failure | I13.2 | Hypertensive heart and chronic kidney disease with heart failure and with stage 5 chronic kidney disease, or end stage renal disease |
| Congestive Heart Failure | I25.5 | Ischemic cardiomyopathy |
| Congestive Heart Failure | I42.0 | Dilated cardiomyopathy |
| Congestive Heart Failure | I42.5 | Other restrictive cardiomyopathy |
| Congestive Heart Failure | I42.6 | Alcoholic cardiomyopathy |
| Congestive Heart Failure | I42.7 | Cardiomyopathy due to drug and external agent |
| Congestive Heart Failure | I42.8 | Other cardiomyopathies |
| Congestive Heart Failure | I42.9 | Cardiomyopathy, unspecified |
| Congestive Heart Failure | I43 | Cardiomyopathy in diseases classified elsewhere |
| Congestive Heart Failure | I50.1 | Left ventricular failure, unspecified |
| Congestive Heart Failure | I50.20 | Unspecified systolic (congestive) heart failure |
| Congestive Heart Failure | I50.21 | Acute systolic (congestive) heart failure |
| Congestive Heart Failure | I50.22 | Chronic systolic (congestive) heart failure |
| Congestive Heart Failure | I50.23 | Acute on chronic systolic (congestive) heart failure |
| Congestive Heart Failure | I50.30 | Unspecified diastolic (congestive) heart failure |
| Congestive Heart Failure | I50.31 | Acute diastolic (congestive) heart failure |
| Congestive Heart Failure | I50.32 | Chronic diastolic (congestive) heart failure |
| Congestive Heart Failure | I50.33 | Acute on chronic diastolic (congestive) heart failure |
| Congestive Heart Failure | I50.40 | Unspecified combined systolic (congestive) and diastolic (congestive) heart failure |
| Congestive Heart Failure | I50.41 | Acute combined systolic (congestive) and diastolic (congestive) heart failure |
| Congestive Heart Failure | I50.42 | Chronic combined systolic (congestive) and diastolic (congestive) heart failure |
| Congestive Heart Failure | I50.43 | Acute on chronic combined systolic (congestive) and diastolic (congestive) heart failure |
| Congestive Heart Failure | I50.9 | Heart failure, unspecified |
| Congestive Heart Failure | P29.0 | Neonatal cardiac failure |
| Deficiency Anemias | D50.8 | Other iron deficiency anemias |
| Deficiency Anemias | D50.9 | Iron deficiency anemia, unspecified |
| Deficiency Anemias | D51.0 | Vitamin B12 deficiency anemia due to intrinsic factor deficiency |
| Deficiency Anemias | D51.1 | Vitamin B12 deficiency anemia due to selective vitamin B12 malabsorption with proteinuria |
| Deficiency Anemias | D51.2 | Transcobalamin II deficiency |
| Deficiency Anemias | D51.3 | Other dietary vitamin B12 deficiency anemia |
| Deficiency Anemias | D51.8 | Other vitamin B12 deficiency anemias |
| Deficiency Anemias | D51.9 | Vitamin B12 deficiency anemia, unspecified |
| Deficiency Anemias | D52.0 | Dietary folate deficiency anemia |
| Deficiency Anemias | D52.1 | Drug-induced folate deficiency anemia |
| Deficiency Anemias | D52.8 | Other folate deficiency anemias |
| Deficiency Anemias | D52.9 | Folate deficiency anemia, unspecified |
| Deficiency Anemias | D53.0 | Protein deficiency anemia |
| Deficiency Anemias | D53.1 | Other megaloblastic anemias, not elsewhere classified |
| Deficiency Anemias | D53.2 | Scorbutic anemia |
| Deficiency Anemias | D53.8 | Other specified nutritional anemias |
| Deficiency Anemias | D53.9 | Nutritional anemia, unspecified |
| Depression | F31.30 | Bipolar disorder, current episode depressed, mild or moderate severity, unspecified |
| Depression | F31.31 | Bipolar disorder, current episode depressed, mild |
| Depression | F31.32 | Bipolar disorder, current episode depressed, moderate |
| Depression | F31.4 | Bipolar disorder, current episode depressed, severe, without psychotic features |
| Depression | F31.5 | Bipolar disorder, current episode depressed, severe, with psychotic features |
| Depression | F32.0 | Major depressive disorder, single episode, mild |
| Depression | F32.1 | Major depressive disorder, single episode, moderate |
| Depression | F32.2 | Major depressive disorder, single episode, severe without psychotic features |
| Depression | F32.3 | Major depressive disorder, single episode, severe with psychotic features |
| Depression | F32.4 | Major depressive disorder, single episode, in partial remission |
| Depression | F32.5 | Major depressive disorder, single episode, in full remission |
| Depression | F32.8 | Other depressive episodes |
| Depression | F32.9 | Major depressive disorder, single episode, unspecified |
| Depression | F33.0 | Major depressive disorder, recurrent, mild |
| Depression | F33.1 | Major depressive disorder, recurrent, moderate |
| Depression | F33.2 | Major depressive disorder, recurrent severe without psychotic features |
| Depression | F33.3 | Major depressive disorder, recurrent, severe with psychotic symptoms |
| Depression | F33.40 | Major depressive disorder, recurrent, in remission, unspecified |
| Depression | F33.41 | Major depressive disorder, recurrent, in partial remission |
| Depression | F33.42 | Major depressive disorder, recurrent, in full remission |
| Depression | F33.8 | Other recurrent depressive disorders |
| Depression | F33.9 | Major depressive disorder, recurrent, unspecified |
| Depression | F34.1 | Dysthymic disorder |
| Depression | F43.20 | Adjustment disorder, unspecified |
| Depression | F43.21 | Adjustment disorder with depressed mood |
| Depression | F43.22 | Adjustment disorder with anxiety |
| Depression | F43.23 | Adjustment disorder with mixed anxiety and depressed mood |
| Depression | F43.24 | Adjustment disorder with disturbance of conduct |
| Depression | F43.25 | Adjustment disorder with mixed disturbance of emotions and conduct |
| Depression | F43.29 | Adjustment disorder with other symptoms |
| Diabetes Complicated | E10.21 | Type 1 diabetes mellitus with diabetic nephropathy |
| Diabetes Complicated | E10.22 | Type 1 diabetes mellitus with diabetic chronic kidney disease |
| Diabetes Complicated | E10.29 | Type 1 diabetes mellitus with other diabetic kidney complication |
| Diabetes Complicated | E10.311 | Type 1 diabetes mellitus with unspecified diabetic retinopathy with macular edema |
| Diabetes Complicated | E10.319 | Type 1 diabetes mellitus with unspecified diabetic retinopathy without macular edema |
| Diabetes Complicated | E10.321 | Type 1 diabetes mellitus with mild nonproliferative diabetic retinopathy with macular edema |
| Diabetes Complicated | E10.329 | Type 1 diabetes mellitus with mild nonproliferative diabetic retinopathy without macular edema |
| Diabetes Complicated | E10.331 | Type 1 diabetes mellitus with moderate nonproliferative diabetic retinopathy with macular edema |
| Diabetes Complicated | E10.339 | Type 1 diabetes mellitus with moderate nonproliferative diabetic retinopathy without macular edema |
| Diabetes Complicated | E10.341 | Type 1 diabetes mellitus with severe nonproliferative diabetic retinopathy with macular edema |
| Diabetes Complicated | E10.349 | Type 1 diabetes mellitus with severe nonproliferative diabetic retinopathy without macular edema |
| Diabetes Complicated | E10.351 | Type 1 diabetes mellitus with proliferative diabetic retinopathy with macular edema |
| Diabetes Complicated | E10.359 | Type 1 diabetes mellitus with proliferative diabetic retinopathy without macular edema |
| Diabetes Complicated | E10.36 | Type 1 diabetes mellitus with diabetic cataract |
| Diabetes Complicated | E10.39 | Type 1 diabetes mellitus with other diabetic ophthalmic complication |
| Diabetes Complicated | E10.40 | Type 1 diabetes mellitus with diabetic neuropathy, unspecified |
| Diabetes Complicated | E10.41 | Type 1 diabetes mellitus with diabetic mononeuropathy |
| Diabetes Complicated | E10.42 | Type 1 diabetes mellitus with diabetic polyneuropathy |
| Diabetes Complicated | E10.43 | Type 1 diabetes mellitus with diabetic autonomic (poly)neuropathy |
| Diabetes Complicated | E10.44 | Type 1 diabetes mellitus with diabetic amyotrophy |
| Diabetes Complicated | E10.49 | Type 1 diabetes mellitus with other diabetic neurological complication |
| Diabetes Complicated | E10.51 | Type 1 diabetes mellitus with diabetic peripheral angiopathy without gangrene |
| Diabetes Complicated | E10.52 | Type 1 diabetes mellitus with diabetic peripheral angiopathy with gangrene |
| Diabetes Complicated | E10.59 | Type 1 diabetes mellitus with other circulatory complications |
| Diabetes Complicated | E10.610 | Type 1 diabetes mellitus with diabetic neuropathic arthropathy |
| Diabetes Complicated | E10.618 | Type 1 diabetes mellitus with other diabetic arthropathy |
| Diabetes Complicated | E10.620 | Type 1 diabetes mellitus with diabetic dermatitis |
| Diabetes Complicated | E10.621 | Type 1 diabetes mellitus with foot ulcer |
| Diabetes Complicated | E10.622 | Type 1 diabetes mellitus with other skin ulcer |
| Diabetes Complicated | E10.628 | Type 1 diabetes mellitus with other skin complications |
| Diabetes Complicated | E10.630 | Type 1 diabetes mellitus with periodontal disease |
| Diabetes Complicated | E10.638 | Type 1 diabetes mellitus with other oral complications |
| Diabetes Complicated | E10.641 | Type 1 diabetes mellitus with hypoglycemia with coma |
| Diabetes Complicated | E10.649 | Type 1 diabetes mellitus with hypoglycemia without coma |
| Diabetes Complicated | E10.65 | Type 1 diabetes mellitus with hyperglycemia |
| Diabetes Complicated | E10.69 | Type 1 diabetes mellitus with other specified complication |
| Diabetes Complicated | E10.8 | Type 1 diabetes mellitus with unspecified complications |
| Diabetes Complicated | E11.21 | Type 2 diabetes mellitus with diabetic nephropathy |
| Diabetes Complicated | E11.22 | Type 2 diabetes mellitus with diabetic chronic kidney disease |
| Diabetes Complicated | E11.29 | Type 2 diabetes mellitus with other diabetic kidney complication |
| Diabetes Complicated | E11.311 | Type 2 diabetes mellitus with unspecified diabetic retinopathy with macular edema |
| Diabetes Complicated | E11.319 | Type 2 diabetes mellitus with unspecified diabetic retinopathy without macular edema |
| Diabetes Complicated | E11.321 | Type 2 diabetes mellitus with mild nonproliferative diabetic retinopathy with macular edema |
| Diabetes Complicated | E11.329 | Type 2 diabetes mellitus with mild nonproliferative diabetic retinopathy without macular edema |
| Diabetes Complicated | E11.331 | Type 2 diabetes mellitus with moderate nonproliferative diabetic retinopathy with macular edema |
| Diabetes Complicated | E11.339 | Type 2 diabetes mellitus with moderate nonproliferative diabetic retinopathy without macular edema |
| Diabetes Complicated | E11.341 | Type 2 diabetes mellitus with severe nonproliferative diabetic retinopathy with macular edema |
| Diabetes Complicated | E11.349 | Type 2 diabetes mellitus with severe nonproliferative diabetic retinopathy without macular edema |
| Diabetes Complicated | E11.351 | Type 2 diabetes mellitus with proliferative diabetic retinopathy with macular edema |
| Diabetes Complicated | E11.359 | Type 2 diabetes mellitus with proliferative diabetic retinopathy without macular edema |
| Diabetes Complicated | E11.36 | Type 2 diabetes mellitus with diabetic cataract |
| Diabetes Complicated | E11.39 | Type 2 diabetes mellitus with other diabetic ophthalmic complication |
| Diabetes Complicated | E11.40 | Type 2 diabetes mellitus with diabetic neuropathy, unspecified |
| Diabetes Complicated | E11.41 | Type 2 diabetes mellitus with diabetic mononeuropathy |
| Diabetes Complicated | E11.42 | Type 2 diabetes mellitus with diabetic polyneuropathy |
| Diabetes Complicated | E11.43 | Type 2 diabetes mellitus with diabetic autonomic (poly)neuropathy |
| Diabetes Complicated | E11.44 | Type 2 diabetes mellitus with diabetic amyotrophy |
| Diabetes Complicated | E11.49 | Type 2 diabetes mellitus with other diabetic neurological complication |
| Diabetes Complicated | E11.51 | Type 2 diabetes mellitus with diabetic peripheral angiopathy without gangrene |
| Diabetes Complicated | E11.52 | Type 2 diabetes mellitus with diabetic peripheral angiopathy with gangrene |
| Diabetes Complicated | E11.59 | Type 2 diabetes mellitus with other circulatory complications |
| Diabetes Complicated | E11.610 | Type 2 diabetes mellitus with diabetic neuropathic arthropathy |
| Diabetes Complicated | E11.618 | Type 2 diabetes mellitus with other diabetic arthropathy |
| Diabetes Complicated | E11.620 | Type 2 diabetes mellitus with diabetic dermatitis |
| Diabetes Complicated | E11.621 | Type 2 diabetes mellitus with foot ulcer |
| Diabetes Complicated | E11.622 | Type 2 diabetes mellitus with other skin ulcer |
| Diabetes Complicated | E11.628 | Type 2 diabetes mellitus with other skin complications |
| Diabetes Complicated | E11.630 | Type 2 diabetes mellitus with periodontal disease |
| Diabetes Complicated | E11.638 | Type 2 diabetes mellitus with other oral complications |
| Diabetes Complicated | E11.641 | Type 2 diabetes mellitus with hypoglycemia with coma |
| Diabetes Complicated | E11.649 | Type 2 diabetes mellitus with hypoglycemia without coma |
| Diabetes Complicated | E11.65 | Type 2 diabetes mellitus with hyperglycemia |
| Diabetes Complicated | E11.69 | Type 2 diabetes mellitus with other specified complication |
| Diabetes Complicated | E11.8 | Type 2 diabetes mellitus with unspecified complications |
| Diabetes Complicated | E13.21 | Other specified diabetes mellitus with diabetic nephropathy |
| Diabetes Complicated | E13.22 | Other specified diabetes mellitus with diabetic chronic kidney disease |
| Diabetes Complicated | E13.29 | Other specified diabetes mellitus with other diabetic kidney complication |
| Diabetes Complicated | E13.311 | Other specified diabetes mellitus with unspecified diabetic retinopathy with macular edema |
| Diabetes Complicated | E13.319 | Other specified diabetes mellitus with unspecified diabetic retinopathy without macular edema |
| Diabetes Complicated | E13.321 | Other specified diabetes mellitus with mild nonproliferative diabetic retinopathy with macular edema |
| Diabetes Complicated | E13.329 | Other specified diabetes mellitus with mild nonproliferative diabetic retinopathy without macular edema |
| Diabetes Complicated | E13.331 | Other specified diabetes mellitus with moderate nonproliferative diabetic retinopathy with macular edema |
| Diabetes Complicated | E13.339 | Other specified diabetes mellitus with moderate nonproliferative diabetic retinopathy without macular edema |
| Diabetes Complicated | E13.341 | Other specified diabetes mellitus with severe nonproliferative diabetic retinopathy with macular edema |
| Diabetes Complicated | E13.349 | Other specified diabetes mellitus with severe nonproliferative diabetic retinopathy without macular edema |
| Diabetes Complicated | E13.351 | Other specified diabetes mellitus with proliferative diabetic retinopathy with macular edema |
| Diabetes Complicated | E13.359 | Other specified diabetes mellitus with proliferative diabetic retinopathy without macular edema |
| Diabetes Complicated | E13.36 | Other specified diabetes mellitus with diabetic cataract |
| Diabetes Complicated | E13.39 | Other specified diabetes mellitus with other diabetic ophthalmic complication |
| Diabetes Complicated | E13.40 | Other specified diabetes mellitus with diabetic neuropathy, unspecified |
| Diabetes Complicated | E13.41 | Other specified diabetes mellitus with diabetic mononeuropathy |
| Diabetes Complicated | E13.42 | Other specified diabetes mellitus with diabetic polyneuropathy |
| Diabetes Complicated | E13.43 | Other specified diabetes mellitus with diabetic autonomic (poly)neuropathy |
| Diabetes Complicated | E13.44 | Other specified diabetes mellitus with diabetic amyotrophy |
| Diabetes Complicated | E13.49 | Other specified diabetes mellitus with other diabetic neurological complication |
| Diabetes Complicated | E13.51 | Other specified diabetes mellitus with diabetic peripheral angiopathy without gangrene |
| Diabetes Complicated | E13.52 | Other specified diabetes mellitus with diabetic peripheral angiopathy with gangrene |
| Diabetes Complicated | E13.59 | Other specified diabetes mellitus with other circulatory complications |
| Diabetes Complicated | E13.610 | Other specified diabetes mellitus with diabetic neuropathic arthropathy |
| Diabetes Complicated | E13.618 | Other specified diabetes mellitus with other diabetic arthropathy |
| Diabetes Complicated | E13.620 | Other specified diabetes mellitus with diabetic dermatitis |
| Diabetes Complicated | E13.621 | Other specified diabetes mellitus with foot ulcer |
| Diabetes Complicated | E13.622 | Other specified diabetes mellitus with other skin ulcer |
| Diabetes Complicated | E13.628 | Other specified diabetes mellitus with other skin complications |
| Diabetes Complicated | E13.630 | Other specified diabetes mellitus with periodontal disease |
| Diabetes Complicated | E13.638 | Other specified diabetes mellitus with other oral complications |
| Diabetes Complicated | E13.641 | Other specified diabetes mellitus with hypoglycemia with coma |
| Diabetes Complicated | E13.649 | Other specified diabetes mellitus with hypoglycemia without coma |
| Diabetes Complicated | E13.65 | Other specified diabetes mellitus with hyperglycemia |
| Diabetes Complicated | E13.69 | Other specified diabetes mellitus with other specified complication |
| Diabetes Complicated | E13.8 | Other specified diabetes mellitus with unspecified complications |
| Diabetes Uncomplicated | E10.10 | Type 1 diabetes mellitus with ketoacidosis without coma |
| Diabetes Uncomplicated | E10.11 | Type 1 diabetes mellitus with ketoacidosis with coma |
| Diabetes Uncomplicated | E10.9 | Type 1 diabetes mellitus without complications |
| Diabetes Uncomplicated | E11.00 | Type 2 diabetes mellitus with hyperosmolarity without nonketotic hyperglycemic-hyperosmolar coma (NKHHC) |
| Diabetes Uncomplicated | E11.01 | Type 2 diabetes mellitus with hyperosmolarity with coma |
| Diabetes Uncomplicated | E11.9 | Type 2 diabetes mellitus without complications |
| Diabetes Uncomplicated | E13.00 | Other specified diabetes mellitus with hyperosmolarity without nonketotic hyperglycemic-hyperosmolar coma (NKHHC) |
| Diabetes Uncomplicated | E13.01 | Other specified diabetes mellitus with hyperosmolarity with coma |
| Diabetes Uncomplicated | E13.10 | Other specified diabetes mellitus with ketoacidosis without coma |
| Diabetes Uncomplicated | E13.11 | Other specified diabetes mellitus with ketoacidosis with coma |
| Diabetes Uncomplicated | E13.9 | Other specified diabetes mellitus without complications |
| Drug Abuse | F11.10 | Opioid abuse, uncomplicated |
| Drug Abuse | F11.120 | Opioid abuse with intoxication, uncomplicated |
| Drug Abuse | F11.121 | Opioid abuse with intoxication delirium |
| Drug Abuse | F11.122 | Opioid abuse with intoxication with perceptual disturbance |
| Drug Abuse | F11.129 | Opioid abuse with intoxication, unspecified |
| Drug Abuse | F11.14 | Opioid abuse with opioid-induced mood disorder |
| Drug Abuse | F11.150 | Opioid abuse with opioid-induced psychotic disorder with delusions |
| Drug Abuse | F11.151 | Opioid abuse with opioid-induced psychotic disorder with hallucinations |
| Drug Abuse | F11.159 | Opioid abuse with opioid-induced psychotic disorder, unspecified |
| Drug Abuse | F11.181 | Opioid abuse with opioid-induced sexual dysfunction |
| Drug Abuse | F11.182 | Opioid abuse with opioid-induced sleep disorder |
| Drug Abuse | F11.188 | Opioid abuse with other opioid-induced disorder |
| Drug Abuse | F11.19 | Opioid abuse with unspecified opioid-induced disorder |
| Drug Abuse | F11.20 | Opioid dependence, uncomplicated |
| Drug Abuse | F11.21 | Opioid dependence, in remission |
| Drug Abuse | F11.220 | Opioid dependence with intoxication, uncomplicated |
| Drug Abuse | F11.221 | Opioid dependence with intoxication delirium |
| Drug Abuse | F11.222 | Opioid dependence with intoxication with perceptual disturbance |
| Drug Abuse | F11.229 | Opioid dependence with intoxication, unspecified |
| Drug Abuse | F11.23 | Opioid dependence with withdrawal |
| Drug Abuse | F11.24 | Opioid dependence with opioid-induced mood disorder |
| Drug Abuse | F11.250 | Opioid dependence with opioid-induced psychotic disorder with delusions |
| Drug Abuse | F11.251 | Opioid dependence with opioid-induced psychotic disorder with hallucinations |
| Drug Abuse | F11.259 | Opioid dependence with opioid-induced psychotic disorder, unspecified |
| Drug Abuse | F11.281 | Opioid dependence with opioid-induced sexual dysfunction |
| Drug Abuse | F11.282 | Opioid dependence with opioid-induced sleep disorder |
| Drug Abuse | F11.288 | Opioid dependence with other opioid-induced disorder |
| Drug Abuse | F11.29 | Opioid dependence with unspecified opioid-induced disorder |
| Drug Abuse | F11.90 | Opioid use, unspecified, uncomplicated |
| Drug Abuse | F11.920 | Opioid use, unspecified with intoxication, uncomplicated |
| Drug Abuse | F11.921 | Opioid use, unspecified with intoxication delirium |
| Drug Abuse | F11.922 | Opioid use, unspecified with intoxication with perceptual disturbance |
| Drug Abuse | F11.929 | Opioid use, unspecified with intoxication, unspecified |
| Drug Abuse | F11.93 | Opioid use, unspecified with withdrawal |
| Drug Abuse | F11.94 | Opioid use, unspecified with opioid-induced mood disorder |
| Drug Abuse | F11.950 | Opioid use, unspecified with opioid-induced psychotic disorder with delusions |
| Drug Abuse | F11.951 | Opioid use, unspecified with opioid-induced psychotic disorder with hallucinations |
| Drug Abuse | F11.959 | Opioid use, unspecified with opioid-induced psychotic disorder, unspecified |
| Drug Abuse | F11.981 | Opioid use, unspecified with opioid-induced sexual dysfunction |
| Drug Abuse | F11.982 | Opioid use, unspecified with opioid-induced sleep disorder |
| Drug Abuse | F11.988 | Opioid use, unspecified with other opioid-induced disorder |
| Drug Abuse | F11.99 | Opioid use, unspecified with unspecified opioid-induced disorder |
| Drug Abuse | F12.10 | Cannabis abuse, uncomplicated |
| Drug Abuse | F12.120 | Cannabis abuse with intoxication, uncomplicated |
| Drug Abuse | F12.121 | Cannabis abuse with intoxication delirium |
| Drug Abuse | F12.122 | Cannabis abuse with intoxication with perceptual disturbance |
| Drug Abuse | F12.129 | Cannabis abuse with intoxication, unspecified |
| Drug Abuse | F12.150 | Cannabis abuse with psychotic disorder with delusions |
| Drug Abuse | F12.151 | Cannabis abuse with psychotic disorder with hallucinations |
| Drug Abuse | F12.159 | Cannabis abuse with psychotic disorder, unspecified |
| Drug Abuse | F12.180 | Cannabis abuse with cannabis-induced anxiety disorder |
| Drug Abuse | F12.188 | Cannabis abuse with other cannabis-induced disorder |
| Drug Abuse | F12.19 | Cannabis abuse with unspecified cannabis-induced disorder |
| Drug Abuse | F12.20 | Cannabis dependence, uncomplicated |
| Drug Abuse | F12.21 | Cannabis dependence, in remission |
| Drug Abuse | F12.220 | Cannabis dependence with intoxication, uncomplicated |
| Drug Abuse | F12.221 | Cannabis dependence with intoxication delirium |
| Drug Abuse | F12.222 | Cannabis dependence with intoxication with perceptual disturbance |
| Drug Abuse | F12.229 | Cannabis dependence with intoxication, unspecified |
| Drug Abuse | F12.250 | Cannabis dependence with psychotic disorder with delusions |
| Drug Abuse | F12.251 | Cannabis dependence with psychotic disorder with hallucinations |
| Drug Abuse | F12.259 | Cannabis dependence with psychotic disorder, unspecified |
| Drug Abuse | F12.280 | Cannabis dependence with cannabis-induced anxiety disorder |
| Drug Abuse | F12.288 | Cannabis dependence with other cannabis-induced disorder |
| Drug Abuse | F12.29 | Cannabis dependence with unspecified cannabis-induced disorder |
| Drug Abuse | F12.90 | Cannabis use, unspecified, uncomplicated |
| Drug Abuse | F12.920 | Cannabis use, unspecified with intoxication, uncomplicated |
| Drug Abuse | F12.921 | Cannabis use, unspecified with intoxication delirium |
| Drug Abuse | F12.922 | Cannabis use, unspecified with intoxication with perceptual disturbance |
| Drug Abuse | F12.929 | Cannabis use, unspecified with intoxication, unspecified |
| Drug Abuse | F12.950 | Cannabis use, unspecified with psychotic disorder with delusions |
| Drug Abuse | F12.951 | Cannabis use, unspecified with psychotic disorder with hallucinations |
| Drug Abuse | F12.959 | Cannabis use, unspecified with psychotic disorder, unspecified |
| Drug Abuse | F12.980 | Cannabis use, unspecified with anxiety disorder |
| Drug Abuse | F12.988 | Cannabis use, unspecified with other cannabis-induced disorder |
| Drug Abuse | F12.99 | Cannabis use, unspecified with unspecified cannabis-induced disorder |
| Drug Abuse | F13.10 | Sedative, hypnotic or anxiolytic abuse, uncomplicated |
| Drug Abuse | F13.120 | Sedative, hypnotic or anxiolytic abuse with intoxication, uncomplicated |
| Drug Abuse | F13.121 | Sedative, hypnotic or anxiolytic abuse with intoxication delirium |
| Drug Abuse | F13.129 | Sedative, hypnotic or anxiolytic abuse with intoxication, unspecified |
| Drug Abuse | F13.14 | Sedative, hypnotic or anxiolytic abuse with sedative, hypnotic or anxiolytic-induced mood disorder |
| Drug Abuse | F13.150 | Sedative, hypnotic or anxiolytic abuse with sedative, hypnotic or anxiolytic-induced psychotic disorder with delusions |
| Drug Abuse | F13.151 | Sedative, hypnotic or anxiolytic abuse with sedative, hypnotic or anxiolytic-induced psychotic disorder with hallucinations |
| Drug Abuse | F13.159 | Sedative, hypnotic or anxiolytic abuse with sedative, hypnotic or anxiolytic-induced psychotic disorder, unspecified |
| Drug Abuse | F13.180 | Sedative, hypnotic or anxiolytic abuse with sedative, hypnotic or anxiolytic-induced anxiety disorder |
| Drug Abuse | F13.181 | Sedative, hypnotic or anxiolytic abuse with sedative, hypnotic or anxiolytic-induced sexual dysfunction |
| Drug Abuse | F13.182 | Sedative, hypnotic or anxiolytic abuse with sedative, hypnotic or anxiolytic-induced sleep disorder |
| Drug Abuse | F13.188 | Sedative, hypnotic or anxiolytic abuse with other sedative, hypnotic or anxiolytic-induced disorder |
| Drug Abuse | F13.19 | Sedative, hypnotic or anxiolytic abuse with unspecified sedative, hypnotic or anxiolytic-induced disorder |
| Drug Abuse | F13.20 | Sedative, hypnotic or anxiolytic dependence, uncomplicated |
| Drug Abuse | F13.21 | Sedative, hypnotic or anxiolytic dependence, in remission |
| Drug Abuse | F13.220 | Sedative, hypnotic or anxiolytic dependence with intoxication, uncomplicated |
| Drug Abuse | F13.221 | Sedative, hypnotic or anxiolytic dependence with intoxication delirium |
| Drug Abuse | F13.229 | Sedative, hypnotic or anxiolytic dependence with intoxication, unspecified |
| Drug Abuse | F13.230 | Sedative, hypnotic or anxiolytic dependence with withdrawal, uncomplicated |
| Drug Abuse | F13.231 | Sedative, hypnotic or anxiolytic dependence with withdrawal delirium |
| Drug Abuse | F13.232 | Sedative, hypnotic or anxiolytic dependence with withdrawal with perceptual disturbance |
| Drug Abuse | F13.239 | Sedative, hypnotic or anxiolytic dependence with withdrawal, unspecified |
| Drug Abuse | F13.24 | Sedative, hypnotic or anxiolytic dependence with sedative, hypnotic or anxiolytic-induced mood disorder |
| Drug Abuse | F13.250 | Sedative, hypnotic or anxiolytic dependence with sedative, hypnotic or anxiolytic-induced psychotic disorder with delusions |
| Drug Abuse | F13.251 | Sedative, hypnotic or anxiolytic dependence with sedative, hypnotic or anxiolytic-induced psychotic disorder with hallucinations |
| Drug Abuse | F13.259 | Sedative, hypnotic or anxiolytic dependence with sedative, hypnotic or anxiolytic-induced psychotic disorder, unspecified |
| Drug Abuse | F13.26 | Sedative, hypnotic or anxiolytic dependence with sedative, hypnotic or anxiolytic-induced persisting amnestic disorder |
| Drug Abuse | F13.27 | Sedative, hypnotic or anxiolytic dependence with sedative, hypnotic or anxiolytic-induced persisting dementia |
| Drug Abuse | F13.280 | Sedative, hypnotic or anxiolytic dependence with sedative, hypnotic or anxiolytic-induced anxiety disorder |
| Drug Abuse | F13.281 | Sedative, hypnotic or anxiolytic dependence with sedative, hypnotic or anxiolytic-induced sexual dysfunction |
| Drug Abuse | F13.282 | Sedative, hypnotic or anxiolytic dependence with sedative, hypnotic or anxiolytic-induced sleep disorder |
| Drug Abuse | F13.288 | Sedative, hypnotic or anxiolytic dependence with other sedative, hypnotic or anxiolytic-induced disorder |
| Drug Abuse | F13.29 | Sedative, hypnotic or anxiolytic dependence with unspecified sedative, hypnotic or anxiolytic-induced disorder |
| Drug Abuse | F13.90 | Sedative, hypnotic, or anxiolytic use, unspecified, uncomplicated |
| Drug Abuse | F13.920 | Sedative, hypnotic or anxiolytic use, unspecified with intoxication, uncomplicated |
| Drug Abuse | F13.921 | Sedative, hypnotic or anxiolytic use, unspecified with intoxication delirium |
| Drug Abuse | F13.929 | Sedative, hypnotic or anxiolytic use, unspecified with intoxication, unspecified |
| Drug Abuse | F13.930 | Sedative, hypnotic or anxiolytic use, unspecified with withdrawal, uncomplicated |
| Drug Abuse | F13.931 | Sedative, hypnotic or anxiolytic use, unspecified with withdrawal delirium |
| Drug Abuse | F13.932 | Sedative, hypnotic or anxiolytic use, unspecified with withdrawal with perceptual disturbances |
| Drug Abuse | F13.939 | Sedative, hypnotic or anxiolytic use, unspecified with withdrawal, unspecified |
| Drug Abuse | F13.94 | Sedative, hypnotic or anxiolytic use, unspecified with sedative, hypnotic or anxiolytic-induced mood disorder |
| Drug Abuse | F13.950 | Sedative, hypnotic or anxiolytic use, unspecified with sedative, hypnotic or anxiolytic-induced psychotic disorder with delusions |
| Drug Abuse | F13.951 | Sedative, hypnotic or anxiolytic use, unspecified with sedative, hypnotic or anxiolytic-induced psychotic disorder with hallucinations |
| Drug Abuse | F13.959 | Sedative, hypnotic or anxiolytic use, unspecified with sedative, hypnotic or anxiolytic-induced psychotic disorder, unspecified |
| Drug Abuse | F13.96 | Sedative, hypnotic or anxiolytic use, unspecified with sedative, hypnotic or anxiolytic-induced persisting amnestic disorder |
| Drug Abuse | F13.97 | Sedative, hypnotic or anxiolytic use, unspecified with sedative, hypnotic or anxiolytic-induced persisting dementia |
| Drug Abuse | F13.980 | Sedative, hypnotic or anxiolytic use, unspecified with sedative, hypnotic or anxiolytic-induced anxiety disorder |
| Drug Abuse | F13.981 | Sedative, hypnotic or anxiolytic use, unspecified with sedative, hypnotic or anxiolytic-induced sexual dysfunction |
| Drug Abuse | F13.982 | Sedative, hypnotic or anxiolytic use, unspecified with sedative, hypnotic or anxiolytic-induced sleep disorder |
| Drug Abuse | F13.988 | Sedative, hypnotic or anxiolytic use, unspecified with other sedative, hypnotic or anxiolytic-induced disorder |
| Drug Abuse | F13.99 | Sedative, hypnotic or anxiolytic use, unspecified with unspecified sedative, hypnotic or anxiolytic-induced disorder |
| Drug Abuse | F14.10 | Cocaine abuse, uncomplicated |
| Drug Abuse | F14.120 | Cocaine abuse with intoxication, uncomplicated |
| Drug Abuse | F14.121 | Cocaine abuse with intoxication with delirium |
| Drug Abuse | F14.122 | Cocaine abuse with intoxication with perceptual disturbance |
| Drug Abuse | F14.129 | Cocaine abuse with intoxication, unspecified |
| Drug Abuse | F14.14 | Cocaine abuse with cocaine-induced mood disorder |
| Drug Abuse | F14.150 | Cocaine abuse with cocaine-induced psychotic disorder with delusions |
| Drug Abuse | F14.151 | Cocaine abuse with cocaine-induced psychotic disorder with hallucinations |
| Drug Abuse | F14.159 | Cocaine abuse with cocaine-induced psychotic disorder, unspecified |
| Drug Abuse | F14.180 | Cocaine abuse with cocaine-induced anxiety disorder |
| Drug Abuse | F14.181 | Cocaine abuse with cocaine-induced sexual dysfunction |
| Drug Abuse | F14.182 | Cocaine abuse with cocaine-induced sleep disorder |
| Drug Abuse | F14.188 | Cocaine abuse with other cocaine-induced disorder |
| Drug Abuse | F14.19 | Cocaine abuse with unspecified cocaine-induced disorder |
| Drug Abuse | F14.20 | Cocaine dependence, uncomplicated |
| Drug Abuse | F14.21 | Cocaine dependence, in remission |
| Drug Abuse | F14.220 | Cocaine dependence with intoxication, uncomplicated |
| Drug Abuse | F14.221 | Cocaine dependence with intoxication delirium |
| Drug Abuse | F14.222 | Cocaine dependence with intoxication with perceptual disturbance |
| Drug Abuse | F14.229 | Cocaine dependence with intoxication, unspecified |
| Drug Abuse | F14.23 | Cocaine dependence with withdrawal |
| Drug Abuse | F14.24 | Cocaine dependence with cocaine-induced mood disorder |
| Drug Abuse | F14.250 | Cocaine dependence with cocaine-induced psychotic disorder with delusions |
| Drug Abuse | F14.251 | Cocaine dependence with cocaine-induced psychotic disorder with hallucinations |
| Drug Abuse | F14.259 | Cocaine dependence with cocaine-induced psychotic disorder, unspecified |
| Drug Abuse | F14.280 | Cocaine dependence with cocaine-induced anxiety disorder |
| Drug Abuse | F14.281 | Cocaine dependence with cocaine-induced sexual dysfunction |
| Drug Abuse | F14.282 | Cocaine dependence with cocaine-induced sleep disorder |
| Drug Abuse | F14.288 | Cocaine dependence with other cocaine-induced disorder |
| Drug Abuse | F14.29 | Cocaine dependence with unspecified cocaine-induced disorder |
| Drug Abuse | F14.90 | Cocaine use, unspecified, uncomplicated |
| Drug Abuse | F14.920 | Cocaine use, unspecified with intoxication, uncomplicated |
| Drug Abuse | F14.921 | Cocaine use, unspecified with intoxication delirium |
| Drug Abuse | F14.922 | Cocaine use, unspecified with intoxication with perceptual disturbance |
| Drug Abuse | F14.929 | Cocaine use, unspecified with intoxication, unspecified |
| Drug Abuse | F14.94 | Cocaine use, unspecified with cocaine-induced mood disorder |
| Drug Abuse | F14.950 | Cocaine use, unspecified with cocaine-induced psychotic disorder with delusions |
| Drug Abuse | F14.951 | Cocaine use, unspecified with cocaine-induced psychotic disorder with hallucinations |
| Drug Abuse | F14.959 | Cocaine use, unspecified with cocaine-induced psychotic disorder, unspecified |
| Drug Abuse | F14.980 | Cocaine use, unspecified with cocaine-induced anxiety disorder |
| Drug Abuse | F14.981 | Cocaine use, unspecified with cocaine-induced sexual dysfunction |
| Drug Abuse | F14.982 | Cocaine use, unspecified with cocaine-induced sleep disorder |
| Drug Abuse | F14.988 | Cocaine use, unspecified with other cocaine-induced disorder |
| Drug Abuse | F14.99 | Cocaine use, unspecified with unspecified cocaine-induced disorder |
| Drug Abuse | F15.10 | Other stimulant abuse, uncomplicated |
| Drug Abuse | F15.120 | Other stimulant abuse with intoxication, uncomplicated |
| Drug Abuse | F15.121 | Other stimulant abuse with intoxication delirium |
| Drug Abuse | F15.122 | Other stimulant abuse with intoxication with perceptual disturbance |
| Drug Abuse | F15.129 | Other stimulant abuse with intoxication, unspecified |
| Drug Abuse | F15.14 | Other stimulant abuse with stimulant-induced mood disorder |
| Drug Abuse | F15.150 | Other stimulant abuse with stimulant-induced psychotic disorder with delusions |
| Drug Abuse | F15.151 | Other stimulant abuse with stimulant-induced psychotic disorder with hallucinations |
| Drug Abuse | F15.159 | Other stimulant abuse with stimulant-induced psychotic disorder, unspecified |
| Drug Abuse | F15.180 | Other stimulant abuse with stimulant-induced anxiety disorder |
| Drug Abuse | F15.181 | Other stimulant abuse with stimulant-induced sexual dysfunction |
| Drug Abuse | F15.182 | Other stimulant abuse with stimulant-induced sleep disorder |
| Drug Abuse | F15.188 | Other stimulant abuse with other stimulant-induced disorder |
| Drug Abuse | F15.19 | Other stimulant abuse with unspecified stimulant-induced disorder |
| Drug Abuse | F15.20 | Other stimulant dependence, uncomplicated |
| Drug Abuse | F15.21 | Other stimulant dependence, in remission |
| Drug Abuse | F15.220 | Other stimulant dependence with intoxication, uncomplicated |
| Drug Abuse | F15.221 | Other stimulant dependence with intoxication delirium |
| Drug Abuse | F15.222 | Other stimulant dependence with intoxication with perceptual disturbance |
| Drug Abuse | F15.229 | Other stimulant dependence with intoxication, unspecified |
| Drug Abuse | F15.23 | Other stimulant dependence with withdrawal |
| Drug Abuse | F15.24 | Other stimulant dependence with stimulant-induced mood disorder |
| Drug Abuse | F15.250 | Other stimulant dependence with stimulant-induced psychotic disorder with delusions |
| Drug Abuse | F15.251 | Other stimulant dependence with stimulant-induced psychotic disorder with hallucinations |
| Drug Abuse | F15.259 | Other stimulant dependence with stimulant-induced psychotic disorder, unspecified |
| Drug Abuse | F15.280 | Other stimulant dependence with stimulant-induced anxiety disorder |
| Drug Abuse | F15.281 | Other stimulant dependence with stimulant-induced sexual dysfunction |
| Drug Abuse | F15.282 | Other stimulant dependence with stimulant-induced sleep disorder |
| Drug Abuse | F15.288 | Other stimulant dependence with other stimulant-induced disorder |
| Drug Abuse | F15.29 | Other stimulant dependence with unspecified stimulant-induced disorder |
| Drug Abuse | F15.90 | Other stimulant use, unspecified, uncomplicated |
| Drug Abuse | F15.920 | Other stimulant use, unspecified with intoxication, uncomplicated |
| Drug Abuse | F15.921 | Other stimulant use, unspecified with intoxication delirium |
| Drug Abuse | F15.922 | Other stimulant use, unspecified with intoxication with perceptual disturbance |
| Drug Abuse | F15.929 | Other stimulant use, unspecified with intoxication, unspecified |
| Drug Abuse | F15.93 | Other stimulant use, unspecified with withdrawal |
| Drug Abuse | F15.94 | Other stimulant use, unspecified with stimulant-induced mood disorder |
| Drug Abuse | F15.950 | Other stimulant use, unspecified with stimulant-induced psychotic disorder with delusions |
| Drug Abuse | F15.951 | Other stimulant use, unspecified with stimulant-induced psychotic disorder with hallucinations |
| Drug Abuse | F15.959 | Other stimulant use, unspecified with stimulant-induced psychotic disorder, unspecified |
| Drug Abuse | F15.980 | Other stimulant use, unspecified with stimulant-induced anxiety disorder |
| Drug Abuse | F15.981 | Other stimulant use, unspecified with stimulant-induced sexual dysfunction |
| Drug Abuse | F15.982 | Other stimulant use, unspecified with stimulant-induced sleep disorder |
| Drug Abuse | F15.988 | Other stimulant use, unspecified with other stimulant-induced disorder |
| Drug Abuse | F15.99 | Other stimulant use, unspecified with unspecified stimulant-induced disorder |
| Drug Abuse | F16.10 | Hallucinogen abuse, uncomplicated |
| Drug Abuse | F16.120 | Hallucinogen abuse with intoxication, uncomplicated |
| Drug Abuse | F16.121 | Hallucinogen abuse with intoxication with delirium |
| Drug Abuse | F16.122 | Hallucinogen abuse with intoxication with perceptual disturbance |
| Drug Abuse | F16.129 | Hallucinogen abuse with intoxication, unspecified |
| Drug Abuse | F16.14 | Hallucinogen abuse with hallucinogen-induced mood disorder |
| Drug Abuse | F16.150 | Hallucinogen abuse with hallucinogen-induced psychotic disorder with delusions |
| Drug Abuse | F16.151 | Hallucinogen abuse with hallucinogen-induced psychotic disorder with hallucinations |
| Drug Abuse | F16.159 | Hallucinogen abuse with hallucinogen-induced psychotic disorder, unspecified |
| Drug Abuse | F16.180 | Hallucinogen abuse with hallucinogen-induced anxiety disorder |
| Drug Abuse | F16.183 | Hallucinogen abuse with hallucinogen persisting perception disorder (flashbacks) |
| Drug Abuse | F16.188 | Hallucinogen abuse with other hallucinogen-induced disorder |
| Drug Abuse | F16.19 | Hallucinogen abuse with unspecified hallucinogen-induced disorder |
| Drug Abuse | F16.20 | Hallucinogen dependence, uncomplicated |
| Drug Abuse | F16.21 | Hallucinogen dependence, in remission |
| Drug Abuse | F16.220 | Hallucinogen dependence with intoxication, uncomplicated |
| Drug Abuse | F16.221 | Hallucinogen dependence with intoxication with delirium |
| Drug Abuse | F16.229 | Hallucinogen dependence with intoxication, unspecified |
| Drug Abuse | F16.24 | Hallucinogen dependence with hallucinogen-induced mood disorder |
| Drug Abuse | F16.250 | Hallucinogen dependence with hallucinogen-induced psychotic disorder with delusions |
| Drug Abuse | F16.251 | Hallucinogen dependence with hallucinogen-induced psychotic disorder with hallucinations |
| Drug Abuse | F16.259 | Hallucinogen dependence with hallucinogen-induced psychotic disorder, unspecified |
| Drug Abuse | F16.280 | Hallucinogen dependence with hallucinogen-induced anxiety disorder |
| Drug Abuse | F16.283 | Hallucinogen dependence with hallucinogen persisting perception disorder (flashbacks) |
| Drug Abuse | F16.288 | Hallucinogen dependence with other hallucinogen-induced disorder |
| Drug Abuse | F16.29 | Hallucinogen dependence with unspecified hallucinogen-induced disorder |
| Drug Abuse | F16.90 | Hallucinogen use, unspecified, uncomplicated |
| Drug Abuse | F16.920 | Hallucinogen use, unspecified with intoxication, uncomplicated |
| Drug Abuse | F16.921 | Hallucinogen use, unspecified with intoxication with delirium |
| Drug Abuse | F16.929 | Hallucinogen use, unspecified with intoxication, unspecified |
| Drug Abuse | F16.94 | Hallucinogen use, unspecified with hallucinogen-induced mood disorder |
| Drug Abuse | F16.950 | Hallucinogen use, unspecified with hallucinogen-induced psychotic disorder with delusions |
| Drug Abuse | F16.951 | Hallucinogen use, unspecified with hallucinogen-induced psychotic disorder with hallucinations |
| Drug Abuse | F16.959 | Hallucinogen use, unspecified with hallucinogen-induced psychotic disorder, unspecified |
| Drug Abuse | F16.980 | Hallucinogen use, unspecified with hallucinogen-induced anxiety disorder |
| Drug Abuse | F16.983 | Hallucinogen use, unspecified with hallucinogen persisting perception disorder (flashbacks) |
| Drug Abuse | F16.988 | Hallucinogen use, unspecified with other hallucinogen-induced disorder |
| Drug Abuse | F16.99 | Hallucinogen use, unspecified with unspecified hallucinogen-induced disorder |
| Drug Abuse | F18.10 | Inhalant abuse, uncomplicated |
| Drug Abuse | F18.120 | Inhalant abuse with intoxication, uncomplicated |
| Drug Abuse | F18.121 | Inhalant abuse with intoxication delirium |
| Drug Abuse | F18.129 | Inhalant abuse with intoxication, unspecified |
| Drug Abuse | F18.14 | Inhalant abuse with inhalant-induced mood disorder |
| Drug Abuse | F18.150 | Inhalant abuse with inhalant-induced psychotic disorder with delusions |
| Drug Abuse | F18.151 | Inhalant abuse with inhalant-induced psychotic disorder with hallucinations |
| Drug Abuse | F18.159 | Inhalant abuse with inhalant-induced psychotic disorder, unspecified |
| Drug Abuse | F18.17 | Inhalant abuse with inhalant-induced dementia |
| Drug Abuse | F18.180 | Inhalant abuse with inhalant-induced anxiety disorder |
| Drug Abuse | F18.188 | Inhalant abuse with other inhalant-induced disorder |
| Drug Abuse | F18.19 | Inhalant abuse with unspecified inhalant-induced disorder |
| Drug Abuse | F18.20 | Inhalant dependence, uncomplicated |
| Drug Abuse | F18.21 | Inhalant dependence, in remission |
| Drug Abuse | F18.220 | Inhalant dependence with intoxication, uncomplicated |
| Drug Abuse | F18.221 | Inhalant dependence with intoxication delirium |
| Drug Abuse | F18.229 | Inhalant dependence with intoxication, unspecified |
| Drug Abuse | F18.24 | Inhalant dependence with inhalant-induced mood disorder |
| Drug Abuse | F18.250 | Inhalant dependence with inhalant-induced psychotic disorder with delusions |
| Drug Abuse | F18.251 | Inhalant dependence with inhalant-induced psychotic disorder with hallucinations |
| Drug Abuse | F18.259 | Inhalant dependence with inhalant-induced psychotic disorder, unspecified |
| Drug Abuse | F18.27 | Inhalant dependence with inhalant-induced dementia |
| Drug Abuse | F18.280 | Inhalant dependence with inhalant-induced anxiety disorder |
| Drug Abuse | F18.288 | Inhalant dependence with other inhalant-induced disorder |
| Drug Abuse | F18.29 | Inhalant dependence with unspecified inhalant-induced disorder |
| Drug Abuse | F18.90 | Inhalant use, unspecified, uncomplicated |
| Drug Abuse | F18.920 | Inhalant use, unspecified with intoxication, uncomplicated |
| Drug Abuse | F18.921 | Inhalant use, unspecified with intoxication with delirium |
| Drug Abuse | F18.929 | Inhalant use, unspecified with intoxication, unspecified |
| Drug Abuse | F18.94 | Inhalant use, unspecified with inhalant-induced mood disorder |
| Drug Abuse | F18.950 | Inhalant use, unspecified with inhalant-induced psychotic disorder with delusions |
| Drug Abuse | F18.951 | Inhalant use, unspecified with inhalant-induced psychotic disorder with hallucinations |
| Drug Abuse | F18.959 | Inhalant use, unspecified with inhalant-induced psychotic disorder, unspecified |
| Drug Abuse | F18.97 | Inhalant use, unspecified with inhalant-induced persisting dementia |
| Drug Abuse | F18.980 | Inhalant use, unspecified with inhalant-induced anxiety disorder |
| Drug Abuse | F18.988 | Inhalant use, unspecified with other inhalant-induced disorder |
| Drug Abuse | F18.99 | Inhalant use, unspecified with unspecified inhalant-induced disorder |
| Drug Abuse | F19.10 | Other psychoactive substance abuse, uncomplicated |
| Drug Abuse | F19.120 | Other psychoactive substance abuse with intoxication, uncomplicated |
| Drug Abuse | F19.121 | Other psychoactive substance abuse with intoxication delirium |
| Drug Abuse | F19.122 | Other psychoactive substance abuse with intoxication with perceptual disturbances |
| Drug Abuse | F19.129 | Other psychoactive substance abuse with intoxication, unspecified |
| Drug Abuse | F19.14 | Other psychoactive substance abuse with psychoactive substance-induced mood disorder |
| Drug Abuse | F19.150 | Other psychoactive substance abuse with psychoactive substance-induced psychotic disorder with delusions |
| Drug Abuse | F19.151 | Other psychoactive substance abuse with psychoactive substance-induced psychotic disorder with hallucinations |
| Drug Abuse | F19.159 | Other psychoactive substance abuse with psychoactive substance-induced psychotic disorder, unspecified |
| Drug Abuse | F19.16 | Other psychoactive substance abuse with psychoactive substance-induced persisting amnestic disorder |
| Drug Abuse | F19.17 | Other psychoactive substance abuse with psychoactive substance-induced persisting dementia |
| Drug Abuse | F19.180 | Other psychoactive substance abuse with psychoactive substance-induced anxiety disorder |
| Drug Abuse | F19.181 | Other psychoactive substance abuse with psychoactive substance-induced sexual dysfunction |
| Drug Abuse | F19.182 | Other psychoactive substance abuse with psychoactive substance-induced sleep disorder |
| Drug Abuse | F19.188 | Other psychoactive substance abuse with other psychoactive substance-induced disorder |
| Drug Abuse | F19.19 | Other psychoactive substance abuse with unspecified psychoactive substance-induced disorder |
| Drug Abuse | F19.20 | Other psychoactive substance dependence, uncomplicated |
| Drug Abuse | F19.21 | Other psychoactive substance dependence, in remission |
| Drug Abuse | F19.220 | Other psychoactive substance dependence with intoxication, uncomplicated |
| Drug Abuse | F19.221 | Other psychoactive substance dependence with intoxication delirium |
| Drug Abuse | F19.222 | Other psychoactive substance dependence with intoxication with perceptual disturbance |
| Drug Abuse | F19.229 | Other psychoactive substance dependence with intoxication, unspecified |
| Drug Abuse | F19.230 | Other psychoactive substance dependence with withdrawal, uncomplicated |
| Drug Abuse | F19.231 | Other psychoactive substance dependence with withdrawal delirium |
| Drug Abuse | F19.232 | Other psychoactive substance dependence with withdrawal with perceptual disturbance |
| Drug Abuse | F19.239 | Other psychoactive substance dependence with withdrawal, unspecified |
| Drug Abuse | F19.24 | Other psychoactive substance dependence with psychoactive substance-induced mood disorder |
| Drug Abuse | F19.250 | Other psychoactive substance dependence with psychoactive substance-induced psychotic disorder with delusions |
| Drug Abuse | F19.251 | Other psychoactive substance dependence with psychoactive substance-induced psychotic disorder with hallucinations |
| Drug Abuse | F19.259 | Other psychoactive substance dependence with psychoactive substance-induced psychotic disorder, unspecified |
| Drug Abuse | F19.26 | Other psychoactive substance dependence with psychoactive substance-induced persisting amnestic disorder |
| Drug Abuse | F19.27 | Other psychoactive substance dependence with psychoactive substance-induced persisting dementia |
| Drug Abuse | F19.280 | Other psychoactive substance dependence with psychoactive substance-induced anxiety disorder |
| Drug Abuse | F19.281 | Other psychoactive substance dependence with psychoactive substance-induced sexual dysfunction |
| Drug Abuse | F19.282 | Other psychoactive substance dependence with psychoactive substance-induced sleep disorder |
| Drug Abuse | F19.288 | Other psychoactive substance dependence with other psychoactive substance-induced disorder |
| Drug Abuse | F19.29 | Other psychoactive substance dependence with unspecified psychoactive substance-induced disorder |
| Drug Abuse | F19.90 | Other psychoactive substance use, unspecified, uncomplicated |
| Drug Abuse | F19.920 | Other psychoactive substance use, unspecified with intoxication, uncomplicated |
| Drug Abuse | F19.921 | Other psychoactive substance use, unspecified with intoxication with delirium |
| Drug Abuse | F19.922 | Other psychoactive substance use, unspecified with intoxication with perceptual disturbance |
| Drug Abuse | F19.929 | Other psychoactive substance use, unspecified with intoxication, unspecified |
| Drug Abuse | F19.930 | Other psychoactive substance use, unspecified with withdrawal, uncomplicated |
| Drug Abuse | F19.931 | Other psychoactive substance use, unspecified with withdrawal delirium |
| Drug Abuse | F19.932 | Other psychoactive substance use, unspecified with withdrawal with perceptual disturbance |
| Drug Abuse | F19.939 | Other psychoactive substance use, unspecified with withdrawal, unspecified |
| Drug Abuse | F19.94 | Other psychoactive substance use, unspecified with psychoactive substance-induced mood disorder |
| Drug Abuse | F19.950 | Other psychoactive substance use, unspecified with psychoactive substance-induced psychotic disorder with delusions |
| Drug Abuse | F19.951 | Other psychoactive substance use, unspecified with psychoactive substance-induced psychotic disorder with hallucinations |
| Drug Abuse | F19.959 | Other psychoactive substance use, unspecified with psychoactive substance-induced psychotic disorder, unspecified |
| Drug Abuse | F19.96 | Other psychoactive substance use, unspecified with psychoactive substance-induced persisting amnestic disorder |
| Drug Abuse | F19.97 | Other psychoactive substance use, unspecified with psychoactive substance-induced persisting dementia |
| Drug Abuse | F19.980 | Other psychoactive substance use, unspecified with psychoactive substance-induced anxiety disorder |
| Drug Abuse | F19.981 | Other psychoactive substance use, unspecified with psychoactive substance-induced sexual dysfunction |
| Drug Abuse | F19.982 | Other psychoactive substance use, unspecified with psychoactive substance-induced sleep disorder |
| Drug Abuse | F19.988 | Other psychoactive substance use, unspecified with other psychoactive substance-induced disorder |
| Drug Abuse | F19.99 | Other psychoactive substance use, unspecified with unspecified psychoactive substance-induced disorder |
| Drug Abuse | Z71.51 | Drug abuse counseling and surveillance of drug abuser |
| Drug Abuse | Z71.52 | Counseling for family member of drug abuser |
| Fluid and Electrolyte Disorders | E22.2 | Syndrome of inappropriate secretion of antidiuretic hormone |
| Fluid and Electrolyte Disorders | E86.0 | Dehydration |
| Fluid and Electrolyte Disorders | E86.1 | Hypovolemia |
| Fluid and Electrolyte Disorders | E86.9 | Volume depletion, unspecified |
| Fluid and Electrolyte Disorders | E87.0 | Hyperosmolality and hypernatremia |
| Fluid and Electrolyte Disorders | E87.1 | Hypo-osmolality and hyponatremia |
| Fluid and Electrolyte Disorders | E87.2 | Acidosis |
| Fluid and Electrolyte Disorders | E87.3 | Alkalosis |
| Fluid and Electrolyte Disorders | E87.4 | Mixed disorder of acid-base balance |
| Fluid and Electrolyte Disorders | E87.5 | Hyperkalemia |
| Fluid and Electrolyte Disorders | E87.6 | Hypokalemia |
| Fluid and Electrolyte Disorders | E87.70 | Fluid overload, unspecified |
| Fluid and Electrolyte Disorders | E87.71 | Transfusion associated circulatory overload |
| Fluid and Electrolyte Disorders | E87.79 | Other fluid overload |
| Fluid and Electrolyte Disorders | E87.8 | Other disorders of electrolyte and fluid balance, not elsewhere classified |
| Hypertension Complicated | I11.0 | Hypertensive heart disease with heart failure |
| Hypertension Complicated | I11.9 | Hypertensive heart disease without heart failure |
| Hypertension Complicated | I12.0 | Hypertensive chronic kidney disease with stage 5 chronic kidney disease or end stage renal disease |
| Hypertension Complicated | I12.9 | Hypertensive chronic kidney disease with stage 1 through stage 4 chronic kidney disease, or unspecified chronic kidney disease |
| Hypertension Complicated | I13.0 | Hypertensive heart and chronic kidney disease with heart failure and stage 1 through stage 4 chronic kidney disease, or unspecified chronic kidney disease |
| Hypertension Complicated | I13.10 | Hypertensive heart and chronic kidney disease without heart failure, with stage 1 through stage 4 chronic kidney disease, or unspecified chronic kidney disease |
| Hypertension Complicated | I13.11 | Hypertensive heart and chronic kidney disease without heart failure, with stage 5 chronic kidney disease, or end stage renal disease |
| Hypertension Complicated | I13.2 | Hypertensive heart and chronic kidney disease with heart failure and with stage 5 chronic kidney disease, or end stage renal disease |
| Hypertension Complicated | I15.0 | Renovascular hypertension |
| Hypertension Complicated | I15.1 | Hypertension secondary to other renal disorders |
| Hypertension Complicated | I15.2 | Hypertension secondary to endocrine disorders |
| Hypertension Complicated | I15.8 | Other secondary hypertension |
| Hypertension Complicated | I15.9 | Secondary hypertension, unspecified |
| Hypertension Uncomplicated | I10 | Essential (primary) hypertension |
| Hypothyroidism | E00.0 | Congenital iodine-deficiency syndrome, neurological type |
| Hypothyroidism | E00.1 | Congenital iodine-deficiency syndrome, myxedematous type |
| Hypothyroidism | E00.2 | Congenital iodine-deficiency syndrome, mixed type |
| Hypothyroidism | E00.9 | Congenital iodine-deficiency syndrome, unspecified |
| Hypothyroidism | E01.0 | Iodine-deficiency related diffuse (endemic) goiter |
| Hypothyroidism | E01.1 | Iodine-deficiency related multinodular (endemic) goiter |
| Hypothyroidism | E01.2 | Iodine-deficiency related (endemic) goiter, unspecified |
| Hypothyroidism | E01.8 | Other iodine-deficiency related thyroid disorders and allied conditions |
| Hypothyroidism | E02 | Subclinical iodine-deficiency hypothyroidism |
| Hypothyroidism | E03.0 | Congenital hypothyroidism with diffuse goiter |
| Hypothyroidism | E03.1 | Congenital hypothyroidism without goiter |
| Hypothyroidism | E03.2 | Hypothyroidism due to medicaments and other exogenous substances |
| Hypothyroidism | E03.3 | Postinfectious hypothyroidism |
| Hypothyroidism | E03.4 | Atrophy of thyroid (acquired) |
| Hypothyroidism | E03.5 | Myxedema coma |
| Hypothyroidism | E03.8 | Other specified hypothyroidism |
| Hypothyroidism | E03.9 | Hypothyroidism, unspecified |
| Hypothyroidism | E89.0 | Postprocedural hypothyroidism |
| Iron Deficiency Anemia | D50.0 | Iron deficiency anemia secondary to blood loss (chronic) |
| Liver Disease | B18.0 | Chronic viral hepatitis B with delta-agent |
| Liver Disease | B18.1 | Chronic viral hepatitis B without delta-agent |
| Liver Disease | B18.2 | Chronic viral hepatitis C |
| Liver Disease | B18.8 | Other chronic viral hepatitis |
| Liver Disease | B18.9 | Chronic viral hepatitis, unspecified |
| Liver Disease | I85.00 | Esophageal varices without bleeding |
| Liver Disease | I85.01 | Esophageal varices with bleeding |
| Liver Disease | I85.10 | Secondary esophageal varices without bleeding |
| Liver Disease | I85.11 | Secondary esophageal varices with bleeding |
| Liver Disease | I86.4 | Gastric varices |
| Liver Disease | K70.0 | Alcoholic fatty liver |
| Liver Disease | K70.10 | Alcoholic hepatitis without ascites |
| Liver Disease | K70.11 | Alcoholic hepatitis with ascites |
| Liver Disease | K70.2 | Alcoholic fibrosis and sclerosis of liver |
| Liver Disease | K70.30 | Alcoholic cirrhosis of liver without ascites |
| Liver Disease | K70.31 | Alcoholic cirrhosis of liver with ascites |
| Liver Disease | K70.40 | Alcoholic hepatic failure without coma |
| Liver Disease | K70.41 | Alcoholic hepatic failure with coma |
| Liver Disease | K70.9 | Alcoholic liver disease, unspecified |
| Liver Disease | K71.10 | Toxic liver disease with hepatic necrosis, without coma |
| Liver Disease | K71.11 | Toxic liver disease with hepatic necrosis, with coma |
| Liver Disease | K71.3 | Toxic liver disease with chronic persistent hepatitis |
| Liver Disease | K71.4 | Toxic liver disease with chronic lobular hepatitis |
| Liver Disease | K71.50 | Toxic liver disease with chronic active hepatitis without ascites |
| Liver Disease | K71.51 | Toxic liver disease with chronic active hepatitis with ascites |
| Liver Disease | K71.7 | Toxic liver disease with fibrosis and cirrhosis of liver |
| Liver Disease | K72.00 | Acute and subacute hepatic failure without coma |
| Liver Disease | K72.01 | Acute and subacute hepatic failure with coma |
| Liver Disease | K72.10 | Chronic hepatic failure without coma |
| Liver Disease | K72.11 | Chronic hepatic failure with coma |
| Liver Disease | K72.90 | Hepatic failure, unspecified without coma |
| Liver Disease | K72.91 | Hepatic failure, unspecified with coma |
| Liver Disease | K73.0 | Chronic persistent hepatitis, not elsewhere classified |
| Liver Disease | K73.1 | Chronic lobular hepatitis, not elsewhere classified |
| Liver Disease | K73.2 | Chronic active hepatitis, not elsewhere classified |
| Liver Disease | K73.8 | Other chronic hepatitis, not elsewhere classified |
| Liver Disease | K73.9 | Chronic hepatitis, unspecified |
| Liver Disease | K74.0 | Hepatic fibrosis |
| Liver Disease | K74.1 | Hepatic sclerosis |
| Liver Disease | K74.2 | Hepatic fibrosis with hepatic sclerosis |
| Liver Disease | K74.3 | Primary biliary cirrhosis |
| Liver Disease | K74.4 | Secondary biliary cirrhosis |
| Liver Disease | K74.5 | Biliary cirrhosis, unspecified |
| Liver Disease | K74.60 | Unspecified cirrhosis of liver |
| Liver Disease | K74.69 | Other cirrhosis of liver |
| Liver Disease | K76.0 | Fatty (change of) liver, not elsewhere classified |
| Liver Disease | K76.2 | Central hemorrhagic necrosis of liver |
| Liver Disease | K76.3 | Infarction of liver |
| Liver Disease | K76.4 | Peliosis hepatis |
| Liver Disease | K76.5 | Hepatic veno-occlusive disease |
| Liver Disease | K76.6 | Portal hypertension |
| Liver Disease | K76.7 | Hepatorenal syndrome |
| Liver Disease | K76.81 | Hepatopulmonary syndrome |
| Liver Disease | K76.89 | Other specified diseases of liver |
| Liver Disease | K76.9 | Liver disease, unspecified |
| Liver Disease | Z94.4 | Liver transplant status |
| Lymphoma | C81.00 | Nodular lymphocyte predominant Hodgkin lymphoma, unspecified site |
| Lymphoma | C81.01 | Nodular lymphocyte predominant Hodgkin lymphoma, lymph nodes of head, face, and neck |
| Lymphoma | C81.02 | Nodular lymphocyte predominant Hodgkin lymphoma, intrathoracic lymph nodes |
| Lymphoma | C81.03 | Nodular lymphocyte predominant Hodgkin lymphoma, intra-abdominal lymph nodes |
| Lymphoma | C81.04 | Nodular lymphocyte predominant Hodgkin lymphoma, lymph nodes of axilla and upper limb |
| Lymphoma | C81.05 | Nodular lymphocyte predominant Hodgkin lymphoma, lymph nodes of inguinal region and lower limb |
| Lymphoma | C81.06 | Nodular lymphocyte predominant Hodgkin lymphoma, intrapelvic lymph nodes |
| Lymphoma | C81.07 | Nodular lymphocyte predominant Hodgkin lymphoma, spleen |
| Lymphoma | C81.08 | Nodular lymphocyte predominant Hodgkin lymphoma, lymph nodes of multiple sites |
| Lymphoma | C81.09 | Nodular lymphocyte predominant Hodgkin lymphoma, extranodal and solid organ sites |
| Lymphoma | C81.10 | Nodular sclerosis Hodgkin lymphoma, unspecified site |
| Lymphoma | C81.11 | Nodular sclerosis Hodgkin lymphoma, lymph nodes of head, face, and neck |
| Lymphoma | C81.12 | Nodular sclerosis Hodgkin lymphoma, intrathoracic lymph nodes |
| Lymphoma | C81.13 | Nodular sclerosis Hodgkin lymphoma, intra-abdominal lymph nodes |
| Lymphoma | C81.14 | Nodular sclerosis Hodgkin lymphoma, lymph nodes of axilla and upper limb |
| Lymphoma | C81.15 | Nodular sclerosis Hodgkin lymphoma, lymph nodes of inguinal region and lower limb |
| Lymphoma | C81.16 | Nodular sclerosis Hodgkin lymphoma, intrapelvic lymph nodes |
| Lymphoma | C81.17 | Nodular sclerosis Hodgkin lymphoma, spleen |
| Lymphoma | C81.18 | Nodular sclerosis Hodgkin lymphoma, lymph nodes of multiple sites |
| Lymphoma | C81.19 | Nodular sclerosis Hodgkin lymphoma, extranodal and solid organ sites |
| Lymphoma | C81.20 | Mixed cellularity Hodgkin lymphoma, unspecified site |
| Lymphoma | C81.21 | Mixed cellularity Hodgkin lymphoma, lymph nodes of head, face, and neck |
| Lymphoma | C81.22 | Mixed cellularity Hodgkin lymphoma, intrathoracic lymph nodes |
| Lymphoma | C81.23 | Mixed cellularity Hodgkin lymphoma, intra-abdominal lymph nodes |
| Lymphoma | C81.24 | Mixed cellularity Hodgkin lymphoma, lymph nodes of axilla and upper limb |
| Lymphoma | C81.25 | Mixed cellularity Hodgkin lymphoma, lymph nodes of inguinal region and lower limb |
| Lymphoma | C81.26 | Mixed cellularity Hodgkin lymphoma, intrapelvic lymph nodes |
| Lymphoma | C81.27 | Mixed cellularity Hodgkin lymphoma, spleen |
| Lymphoma | C81.28 | Mixed cellularity Hodgkin lymphoma, lymph nodes of multiple sites |
| Lymphoma | C81.29 | Mixed cellularity Hodgkin lymphoma, extranodal and solid organ sites |
| Lymphoma | C81.30 | Lymphocyte depleted Hodgkin lymphoma, unspecified site |
| Lymphoma | C81.31 | Lymphocyte depleted Hodgkin lymphoma, lymph nodes of head, face, and neck |
| Lymphoma | C81.32 | Lymphocyte depleted Hodgkin lymphoma, intrathoracic lymph nodes |
| Lymphoma | C81.33 | Lymphocyte depleted Hodgkin lymphoma, intra-abdominal lymph nodes |
| Lymphoma | C81.34 | Lymphocyte depleted Hodgkin lymphoma, lymph nodes of axilla and upper limb |
| Lymphoma | C81.35 | Lymphocyte depleted Hodgkin lymphoma, lymph nodes of inguinal region and lower limb |
| Lymphoma | C81.36 | Lymphocyte depleted Hodgkin lymphoma, intrapelvic lymph nodes |
| Lymphoma | C81.37 | Lymphocyte depleted Hodgkin lymphoma, spleen |
| Lymphoma | C81.38 | Lymphocyte depleted Hodgkin lymphoma, lymph nodes of multiple sites |
| Lymphoma | C81.39 | Lymphocyte depleted Hodgkin lymphoma, extranodal and solid organ sites |
| Lymphoma | C81.40 | Lymphocyte-rich Hodgkin lymphoma, unspecified site |
| Lymphoma | C81.41 | Lymphocyte-rich Hodgkin lymphoma, lymph nodes of head, face, and neck |
| Lymphoma | C81.42 | Lymphocyte-rich Hodgkin lymphoma, intrathoracic lymph nodes |
| Lymphoma | C81.43 | Lymphocyte-rich Hodgkin lymphoma, intra-abdominal lymph nodes |
| Lymphoma | C81.44 | Lymphocyte-rich Hodgkin lymphoma, lymph nodes of axilla and upper limb |
| Lymphoma | C81.45 | Lymphocyte-rich Hodgkin lymphoma, lymph nodes of inguinal region and lower limb |
| Lymphoma | C81.46 | Lymphocyte-rich Hodgkin lymphoma, intrapelvic lymph nodes |
| Lymphoma | C81.47 | Lymphocyte-rich Hodgkin lymphoma, spleen |
| Lymphoma | C81.48 | Lymphocyte-rich Hodgkin lymphoma, lymph nodes of multiple sites |
| Lymphoma | C81.49 | Lymphocyte-rich Hodgkin lymphoma, extranodal and solid organ sites |
| Lymphoma | C81.70 | Other Hodgkin lymphoma, unspecified site |
| Lymphoma | C81.71 | Other Hodgkin lymphoma, lymph nodes of head, face, and neck |
| Lymphoma | C81.72 | Other Hodgkin lymphoma, intrathoracic lymph nodes |
| Lymphoma | C81.73 | Other Hodgkin lymphoma, intra-abdominal lymph nodes |
| Lymphoma | C81.74 | Other Hodgkin lymphoma, lymph nodes of axilla and upper limb |
| Lymphoma | C81.75 | Other Hodgkin lymphoma, lymph nodes of inguinal region and lower limb |
| Lymphoma | C81.76 | Other Hodgkin lymphoma, intrapelvic lymph nodes |
| Lymphoma | C81.77 | Other Hodgkin lymphoma, spleen |
| Lymphoma | C81.78 | Other Hodgkin lymphoma, lymph nodes of multiple sites |
| Lymphoma | C81.79 | Other Hodgkin lymphoma, extranodal and solid organ sites |
| Lymphoma | C81.90 | Hodgkin lymphoma, unspecified, unspecified site |
| Lymphoma | C81.91 | Hodgkin lymphoma, unspecified, lymph nodes of head, face, and neck |
| Lymphoma | C81.92 | Hodgkin lymphoma, unspecified, intrathoracic lymph nodes |
| Lymphoma | C81.93 | Hodgkin lymphoma, unspecified, intra-abdominal lymph nodes |
| Lymphoma | C81.94 | Hodgkin lymphoma, unspecified, lymph nodes of axilla and upper limb |
| Lymphoma | C81.95 | Hodgkin lymphoma, unspecified, lymph nodes of inguinal region and lower limb |
| Lymphoma | C81.96 | Hodgkin lymphoma, unspecified, intrapelvic lymph nodes |
| Lymphoma | C81.97 | Hodgkin lymphoma, unspecified, spleen |
| Lymphoma | C81.98 | Hodgkin lymphoma, unspecified, lymph nodes of multiple sites |
| Lymphoma | C81.99 | Hodgkin lymphoma, unspecified, extranodal and solid organ sites |
| Lymphoma | C82.00 | Follicular lymphoma grade I, unspecified site |
| Lymphoma | C82.01 | Follicular lymphoma grade I, lymph nodes of head, face, and neck |
| Lymphoma | C82.02 | Follicular lymphoma grade I, intrathoracic lymph nodes |
| Lymphoma | C82.03 | Follicular lymphoma grade I, intra-abdominal lymph nodes |
| Lymphoma | C82.04 | Follicular lymphoma grade I, lymph nodes of axilla and upper limb |
| Lymphoma | C82.05 | Follicular lymphoma grade I, lymph nodes of inguinal region and lower limb |
| Lymphoma | C82.06 | Follicular lymphoma grade I, intrapelvic lymph nodes |
| Lymphoma | C82.07 | Follicular lymphoma grade I, spleen |
| Lymphoma | C82.08 | Follicular lymphoma grade I, lymph nodes of multiple sites |
| Lymphoma | C82.09 | Follicular lymphoma grade I, extranodal and solid organ sites |
| Lymphoma | C82.10 | Follicular lymphoma grade II, unspecified site |
| Lymphoma | C82.11 | Follicular lymphoma grade II, lymph nodes of head, face, and neck |
| Lymphoma | C82.12 | Follicular lymphoma grade II, intrathoracic lymph nodes |
| Lymphoma | C82.13 | Follicular lymphoma grade II, intra-abdominal lymph nodes |
| Lymphoma | C82.14 | Follicular lymphoma grade II, lymph nodes of axilla and upper limb |
| Lymphoma | C82.15 | Follicular lymphoma grade II, lymph nodes of inguinal region and lower limb |
| Lymphoma | C82.16 | Follicular lymphoma grade II, intrapelvic lymph nodes |
| Lymphoma | C82.17 | Follicular lymphoma grade II, spleen |
| Lymphoma | C82.18 | Follicular lymphoma grade II, lymph nodes of multiple sites |
| Lymphoma | C82.19 | Follicular lymphoma grade II, extranodal and solid organ sites |
| Lymphoma | C82.20 | Follicular lymphoma grade III, unspecified, unspecified site |
| Lymphoma | C82.21 | Follicular lymphoma grade III, unspecified, lymph nodes of head, face, and neck |
| Lymphoma | C82.22 | Follicular lymphoma grade III, unspecified, intrathoracic lymph nodes |
| Lymphoma | C82.23 | Follicular lymphoma grade III, unspecified, intra-abdominal lymph nodes |
| Lymphoma | C82.24 | Follicular lymphoma grade III, unspecified, lymph nodes of axilla and upper limb |
| Lymphoma | C82.25 | Follicular lymphoma grade III, unspecified, lymph nodes of inguinal region and lower limb |
| Lymphoma | C82.26 | Follicular lymphoma grade III, unspecified, intrapelvic lymph nodes |
| Lymphoma | C82.27 | Follicular lymphoma grade III, unspecified, spleen |
| Lymphoma | C82.28 | Follicular lymphoma grade III, unspecified, lymph nodes of multiple sites |
| Lymphoma | C82.29 | Follicular lymphoma grade III, unspecified, extranodal and solid organ sites |
| Lymphoma | C82.30 | Follicular lymphoma grade IIIa, unspecified site |
| Lymphoma | C82.31 | Follicular lymphoma grade IIIa, lymph nodes of head, face, and neck |
| Lymphoma | C82.32 | Follicular lymphoma grade IIIa, intrathoracic lymph nodes |
| Lymphoma | C82.33 | Follicular lymphoma grade IIIa, intra-abdominal lymph nodes |
| Lymphoma | C82.34 | Follicular lymphoma grade IIIa, lymph nodes of axilla and upper limb |
| Lymphoma | C82.35 | Follicular lymphoma grade IIIa, lymph nodes of inguinal region and lower limb |
| Lymphoma | C82.36 | Follicular lymphoma grade IIIa, intrapelvic lymph nodes |
| Lymphoma | C82.37 | Follicular lymphoma grade IIIa, spleen |
| Lymphoma | C82.38 | Follicular lymphoma grade IIIa, lymph nodes of multiple sites |
| Lymphoma | C82.39 | Follicular lymphoma grade IIIa, extranodal and solid organ sites |
| Lymphoma | C82.40 | Follicular lymphoma grade IIIb, unspecified site |
| Lymphoma | C82.41 | Follicular lymphoma grade IIIb, lymph nodes of head, face, and neck |
| Lymphoma | C82.42 | Follicular lymphoma grade IIIb, intrathoracic lymph nodes |
| Lymphoma | C82.43 | Follicular lymphoma grade IIIb, intra-abdominal lymph nodes |
| Lymphoma | C82.44 | Follicular lymphoma grade IIIb, lymph nodes of axilla and upper limb |
| Lymphoma | C82.45 | Follicular lymphoma grade IIIb, lymph nodes of inguinal region and lower limb |
| Lymphoma | C82.46 | Follicular lymphoma grade IIIb, intrapelvic lymph nodes |
| Lymphoma | C82.47 | Follicular lymphoma grade IIIb, spleen |
| Lymphoma | C82.48 | Follicular lymphoma grade IIIb, lymph nodes of multiple sites |
| Lymphoma | C82.49 | Follicular lymphoma grade IIIb, extranodal and solid organ sites |
| Lymphoma | C82.50 | Diffuse follicle center lymphoma, unspecified site |
| Lymphoma | C82.51 | Diffuse follicle center lymphoma, lymph nodes of head, face, and neck |
| Lymphoma | C82.52 | Diffuse follicle center lymphoma, intrathoracic lymph nodes |
| Lymphoma | C82.53 | Diffuse follicle center lymphoma, intra-abdominal lymph nodes |
| Lymphoma | C82.54 | Diffuse follicle center lymphoma, lymph nodes of axilla and upper limb |
| Lymphoma | C82.55 | Diffuse follicle center lymphoma, lymph nodes of inguinal region and lower limb |
| Lymphoma | C82.56 | Diffuse follicle center lymphoma, intrapelvic lymph nodes |
| Lymphoma | C82.57 | Diffuse follicle center lymphoma, spleen |
| Lymphoma | C82.58 | Diffuse follicle center lymphoma, lymph nodes of multiple sites |
| Lymphoma | C82.59 | Diffuse follicle center lymphoma, extranodal and solid organ sites |
| Lymphoma | C82.60 | Cutaneous follicle center lymphoma, unspecified site |
| Lymphoma | C82.61 | Cutaneous follicle center lymphoma, lymph nodes of head, face, and neck |
| Lymphoma | C82.62 | Cutaneous follicle center lymphoma, intrathoracic lymph nodes |
| Lymphoma | C82.63 | Cutaneous follicle center lymphoma, intra-abdominal lymph nodes |
| Lymphoma | C82.64 | Cutaneous follicle center lymphoma, lymph nodes of axilla and upper limb |
| Lymphoma | C82.65 | Cutaneous follicle center lymphoma, lymph nodes of inguinal region and lower limb |
| Lymphoma | C82.66 | Cutaneous follicle center lymphoma, intrapelvic lymph nodes |
| Lymphoma | C82.67 | Cutaneous follicle center lymphoma, spleen |
| Lymphoma | C82.68 | Cutaneous follicle center lymphoma, lymph nodes of multiple sites |
| Lymphoma | C82.69 | Cutaneous follicle center lymphoma, extranodal and solid organ sites |
| Lymphoma | C82.80 | Other types of follicular lymphoma, unspecified site |
| Lymphoma | C82.81 | Other types of follicular lymphoma, lymph nodes of head, face, and neck |
| Lymphoma | C82.82 | Other types of follicular lymphoma, intrathoracic lymph nodes |
| Lymphoma | C82.83 | Other types of follicular lymphoma, intra-abdominal lymph nodes |
| Lymphoma | C82.84 | Other types of follicular lymphoma, lymph nodes of axilla and upper limb |
| Lymphoma | C82.85 | Other types of follicular lymphoma, lymph nodes of inguinal region and lower limb |
| Lymphoma | C82.86 | Other types of follicular lymphoma, intrapelvic lymph nodes |
| Lymphoma | C82.87 | Other types of follicular lymphoma, spleen |
| Lymphoma | C82.88 | Other types of follicular lymphoma, lymph nodes of multiple sites |
| Lymphoma | C82.89 | Other types of follicular lymphoma, extranodal and solid organ sites |
| Lymphoma | C82.90 | Follicular lymphoma, unspecified, unspecified site |
| Lymphoma | C82.91 | Follicular lymphoma, unspecified, lymph nodes of head, face, and neck |
| Lymphoma | C82.92 | Follicular lymphoma, unspecified, intrathoracic lymph nodes |
| Lymphoma | C82.93 | Follicular lymphoma, unspecified, intra-abdominal lymph nodes |
| Lymphoma | C82.94 | Follicular lymphoma, unspecified, lymph nodes of axilla and upper limb |
| Lymphoma | C82.95 | Follicular lymphoma, unspecified, lymph nodes of inguinal region and lower limb |
| Lymphoma | C82.96 | Follicular lymphoma, unspecified, intrapelvic lymph nodes |
| Lymphoma | C82.97 | Follicular lymphoma, unspecified, spleen |
| Lymphoma | C82.98 | Follicular lymphoma, unspecified, lymph nodes of multiple sites |
| Lymphoma | C82.99 | Follicular lymphoma, unspecified, extranodal and solid organ sites |
| Lymphoma | C83.00 | Small cell B-cell lymphoma, unspecified site |
| Lymphoma | C83.01 | Small cell B-cell lymphoma, lymph nodes of head, face, and neck |
| Lymphoma | C83.02 | Small cell B-cell lymphoma, intrathoracic lymph nodes |
| Lymphoma | C83.03 | Small cell B-cell lymphoma, intra-abdominal lymph nodes |
| Lymphoma | C83.04 | Small cell B-cell lymphoma, lymph nodes of axilla and upper limb |
| Lymphoma | C83.05 | Small cell B-cell lymphoma, lymph nodes of inguinal region and lower limb |
| Lymphoma | C83.06 | Small cell B-cell lymphoma, intrapelvic lymph nodes |
| Lymphoma | C83.07 | Small cell B-cell lymphoma, spleen |
| Lymphoma | C83.08 | Small cell B-cell lymphoma, lymph nodes of multiple sites |
| Lymphoma | C83.09 | Small cell B-cell lymphoma, extranodal and solid organ sites |
| Lymphoma | C83.10 | Mantle cell lymphoma, unspecified site |
| Lymphoma | C83.11 | Mantle cell lymphoma, lymph nodes of head, face, and neck |
| Lymphoma | C83.12 | Mantle cell lymphoma, intrathoracic lymph nodes |
| Lymphoma | C83.13 | Mantle cell lymphoma, intra-abdominal lymph nodes |
| Lymphoma | C83.14 | Mantle cell lymphoma, lymph nodes of axilla and upper limb |
| Lymphoma | C83.15 | Mantle cell lymphoma, lymph nodes of inguinal region and lower limb |
| Lymphoma | C83.16 | Mantle cell lymphoma, intrapelvic lymph nodes |
| Lymphoma | C83.17 | Mantle cell lymphoma, spleen |
| Lymphoma | C83.18 | Mantle cell lymphoma, lymph nodes of multiple sites |
| Lymphoma | C83.19 | Mantle cell lymphoma, extranodal and solid organ sites |
| Lymphoma | C83.30 | Diffuse large B-cell lymphoma, unspecified site |
| Lymphoma | C83.31 | Diffuse large B-cell lymphoma, lymph nodes of head, face, and neck |
| Lymphoma | C83.32 | Diffuse large B-cell lymphoma, intrathoracic lymph nodes |
| Lymphoma | C83.33 | Diffuse large B-cell lymphoma, intra-abdominal lymph nodes |
| Lymphoma | C83.34 | Diffuse large B-cell lymphoma, lymph nodes of axilla and upper limb |
| Lymphoma | C83.35 | Diffuse large B-cell lymphoma, lymph nodes of inguinal region and lower limb |
| Lymphoma | C83.36 | Diffuse large B-cell lymphoma, intrapelvic lymph nodes |
| Lymphoma | C83.37 | Diffuse large B-cell lymphoma, spleen |
| Lymphoma | C83.38 | Diffuse large B-cell lymphoma, lymph nodes of multiple sites |
| Lymphoma | C83.39 | Diffuse large B-cell lymphoma, extranodal and solid organ sites |
| Lymphoma | C83.50 | Lymphoblastic (diffuse) lymphoma, unspecified site |
| Lymphoma | C83.51 | Lymphoblastic (diffuse) lymphoma, lymph nodes of head, face, and neck |
| Lymphoma | C83.52 | Lymphoblastic (diffuse) lymphoma, intrathoracic lymph nodes |
| Lymphoma | C83.53 | Lymphoblastic (diffuse) lymphoma, intra-abdominal lymph nodes |
| Lymphoma | C83.54 | Lymphoblastic (diffuse) lymphoma, lymph nodes of axilla and upper limb |
| Lymphoma | C83.55 | Lymphoblastic (diffuse) lymphoma, lymph nodes of inguinal region and lower limb |
| Lymphoma | C83.56 | Lymphoblastic (diffuse) lymphoma, intrapelvic lymph nodes |
| Lymphoma | C83.57 | Lymphoblastic (diffuse) lymphoma, spleen |
| Lymphoma | C83.58 | Lymphoblastic (diffuse) lymphoma, lymph nodes of multiple sites |
| Lymphoma | C83.59 | Lymphoblastic (diffuse) lymphoma, extranodal and solid organ sites |
| Lymphoma | C83.70 | Burkitt lymphoma, unspecified site |
| Lymphoma | C83.71 | Burkitt lymphoma, lymph nodes of head, face, and neck |
| Lymphoma | C83.72 | Burkitt lymphoma, intrathoracic lymph nodes |
| Lymphoma | C83.73 | Burkitt lymphoma, intra-abdominal lymph nodes |
| Lymphoma | C83.74 | Burkitt lymphoma, lymph nodes of axilla and upper limb |
| Lymphoma | C83.75 | Burkitt lymphoma, lymph nodes of inguinal region and lower limb |
| Lymphoma | C83.76 | Burkitt lymphoma, intrapelvic lymph nodes |
| Lymphoma | C83.77 | Burkitt lymphoma, spleen |
| Lymphoma | C83.78 | Burkitt lymphoma, lymph nodes of multiple sites |
| Lymphoma | C83.79 | Burkitt lymphoma, extranodal and solid organ sites |
| Lymphoma | C83.80 | Other non-follicular lymphoma, unspecified site |
| Lymphoma | C83.81 | Other non-follicular lymphoma, lymph nodes of head, face, and neck |
| Lymphoma | C83.82 | Other non-follicular lymphoma, intrathoracic lymph nodes |
| Lymphoma | C83.83 | Other non-follicular lymphoma, intra-abdominal lymph nodes |
| Lymphoma | C83.84 | Other non-follicular lymphoma, lymph nodes of axilla and upper limb |
| Lymphoma | C83.85 | Other non-follicular lymphoma, lymph nodes of inguinal region and lower limb |
| Lymphoma | C83.86 | Other non-follicular lymphoma, intrapelvic lymph nodes |
| Lymphoma | C83.87 | Other non-follicular lymphoma, spleen |
| Lymphoma | C83.88 | Other non-follicular lymphoma, lymph nodes of multiple sites |
| Lymphoma | C83.89 | Other non-follicular lymphoma, extranodal and solid organ sites |
| Lymphoma | C83.90 | Non-follicular (diffuse) lymphoma, unspecified, unspecified site |
| Lymphoma | C83.91 | Non-follicular (diffuse) lymphoma, unspecified, lymph nodes of head, face, and neck |
| Lymphoma | C83.92 | Non-follicular (diffuse) lymphoma, unspecified, intrathoracic lymph nodes |
| Lymphoma | C83.93 | Non-follicular (diffuse) lymphoma, unspecified, intra-abdominal lymph nodes |
| Lymphoma | C83.94 | Non-follicular (diffuse) lymphoma, unspecified, lymph nodes of axilla and upper limb |
| Lymphoma | C83.95 | Non-follicular (diffuse) lymphoma, unspecified, lymph nodes of inguinal region and lower limb |
| Lymphoma | C83.96 | Non-follicular (diffuse) lymphoma, unspecified, intrapelvic lymph nodes |
| Lymphoma | C83.97 | Non-follicular (diffuse) lymphoma, unspecified, spleen |
| Lymphoma | C83.98 | Non-follicular (diffuse) lymphoma, unspecified, lymph nodes of multiple sites |
| Lymphoma | C83.99 | Non-follicular (diffuse) lymphoma, unspecified, extranodal and solid organ sites |
| Lymphoma | C84.00 | Mycosis fungoides, unspecified site |
| Lymphoma | C84.01 | Mycosis fungoides, lymph nodes of head, face, and neck |
| Lymphoma | C84.02 | Mycosis fungoides, intrathoracic lymph nodes |
| Lymphoma | C84.03 | Mycosis fungoides, intra-abdominal lymph nodes |
| Lymphoma | C84.04 | Mycosis fungoides, lymph nodes of axilla and upper limb |
| Lymphoma | C84.05 | Mycosis fungoides, lymph nodes of inguinal region and lower limb |
| Lymphoma | C84.06 | Mycosis fungoides, intrapelvic lymph nodes |
| Lymphoma | C84.07 | Mycosis fungoides, spleen |
| Lymphoma | C84.08 | Mycosis fungoides, lymph nodes of multiple sites |
| Lymphoma | C84.09 | Mycosis fungoides, extranodal and solid organ sites |
| Lymphoma | C84.10 | Sézary disease, unspecified site |
| Lymphoma | C84.11 | Sézary disease, lymph nodes of head, face, and neck |
| Lymphoma | C84.12 | Sézary disease, intrathoracic lymph nodes |
| Lymphoma | C84.13 | Sézary disease, intra-abdominal lymph nodes |
| Lymphoma | C84.14 | Sézary disease, lymph nodes of axilla and upper limb |
| Lymphoma | C84.15 | Sézary disease, lymph nodes of inguinal region and lower limb |
| Lymphoma | C84.16 | Sézary disease, intrapelvic lymph nodes |
| Lymphoma | C84.17 | Sézary disease, spleen |
| Lymphoma | C84.18 | Sézary disease, lymph nodes of multiple sites |
| Lymphoma | C84.19 | Sézary disease, extranodal and solid organ sites |
| Lymphoma | C84.40 | Peripheral T-cell lymphoma, not elsewhere classified, unspecified site |
| Lymphoma | C84.41 | Peripheral T-cell lymphoma, not elsewhere classified, lymph nodes of head, face, and neck |
| Lymphoma | C84.42 | Peripheral T-cell lymphoma, not elsewhere classified, intrathoracic lymph nodes |
| Lymphoma | C84.43 | Peripheral T-cell lymphoma, not elsewhere classified, intra-abdominal lymph nodes |
| Lymphoma | C84.44 | Peripheral T-cell lymphoma, not elsewhere classified, lymph nodes of axilla and upper limb |
| Lymphoma | C84.45 | Peripheral T-cell lymphoma, not elsewhere classified, lymph nodes of inguinal region and lower limb |
| Lymphoma | C84.46 | Peripheral T-cell lymphoma, not elsewhere classified, intrapelvic lymph nodes |
| Lymphoma | C84.47 | Peripheral T-cell lymphoma, not elsewhere classified, spleen |
| Lymphoma | C84.48 | Peripheral T-cell lymphoma, not elsewhere classified, lymph nodes of multiple sites |
| Lymphoma | C84.49 | Peripheral T-cell lymphoma, not elsewhere classified, extranodal and solid organ sites |
| Lymphoma | C84.60 | Anaplastic large cell lymphoma, ALK-positive, unspecified site |
| Lymphoma | C84.61 | Anaplastic large cell lymphoma, ALK-positive, lymph nodes of head, face, and neck |
| Lymphoma | C84.62 | Anaplastic large cell lymphoma, ALK-positive, intrathoracic lymph nodes |
| Lymphoma | C84.63 | Anaplastic large cell lymphoma, ALK-positive, intra-abdominal lymph nodes |
| Lymphoma | C84.64 | Anaplastic large cell lymphoma, ALK-positive, lymph nodes of axilla and upper limb |
| Lymphoma | C84.65 | Anaplastic large cell lymphoma, ALK-positive, lymph nodes of inguinal region and lower limb |
| Lymphoma | C84.66 | Anaplastic large cell lymphoma, ALK-positive, intrapelvic lymph nodes |
| Lymphoma | C84.67 | Anaplastic large cell lymphoma, ALK-positive, spleen |
| Lymphoma | C84.68 | Anaplastic large cell lymphoma, ALK-positive, lymph nodes of multiple sites |
| Lymphoma | C84.69 | Anaplastic large cell lymphoma, ALK-positive, extranodal and solid organ sites |
| Lymphoma | C84.70 | Anaplastic large cell lymphoma, ALK-negative, unspecified site |
| Lymphoma | C84.71 | Anaplastic large cell lymphoma, ALK-negative, lymph nodes of head, face, and neck |
| Lymphoma | C84.72 | Anaplastic large cell lymphoma, ALK-negative, intrathoracic lymph nodes |
| Lymphoma | C84.73 | Anaplastic large cell lymphoma, ALK-negative, intra-abdominal lymph nodes |
| Lymphoma | C84.74 | Anaplastic large cell lymphoma, ALK-negative, lymph nodes of axilla and upper limb |
| Lymphoma | C84.75 | Anaplastic large cell lymphoma, ALK-negative, lymph nodes of inguinal region and lower limb |
| Lymphoma | C84.76 | Anaplastic large cell lymphoma, ALK-negative, intrapelvic lymph nodes |
| Lymphoma | C84.77 | Anaplastic large cell lymphoma, ALK-negative, spleen |
| Lymphoma | C84.78 | Anaplastic large cell lymphoma, ALK-negative, lymph nodes of multiple sites |
| Lymphoma | C84.79 | Anaplastic large cell lymphoma, ALK-negative, extranodal and solid organ sites |
| Lymphoma | C84.90 | Mature T/NK-cell lymphomas, unspecified, unspecified site |
| Lymphoma | C84.91 | Mature T/NK-cell lymphomas, unspecified, lymph nodes of head, face, and neck |
| Lymphoma | C84.92 | Mature T/NK-cell lymphomas, unspecified, intrathoracic lymph nodes |
| Lymphoma | C84.93 | Mature T/NK-cell lymphomas, unspecified, intra-abdominal lymph nodes |
| Lymphoma | C84.94 | Mature T/NK-cell lymphomas, unspecified, lymph nodes of axilla and upper limb |
| Lymphoma | C84.95 | Mature T/NK-cell lymphomas, unspecified, lymph nodes of inguinal region and lower limb |
| Lymphoma | C84.96 | Mature T/NK-cell lymphomas, unspecified, intrapelvic lymph nodes |
| Lymphoma | C84.97 | Mature T/NK-cell lymphomas, unspecified, spleen |
| Lymphoma | C84.98 | Mature T/NK-cell lymphomas, unspecified, lymph nodes of multiple sites |
| Lymphoma | C84.99 | Mature T/NK-cell lymphomas, unspecified, extranodal and solid organ sites |
| Lymphoma | C84.A0 | Cutaneous T-cell lymphoma, unspecified, unspecified site |
| Lymphoma | C84.A1 | Cutaneous T-cell lymphoma, unspecified lymph nodes of head, face, and neck |
| Lymphoma | C84.A2 | Cutaneous T-cell lymphoma, unspecified, intrathoracic lymph nodes |
| Lymphoma | C84.A3 | Cutaneous T-cell lymphoma, unspecified, intra-abdominal lymph nodes |
| Lymphoma | C84.A4 | Cutaneous T-cell lymphoma, unspecified, lymph nodes of axilla and upper limb |
| Lymphoma | C84.A5 | Cutaneous T-cell lymphoma, unspecified, lymph nodes of inguinal region and lower limb |
| Lymphoma | C84.A6 | Cutaneous T-cell lymphoma, unspecified, intrapelvic lymph nodes |
| Lymphoma | C84.A7 | Cutaneous T-cell lymphoma, unspecified, spleen |
| Lymphoma | C84.A8 | Cutaneous T-cell lymphoma, unspecified, lymph nodes of multiple sites |
| Lymphoma | C84.A9 | Cutaneous T-cell lymphoma, unspecified, extranodal and solid organ sites |
| Lymphoma | C84.Z0 | Other mature T/NK-cell lymphomas, unspecified site |
| Lymphoma | C84.Z1 | Other mature T/NK-cell lymphomas, lymph nodes of head, face, and neck |
| Lymphoma | C84.Z2 | Other mature T/NK-cell lymphomas, intrathoracic lymph nodes |
| Lymphoma | C84.Z3 | Other mature T/NK-cell lymphomas, intra-abdominal lymph nodes |
| Lymphoma | C84.Z4 | Other mature T/NK-cell lymphomas, lymph nodes of axilla and upper limb |
| Lymphoma | C84.Z5 | Other mature T/NK-cell lymphomas, lymph nodes of inguinal region and lower limb |
| Lymphoma | C84.Z6 | Other mature T/NK-cell lymphomas, intrapelvic lymph nodes |
| Lymphoma | C84.Z7 | Other mature T/NK-cell lymphomas, spleen |
| Lymphoma | C84.Z8 | Other mature T/NK-cell lymphomas, lymph nodes of multiple sites |
| Lymphoma | C84.Z9 | Other mature T/NK-cell lymphomas, extranodal and solid organ sites |
| Lymphoma | C85.10 | Unspecified B-cell lymphoma, unspecified site |
| Lymphoma | C85.11 | Unspecified B-cell lymphoma, lymph nodes of head, face, and neck |
| Lymphoma | C85.12 | Unspecified B-cell lymphoma, intrathoracic lymph nodes |
| Lymphoma | C85.13 | Unspecified B-cell lymphoma, intra-abdominal lymph nodes |
| Lymphoma | C85.14 | Unspecified B-cell lymphoma, lymph nodes of axilla and upper limb |
| Lymphoma | C85.15 | Unspecified B-cell lymphoma, lymph nodes of inguinal region and lower limb |
| Lymphoma | C85.16 | Unspecified B-cell lymphoma, intrapelvic lymph nodes |
| Lymphoma | C85.17 | Unspecified B-cell lymphoma, spleen |
| Lymphoma | C85.18 | Unspecified B-cell lymphoma, lymph nodes of multiple sites |
| Lymphoma | C85.19 | Unspecified B-cell lymphoma, extranodal and solid organ sites |
| Lymphoma | C85.20 | Mediastinal (thymic) large B-cell lymphoma, unspecified site |
| Lymphoma | C85.21 | Mediastinal (thymic) large B-cell lymphoma, lymph nodes of head, face, and neck |
| Lymphoma | C85.22 | Mediastinal (thymic) large B-cell lymphoma, intrathoracic lymph nodes |
| Lymphoma | C85.23 | Mediastinal (thymic) large B-cell lymphoma, intra-abdominal lymph nodes |
| Lymphoma | C85.24 | Mediastinal (thymic) large B-cell lymphoma, lymph nodes of axilla and upper limb |
| Lymphoma | C85.25 | Mediastinal (thymic) large B-cell lymphoma, lymph nodes of inguinal region and lower limb |
| Lymphoma | C85.26 | Mediastinal (thymic) large B-cell lymphoma, intrapelvic lymph nodes |
| Lymphoma | C85.27 | Mediastinal (thymic) large B-cell lymphoma, spleen |
| Lymphoma | C85.28 | Mediastinal (thymic) large B-cell lymphoma, lymph nodes of multiple sites |
| Lymphoma | C85.29 | Mediastinal (thymic) large B-cell lymphoma, extranodal and solid organ sites |
| Lymphoma | C85.80 | Other specified types of non-Hodgkin lymphoma, unspecified site |
| Lymphoma | C85.81 | Other specified types of non-Hodgkin lymphoma, lymph nodes of head, face, and neck |
| Lymphoma | C85.82 | Other specified types of non-Hodgkin lymphoma, intrathoracic lymph nodes |
| Lymphoma | C85.83 | Other specified types of non-Hodgkin lymphoma, intra-abdominal lymph nodes |
| Lymphoma | C85.84 | Other specified types of non-Hodgkin lymphoma, lymph nodes of axilla and upper limb |
| Lymphoma | C85.85 | Other specified types of non-Hodgkin lymphoma, lymph nodes of inguinal region and lower limb |
| Lymphoma | C85.86 | Other specified types of non-Hodgkin lymphoma, intrapelvic lymph nodes |
| Lymphoma | C85.87 | Other specified types of non-Hodgkin lymphoma, spleen |
| Lymphoma | C85.88 | Other specified types of non-Hodgkin lymphoma, lymph nodes of multiple sites |
| Lymphoma | C85.89 | Other specified types of non-Hodgkin lymphoma, extranodal and solid organ sites |
| Lymphoma | C85.90 | Non-Hodgkin lymphoma, unspecified, unspecified site |
| Lymphoma | C85.91 | Non-Hodgkin lymphoma, unspecified, lymph nodes of head, face, and neck |
| Lymphoma | C85.92 | Non-Hodgkin lymphoma, unspecified, intrathoracic lymph nodes |
| Lymphoma | C85.93 | Non-Hodgkin lymphoma, unspecified, intra-abdominal lymph nodes |
| Lymphoma | C85.94 | Non-Hodgkin lymphoma, unspecified, lymph nodes of axilla and upper limb |
| Lymphoma | C85.95 | Non-Hodgkin lymphoma, unspecified, lymph nodes of inguinal region and lower limb |
| Lymphoma | C85.96 | Non-Hodgkin lymphoma, unspecified, intrapelvic lymph nodes |
| Lymphoma | C85.97 | Non-Hodgkin lymphoma, unspecified, spleen |
| Lymphoma | C85.98 | Non-Hodgkin lymphoma, unspecified, lymph nodes of multiple sites |
| Lymphoma | C85.99 | Non-Hodgkin lymphoma, unspecified, extranodal and solid organ sites |
| Lymphoma | C88.0 | Waldenström macroglobulinemia |
| Lymphoma | C88.2 | Heavy chain disease |
| Lymphoma | C88.3 | Immunoproliferative small intestinal disease |
| Lymphoma | C88.4 | Extranodal marginal zone B-cell lymphoma of mucosa-associated lymphoid tissue [MALT-lymphoma] |
| Lymphoma | C88.8 | Other malignant immunoproliferative diseases |
| Lymphoma | C88.9 | Malignant immunoproliferative disease, unspecified |
| Lymphoma | C90.00 | Multiple myeloma not having achieved remission |
| Lymphoma | C90.01 | Multiple myeloma in remission |
| Lymphoma | C90.02 | Multiple myeloma in relapse |
| Lymphoma | C90.20 | Extramedullary plasmacytoma not having achieved remission |
| Lymphoma | C90.21 | Extramedullary plasmacytoma in remission |
| Lymphoma | C90.22 | Extramedullary plasmacytoma in relapse |
| Lymphoma | C96.0 | Multifocal and multisystemic (disseminated) Langerhans-cell histiocytosis |
| Lymphoma | C96.2 | Malignant mast cell tumor |
| Lymphoma | C96.4 | Sarcoma of dendritic cells (accessory cells) |
| Lymphoma | C96.5 | Multifocal and unisystemic Langerhans-cell histiocytosis |
| Lymphoma | C96.6 | Unifocal Langerhans-cell histiocytosis |
| Lymphoma | C96.9 | Malignant neoplasm of lymphoid, hematopoietic and related tissue, unspecified |
| Lymphoma | C96.A | Histiocytic sarcoma |
| Lymphoma | C96.Z | Other specified malignant neoplasms of lymphoid, hematopoietic and related tissue |
| Metastatic Cancer | C77.0 | Secondary and unspecified malignant neoplasm of lymph nodes of head, face and neck |
| Metastatic Cancer | C77.1 | Secondary and unspecified malignant neoplasm of intrathoracic lymph nodes |
| Metastatic Cancer | C77.2 | Secondary and unspecified malignant neoplasm of intra-abdominal lymph nodes |
| Metastatic Cancer | C77.3 | Secondary and unspecified malignant neoplasm of axilla and upper limb lymph nodes |
| Metastatic Cancer | C77.4 | Secondary and unspecified malignant neoplasm of inguinal and lower limb lymph nodes |
| Metastatic Cancer | C77.5 | Secondary and unspecified malignant neoplasm of intrapelvic lymph nodes |
| Metastatic Cancer | C77.8 | Secondary and unspecified malignant neoplasm of lymph nodes of multiple regions |
| Metastatic Cancer | C77.9 | Secondary and unspecified malignant neoplasm of lymph node, unspecified |
| Metastatic Cancer | C78.00 | Secondary malignant neoplasm of unspecified lung |
| Metastatic Cancer | C78.01 | Secondary malignant neoplasm of right lung |
| Metastatic Cancer | C78.02 | Secondary malignant neoplasm of left lung |
| Metastatic Cancer | C78.1 | Secondary malignant neoplasm of mediastinum |
| Metastatic Cancer | C78.2 | Secondary malignant neoplasm of pleura |
| Metastatic Cancer | C78.30 | Secondary malignant neoplasm of unspecified respiratory organ |
| Metastatic Cancer | C78.39 | Secondary malignant neoplasm of other respiratory organs |
| Metastatic Cancer | C78.4 | Secondary malignant neoplasm of small intestine |
| Metastatic Cancer | C78.5 | Secondary malignant neoplasm of large intestine and rectum |
| Metastatic Cancer | C78.6 | Secondary malignant neoplasm of retroperitoneum and peritoneum |
| Metastatic Cancer | C78.7 | Secondary malignant neoplasm of liver and intrahepatic bile duct |
| Metastatic Cancer | C78.80 | Secondary malignant neoplasm of unspecified digestive organ |
| Metastatic Cancer | C78.89 | Secondary malignant neoplasm of other digestive organs |
| Metastatic Cancer | C79.00 | Secondary malignant neoplasm of unspecified kidney and renal pelvis |
| Metastatic Cancer | C79.01 | Secondary malignant neoplasm of right kidney and renal pelvis |
| Metastatic Cancer | C79.02 | Secondary malignant neoplasm of left kidney and renal pelvis |
| Metastatic Cancer | C79.10 | Secondary malignant neoplasm of unspecified urinary organs |
| Metastatic Cancer | C79.11 | Secondary malignant neoplasm of bladder |
| Metastatic Cancer | C79.19 | Secondary malignant neoplasm of other urinary organs |
| Metastatic Cancer | C79.2 | Secondary malignant neoplasm of skin |
| Metastatic Cancer | C79.31 | Secondary malignant neoplasm of brain |
| Metastatic Cancer | C79.32 | Secondary malignant neoplasm of cerebral meninges |
| Metastatic Cancer | C79.40 | Secondary malignant neoplasm of unspecified part of nervous system |
| Metastatic Cancer | C79.49 | Secondary malignant neoplasm of other parts of nervous system |
| Metastatic Cancer | C79.51 | Secondary malignant neoplasm of bone |
| Metastatic Cancer | C79.52 | Secondary malignant neoplasm of bone marrow |
| Metastatic Cancer | C79.60 | Secondary malignant neoplasm of unspecified ovary |
| Metastatic Cancer | C79.61 | Secondary malignant neoplasm of right ovary |
| Metastatic Cancer | C79.62 | Secondary malignant neoplasm of left ovary |
| Metastatic Cancer | C79.70 | Secondary malignant neoplasm of unspecified adrenal gland |
| Metastatic Cancer | C79.71 | Secondary malignant neoplasm of right adrenal gland |
| Metastatic Cancer | C79.72 | Secondary malignant neoplasm of left adrenal gland |
| Metastatic Cancer | C79.81 | Secondary malignant neoplasm of breast |
| Metastatic Cancer | C79.82 | Secondary malignant neoplasm of genital organs |
| Metastatic Cancer | C79.89 | Secondary malignant neoplasm of other specified sites |
| Metastatic Cancer | C79.9 | Secondary malignant neoplasm of unspecified site |
| Metastatic Cancer | C80.0 | Disseminated malignant neoplasm, unspecified |
| Metastatic Cancer | C80.1 | Malignant (primary) neoplasm, unspecified |
| Metastatic Cancer | C80.2 | Malignant neoplasm associated with transplanted organ |
| Obesity | E66.01 | Morbid (severe) obesity due to excess calories |
| Obesity | E66.09 | Other obesity due to excess calories |
| Obesity | E66.1 | Drug-induced obesity |
| Obesity | E66.2 | Morbid (severe) obesity with alveolar hypoventilation |
| Obesity | E66.3 | Overweight |
| Obesity | E66.8 | Other obesity |
| Obesity | E66.9 | Obesity, unspecified |
| Other Neurological Disorders | G10 | Huntington's disease |
| Other Neurological Disorders | G11.0 | Congenital nonprogressive ataxia |
| Other Neurological Disorders | G11.1 | Early-onset cerebellar ataxia |
| Other Neurological Disorders | G11.2 | Late-onset cerebellar ataxia |
| Other Neurological Disorders | G11.3 | Cerebellar ataxia with defective DNA repair |
| Other Neurological Disorders | G11.4 | Hereditary spastic paraplegia |
| Other Neurological Disorders | G11.8 | Other hereditary ataxias |
| Other Neurological Disorders | G11.9 | Hereditary ataxia, unspecified |
| Other Neurological Disorders | G12.0 | Infantile spinal muscular atrophy, type I [Werdnig-Hoffman] |
| Other Neurological Disorders | G12.1 | Other inherited spinal muscular atrophy |
| Other Neurological Disorders | G12.20 | Motor neuron disease, unspecified |
| Other Neurological Disorders | G12.21 | Amyotrophic lateral sclerosis |
| Other Neurological Disorders | G12.22 | Progressive bulbar palsy |
| Other Neurological Disorders | G12.29 | Other motor neuron disease |
| Other Neurological Disorders | G12.8 | Other spinal muscular atrophies and related syndromes |
| Other Neurological Disorders | G12.9 | Spinal muscular atrophy, unspecified |
| Other Neurological Disorders | G13.0 | Paraneoplastic neuromyopathy and neuropathy |
| Other Neurological Disorders | G13.1 | Other systemic atrophy primarily affecting central nervous system in neoplastic disease |
| Other Neurological Disorders | G13.2 | Systemic atrophy primarily affecting the central nervous system in myxedema |
| Other Neurological Disorders | G13.8 | Systemic atrophy primarily affecting central nervous system in other diseases classified elsewhere |
| Other Neurological Disorders | G20 | Parkinson's disease |
| Other Neurological Disorders | G21.0 | Malignant neuroleptic syndrome |
| Other Neurological Disorders | G21.11 | Neuroleptic induced parkinsonism |
| Other Neurological Disorders | G21.19 | Other drug induced secondary parkinsonism |
| Other Neurological Disorders | G21.2 | Secondary parkinsonism due to other external agents |
| Other Neurological Disorders | G21.3 | Postencephalitic parkinsonism |
| Other Neurological Disorders | G21.4 | Vascular parkinsonism |
| Other Neurological Disorders | G21.8 | Other secondary parkinsonism |
| Other Neurological Disorders | G21.9 | Secondary parkinsonism, unspecified |
| Other Neurological Disorders | G25.4 | Drug-induced chorea |
| Other Neurological Disorders | G25.5 | Other chorea |
| Other Neurological Disorders | G31.2 | Degeneration of nervous system due to alcohol |
| Other Neurological Disorders | G31.81 | Alpers disease |
| Other Neurological Disorders | G31.82 | Leigh's disease |
| Other Neurological Disorders | G31.83 | Neurocognitive disorder with Lewy bodies |
| Other Neurological Disorders | G31.84 | Mild cognitive impairment of uncertain or unknown etiology |
| Other Neurological Disorders | G31.85 | Corticobasal degeneration |
| Other Neurological Disorders | G31.89 | Other specified degenerative diseases of nervous system |
| Other Neurological Disorders | G31.9 | Degenerative disease of nervous system, unspecified |
| Other Neurological Disorders | G32.0 | Subacute combined degeneration of spinal cord in diseases classified elsewhere |
| Other Neurological Disorders | G32.81 | Cerebellar ataxia in diseases classified elsewhere |
| Other Neurological Disorders | G32.89 | Other specified degenerative disorders of nervous system in diseases classified elsewhere |
| Other Neurological Disorders | G35 | Multiple sclerosis |
| Other Neurological Disorders | G36.0 | Neuromyelitis optica [Devic] |
| Other Neurological Disorders | G36.1 | Acute and subacute hemorrhagic leukoencephalitis [Hurst] |
| Other Neurological Disorders | G36.8 | Other specified acute disseminated demyelination |
| Other Neurological Disorders | G36.9 | Acute disseminated demyelination, unspecified |
| Other Neurological Disorders | G37.0 | Diffuse sclerosis of central nervous system |
| Other Neurological Disorders | G37.1 | Central demyelination of corpus callosum |
| Other Neurological Disorders | G37.2 | Central pontine myelinolysis |
| Other Neurological Disorders | G37.3 | Acute transverse myelitis in demyelinating disease of central nervous system |
| Other Neurological Disorders | G37.4 | Subacute necrotizing myelitis of central nervous system |
| Other Neurological Disorders | G37.5 | Concentric sclerosis [Balo] of central nervous system |
| Other Neurological Disorders | G37.8 | Other specified demyelinating diseases of central nervous system |
| Other Neurological Disorders | G37.9 | Demyelinating disease of central nervous system, unspecified |
| Other Neurological Disorders | G40.001 | Localization-related (focal) (partial) idiopathic epilepsy and epileptic syndromes with seizures of localized onset, not intractable, with status epilepticus |
| Other Neurological Disorders | G40.009 | Localization-related (focal) (partial) idiopathic epilepsy and epileptic syndromes with seizures of localized onset, not intractable, without status epilepticus |
| Other Neurological Disorders | G40.011 | Localization-related (focal) (partial) idiopathic epilepsy and epileptic syndromes with seizures of localized onset, intractable, with status epilepticus |
| Other Neurological Disorders | G40.019 | Localization-related (focal) (partial) idiopathic epilepsy and epileptic syndromes with seizures of localized onset, intractable, without status epilepticus |
| Other Neurological Disorders | G40.101 | Localization-related (focal) (partial) symptomatic epilepsy and epileptic syndromes with simple partial seizures, not intractable, with status epilepticus |
| Other Neurological Disorders | G40.109 | Localization-related (focal) (partial) symptomatic epilepsy and epileptic syndromes with simple partial seizures, not intractable, without status epilepticus |
| Other Neurological Disorders | G40.111 | Localization-related (focal) (partial) symptomatic epilepsy and epileptic syndromes with simple partial seizures, intractable, with status epilepticus |
| Other Neurological Disorders | G40.119 | Localization-related (focal) (partial) symptomatic epilepsy and epileptic syndromes with simple partial seizures, intractable, without status epilepticus |
| Other Neurological Disorders | G40.201 | Localization-related (focal) (partial) symptomatic epilepsy and epileptic syndromes with complex partial seizures, not intractable, with status epilepticus |
| Other Neurological Disorders | G40.209 | Localization-related (focal) (partial) symptomatic epilepsy and epileptic syndromes with complex partial seizures, not intractable, without status epilepticus |
| Other Neurological Disorders | G40.211 | Localization-related (focal) (partial) symptomatic epilepsy and epileptic syndromes with complex partial seizures, intractable, with status epilepticus |
| Other Neurological Disorders | G40.219 | Localization-related (focal) (partial) symptomatic epilepsy and epileptic syndromes with complex partial seizures, intractable, without status epilepticus |
| Other Neurological Disorders | G40.301 | Generalized idiopathic epilepsy and epileptic syndromes, not intractable, with status epilepticus |
| Other Neurological Disorders | G40.309 | Generalized idiopathic epilepsy and epileptic syndromes, not intractable, without status epilepticus |
| Other Neurological Disorders | G40.311 | Generalized idiopathic epilepsy and epileptic syndromes, intractable, with status epilepticus |
| Other Neurological Disorders | G40.319 | Generalized idiopathic epilepsy and epileptic syndromes, intractable, without status epilepticus |
| Other Neurological Disorders | G40.401 | Other generalized epilepsy and epileptic syndromes, not intractable, with status epilepticus |
| Other Neurological Disorders | G40.409 | Other generalized epilepsy and epileptic syndromes, not intractable, without status epilepticus |
| Other Neurological Disorders | G40.411 | Other generalized epilepsy and epileptic syndromes, intractable, with status epilepticus |
| Other Neurological Disorders | G40.419 | Other generalized epilepsy and epileptic syndromes, intractable, without status epilepticus |
| Other Neurological Disorders | G40.501 | Epileptic seizures related to external causes, not intractable, with status epilepticus |
| Other Neurological Disorders | G40.509 | Epileptic seizures related to external causes, not intractable, without status epilepticus |
| Other Neurological Disorders | G40.801 | Other epilepsy, not intractable, with status epilepticus |
| Other Neurological Disorders | G40.802 | Other epilepsy, not intractable, without status epilepticus |
| Other Neurological Disorders | G40.803 | Other epilepsy, intractable, with status epilepticus |
| Other Neurological Disorders | G40.804 | Other epilepsy, intractable, without status epilepticus |
| Other Neurological Disorders | G40.811 | Lennox-Gastaut syndrome, not intractable, with status epilepticus |
| Other Neurological Disorders | G40.812 | Lennox-Gastaut syndrome, not intractable, without status epilepticus |
| Other Neurological Disorders | G40.813 | Lennox-Gastaut syndrome, intractable, with status epilepticus |
| Other Neurological Disorders | G40.814 | Lennox-Gastaut syndrome, intractable, without status epilepticus |
| Other Neurological Disorders | G40.821 | Epileptic spasms, not intractable, with status epilepticus |
| Other Neurological Disorders | G40.822 | Epileptic spasms, not intractable, without status epilepticus |
| Other Neurological Disorders | G40.823 | Epileptic spasms, intractable, with status epilepticus |
| Other Neurological Disorders | G40.824 | Epileptic spasms, intractable, without status epilepticus |
| Other Neurological Disorders | G40.89 | Other seizures |
| Other Neurological Disorders | G40.901 | Epilepsy, unspecified, not intractable, with status epilepticus |
| Other Neurological Disorders | G40.909 | Epilepsy, unspecified, not intractable, without status epilepticus |
| Other Neurological Disorders | G40.911 | Epilepsy, unspecified, intractable, with status epilepticus |
| Other Neurological Disorders | G40.919 | Epilepsy, unspecified, intractable, without status epilepticus |
| Other Neurological Disorders | G40.A01 | Absence epileptic syndrome, not intractable, with status epilepticus |
| Other Neurological Disorders | G40.A09 | Absence epileptic syndrome, not intractable, without status epilepticus |
| Other Neurological Disorders | G40.A11 | Absence epileptic syndrome, intractable, with status epilepticus |
| Other Neurological Disorders | G40.A19 | Absence epileptic syndrome, intractable, without status epilepticus |
| Other Neurological Disorders | G40.B01 | Juvenile myoclonic epilepsy, not intractable, with status epilepticus |
| Other Neurological Disorders | G40.B09 | Juvenile myoclonic epilepsy, not intractable, without status epilepticus |
| Other Neurological Disorders | G40.B11 | Juvenile myoclonic epilepsy, intractable, with status epilepticus |
| Other Neurological Disorders | G40.B19 | Juvenile myoclonic epilepsy, intractable, without status epilepticus |
| Other Neurological Disorders | G93.1 | Anoxic brain damage, not elsewhere classified |
| Other Neurological Disorders | G93.40 | Encephalopathy, unspecified |
| Other Neurological Disorders | G93.41 | Metabolic encephalopathy |
| Other Neurological Disorders | G93.49 | Other encephalopathy |
| Other Neurological Disorders | R47.01 | Aphasia |
| Other Neurological Disorders | R47.02 | Dysphasia |
| Other Neurological Disorders | R56.00 | Simple febrile convulsions |
| Other Neurological Disorders | R56.01 | Complex febrile convulsions |
| Other Neurological Disorders | R56.1 | Post traumatic seizures |
| Other Neurological Disorders | R56.9 | Unspecified convulsions |
| Paralysis | G04.1 | Tropical spastic paraplegia |
| Paralysis | G11.4 | Hereditary spastic paraplegia |
| Paralysis | G80.1 | Spastic diplegic cerebral palsy |
| Paralysis | G80.2 | Spastic hemiplegic cerebral palsy |
| Paralysis | G81.00 | Flaccid hemiplegia affecting unspecified side |
| Paralysis | G81.01 | Flaccid hemiplegia affecting right dominant side |
| Paralysis | G81.02 | Flaccid hemiplegia affecting left dominant side |
| Paralysis | G81.03 | Flaccid hemiplegia affecting right nondominant side |
| Paralysis | G81.04 | Flaccid hemiplegia affecting left nondominant side |
| Paralysis | G81.10 | Spastic hemiplegia affecting unspecified side |
| Paralysis | G81.11 | Spastic hemiplegia affecting right dominant side |
| Paralysis | G81.12 | Spastic hemiplegia affecting left dominant side |
| Paralysis | G81.13 | Spastic hemiplegia affecting right nondominant side |
| Paralysis | G81.14 | Spastic hemiplegia affecting left nondominant side |
| Paralysis | G81.90 | Hemiplegia, unspecified affecting unspecified side |
| Paralysis | G81.91 | Hemiplegia, unspecified affecting right dominant side |
| Paralysis | G81.92 | Hemiplegia, unspecified affecting left dominant side |
| Paralysis | G81.93 | Hemiplegia, unspecified affecting right nondominant side |
| Paralysis | G81.94 | Hemiplegia, unspecified affecting left nondominant side |
| Paralysis | G82.20 | Paraplegia, unspecified |
| Paralysis | G82.21 | Paraplegia, complete |
| Paralysis | G82.22 | Paraplegia, incomplete |
| Paralysis | G82.50 | Quadriplegia, unspecified |
| Paralysis | G82.51 | Quadriplegia, C1-C4 complete |
| Paralysis | G82.52 | Quadriplegia, C1-C4 incomplete |
| Paralysis | G82.53 | Quadriplegia, C5-C7 complete |
| Paralysis | G82.54 | Quadriplegia, C5-C7 incomplete |
| Paralysis | G83.0 | Diplegia of upper limbs |
| Paralysis | G83.10 | Monoplegia of lower limb affecting unspecified side |
| Paralysis | G83.11 | Monoplegia of lower limb affecting right dominant side |
| Paralysis | G83.12 | Monoplegia of lower limb affecting left dominant side |
| Paralysis | G83.13 | Monoplegia of lower limb affecting right nondominant side |
| Paralysis | G83.14 | Monoplegia of lower limb affecting left nondominant side |
| Paralysis | G83.20 | Monoplegia of upper limb affecting unspecified side |
| Paralysis | G83.21 | Monoplegia of upper limb affecting right dominant side |
| Paralysis | G83.22 | Monoplegia of upper limb affecting left dominant side |
| Paralysis | G83.23 | Monoplegia of upper limb affecting right nondominant side |
| Paralysis | G83.24 | Monoplegia of upper limb affecting left nondominant side |
| Paralysis | G83.30 | Monoplegia, unspecified affecting unspecified side |
| Paralysis | G83.31 | Monoplegia, unspecified affecting right dominant side |
| Paralysis | G83.32 | Monoplegia, unspecified affecting left dominant side |
| Paralysis | G83.33 | Monoplegia, unspecified affecting right nondominant side |
| Paralysis | G83.34 | Monoplegia, unspecified affecting left nondominant side |
| Paralysis | G83.4 | Cauda equina syndrome |
| Paralysis | G83.9 | Paralytic syndrome, unspecified |
| Peptic Ulcer Disease Excluding Bleeding | K25.7 | Chronic gastric ulcer without hemorrhage or perforation |
| Peptic Ulcer Disease Excluding Bleeding | K25.9 | Gastric ulcer, unspecified as acute or chronic, without hemorrhage or perforation |
| Peptic Ulcer Disease Excluding Bleeding | K26.7 | Chronic duodenal ulcer without hemorrhage or perforation |
| Peptic Ulcer Disease Excluding Bleeding | K26.9 | Duodenal ulcer, unspecified as acute or chronic, without hemorrhage or perforation |
| Peptic Ulcer Disease Excluding Bleeding | K27.7 | Chronic peptic ulcer, site unspecified, without hemorrhage or perforation |
| Peptic Ulcer Disease Excluding Bleeding | K27.9 | Peptic ulcer, site unspecified, unspecified as acute or chronic, without hemorrhage or perforation |
| Peptic Ulcer Disease Excluding Bleeding | K28.7 | Chronic gastrojejunal ulcer without hemorrhage or perforation |
| Peptic Ulcer Disease Excluding Bleeding | K28.9 | Gastrojejunal ulcer, unspecified as acute or chronic, without hemorrhage or perforation |
| Peripheral Vascular Disease | I70.0 | Atherosclerosis of aorta |
| Peripheral Vascular Disease | I70.1 | Atherosclerosis of renal artery |
| Peripheral Vascular Disease | I70.201 | Unspecified atherosclerosis of native arteries of extremities, right leg |
| Peripheral Vascular Disease | I70.202 | Unspecified atherosclerosis of native arteries of extremities, left leg |
| Peripheral Vascular Disease | I70.203 | Unspecified atherosclerosis of native arteries of extremities, bilateral legs |
| Peripheral Vascular Disease | I70.208 | Unspecified atherosclerosis of native arteries of extremities, other extremity |
| Peripheral Vascular Disease | I70.209 | Unspecified atherosclerosis of native arteries of extremities, unspecified extremity |
| Peripheral Vascular Disease | I70.211 | Atherosclerosis of native arteries of extremities with intermittent claudication, right leg |
| Peripheral Vascular Disease | I70.212 | Atherosclerosis of native arteries of extremities with intermittent claudication, left leg |
| Peripheral Vascular Disease | I70.213 | Atherosclerosis of native arteries of extremities with intermittent claudication, bilateral legs |
| Peripheral Vascular Disease | I70.218 | Atherosclerosis of native arteries of extremities with intermittent claudication, other extremity |
| Peripheral Vascular Disease | I70.219 | Atherosclerosis of native arteries of extremities with intermittent claudication, unspecified extremity |
| Peripheral Vascular Disease | I70.221 | Atherosclerosis of native arteries of extremities with rest pain, right leg |
| Peripheral Vascular Disease | I70.222 | Atherosclerosis of native arteries of extremities with rest pain, left leg |
| Peripheral Vascular Disease | I70.223 | Atherosclerosis of native arteries of extremities with rest pain, bilateral legs |
| Peripheral Vascular Disease | I70.228 | Atherosclerosis of native arteries of extremities with rest pain, other extremity |
| Peripheral Vascular Disease | I70.229 | Atherosclerosis of native arteries of extremities with rest pain, unspecified extremity |
| Peripheral Vascular Disease | I70.231 | Atherosclerosis of native arteries of right leg with ulceration of thigh |
| Peripheral Vascular Disease | I70.232 | Atherosclerosis of native arteries of right leg with ulceration of calf |
| Peripheral Vascular Disease | I70.233 | Atherosclerosis of native arteries of right leg with ulceration of ankle |
| Peripheral Vascular Disease | I70.234 | Atherosclerosis of native arteries of right leg with ulceration of heel and midfoot |
| Peripheral Vascular Disease | I70.235 | Atherosclerosis of native arteries of right leg with ulceration of other part of foot |
| Peripheral Vascular Disease | I70.238 | Atherosclerosis of native arteries of right leg with ulceration of other part of lower leg |
| Peripheral Vascular Disease | I70.239 | Atherosclerosis of native arteries of right leg with ulceration of unspecified site |
| Peripheral Vascular Disease | I70.241 | Atherosclerosis of native arteries of left leg with ulceration of thigh |
| Peripheral Vascular Disease | I70.242 | Atherosclerosis of native arteries of left leg with ulceration of calf |
| Peripheral Vascular Disease | I70.243 | Atherosclerosis of native arteries of left leg with ulceration of ankle |
| Peripheral Vascular Disease | I70.244 | Atherosclerosis of native arteries of left leg with ulceration of heel and midfoot |
| Peripheral Vascular Disease | I70.245 | Atherosclerosis of native arteries of left leg with ulceration of other part of foot |
| Peripheral Vascular Disease | I70.248 | Atherosclerosis of native arteries of left leg with ulceration of other part of lower leg |
| Peripheral Vascular Disease | I70.249 | Atherosclerosis of native arteries of left leg with ulceration of unspecified site |
| Peripheral Vascular Disease | I70.25 | Atherosclerosis of native arteries of other extremities with ulceration |
| Peripheral Vascular Disease | I70.261 | Atherosclerosis of native arteries of extremities with gangrene, right leg |
| Peripheral Vascular Disease | I70.262 | Atherosclerosis of native arteries of extremities with gangrene, left leg |
| Peripheral Vascular Disease | I70.263 | Atherosclerosis of native arteries of extremities with gangrene, bilateral legs |
| Peripheral Vascular Disease | I70.268 | Atherosclerosis of native arteries of extremities with gangrene, other extremity |
| Peripheral Vascular Disease | I70.269 | Atherosclerosis of native arteries of extremities with gangrene, unspecified extremity |
| Peripheral Vascular Disease | I70.291 | Other atherosclerosis of native arteries of extremities, right leg |
| Peripheral Vascular Disease | I70.292 | Other atherosclerosis of native arteries of extremities, left leg |
| Peripheral Vascular Disease | I70.293 | Other atherosclerosis of native arteries of extremities, bilateral legs |
| Peripheral Vascular Disease | I70.298 | Other atherosclerosis of native arteries of extremities, other extremity |
| Peripheral Vascular Disease | I70.299 | Other atherosclerosis of native arteries of extremities, unspecified extremity |
| Peripheral Vascular Disease | I70.301 | Unspecified atherosclerosis of unspecified type of bypass graft(s) of the extremities, right leg |
| Peripheral Vascular Disease | I70.302 | Unspecified atherosclerosis of unspecified type of bypass graft(s) of the extremities, left leg |
| Peripheral Vascular Disease | I70.303 | Unspecified atherosclerosis of unspecified type of bypass graft(s) of the extremities, bilateral legs |
| Peripheral Vascular Disease | I70.308 | Unspecified atherosclerosis of unspecified type of bypass graft(s) of the extremities, other extremity |
| Peripheral Vascular Disease | I70.309 | Unspecified atherosclerosis of unspecified type of bypass graft(s) of the extremities, unspecified extremity |
| Peripheral Vascular Disease | I70.311 | Atherosclerosis of unspecified type of bypass graft(s) of the extremities with intermittent claudication, right leg |
| Peripheral Vascular Disease | I70.312 | Atherosclerosis of unspecified type of bypass graft(s) of the extremities with intermittent claudication, left leg |
| Peripheral Vascular Disease | I70.313 | Atherosclerosis of unspecified type of bypass graft(s) of the extremities with intermittent claudication, bilateral legs |
| Peripheral Vascular Disease | I70.318 | Atherosclerosis of unspecified type of bypass graft(s) of the extremities with intermittent claudication, other extremity |
| Peripheral Vascular Disease | I70.319 | Atherosclerosis of unspecified type of bypass graft(s) of the extremities with intermittent claudication, unspecified extremity |
| Peripheral Vascular Disease | I70.321 | Atherosclerosis of unspecified type of bypass graft(s) of the extremities with rest pain, right leg |
| Peripheral Vascular Disease | I70.322 | Atherosclerosis of unspecified type of bypass graft(s) of the extremities with rest pain, left leg |
| Peripheral Vascular Disease | I70.323 | Atherosclerosis of unspecified type of bypass graft(s) of the extremities with rest pain, bilateral legs |
| Peripheral Vascular Disease | I70.328 | Atherosclerosis of unspecified type of bypass graft(s) of the extremities with rest pain, other extremity |
| Peripheral Vascular Disease | I70.329 | Atherosclerosis of unspecified type of bypass graft(s) of the extremities with rest pain, unspecified extremity |
| Peripheral Vascular Disease | I70.331 | Atherosclerosis of unspecified type of bypass graft(s) of the right leg with ulceration of thigh |
| Peripheral Vascular Disease | I70.332 | Atherosclerosis of unspecified type of bypass graft(s) of the right leg with ulceration of calf |
| Peripheral Vascular Disease | I70.333 | Atherosclerosis of unspecified type of bypass graft(s) of the right leg with ulceration of ankle |
| Peripheral Vascular Disease | I70.334 | Atherosclerosis of unspecified type of bypass graft(s) of the right leg with ulceration of heel and midfoot |
| Peripheral Vascular Disease | I70.335 | Atherosclerosis of unspecified type of bypass graft(s) of the right leg with ulceration of other part of foot |
| Peripheral Vascular Disease | I70.338 | Atherosclerosis of unspecified type of bypass graft(s) of the right leg with ulceration of other part of lower leg |
| Peripheral Vascular Disease | I70.339 | Atherosclerosis of unspecified type of bypass graft(s) of the right leg with ulceration of unspecified site |
| Peripheral Vascular Disease | I70.341 | Atherosclerosis of unspecified type of bypass graft(s) of the left leg with ulceration of thigh |
| Peripheral Vascular Disease | I70.342 | Atherosclerosis of unspecified type of bypass graft(s) of the left leg with ulceration of calf |
| Peripheral Vascular Disease | I70.343 | Atherosclerosis of unspecified type of bypass graft(s) of the left leg with ulceration of ankle |
| Peripheral Vascular Disease | I70.344 | Atherosclerosis of unspecified type of bypass graft(s) of the left leg with ulceration of heel and midfoot |
| Peripheral Vascular Disease | I70.345 | Atherosclerosis of unspecified type of bypass graft(s) of the left leg with ulceration of other part of foot |
| Peripheral Vascular Disease | I70.348 | Atherosclerosis of unspecified type of bypass graft(s) of the left leg with ulceration of other part of lower leg |
| Peripheral Vascular Disease | I70.349 | Atherosclerosis of unspecified type of bypass graft(s) of the left leg with ulceration of unspecified site |
| Peripheral Vascular Disease | I70.35 | Atherosclerosis of unspecified type of bypass graft(s) of other extremity with ulceration |
| Peripheral Vascular Disease | I70.361 | Atherosclerosis of unspecified type of bypass graft(s) of the extremities with gangrene, right leg |
| Peripheral Vascular Disease | I70.362 | Atherosclerosis of unspecified type of bypass graft(s) of the extremities with gangrene, left leg |
| Peripheral Vascular Disease | I70.363 | Atherosclerosis of unspecified type of bypass graft(s) of the extremities with gangrene, bilateral legs |
| Peripheral Vascular Disease | I70.368 | Atherosclerosis of unspecified type of bypass graft(s) of the extremities with gangrene, other extremity |
| Peripheral Vascular Disease | I70.369 | Atherosclerosis of unspecified type of bypass graft(s) of the extremities with gangrene, unspecified extremity |
| Peripheral Vascular Disease | I70.391 | Other atherosclerosis of unspecified type of bypass graft(s) of the extremities, right leg |
| Peripheral Vascular Disease | I70.392 | Other atherosclerosis of unspecified type of bypass graft(s) of the extremities, left leg |
| Peripheral Vascular Disease | I70.393 | Other atherosclerosis of unspecified type of bypass graft(s) of the extremities, bilateral legs |
| Peripheral Vascular Disease | I70.398 | Other atherosclerosis of unspecified type of bypass graft(s) of the extremities, other extremity |
| Peripheral Vascular Disease | I70.399 | Other atherosclerosis of unspecified type of bypass graft(s) of the extremities, unspecified extremity |
| Peripheral Vascular Disease | I70.401 | Unspecified atherosclerosis of autologous vein bypass graft(s) of the extremities, right leg |
| Peripheral Vascular Disease | I70.402 | Unspecified atherosclerosis of autologous vein bypass graft(s) of the extremities, left leg |
| Peripheral Vascular Disease | I70.403 | Unspecified atherosclerosis of autologous vein bypass graft(s) of the extremities, bilateral legs |
| Peripheral Vascular Disease | I70.408 | Unspecified atherosclerosis of autologous vein bypass graft(s) of the extremities, other extremity |
| Peripheral Vascular Disease | I70.409 | Unspecified atherosclerosis of autologous vein bypass graft(s) of the extremities, unspecified extremity |
| Peripheral Vascular Disease | I70.411 | Atherosclerosis of autologous vein bypass graft(s) of the extremities with intermittent claudication, right leg |
| Peripheral Vascular Disease | I70.412 | Atherosclerosis of autologous vein bypass graft(s) of the extremities with intermittent claudication, left leg |
| Peripheral Vascular Disease | I70.413 | Atherosclerosis of autologous vein bypass graft(s) of the extremities with intermittent claudication, bilateral legs |
| Peripheral Vascular Disease | I70.418 | Atherosclerosis of autologous vein bypass graft(s) of the extremities with intermittent claudication, other extremity |
| Peripheral Vascular Disease | I70.419 | Atherosclerosis of autologous vein bypass graft(s) of the extremities with intermittent claudication, unspecified extremity |
| Peripheral Vascular Disease | I70.421 | Atherosclerosis of autologous vein bypass graft(s) of the extremities with rest pain, right leg |
| Peripheral Vascular Disease | I70.422 | Atherosclerosis of autologous vein bypass graft(s) of the extremities with rest pain, left leg |
| Peripheral Vascular Disease | I70.423 | Atherosclerosis of autologous vein bypass graft(s) of the extremities with rest pain, bilateral legs |
| Peripheral Vascular Disease | I70.428 | Atherosclerosis of autologous vein bypass graft(s) of the extremities with rest pain, other extremity |
| Peripheral Vascular Disease | I70.429 | Atherosclerosis of autologous vein bypass graft(s) of the extremities with rest pain, unspecified extremity |
| Peripheral Vascular Disease | I70.431 | Atherosclerosis of autologous vein bypass graft(s) of the right leg with ulceration of thigh |
| Peripheral Vascular Disease | I70.432 | Atherosclerosis of autologous vein bypass graft(s) of the right leg with ulceration of calf |
| Peripheral Vascular Disease | I70.433 | Atherosclerosis of autologous vein bypass graft(s) of the right leg with ulceration of ankle |
| Peripheral Vascular Disease | I70.434 | Atherosclerosis of autologous vein bypass graft(s) of the right leg with ulceration of heel and midfoot |
| Peripheral Vascular Disease | I70.435 | Atherosclerosis of autologous vein bypass graft(s) of the right leg with ulceration of other part of foot |
| Peripheral Vascular Disease | I70.438 | Atherosclerosis of autologous vein bypass graft(s) of the right leg with ulceration of other part of lower leg |
| Peripheral Vascular Disease | I70.439 | Atherosclerosis of autologous vein bypass graft(s) of the right leg with ulceration of unspecified site |
| Peripheral Vascular Disease | I70.441 | Atherosclerosis of autologous vein bypass graft(s) of the left leg with ulceration of thigh |
| Peripheral Vascular Disease | I70.442 | Atherosclerosis of autologous vein bypass graft(s) of the left leg with ulceration of calf |
| Peripheral Vascular Disease | I70.443 | Atherosclerosis of autologous vein bypass graft(s) of the left leg with ulceration of ankle |
| Peripheral Vascular Disease | I70.444 | Atherosclerosis of autologous vein bypass graft(s) of the left leg with ulceration of heel and midfoot |
| Peripheral Vascular Disease | I70.445 | Atherosclerosis of autologous vein bypass graft(s) of the left leg with ulceration of other part of foot |
| Peripheral Vascular Disease | I70.448 | Atherosclerosis of autologous vein bypass graft(s) of the left leg with ulceration of other part of lower leg |
| Peripheral Vascular Disease | I70.449 | Atherosclerosis of autologous vein bypass graft(s) of the left leg with ulceration of unspecified site |
| Peripheral Vascular Disease | I70.45 | Atherosclerosis of autologous vein bypass graft(s) of other extremity with ulceration |
| Peripheral Vascular Disease | I70.461 | Atherosclerosis of autologous vein bypass graft(s) of the extremities with gangrene, right leg |
| Peripheral Vascular Disease | I70.462 | Atherosclerosis of autologous vein bypass graft(s) of the extremities with gangrene, left leg |
| Peripheral Vascular Disease | I70.463 | Atherosclerosis of autologous vein bypass graft(s) of the extremities with gangrene, bilateral legs |
| Peripheral Vascular Disease | I70.468 | Atherosclerosis of autologous vein bypass graft(s) of the extremities with gangrene, other extremity |
| Peripheral Vascular Disease | I70.469 | Atherosclerosis of autologous vein bypass graft(s) of the extremities with gangrene, unspecified extremity |
| Peripheral Vascular Disease | I70.491 | Other atherosclerosis of autologous vein bypass graft(s) of the extremities, right leg |
| Peripheral Vascular Disease | I70.492 | Other atherosclerosis of autologous vein bypass graft(s) of the extremities, left leg |
| Peripheral Vascular Disease | I70.493 | Other atherosclerosis of autologous vein bypass graft(s) of the extremities, bilateral legs |
| Peripheral Vascular Disease | I70.498 | Other atherosclerosis of autologous vein bypass graft(s) of the extremities, other extremity |
| Peripheral Vascular Disease | I70.499 | Other atherosclerosis of autologous vein bypass graft(s) of the extremities, unspecified extremity |
| Peripheral Vascular Disease | I70.501 | Unspecified atherosclerosis of nonautologous biological bypass graft(s) of the extremities, right leg |
| Peripheral Vascular Disease | I70.502 | Unspecified atherosclerosis of nonautologous biological bypass graft(s) of the extremities, left leg |
| Peripheral Vascular Disease | I70.503 | Unspecified atherosclerosis of nonautologous biological bypass graft(s) of the extremities, bilateral legs |
| Peripheral Vascular Disease | I70.508 | Unspecified atherosclerosis of nonautologous biological bypass graft(s) of the extremities, other extremity |
| Peripheral Vascular Disease | I70.509 | Unspecified atherosclerosis of nonautologous biological bypass graft(s) of the extremities, unspecified extremity |
| Peripheral Vascular Disease | I70.511 | Atherosclerosis of nonautologous biological bypass graft(s) of the extremities with intermittent claudication, right leg |
| Peripheral Vascular Disease | I70.512 | Atherosclerosis of nonautologous biological bypass graft(s) of the extremities with intermittent claudication, left leg |
| Peripheral Vascular Disease | I70.513 | Atherosclerosis of nonautologous biological bypass graft(s) of the extremities with intermittent claudication, bilateral legs |
| Peripheral Vascular Disease | I70.518 | Atherosclerosis of nonautologous biological bypass graft(s) of the extremities with intermittent claudication, other extremity |
| Peripheral Vascular Disease | I70.519 | Atherosclerosis of nonautologous biological bypass graft(s) of the extremities with intermittent claudication, unspecified extremity |
| Peripheral Vascular Disease | I70.521 | Atherosclerosis of nonautologous biological bypass graft(s) of the extremities with rest pain, right leg |
| Peripheral Vascular Disease | I70.522 | Atherosclerosis of nonautologous biological bypass graft(s) of the extremities with rest pain, left leg |
| Peripheral Vascular Disease | I70.523 | Atherosclerosis of nonautologous biological bypass graft(s) of the extremities with rest pain, bilateral legs |
| Peripheral Vascular Disease | I70.528 | Atherosclerosis of nonautologous biological bypass graft(s) of the extremities with rest pain, other extremity |
| Peripheral Vascular Disease | I70.529 | Atherosclerosis of nonautologous biological bypass graft(s) of the extremities with rest pain, unspecified extremity |
| Peripheral Vascular Disease | I70.531 | Atherosclerosis of nonautologous biological bypass graft(s) of the right leg with ulceration of thigh |
| Peripheral Vascular Disease | I70.532 | Atherosclerosis of nonautologous biological bypass graft(s) of the right leg with ulceration of calf |
| Peripheral Vascular Disease | I70.533 | Atherosclerosis of nonautologous biological bypass graft(s) of the right leg with ulceration of ankle |
| Peripheral Vascular Disease | I70.534 | Atherosclerosis of nonautologous biological bypass graft(s) of the right leg with ulceration of heel and midfoot |
| Peripheral Vascular Disease | I70.535 | Atherosclerosis of nonautologous biological bypass graft(s) of the right leg with ulceration of other part of foot |
| Peripheral Vascular Disease | I70.538 | Atherosclerosis of nonautologous biological bypass graft(s) of the right leg with ulceration of other part of lower leg |
| Peripheral Vascular Disease | I70.539 | Atherosclerosis of nonautologous biological bypass graft(s) of the right leg with ulceration of unspecified site |
| Peripheral Vascular Disease | I70.541 | Atherosclerosis of nonautologous biological bypass graft(s) of the left leg with ulceration of thigh |
| Peripheral Vascular Disease | I70.542 | Atherosclerosis of nonautologous biological bypass graft(s) of the left leg with ulceration of calf |
| Peripheral Vascular Disease | I70.543 | Atherosclerosis of nonautologous biological bypass graft(s) of the left leg with ulceration of ankle |
| Peripheral Vascular Disease | I70.544 | Atherosclerosis of nonautologous biological bypass graft(s) of the left leg with ulceration of heel and midfoot |
| Peripheral Vascular Disease | I70.545 | Atherosclerosis of nonautologous biological bypass graft(s) of the left leg with ulceration of other part of foot |
| Peripheral Vascular Disease | I70.548 | Atherosclerosis of nonautologous biological bypass graft(s) of the left leg with ulceration of other part of lower leg |
| Peripheral Vascular Disease | I70.549 | Atherosclerosis of nonautologous biological bypass graft(s) of the left leg with ulceration of unspecified site |
| Peripheral Vascular Disease | I70.55 | Atherosclerosis of nonautologous biological bypass graft(s) of other extremity with ulceration |
| Peripheral Vascular Disease | I70.561 | Atherosclerosis of nonautologous biological bypass graft(s) of the extremities with gangrene, right leg |
| Peripheral Vascular Disease | I70.562 | Atherosclerosis of nonautologous biological bypass graft(s) of the extremities with gangrene, left leg |
| Peripheral Vascular Disease | I70.563 | Atherosclerosis of nonautologous biological bypass graft(s) of the extremities with gangrene, bilateral legs |
| Peripheral Vascular Disease | I70.568 | Atherosclerosis of nonautologous biological bypass graft(s) of the extremities with gangrene, other extremity |
| Peripheral Vascular Disease | I70.569 | Atherosclerosis of nonautologous biological bypass graft(s) of the extremities with gangrene, unspecified extremity |
| Peripheral Vascular Disease | I70.591 | Other atherosclerosis of nonautologous biological bypass graft(s) of the extremities, right leg |
| Peripheral Vascular Disease | I70.592 | Other atherosclerosis of nonautologous biological bypass graft(s) of the extremities, left leg |
| Peripheral Vascular Disease | I70.593 | Other atherosclerosis of nonautologous biological bypass graft(s) of the extremities, bilateral legs |
| Peripheral Vascular Disease | I70.598 | Other atherosclerosis of nonautologous biological bypass graft(s) of the extremities, other extremity |
| Peripheral Vascular Disease | I70.599 | Other atherosclerosis of nonautologous biological bypass graft(s) of the extremities, unspecified extremity |
| Peripheral Vascular Disease | I70.601 | Unspecified atherosclerosis of nonbiological bypass graft(s) of the extremities, right leg |
| Peripheral Vascular Disease | I70.602 | Unspecified atherosclerosis of nonbiological bypass graft(s) of the extremities, left leg |
| Peripheral Vascular Disease | I70.603 | Unspecified atherosclerosis of nonbiological bypass graft(s) of the extremities, bilateral legs |
| Peripheral Vascular Disease | I70.608 | Unspecified atherosclerosis of nonbiological bypass graft(s) of the extremities, other extremity |
| Peripheral Vascular Disease | I70.609 | Unspecified atherosclerosis of nonbiological bypass graft(s) of the extremities, unspecified extremity |
| Peripheral Vascular Disease | I70.611 | Atherosclerosis of nonbiological bypass graft(s) of the extremities with intermittent claudication, right leg |
| Peripheral Vascular Disease | I70.612 | Atherosclerosis of nonbiological bypass graft(s) of the extremities with intermittent claudication, left leg |
| Peripheral Vascular Disease | I70.613 | Atherosclerosis of nonbiological bypass graft(s) of the extremities with intermittent claudication, bilateral legs |
| Peripheral Vascular Disease | I70.618 | Atherosclerosis of nonbiological bypass graft(s) of the extremities with intermittent claudication, other extremity |
| Peripheral Vascular Disease | I70.619 | Atherosclerosis of nonbiological bypass graft(s) of the extremities with intermittent claudication, unspecified extremity |
| Peripheral Vascular Disease | I70.621 | Atherosclerosis of nonbiological bypass graft(s) of the extremities with rest pain, right leg |
| Peripheral Vascular Disease | I70.622 | Atherosclerosis of nonbiological bypass graft(s) of the extremities with rest pain, left leg |
| Peripheral Vascular Disease | I70.623 | Atherosclerosis of nonbiological bypass graft(s) of the extremities with rest pain, bilateral legs |
| Peripheral Vascular Disease | I70.628 | Atherosclerosis of nonbiological bypass graft(s) of the extremities with rest pain, other extremity |
| Peripheral Vascular Disease | I70.629 | Atherosclerosis of nonbiological bypass graft(s) of the extremities with rest pain, unspecified extremity |
| Peripheral Vascular Disease | I70.631 | Atherosclerosis of nonbiological bypass graft(s) of the right leg with ulceration of thigh |
| Peripheral Vascular Disease | I70.632 | Atherosclerosis of nonbiological bypass graft(s) of the right leg with ulceration of calf |
| Peripheral Vascular Disease | I70.633 | Atherosclerosis of nonbiological bypass graft(s) of the right leg with ulceration of ankle |
| Peripheral Vascular Disease | I70.634 | Atherosclerosis of nonbiological bypass graft(s) of the right leg with ulceration of heel and midfoot |
| Peripheral Vascular Disease | I70.635 | Atherosclerosis of nonbiological bypass graft(s) of the right leg with ulceration of other part of foot |
| Peripheral Vascular Disease | I70.638 | Atherosclerosis of nonbiological bypass graft(s) of the right leg with ulceration of other part of lower leg |
| Peripheral Vascular Disease | I70.639 | Atherosclerosis of nonbiological bypass graft(s) of the right leg with ulceration of unspecified site |
| Peripheral Vascular Disease | I70.641 | Atherosclerosis of nonbiological bypass graft(s) of the left leg with ulceration of thigh |
| Peripheral Vascular Disease | I70.642 | Atherosclerosis of nonbiological bypass graft(s) of the left leg with ulceration of calf |
| Peripheral Vascular Disease | I70.643 | Atherosclerosis of nonbiological bypass graft(s) of the left leg with ulceration of ankle |
| Peripheral Vascular Disease | I70.644 | Atherosclerosis of nonbiological bypass graft(s) of the left leg with ulceration of heel and midfoot |
| Peripheral Vascular Disease | I70.645 | Atherosclerosis of nonbiological bypass graft(s) of the left leg with ulceration of other part of foot |
| Peripheral Vascular Disease | I70.648 | Atherosclerosis of nonbiological bypass graft(s) of the left leg with ulceration of other part of lower leg |
| Peripheral Vascular Disease | I70.649 | Atherosclerosis of nonbiological bypass graft(s) of the left leg with ulceration of unspecified site |
| Peripheral Vascular Disease | I70.65 | Atherosclerosis of nonbiological bypass graft(s) of other extremity with ulceration |
| Peripheral Vascular Disease | I70.661 | Atherosclerosis of nonbiological bypass graft(s) of the extremities with gangrene, right leg |
| Peripheral Vascular Disease | I70.662 | Atherosclerosis of nonbiological bypass graft(s) of the extremities with gangrene, left leg |
| Peripheral Vascular Disease | I70.663 | Atherosclerosis of nonbiological bypass graft(s) of the extremities with gangrene, bilateral legs |
| Peripheral Vascular Disease | I70.668 | Atherosclerosis of nonbiological bypass graft(s) of the extremities with gangrene, other extremity |
| Peripheral Vascular Disease | I70.669 | Atherosclerosis of nonbiological bypass graft(s) of the extremities with gangrene, unspecified extremity |
| Peripheral Vascular Disease | I70.691 | Other atherosclerosis of nonbiological bypass graft(s) of the extremities, right leg |
| Peripheral Vascular Disease | I70.692 | Other atherosclerosis of nonbiological bypass graft(s) of the extremities, left leg |
| Peripheral Vascular Disease | I70.693 | Other atherosclerosis of nonbiological bypass graft(s) of the extremities, bilateral legs |
| Peripheral Vascular Disease | I70.698 | Other atherosclerosis of nonbiological bypass graft(s) of the extremities, other extremity |
| Peripheral Vascular Disease | I70.699 | Other atherosclerosis of nonbiological bypass graft(s) of the extremities, unspecified extremity |
| Peripheral Vascular Disease | I70.701 | Unspecified atherosclerosis of other type of bypass graft(s) of the extremities, right leg |
| Peripheral Vascular Disease | I70.702 | Unspecified atherosclerosis of other type of bypass graft(s) of the extremities, left leg |
| Peripheral Vascular Disease | I70.703 | Unspecified atherosclerosis of other type of bypass graft(s) of the extremities, bilateral legs |
| Peripheral Vascular Disease | I70.708 | Unspecified atherosclerosis of other type of bypass graft(s) of the extremities, other extremity |
| Peripheral Vascular Disease | I70.709 | Unspecified atherosclerosis of other type of bypass graft(s) of the extremities, unspecified extremity |
| Peripheral Vascular Disease | I70.711 | Atherosclerosis of other type of bypass graft(s) of the extremities with intermittent claudication, right leg |
| Peripheral Vascular Disease | I70.712 | Atherosclerosis of other type of bypass graft(s) of the extremities with intermittent claudication, left leg |
| Peripheral Vascular Disease | I70.713 | Atherosclerosis of other type of bypass graft(s) of the extremities with intermittent claudication, bilateral legs |
| Peripheral Vascular Disease | I70.718 | Atherosclerosis of other type of bypass graft(s) of the extremities with intermittent claudication, other extremity |
| Peripheral Vascular Disease | I70.719 | Atherosclerosis of other type of bypass graft(s) of the extremities with intermittent claudication, unspecified extremity |
| Peripheral Vascular Disease | I70.721 | Atherosclerosis of other type of bypass graft(s) of the extremities with rest pain, right leg |
| Peripheral Vascular Disease | I70.722 | Atherosclerosis of other type of bypass graft(s) of the extremities with rest pain, left leg |
| Peripheral Vascular Disease | I70.723 | Atherosclerosis of other type of bypass graft(s) of the extremities with rest pain, bilateral legs |
| Peripheral Vascular Disease | I70.728 | Atherosclerosis of other type of bypass graft(s) of the extremities with rest pain, other extremity |
| Peripheral Vascular Disease | I70.729 | Atherosclerosis of other type of bypass graft(s) of the extremities with rest pain, unspecified extremity |
| Peripheral Vascular Disease | I70.731 | Atherosclerosis of other type of bypass graft(s) of the right leg with ulceration of thigh |
| Peripheral Vascular Disease | I70.732 | Atherosclerosis of other type of bypass graft(s) of the right leg with ulceration of calf |
| Peripheral Vascular Disease | I70.733 | Atherosclerosis of other type of bypass graft(s) of the right leg with ulceration of ankle |
| Peripheral Vascular Disease | I70.734 | Atherosclerosis of other type of bypass graft(s) of the right leg with ulceration of heel and midfoot |
| Peripheral Vascular Disease | I70.735 | Atherosclerosis of other type of bypass graft(s) of the right leg with ulceration of other part of foot |
| Peripheral Vascular Disease | I70.738 | Atherosclerosis of other type of bypass graft(s) of the right leg with ulceration of other part of lower leg |
| Peripheral Vascular Disease | I70.739 | Atherosclerosis of other type of bypass graft(s) of the right leg with ulceration of unspecified site |
| Peripheral Vascular Disease | I70.741 | Atherosclerosis of other type of bypass graft(s) of the left leg with ulceration of thigh |
| Peripheral Vascular Disease | I70.742 | Atherosclerosis of other type of bypass graft(s) of the left leg with ulceration of calf |
| Peripheral Vascular Disease | I70.743 | Atherosclerosis of other type of bypass graft(s) of the left leg with ulceration of ankle |
| Peripheral Vascular Disease | I70.744 | Atherosclerosis of other type of bypass graft(s) of the left leg with ulceration of heel and midfoot |
| Peripheral Vascular Disease | I70.745 | Atherosclerosis of other type of bypass graft(s) of the left leg with ulceration of other part of foot |
| Peripheral Vascular Disease | I70.748 | Atherosclerosis of other type of bypass graft(s) of the left leg with ulceration of other part of lower leg |
| Peripheral Vascular Disease | I70.749 | Atherosclerosis of other type of bypass graft(s) of the left leg with ulceration of unspecified site |
| Peripheral Vascular Disease | I70.75 | Atherosclerosis of other type of bypass graft(s) of other extremity with ulceration |
| Peripheral Vascular Disease | I70.761 | Atherosclerosis of other type of bypass graft(s) of the extremities with gangrene, right leg |
| Peripheral Vascular Disease | I70.762 | Atherosclerosis of other type of bypass graft(s) of the extremities with gangrene, left leg |
| Peripheral Vascular Disease | I70.763 | Atherosclerosis of other type of bypass graft(s) of the extremities with gangrene, bilateral legs |
| Peripheral Vascular Disease | I70.768 | Atherosclerosis of other type of bypass graft(s) of the extremities with gangrene, other extremity |
| Peripheral Vascular Disease | I70.769 | Atherosclerosis of other type of bypass graft(s) of the extremities with gangrene, unspecified extremity |
| Peripheral Vascular Disease | I70.791 | Other atherosclerosis of other type of bypass graft(s) of the extremities, right leg |
| Peripheral Vascular Disease | I70.792 | Other atherosclerosis of other type of bypass graft(s) of the extremities, left leg |
| Peripheral Vascular Disease | I70.793 | Other atherosclerosis of other type of bypass graft(s) of the extremities, bilateral legs |
| Peripheral Vascular Disease | I70.798 | Other atherosclerosis of other type of bypass graft(s) of the extremities, other extremity |
| Peripheral Vascular Disease | I70.799 | Other atherosclerosis of other type of bypass graft(s) of the extremities, unspecified extremity |
| Peripheral Vascular Disease | I70.8 | Atherosclerosis of other arteries |
| Peripheral Vascular Disease | I70.90 | Unspecified atherosclerosis |
| Peripheral Vascular Disease | I70.91 | Generalized atherosclerosis |
| Peripheral Vascular Disease | I70.92 | Chronic total occlusion of artery of the extremities |
| Peripheral Vascular Disease | I71.00 | Dissection of unspecified site of aorta |
| Peripheral Vascular Disease | I71.01 | Dissection of thoracic aorta |
| Peripheral Vascular Disease | I71.02 | Dissection of abdominal aorta |
| Peripheral Vascular Disease | I71.03 | Dissection of thoracoabdominal aorta |
| Peripheral Vascular Disease | I71.1 | Thoracic aortic aneurysm, ruptured |
| Peripheral Vascular Disease | I71.2 | Thoracic aortic aneurysm, without rupture |
| Peripheral Vascular Disease | I71.3 | Abdominal aortic aneurysm, ruptured |
| Peripheral Vascular Disease | I71.4 | Abdominal aortic aneurysm, without rupture |
| Peripheral Vascular Disease | I71.5 | Thoracoabdominal aortic aneurysm, ruptured |
| Peripheral Vascular Disease | I71.6 | Thoracoabdominal aortic aneurysm, without rupture |
| Peripheral Vascular Disease | I71.8 | Aortic aneurysm of unspecified site, ruptured |
| Peripheral Vascular Disease | I71.9 | Aortic aneurysm of unspecified site, without rupture |
| Peripheral Vascular Disease | I73.1 | Thromboangiitis obliterans [Buerger's disease] |
| Peripheral Vascular Disease | I73.81 | Erythromelalgia |
| Peripheral Vascular Disease | I73.89 | Other specified peripheral vascular diseases |
| Peripheral Vascular Disease | I73.9 | Peripheral vascular disease, unspecified |
| Peripheral Vascular Disease | I77.1 | Stricture of artery |
| Peripheral Vascular Disease | I79.0 | Aneurysm of aorta in diseases classified elsewhere |
| Peripheral Vascular Disease | K55.1 | Chronic vascular disorders of intestine |
| Peripheral Vascular Disease | K55.8 | Other vascular disorders of intestine |
| Peripheral Vascular Disease | K55.9 | Vascular disorder of intestine, unspecified |
| Peripheral Vascular Disease | Z95.810 | Presence of automatic (implantable) cardiac defibrillator |
| Peripheral Vascular Disease | Z95.811 | Presence of heart assist device |
| Peripheral Vascular Disease | Z95.812 | Presence of fully implantable artificial heart |
| Peripheral Vascular Disease | Z95.818 | Presence of other cardiac implants and grafts |
| Peripheral Vascular Disease | Z95.820 | Peripheral vascular angioplasty status with implants and grafts |
| Peripheral Vascular Disease | Z95.828 | Presence of other vascular implants and grafts |
| Peripheral Vascular Disease | Z95.9 | Presence of cardiac and vascular implant and graft, unspecified |
| Psychoses | F20.0 | Paranoid schizophrenia |
| Psychoses | F20.1 | Disorganized schizophrenia |
| Psychoses | F20.2 | Catatonic schizophrenia |
| Psychoses | F20.3 | Undifferentiated schizophrenia |
| Psychoses | F20.5 | Residual schizophrenia |
| Psychoses | F20.81 | Schizophreniform disorder |
| Psychoses | F20.89 | Other schizophrenia |
| Psychoses | F20.9 | Schizophrenia, unspecified |
| Psychoses | F22 | Delusional disorders |
| Psychoses | F23 | Brief psychotic disorder |
| Psychoses | F24 | Shared psychotic disorder |
| Psychoses | F25.0 | Schizoaffective disorder, bipolar type |
| Psychoses | F25.1 | Schizoaffective disorder, depressive type |
| Psychoses | F25.8 | Other schizoaffective disorders |
| Psychoses | F25.9 | Schizoaffective disorder, unspecified |
| Psychoses | F28 | Other psychotic disorder not due to a substance or known physiological condition |
| Psychoses | F29 | Unspecified psychosis not due to a substance or known physiological condition |
| Psychoses | F30.2 | Manic episode, severe with psychotic symptoms |
| Psychoses | F31.2 | Bipolar disorder, current episode manic severe with psychotic features |
| Psychoses | F31.5 | Bipolar disorder, current episode depressed, severe, with psychotic features |
| Pulmonary Circulation Disorders | I26.01 | Septic pulmonary embolism with acute cor pulmonale |
| Pulmonary Circulation Disorders | I26.02 | Saddle embolus of pulmonary artery with acute cor pulmonale |
| Pulmonary Circulation Disorders | I26.09 | Other pulmonary embolism with acute cor pulmonale |
| Pulmonary Circulation Disorders | I26.90 | Septic pulmonary embolism without acute cor pulmonale |
| Pulmonary Circulation Disorders | I26.92 | Saddle embolus of pulmonary artery without acute cor pulmonale |
| Pulmonary Circulation Disorders | I26.99 | Other pulmonary embolism without acute cor pulmonale |
| Pulmonary Circulation Disorders | I27.0 | Primary pulmonary hypertension |
| Pulmonary Circulation Disorders | I27.1 | Kyphoscoliotic heart disease |
| Pulmonary Circulation Disorders | I27.2 | Other secondary pulmonary hypertension |
| Pulmonary Circulation Disorders | I27.81 | Cor pulmonale (chronic) |
| Pulmonary Circulation Disorders | I27.82 | Chronic pulmonary embolism |
| Pulmonary Circulation Disorders | I27.89 | Other specified pulmonary heart diseases |
| Pulmonary Circulation Disorders | I27.9 | Pulmonary heart disease, unspecified |
| Pulmonary Circulation Disorders | I28.0 | Arteriovenous fistula of pulmonary vessels |
| Pulmonary Circulation Disorders | I28.8 | Other diseases of pulmonary vessels |
| Pulmonary Circulation Disorders | I28.9 | Disease of pulmonary vessels, unspecified |
| Renal Failure | I12.0 | Hypertensive chronic kidney disease with stage 5 chronic kidney disease or end stage renal disease |
| Renal Failure | N18.1 | Chronic kidney disease, stage 1 |
| Renal Failure | N18.2 | Chronic kidney disease, stage 2 (mild) |
| Renal Failure | N18.3 | Chronic kidney disease, stage 3 (moderate) |
| Renal Failure | N18.4 | Chronic kidney disease, stage 4 (severe) |
| Renal Failure | N18.5 | Chronic kidney disease, stage 5 |
| Renal Failure | N18.6 | End stage renal disease |
| Renal Failure | N18.9 | Chronic kidney disease, unspecified |
| Renal Failure | N19 | Unspecified kidney failure |
| Renal Failure | N25.0 | Renal osteodystrophy |
| Renal Failure | Z49.01 | Encounter for fitting and adjustment of extracorporeal dialysis catheter |
| Renal Failure | Z49.02 | Encounter for fitting and adjustment of peritoneal dialysis catheter |
| Renal Failure | Z94.0 | Kidney transplant status |
| Renal Failure | Z99.2 | Dependence on renal dialysis |
| Rheumatoid Arthritis or Collagen Vascular Diseases | L94.0 | Localized scleroderma [morphea] |
| Rheumatoid Arthritis or Collagen Vascular Diseases | L94.1 | Linear scleroderma |
| Rheumatoid Arthritis or Collagen Vascular Diseases | L94.3 | Sclerodactyly |
| Rheumatoid Arthritis or Collagen Vascular Diseases | M05.00 | Felty's syndrome, unspecified site |
| Rheumatoid Arthritis or Collagen Vascular Diseases | M05.011 | Felty's syndrome, right shoulder |
| Rheumatoid Arthritis or Collagen Vascular Diseases | M05.012 | Felty's syndrome, left shoulder |
| Rheumatoid Arthritis or Collagen Vascular Diseases | M05.019 | Felty's syndrome, unspecified shoulder |
| Rheumatoid Arthritis or Collagen Vascular Diseases | M05.021 | Felty's syndrome, right elbow |
| Rheumatoid Arthritis or Collagen Vascular Diseases | M05.022 | Felty's syndrome, left elbow |
| Rheumatoid Arthritis or Collagen Vascular Diseases | M05.029 | Felty's syndrome, unspecified elbow |
| Rheumatoid Arthritis or Collagen Vascular Diseases | M05.031 | Felty's syndrome, right wrist |
| Rheumatoid Arthritis or Collagen Vascular Diseases | M05.032 | Felty's syndrome, left wrist |
| Rheumatoid Arthritis or Collagen Vascular Diseases | M05.039 | Felty's syndrome, unspecified wrist |
| Rheumatoid Arthritis or Collagen Vascular Diseases | M05.041 | Felty's syndrome, right hand |
| Rheumatoid Arthritis or Collagen Vascular Diseases | M05.042 | Felty's syndrome, left hand |
| Rheumatoid Arthritis or Collagen Vascular Diseases | M05.049 | Felty's syndrome, unspecified hand |
| Rheumatoid Arthritis or Collagen Vascular Diseases | M05.051 | Felty's syndrome, right hip |
| Rheumatoid Arthritis or Collagen Vascular Diseases | M05.052 | Felty's syndrome, left hip |
| Rheumatoid Arthritis or Collagen Vascular Diseases | M05.059 | Felty's syndrome, unspecified hip |
| Rheumatoid Arthritis or Collagen Vascular Diseases | M05.061 | Felty's syndrome, right knee |
| Rheumatoid Arthritis or Collagen Vascular Diseases | M05.062 | Felty's syndrome, left knee |
| Rheumatoid Arthritis or Collagen Vascular Diseases | M05.069 | Felty's syndrome, unspecified knee |
| Rheumatoid Arthritis or Collagen Vascular Diseases | M05.071 | Felty's syndrome, right ankle and foot |
| Rheumatoid Arthritis or Collagen Vascular Diseases | M05.072 | Felty's syndrome, left ankle and foot |
| Rheumatoid Arthritis or Collagen Vascular Diseases | M05.079 | Felty's syndrome, unspecified ankle and foot |
| Rheumatoid Arthritis or Collagen Vascular Diseases | M05.09 | Felty's syndrome, multiple sites |
| Rheumatoid Arthritis or Collagen Vascular Diseases | M05.10 | Rheumatoid lung disease with rheumatoid arthritis of unspecified site |
| Rheumatoid Arthritis or Collagen Vascular Diseases | M05.111 | Rheumatoid lung disease with rheumatoid arthritis of right shoulder |
| Rheumatoid Arthritis or Collagen Vascular Diseases | M05.112 | Rheumatoid lung disease with rheumatoid arthritis of left shoulder |
| Rheumatoid Arthritis or Collagen Vascular Diseases | M05.119 | Rheumatoid lung disease with rheumatoid arthritis of unspecified shoulder |
| Rheumatoid Arthritis or Collagen Vascular Diseases | M05.121 | Rheumatoid lung disease with rheumatoid arthritis of right elbow |
| Rheumatoid Arthritis or Collagen Vascular Diseases | M05.122 | Rheumatoid lung disease with rheumatoid arthritis of left elbow |
| Rheumatoid Arthritis or Collagen Vascular Diseases | M05.129 | Rheumatoid lung disease with rheumatoid arthritis of unspecified elbow |
| Rheumatoid Arthritis or Collagen Vascular Diseases | M05.131 | Rheumatoid lung disease with rheumatoid arthritis of right wrist |
| Rheumatoid Arthritis or Collagen Vascular Diseases | M05.132 | Rheumatoid lung disease with rheumatoid arthritis of left wrist |
| Rheumatoid Arthritis or Collagen Vascular Diseases | M05.139 | Rheumatoid lung disease with rheumatoid arthritis of unspecified wrist |
| Rheumatoid Arthritis or Collagen Vascular Diseases | M05.141 | Rheumatoid lung disease with rheumatoid arthritis of right hand |
| Rheumatoid Arthritis or Collagen Vascular Diseases | M05.142 | Rheumatoid lung disease with rheumatoid arthritis of left hand |
| Rheumatoid Arthritis or Collagen Vascular Diseases | M05.149 | Rheumatoid lung disease with rheumatoid arthritis of unspecified hand |
| Rheumatoid Arthritis or Collagen Vascular Diseases | M05.151 | Rheumatoid lung disease with rheumatoid arthritis of right hip |
| Rheumatoid Arthritis or Collagen Vascular Diseases | M05.152 | Rheumatoid lung disease with rheumatoid arthritis of left hip |
| Rheumatoid Arthritis or Collagen Vascular Diseases | M05.159 | Rheumatoid lung disease with rheumatoid arthritis of unspecified hip |
| Rheumatoid Arthritis or Collagen Vascular Diseases | M05.161 | Rheumatoid lung disease with rheumatoid arthritis of right knee |
| Rheumatoid Arthritis or Collagen Vascular Diseases | M05.162 | Rheumatoid lung disease with rheumatoid arthritis of left knee |
| Rheumatoid Arthritis or Collagen Vascular Diseases | M05.169 | Rheumatoid lung disease with rheumatoid arthritis of unspecified knee |
| Rheumatoid Arthritis or Collagen Vascular Diseases | M05.171 | Rheumatoid lung disease with rheumatoid arthritis of right ankle and foot |
| Rheumatoid Arthritis or Collagen Vascular Diseases | M05.172 | Rheumatoid lung disease with rheumatoid arthritis of left ankle and foot |
| Rheumatoid Arthritis or Collagen Vascular Diseases | M05.179 | Rheumatoid lung disease with rheumatoid arthritis of unspecified ankle and foot |
| Rheumatoid Arthritis or Collagen Vascular Diseases | M05.19 | Rheumatoid lung disease with rheumatoid arthritis of multiple sites |
| Rheumatoid Arthritis or Collagen Vascular Diseases | M05.20 | Rheumatoid vasculitis with rheumatoid arthritis of unspecified site |
| Rheumatoid Arthritis or Collagen Vascular Diseases | M05.211 | Rheumatoid vasculitis with rheumatoid arthritis of right shoulder |
| Rheumatoid Arthritis or Collagen Vascular Diseases | M05.212 | Rheumatoid vasculitis with rheumatoid arthritis of left shoulder |
| Rheumatoid Arthritis or Collagen Vascular Diseases | M05.219 | Rheumatoid vasculitis with rheumatoid arthritis of unspecified shoulder |
| Rheumatoid Arthritis or Collagen Vascular Diseases | M05.221 | Rheumatoid vasculitis with rheumatoid arthritis of right elbow |
| Rheumatoid Arthritis or Collagen Vascular Diseases | M05.222 | Rheumatoid vasculitis with rheumatoid arthritis of left elbow |
| Rheumatoid Arthritis or Collagen Vascular Diseases | M05.229 | Rheumatoid vasculitis with rheumatoid arthritis of unspecified elbow |
| Rheumatoid Arthritis or Collagen Vascular Diseases | M05.231 | Rheumatoid vasculitis with rheumatoid arthritis of right wrist |
| Rheumatoid Arthritis or Collagen Vascular Diseases | M05.232 | Rheumatoid vasculitis with rheumatoid arthritis of left wrist |
| Rheumatoid Arthritis or Collagen Vascular Diseases | M05.239 | Rheumatoid vasculitis with rheumatoid arthritis of unspecified wrist |
| Rheumatoid Arthritis or Collagen Vascular Diseases | M05.241 | Rheumatoid vasculitis with rheumatoid arthritis of right hand |
| Rheumatoid Arthritis or Collagen Vascular Diseases | M05.242 | Rheumatoid vasculitis with rheumatoid arthritis of left hand |
| Rheumatoid Arthritis or Collagen Vascular Diseases | M05.249 | Rheumatoid vasculitis with rheumatoid arthritis of unspecified hand |
| Rheumatoid Arthritis or Collagen Vascular Diseases | M05.251 | Rheumatoid vasculitis with rheumatoid arthritis of right hip |
| Rheumatoid Arthritis or Collagen Vascular Diseases | M05.252 | Rheumatoid vasculitis with rheumatoid arthritis of left hip |
| Rheumatoid Arthritis or Collagen Vascular Diseases | M05.259 | Rheumatoid vasculitis with rheumatoid arthritis of unspecified hip |
| Rheumatoid Arthritis or Collagen Vascular Diseases | M05.261 | Rheumatoid vasculitis with rheumatoid arthritis of right knee |
| Rheumatoid Arthritis or Collagen Vascular Diseases | M05.262 | Rheumatoid vasculitis with rheumatoid arthritis of left knee |
| Rheumatoid Arthritis or Collagen Vascular Diseases | M05.269 | Rheumatoid vasculitis with rheumatoid arthritis of unspecified knee |
| Rheumatoid Arthritis or Collagen Vascular Diseases | M05.271 | Rheumatoid vasculitis with rheumatoid arthritis of right ankle and foot |
| Rheumatoid Arthritis or Collagen Vascular Diseases | M05.272 | Rheumatoid vasculitis with rheumatoid arthritis of left ankle and foot |
| Rheumatoid Arthritis or Collagen Vascular Diseases | M05.279 | Rheumatoid vasculitis with rheumatoid arthritis of unspecified ankle and foot |
| Rheumatoid Arthritis or Collagen Vascular Diseases | M05.29 | Rheumatoid vasculitis with rheumatoid arthritis of multiple sites |
| Rheumatoid Arthritis or Collagen Vascular Diseases | M05.30 | Rheumatoid heart disease with rheumatoid arthritis of unspecified site |
| Rheumatoid Arthritis or Collagen Vascular Diseases | M05.311 | Rheumatoid heart disease with rheumatoid arthritis of right shoulder |
| Rheumatoid Arthritis or Collagen Vascular Diseases | M05.312 | Rheumatoid heart disease with rheumatoid arthritis of left shoulder |
| Rheumatoid Arthritis or Collagen Vascular Diseases | M05.319 | Rheumatoid heart disease with rheumatoid arthritis of unspecified shoulder |
| Rheumatoid Arthritis or Collagen Vascular Diseases | M05.321 | Rheumatoid heart disease with rheumatoid arthritis of right elbow |
| Rheumatoid Arthritis or Collagen Vascular Diseases | M05.322 | Rheumatoid heart disease with rheumatoid arthritis of left elbow |
| Rheumatoid Arthritis or Collagen Vascular Diseases | M05.329 | Rheumatoid heart disease with rheumatoid arthritis of unspecified elbow |
| Rheumatoid Arthritis or Collagen Vascular Diseases | M05.331 | Rheumatoid heart disease with rheumatoid arthritis of right wrist |
| Rheumatoid Arthritis or Collagen Vascular Diseases | M05.332 | Rheumatoid heart disease with rheumatoid arthritis of left wrist |
| Rheumatoid Arthritis or Collagen Vascular Diseases | M05.339 | Rheumatoid heart disease with rheumatoid arthritis of unspecified wrist |
| Rheumatoid Arthritis or Collagen Vascular Diseases | M05.341 | Rheumatoid heart disease with rheumatoid arthritis of right hand |
| Rheumatoid Arthritis or Collagen Vascular Diseases | M05.342 | Rheumatoid heart disease with rheumatoid arthritis of left hand |
| Rheumatoid Arthritis or Collagen Vascular Diseases | M05.349 | Rheumatoid heart disease with rheumatoid arthritis of unspecified hand |
| Rheumatoid Arthritis or Collagen Vascular Diseases | M05.351 | Rheumatoid heart disease with rheumatoid arthritis of right hip |
| Rheumatoid Arthritis or Collagen Vascular Diseases | M05.352 | Rheumatoid heart disease with rheumatoid arthritis of left hip |
| Rheumatoid Arthritis or Collagen Vascular Diseases | M05.359 | Rheumatoid heart disease with rheumatoid arthritis of unspecified hip |
| Rheumatoid Arthritis or Collagen Vascular Diseases | M05.361 | Rheumatoid heart disease with rheumatoid arthritis of right knee |
| Rheumatoid Arthritis or Collagen Vascular Diseases | M05.362 | Rheumatoid heart disease with rheumatoid arthritis of left knee |
| Rheumatoid Arthritis or Collagen Vascular Diseases | M05.369 | Rheumatoid heart disease with rheumatoid arthritis of unspecified knee |
| Rheumatoid Arthritis or Collagen Vascular Diseases | M05.371 | Rheumatoid heart disease with rheumatoid arthritis of right ankle and foot |
| Rheumatoid Arthritis or Collagen Vascular Diseases | M05.372 | Rheumatoid heart disease with rheumatoid arthritis of left ankle and foot |
| Rheumatoid Arthritis or Collagen Vascular Diseases | M05.379 | Rheumatoid heart disease with rheumatoid arthritis of unspecified ankle and foot |
| Rheumatoid Arthritis or Collagen Vascular Diseases | M05.39 | Rheumatoid heart disease with rheumatoid arthritis of multiple sites |
| Rheumatoid Arthritis or Collagen Vascular Diseases | M05.40 | Rheumatoid myopathy with rheumatoid arthritis of unspecified site |
| Rheumatoid Arthritis or Collagen Vascular Diseases | M05.411 | Rheumatoid myopathy with rheumatoid arthritis of right shoulder |
| Rheumatoid Arthritis or Collagen Vascular Diseases | M05.412 | Rheumatoid myopathy with rheumatoid arthritis of left shoulder |
| Rheumatoid Arthritis or Collagen Vascular Diseases | M05.419 | Rheumatoid myopathy with rheumatoid arthritis of unspecified shoulder |
| Rheumatoid Arthritis or Collagen Vascular Diseases | M05.421 | Rheumatoid myopathy with rheumatoid arthritis of right elbow |
| Rheumatoid Arthritis or Collagen Vascular Diseases | M05.422 | Rheumatoid myopathy with rheumatoid arthritis of left elbow |
| Rheumatoid Arthritis or Collagen Vascular Diseases | M05.429 | Rheumatoid myopathy with rheumatoid arthritis of unspecified elbow |
| Rheumatoid Arthritis or Collagen Vascular Diseases | M05.431 | Rheumatoid myopathy with rheumatoid arthritis of right wrist |
| Rheumatoid Arthritis or Collagen Vascular Diseases | M05.432 | Rheumatoid myopathy with rheumatoid arthritis of left wrist |
| Rheumatoid Arthritis or Collagen Vascular Diseases | M05.439 | Rheumatoid myopathy with rheumatoid arthritis of unspecified wrist |
| Rheumatoid Arthritis or Collagen Vascular Diseases | M05.441 | Rheumatoid myopathy with rheumatoid arthritis of right hand |
| Rheumatoid Arthritis or Collagen Vascular Diseases | M05.442 | Rheumatoid myopathy with rheumatoid arthritis of left hand |
| Rheumatoid Arthritis or Collagen Vascular Diseases | M05.449 | Rheumatoid myopathy with rheumatoid arthritis of unspecified hand |
| Rheumatoid Arthritis or Collagen Vascular Diseases | M05.451 | Rheumatoid myopathy with rheumatoid arthritis of right hip |
| Rheumatoid Arthritis or Collagen Vascular Diseases | M05.452 | Rheumatoid myopathy with rheumatoid arthritis of left hip |
| Rheumatoid Arthritis or Collagen Vascular Diseases | M05.459 | Rheumatoid myopathy with rheumatoid arthritis of unspecified hip |
| Rheumatoid Arthritis or Collagen Vascular Diseases | M05.461 | Rheumatoid myopathy with rheumatoid arthritis of right knee |
| Rheumatoid Arthritis or Collagen Vascular Diseases | M05.462 | Rheumatoid myopathy with rheumatoid arthritis of left knee |
| Rheumatoid Arthritis or Collagen Vascular Diseases | M05.469 | Rheumatoid myopathy with rheumatoid arthritis of unspecified knee |
| Rheumatoid Arthritis or Collagen Vascular Diseases | M05.471 | Rheumatoid myopathy with rheumatoid arthritis of right ankle and foot |
| Rheumatoid Arthritis or Collagen Vascular Diseases | M05.472 | Rheumatoid myopathy with rheumatoid arthritis of left ankle and foot |
| Rheumatoid Arthritis or Collagen Vascular Diseases | M05.479 | Rheumatoid myopathy with rheumatoid arthritis of unspecified ankle and foot |
| Rheumatoid Arthritis or Collagen Vascular Diseases | M05.49 | Rheumatoid myopathy with rheumatoid arthritis of multiple sites |
| Rheumatoid Arthritis or Collagen Vascular Diseases | M05.50 | Rheumatoid polyneuropathy with rheumatoid arthritis of unspecified site |
| Rheumatoid Arthritis or Collagen Vascular Diseases | M05.511 | Rheumatoid polyneuropathy with rheumatoid arthritis of right shoulder |
| Rheumatoid Arthritis or Collagen Vascular Diseases | M05.512 | Rheumatoid polyneuropathy with rheumatoid arthritis of left shoulder |
| Rheumatoid Arthritis or Collagen Vascular Diseases | M05.519 | Rheumatoid polyneuropathy with rheumatoid arthritis of unspecified shoulder |
| Rheumatoid Arthritis or Collagen Vascular Diseases | M05.521 | Rheumatoid polyneuropathy with rheumatoid arthritis of right elbow |
| Rheumatoid Arthritis or Collagen Vascular Diseases | M05.522 | Rheumatoid polyneuropathy with rheumatoid arthritis of left elbow |
| Rheumatoid Arthritis or Collagen Vascular Diseases | M05.529 | Rheumatoid polyneuropathy with rheumatoid arthritis of unspecified elbow |
| Rheumatoid Arthritis or Collagen Vascular Diseases | M05.531 | Rheumatoid polyneuropathy with rheumatoid arthritis of right wrist |
| Rheumatoid Arthritis or Collagen Vascular Diseases | M05.532 | Rheumatoid polyneuropathy with rheumatoid arthritis of left wrist |
| Rheumatoid Arthritis or Collagen Vascular Diseases | M05.539 | Rheumatoid polyneuropathy with rheumatoid arthritis of unspecified wrist |
| Rheumatoid Arthritis or Collagen Vascular Diseases | M05.541 | Rheumatoid polyneuropathy with rheumatoid arthritis of right hand |
| Rheumatoid Arthritis or Collagen Vascular Diseases | M05.542 | Rheumatoid polyneuropathy with rheumatoid arthritis of left hand |
| Rheumatoid Arthritis or Collagen Vascular Diseases | M05.549 | Rheumatoid polyneuropathy with rheumatoid arthritis of unspecified hand |
| Rheumatoid Arthritis or Collagen Vascular Diseases | M05.551 | Rheumatoid polyneuropathy with rheumatoid arthritis of right hip |
| Rheumatoid Arthritis or Collagen Vascular Diseases | M05.552 | Rheumatoid polyneuropathy with rheumatoid arthritis of left hip |
| Rheumatoid Arthritis or Collagen Vascular Diseases | M05.559 | Rheumatoid polyneuropathy with rheumatoid arthritis of unspecified hip |
| Rheumatoid Arthritis or Collagen Vascular Diseases | M05.561 | Rheumatoid polyneuropathy with rheumatoid arthritis of right knee |
| Rheumatoid Arthritis or Collagen Vascular Diseases | M05.562 | Rheumatoid polyneuropathy with rheumatoid arthritis of left knee |
| Rheumatoid Arthritis or Collagen Vascular Diseases | M05.569 | Rheumatoid polyneuropathy with rheumatoid arthritis of unspecified knee |
| Rheumatoid Arthritis or Collagen Vascular Diseases | M05.571 | Rheumatoid polyneuropathy with rheumatoid arthritis of right ankle and foot |
| Rheumatoid Arthritis or Collagen Vascular Diseases | M05.572 | Rheumatoid polyneuropathy with rheumatoid arthritis of left ankle and foot |
| Rheumatoid Arthritis or Collagen Vascular Diseases | M05.579 | Rheumatoid polyneuropathy with rheumatoid arthritis of unspecified ankle and foot |
| Rheumatoid Arthritis or Collagen Vascular Diseases | M05.59 | Rheumatoid polyneuropathy with rheumatoid arthritis of multiple sites |
| Rheumatoid Arthritis or Collagen Vascular Diseases | M05.60 | Rheumatoid arthritis of unspecified site with involvement of other organs and systems |
| Rheumatoid Arthritis or Collagen Vascular Diseases | M05.611 | Rheumatoid arthritis of right shoulder with involvement of other organs and systems |
| Rheumatoid Arthritis or Collagen Vascular Diseases | M05.612 | Rheumatoid arthritis of left shoulder with involvement of other organs and systems |
| Rheumatoid Arthritis or Collagen Vascular Diseases | M05.619 | Rheumatoid arthritis of unspecified shoulder with involvement of other organs and systems |
| Rheumatoid Arthritis or Collagen Vascular Diseases | M05.621 | Rheumatoid arthritis of right elbow with involvement of other organs and systems |
| Rheumatoid Arthritis or Collagen Vascular Diseases | M05.622 | Rheumatoid arthritis of left elbow with involvement of other organs and systems |
| Rheumatoid Arthritis or Collagen Vascular Diseases | M05.629 | Rheumatoid arthritis of unspecified elbow with involvement of other organs and systems |
| Rheumatoid Arthritis or Collagen Vascular Diseases | M05.631 | Rheumatoid arthritis of right wrist with involvement of other organs and systems |
| Rheumatoid Arthritis or Collagen Vascular Diseases | M05.632 | Rheumatoid arthritis of left wrist with involvement of other organs and systems |
| Rheumatoid Arthritis or Collagen Vascular Diseases | M05.639 | Rheumatoid arthritis of unspecified wrist with involvement of other organs and systems |
| Rheumatoid Arthritis or Collagen Vascular Diseases | M05.641 | Rheumatoid arthritis of right hand with involvement of other organs and systems |
| Rheumatoid Arthritis or Collagen Vascular Diseases | M05.642 | Rheumatoid arthritis of left hand with involvement of other organs and systems |
| Rheumatoid Arthritis or Collagen Vascular Diseases | M05.649 | Rheumatoid arthritis of unspecified hand with involvement of other organs and systems |
| Rheumatoid Arthritis or Collagen Vascular Diseases | M05.651 | Rheumatoid arthritis of right hip with involvement of other organs and systems |
| Rheumatoid Arthritis or Collagen Vascular Diseases | M05.652 | Rheumatoid arthritis of left hip with involvement of other organs and systems |
| Rheumatoid Arthritis or Collagen Vascular Diseases | M05.659 | Rheumatoid arthritis of unspecified hip with involvement of other organs and systems |
| Rheumatoid Arthritis or Collagen Vascular Diseases | M05.661 | Rheumatoid arthritis of right knee with involvement of other organs and systems |
| Rheumatoid Arthritis or Collagen Vascular Diseases | M05.662 | Rheumatoid arthritis of left knee with involvement of other organs and systems |
| Rheumatoid Arthritis or Collagen Vascular Diseases | M05.669 | Rheumatoid arthritis of unspecified knee with involvement of other organs and systems |
| Rheumatoid Arthritis or Collagen Vascular Diseases | M05.671 | Rheumatoid arthritis of right ankle and foot with involvement of other organs and systems |
| Rheumatoid Arthritis or Collagen Vascular Diseases | M05.672 | Rheumatoid arthritis of left ankle and foot with involvement of other organs and systems |
| Rheumatoid Arthritis or Collagen Vascular Diseases | M05.679 | Rheumatoid arthritis of unspecified ankle and foot with involvement of other organs and systems |
| Rheumatoid Arthritis or Collagen Vascular Diseases | M05.69 | Rheumatoid arthritis of multiple sites with involvement of other organs and systems |
| Rheumatoid Arthritis or Collagen Vascular Diseases | M05.70 | Rheumatoid arthritis with rheumatoid factor of unspecified site without organ or systems involvement |
| Rheumatoid Arthritis or Collagen Vascular Diseases | M05.711 | Rheumatoid arthritis with rheumatoid factor of right shoulder without organ or systems involvement |
| Rheumatoid Arthritis or Collagen Vascular Diseases | M05.712 | Rheumatoid arthritis with rheumatoid factor of left shoulder without organ or systems involvement |
| Rheumatoid Arthritis or Collagen Vascular Diseases | M05.719 | Rheumatoid arthritis with rheumatoid factor of unspecified shoulder without organ or systems involvement |
| Rheumatoid Arthritis or Collagen Vascular Diseases | M05.721 | Rheumatoid arthritis with rheumatoid factor of right elbow without organ or systems involvement |
| Rheumatoid Arthritis or Collagen Vascular Diseases | M05.722 | Rheumatoid arthritis with rheumatoid factor of left elbow without organ or systems involvement |
| Rheumatoid Arthritis or Collagen Vascular Diseases | M05.729 | Rheumatoid arthritis with rheumatoid factor of unspecified elbow without organ or systems involvement |
| Rheumatoid Arthritis or Collagen Vascular Diseases | M05.731 | Rheumatoid arthritis with rheumatoid factor of right wrist without organ or systems involvement |
| Rheumatoid Arthritis or Collagen Vascular Diseases | M05.732 | Rheumatoid arthritis with rheumatoid factor of left wrist without organ or systems involvement |
| Rheumatoid Arthritis or Collagen Vascular Diseases | M05.739 | Rheumatoid arthritis with rheumatoid factor of unspecified wrist without organ or systems involvement |
| Rheumatoid Arthritis or Collagen Vascular Diseases | M05.741 | Rheumatoid arthritis with rheumatoid factor of right hand without organ or systems involvement |
| Rheumatoid Arthritis or Collagen Vascular Diseases | M05.742 | Rheumatoid arthritis with rheumatoid factor of left hand without organ or systems involvement |
| Rheumatoid Arthritis or Collagen Vascular Diseases | M05.749 | Rheumatoid arthritis with rheumatoid factor of unspecified hand without organ or systems involvement |
| Rheumatoid Arthritis or Collagen Vascular Diseases | M05.751 | Rheumatoid arthritis with rheumatoid factor of right hip without organ or systems involvement |
| Rheumatoid Arthritis or Collagen Vascular Diseases | M05.752 | Rheumatoid arthritis with rheumatoid factor of left hip without organ or systems involvement |
| Rheumatoid Arthritis or Collagen Vascular Diseases | M05.759 | Rheumatoid arthritis with rheumatoid factor of unspecified hip without organ or systems involvement |
| Rheumatoid Arthritis or Collagen Vascular Diseases | M05.761 | Rheumatoid arthritis with rheumatoid factor of right knee without organ or systems involvement |
| Rheumatoid Arthritis or Collagen Vascular Diseases | M05.762 | Rheumatoid arthritis with rheumatoid factor of left knee without organ or systems involvement |
| Rheumatoid Arthritis or Collagen Vascular Diseases | M05.769 | Rheumatoid arthritis with rheumatoid factor of unspecified knee without organ or systems involvement |
| Rheumatoid Arthritis or Collagen Vascular Diseases | M05.771 | Rheumatoid arthritis with rheumatoid factor of right ankle and foot without organ or systems involvement |
| Rheumatoid Arthritis or Collagen Vascular Diseases | M05.772 | Rheumatoid arthritis with rheumatoid factor of left ankle and foot without organ or systems involvement |
| Rheumatoid Arthritis or Collagen Vascular Diseases | M05.779 | Rheumatoid arthritis with rheumatoid factor of unspecified ankle and foot without organ or systems involvement |
| Rheumatoid Arthritis or Collagen Vascular Diseases | M05.79 | Rheumatoid arthritis with rheumatoid factor of multiple sites without organ or systems involvement |
| Rheumatoid Arthritis or Collagen Vascular Diseases | M05.80 | Other rheumatoid arthritis with rheumatoid factor of unspecified site |
| Rheumatoid Arthritis or Collagen Vascular Diseases | M05.811 | Other rheumatoid arthritis with rheumatoid factor of right shoulder |
| Rheumatoid Arthritis or Collagen Vascular Diseases | M05.812 | Other rheumatoid arthritis with rheumatoid factor of left shoulder |
| Rheumatoid Arthritis or Collagen Vascular Diseases | M05.819 | Other rheumatoid arthritis with rheumatoid factor of unspecified shoulder |
| Rheumatoid Arthritis or Collagen Vascular Diseases | M05.821 | Other rheumatoid arthritis with rheumatoid factor of right elbow |
| Rheumatoid Arthritis or Collagen Vascular Diseases | M05.822 | Other rheumatoid arthritis with rheumatoid factor of left elbow |
| Rheumatoid Arthritis or Collagen Vascular Diseases | M05.829 | Other rheumatoid arthritis with rheumatoid factor of unspecified elbow |
| Rheumatoid Arthritis or Collagen Vascular Diseases | M05.831 | Other rheumatoid arthritis with rheumatoid factor of right wrist |
| Rheumatoid Arthritis or Collagen Vascular Diseases | M05.832 | Other rheumatoid arthritis with rheumatoid factor of left wrist |
| Rheumatoid Arthritis or Collagen Vascular Diseases | M05.839 | Other rheumatoid arthritis with rheumatoid factor of unspecified wrist |
| Rheumatoid Arthritis or Collagen Vascular Diseases | M05.841 | Other rheumatoid arthritis with rheumatoid factor of right hand |
| Rheumatoid Arthritis or Collagen Vascular Diseases | M05.842 | Other rheumatoid arthritis with rheumatoid factor of left hand |
| Rheumatoid Arthritis or Collagen Vascular Diseases | M05.849 | Other rheumatoid arthritis with rheumatoid factor of unspecified hand |
| Rheumatoid Arthritis or Collagen Vascular Diseases | M05.851 | Other rheumatoid arthritis with rheumatoid factor of right hip |
| Rheumatoid Arthritis or Collagen Vascular Diseases | M05.852 | Other rheumatoid arthritis with rheumatoid factor of left hip |
| Rheumatoid Arthritis or Collagen Vascular Diseases | M05.859 | Other rheumatoid arthritis with rheumatoid factor of unspecified hip |
| Rheumatoid Arthritis or Collagen Vascular Diseases | M05.861 | Other rheumatoid arthritis with rheumatoid factor of right knee |
| Rheumatoid Arthritis or Collagen Vascular Diseases | M05.862 | Other rheumatoid arthritis with rheumatoid factor of left knee |
| Rheumatoid Arthritis or Collagen Vascular Diseases | M05.869 | Other rheumatoid arthritis with rheumatoid factor of unspecified knee |
| Rheumatoid Arthritis or Collagen Vascular Diseases | M05.871 | Other rheumatoid arthritis with rheumatoid factor of right ankle and foot |
| Rheumatoid Arthritis or Collagen Vascular Diseases | M05.872 | Other rheumatoid arthritis with rheumatoid factor of left ankle and foot |
| Rheumatoid Arthritis or Collagen Vascular Diseases | M05.879 | Other rheumatoid arthritis with rheumatoid factor of unspecified ankle and foot |
| Rheumatoid Arthritis or Collagen Vascular Diseases | M05.89 | Other rheumatoid arthritis with rheumatoid factor of multiple sites |
| Rheumatoid Arthritis or Collagen Vascular Diseases | M05.9 | Rheumatoid arthritis with rheumatoid factor, unspecified |
| Rheumatoid Arthritis or Collagen Vascular Diseases | M06.00 | Rheumatoid arthritis without rheumatoid factor, unspecified site |
| Rheumatoid Arthritis or Collagen Vascular Diseases | M06.011 | Rheumatoid arthritis without rheumatoid factor, right shoulder |
| Rheumatoid Arthritis or Collagen Vascular Diseases | M06.012 | Rheumatoid arthritis without rheumatoid factor, left shoulder |
| Rheumatoid Arthritis or Collagen Vascular Diseases | M06.019 | Rheumatoid arthritis without rheumatoid factor, unspecified shoulder |
| Rheumatoid Arthritis or Collagen Vascular Diseases | M06.021 | Rheumatoid arthritis without rheumatoid factor, right elbow |
| Rheumatoid Arthritis or Collagen Vascular Diseases | M06.022 | Rheumatoid arthritis without rheumatoid factor, left elbow |
| Rheumatoid Arthritis or Collagen Vascular Diseases | M06.029 | Rheumatoid arthritis without rheumatoid factor, unspecified elbow |
| Rheumatoid Arthritis or Collagen Vascular Diseases | M06.031 | Rheumatoid arthritis without rheumatoid factor, right wrist |
| Rheumatoid Arthritis or Collagen Vascular Diseases | M06.032 | Rheumatoid arthritis without rheumatoid factor, left wrist |
| Rheumatoid Arthritis or Collagen Vascular Diseases | M06.039 | Rheumatoid arthritis without rheumatoid factor, unspecified wrist |
| Rheumatoid Arthritis or Collagen Vascular Diseases | M06.041 | Rheumatoid arthritis without rheumatoid factor, right hand |
| Rheumatoid Arthritis or Collagen Vascular Diseases | M06.042 | Rheumatoid arthritis without rheumatoid factor, left hand |
| Rheumatoid Arthritis or Collagen Vascular Diseases | M06.049 | Rheumatoid arthritis without rheumatoid factor, unspecified hand |
| Rheumatoid Arthritis or Collagen Vascular Diseases | M06.051 | Rheumatoid arthritis without rheumatoid factor, right hip |
| Rheumatoid Arthritis or Collagen Vascular Diseases | M06.052 | Rheumatoid arthritis without rheumatoid factor, left hip |
| Rheumatoid Arthritis or Collagen Vascular Diseases | M06.059 | Rheumatoid arthritis without rheumatoid factor, unspecified hip |
| Rheumatoid Arthritis or Collagen Vascular Diseases | M06.061 | Rheumatoid arthritis without rheumatoid factor, right knee |
| Rheumatoid Arthritis or Collagen Vascular Diseases | M06.062 | Rheumatoid arthritis without rheumatoid factor, left knee |
| Rheumatoid Arthritis or Collagen Vascular Diseases | M06.069 | Rheumatoid arthritis without rheumatoid factor, unspecified knee |
| Rheumatoid Arthritis or Collagen Vascular Diseases | M06.071 | Rheumatoid arthritis without rheumatoid factor, right ankle and foot |
| Rheumatoid Arthritis or Collagen Vascular Diseases | M06.072 | Rheumatoid arthritis without rheumatoid factor, left ankle and foot |
| Rheumatoid Arthritis or Collagen Vascular Diseases | M06.079 | Rheumatoid arthritis without rheumatoid factor, unspecified ankle and foot |
| Rheumatoid Arthritis or Collagen Vascular Diseases | M06.08 | Rheumatoid arthritis without rheumatoid factor, vertebrae |
| Rheumatoid Arthritis or Collagen Vascular Diseases | M06.09 | Rheumatoid arthritis without rheumatoid factor, multiple sites |
| Rheumatoid Arthritis or Collagen Vascular Diseases | M06.1 | Adult-onset Still's disease |
| Rheumatoid Arthritis or Collagen Vascular Diseases | M06.20 | Rheumatoid bursitis, unspecified site |
| Rheumatoid Arthritis or Collagen Vascular Diseases | M06.211 | Rheumatoid bursitis, right shoulder |
| Rheumatoid Arthritis or Collagen Vascular Diseases | M06.212 | Rheumatoid bursitis, left shoulder |
| Rheumatoid Arthritis or Collagen Vascular Diseases | M06.219 | Rheumatoid bursitis, unspecified shoulder |
| Rheumatoid Arthritis or Collagen Vascular Diseases | M06.221 | Rheumatoid bursitis, right elbow |
| Rheumatoid Arthritis or Collagen Vascular Diseases | M06.222 | Rheumatoid bursitis, left elbow |
| Rheumatoid Arthritis or Collagen Vascular Diseases | M06.229 | Rheumatoid bursitis, unspecified elbow |
| Rheumatoid Arthritis or Collagen Vascular Diseases | M06.231 | Rheumatoid bursitis, right wrist |
| Rheumatoid Arthritis or Collagen Vascular Diseases | M06.232 | Rheumatoid bursitis, left wrist |
| Rheumatoid Arthritis or Collagen Vascular Diseases | M06.239 | Rheumatoid bursitis, unspecified wrist |
| Rheumatoid Arthritis or Collagen Vascular Diseases | M06.241 | Rheumatoid bursitis, right hand |
| Rheumatoid Arthritis or Collagen Vascular Diseases | M06.242 | Rheumatoid bursitis, left hand |
| Rheumatoid Arthritis or Collagen Vascular Diseases | M06.249 | Rheumatoid bursitis, unspecified hand |
| Rheumatoid Arthritis or Collagen Vascular Diseases | M06.251 | Rheumatoid bursitis, right hip |
| Rheumatoid Arthritis or Collagen Vascular Diseases | M06.252 | Rheumatoid bursitis, left hip |
| Rheumatoid Arthritis or Collagen Vascular Diseases | M06.259 | Rheumatoid bursitis, unspecified hip |
| Rheumatoid Arthritis or Collagen Vascular Diseases | M06.261 | Rheumatoid bursitis, right knee |
| Rheumatoid Arthritis or Collagen Vascular Diseases | M06.262 | Rheumatoid bursitis, left knee |
| Rheumatoid Arthritis or Collagen Vascular Diseases | M06.269 | Rheumatoid bursitis, unspecified knee |
| Rheumatoid Arthritis or Collagen Vascular Diseases | M06.271 | Rheumatoid bursitis, right ankle and foot |
| Rheumatoid Arthritis or Collagen Vascular Diseases | M06.272 | Rheumatoid bursitis, left ankle and foot |
| Rheumatoid Arthritis or Collagen Vascular Diseases | M06.279 | Rheumatoid bursitis, unspecified ankle and foot |
| Rheumatoid Arthritis or Collagen Vascular Diseases | M06.28 | Rheumatoid bursitis, vertebrae |
| Rheumatoid Arthritis or Collagen Vascular Diseases | M06.29 | Rheumatoid bursitis, multiple sites |
| Rheumatoid Arthritis or Collagen Vascular Diseases | M06.30 | Rheumatoid nodule, unspecified site |
| Rheumatoid Arthritis or Collagen Vascular Diseases | M06.311 | Rheumatoid nodule, right shoulder |
| Rheumatoid Arthritis or Collagen Vascular Diseases | M06.312 | Rheumatoid nodule, left shoulder |
| Rheumatoid Arthritis or Collagen Vascular Diseases | M06.319 | Rheumatoid nodule, unspecified shoulder |
| Rheumatoid Arthritis or Collagen Vascular Diseases | M06.321 | Rheumatoid nodule, right elbow |
| Rheumatoid Arthritis or Collagen Vascular Diseases | M06.322 | Rheumatoid nodule, left elbow |
| Rheumatoid Arthritis or Collagen Vascular Diseases | M06.329 | Rheumatoid nodule, unspecified elbow |
| Rheumatoid Arthritis or Collagen Vascular Diseases | M06.331 | Rheumatoid nodule, right wrist |
| Rheumatoid Arthritis or Collagen Vascular Diseases | M06.332 | Rheumatoid nodule, left wrist |
| Rheumatoid Arthritis or Collagen Vascular Diseases | M06.339 | Rheumatoid nodule, unspecified wrist |
| Rheumatoid Arthritis or Collagen Vascular Diseases | M06.341 | Rheumatoid nodule, right hand |
| Rheumatoid Arthritis or Collagen Vascular Diseases | M06.342 | Rheumatoid nodule, left hand |
| Rheumatoid Arthritis or Collagen Vascular Diseases | M06.349 | Rheumatoid nodule, unspecified hand |
| Rheumatoid Arthritis or Collagen Vascular Diseases | M06.351 | Rheumatoid nodule, right hip |
| Rheumatoid Arthritis or Collagen Vascular Diseases | M06.352 | Rheumatoid nodule, left hip |
| Rheumatoid Arthritis or Collagen Vascular Diseases | M06.359 | Rheumatoid nodule, unspecified hip |
| Rheumatoid Arthritis or Collagen Vascular Diseases | M06.361 | Rheumatoid nodule, right knee |
| Rheumatoid Arthritis or Collagen Vascular Diseases | M06.362 | Rheumatoid nodule, left knee |
| Rheumatoid Arthritis or Collagen Vascular Diseases | M06.369 | Rheumatoid nodule, unspecified knee |
| Rheumatoid Arthritis or Collagen Vascular Diseases | M06.371 | Rheumatoid nodule, right ankle and foot |
| Rheumatoid Arthritis or Collagen Vascular Diseases | M06.372 | Rheumatoid nodule, left ankle and foot |
| Rheumatoid Arthritis or Collagen Vascular Diseases | M06.379 | Rheumatoid nodule, unspecified ankle and foot |
| Rheumatoid Arthritis or Collagen Vascular Diseases | M06.38 | Rheumatoid nodule, vertebrae |
| Rheumatoid Arthritis or Collagen Vascular Diseases | M06.39 | Rheumatoid nodule, multiple sites |
| Rheumatoid Arthritis or Collagen Vascular Diseases | M06.4 | Inflammatory polyarthropathy |
| Rheumatoid Arthritis or Collagen Vascular Diseases | M06.80 | Other specified rheumatoid arthritis, unspecified site |
| Rheumatoid Arthritis or Collagen Vascular Diseases | M06.811 | Other specified rheumatoid arthritis, right shoulder |
| Rheumatoid Arthritis or Collagen Vascular Diseases | M06.812 | Other specified rheumatoid arthritis, left shoulder |
| Rheumatoid Arthritis or Collagen Vascular Diseases | M06.819 | Other specified rheumatoid arthritis, unspecified shoulder |
| Rheumatoid Arthritis or Collagen Vascular Diseases | M06.821 | Other specified rheumatoid arthritis, right elbow |
| Rheumatoid Arthritis or Collagen Vascular Diseases | M06.822 | Other specified rheumatoid arthritis, left elbow |
| Rheumatoid Arthritis or Collagen Vascular Diseases | M06.829 | Other specified rheumatoid arthritis, unspecified elbow |
| Rheumatoid Arthritis or Collagen Vascular Diseases | M06.831 | Other specified rheumatoid arthritis, right wrist |
| Rheumatoid Arthritis or Collagen Vascular Diseases | M06.832 | Other specified rheumatoid arthritis, left wrist |
| Rheumatoid Arthritis or Collagen Vascular Diseases | M06.839 | Other specified rheumatoid arthritis, unspecified wrist |
| Rheumatoid Arthritis or Collagen Vascular Diseases | M06.841 | Other specified rheumatoid arthritis, right hand |
| Rheumatoid Arthritis or Collagen Vascular Diseases | M06.842 | Other specified rheumatoid arthritis, left hand |
| Rheumatoid Arthritis or Collagen Vascular Diseases | M06.849 | Other specified rheumatoid arthritis, unspecified hand |
| Rheumatoid Arthritis or Collagen Vascular Diseases | M06.851 | Other specified rheumatoid arthritis, right hip |
| Rheumatoid Arthritis or Collagen Vascular Diseases | M06.852 | Other specified rheumatoid arthritis, left hip |
| Rheumatoid Arthritis or Collagen Vascular Diseases | M06.859 | Other specified rheumatoid arthritis, unspecified hip |
| Rheumatoid Arthritis or Collagen Vascular Diseases | M06.861 | Other specified rheumatoid arthritis, right knee |
| Rheumatoid Arthritis or Collagen Vascular Diseases | M06.862 | Other specified rheumatoid arthritis, left knee |
| Rheumatoid Arthritis or Collagen Vascular Diseases | M06.869 | Other specified rheumatoid arthritis, unspecified knee |
| Rheumatoid Arthritis or Collagen Vascular Diseases | M06.871 | Other specified rheumatoid arthritis, right ankle and foot |
| Rheumatoid Arthritis or Collagen Vascular Diseases | M06.872 | Other specified rheumatoid arthritis, left ankle and foot |
| Rheumatoid Arthritis or Collagen Vascular Diseases | M06.879 | Other specified rheumatoid arthritis, unspecified ankle and foot |
| Rheumatoid Arthritis or Collagen Vascular Diseases | M06.88 | Other specified rheumatoid arthritis, vertebrae |
| Rheumatoid Arthritis or Collagen Vascular Diseases | M06.89 | Other specified rheumatoid arthritis, multiple sites |
| Rheumatoid Arthritis or Collagen Vascular Diseases | M06.9 | Rheumatoid arthritis, unspecified |
| Rheumatoid Arthritis or Collagen Vascular Diseases | M08.00 | Unspecified juvenile rheumatoid arthritis of unspecified site |
| Rheumatoid Arthritis or Collagen Vascular Diseases | M08.011 | Unspecified juvenile rheumatoid arthritis, right shoulder |
| Rheumatoid Arthritis or Collagen Vascular Diseases | M08.012 | Unspecified juvenile rheumatoid arthritis, left shoulder |
| Rheumatoid Arthritis or Collagen Vascular Diseases | M08.019 | Unspecified juvenile rheumatoid arthritis, unspecified shoulder |
| Rheumatoid Arthritis or Collagen Vascular Diseases | M08.021 | Unspecified juvenile rheumatoid arthritis, right elbow |
| Rheumatoid Arthritis or Collagen Vascular Diseases | M08.022 | Unspecified juvenile rheumatoid arthritis, left elbow |
| Rheumatoid Arthritis or Collagen Vascular Diseases | M08.029 | Unspecified juvenile rheumatoid arthritis, unspecified elbow |
| Rheumatoid Arthritis or Collagen Vascular Diseases | M08.031 | Unspecified juvenile rheumatoid arthritis, right wrist |
| Rheumatoid Arthritis or Collagen Vascular Diseases | M08.032 | Unspecified juvenile rheumatoid arthritis, left wrist |
| Rheumatoid Arthritis or Collagen Vascular Diseases | M08.039 | Unspecified juvenile rheumatoid arthritis, unspecified wrist |
| Rheumatoid Arthritis or Collagen Vascular Diseases | M08.041 | Unspecified juvenile rheumatoid arthritis, right hand |
| Rheumatoid Arthritis or Collagen Vascular Diseases | M08.042 | Unspecified juvenile rheumatoid arthritis, left hand |
| Rheumatoid Arthritis or Collagen Vascular Diseases | M08.049 | Unspecified juvenile rheumatoid arthritis, unspecified hand |
| Rheumatoid Arthritis or Collagen Vascular Diseases | M08.051 | Unspecified juvenile rheumatoid arthritis, right hip |
| Rheumatoid Arthritis or Collagen Vascular Diseases | M08.052 | Unspecified juvenile rheumatoid arthritis, left hip |
| Rheumatoid Arthritis or Collagen Vascular Diseases | M08.059 | Unspecified juvenile rheumatoid arthritis, unspecified hip |
| Rheumatoid Arthritis or Collagen Vascular Diseases | M08.061 | Unspecified juvenile rheumatoid arthritis, right knee |
| Rheumatoid Arthritis or Collagen Vascular Diseases | M08.062 | Unspecified juvenile rheumatoid arthritis, left knee |
| Rheumatoid Arthritis or Collagen Vascular Diseases | M08.069 | Unspecified juvenile rheumatoid arthritis, unspecified knee |
| Rheumatoid Arthritis or Collagen Vascular Diseases | M08.071 | Unspecified juvenile rheumatoid arthritis, right ankle and foot |
| Rheumatoid Arthritis or Collagen Vascular Diseases | M08.072 | Unspecified juvenile rheumatoid arthritis, left ankle and foot |
| Rheumatoid Arthritis or Collagen Vascular Diseases | M08.079 | Unspecified juvenile rheumatoid arthritis, unspecified ankle and foot |
| Rheumatoid Arthritis or Collagen Vascular Diseases | M08.08 | Unspecified juvenile rheumatoid arthritis, vertebrae |
| Rheumatoid Arthritis or Collagen Vascular Diseases | M08.09 | Unspecified juvenile rheumatoid arthritis, multiple sites |
| Rheumatoid Arthritis or Collagen Vascular Diseases | M08.1 | Juvenile ankylosing spondylitis |
| Rheumatoid Arthritis or Collagen Vascular Diseases | M08.20 | Juvenile rheumatoid arthritis with systemic onset, unspecified site |
| Rheumatoid Arthritis or Collagen Vascular Diseases | M08.211 | Juvenile rheumatoid arthritis with systemic onset, right shoulder |
| Rheumatoid Arthritis or Collagen Vascular Diseases | M08.212 | Juvenile rheumatoid arthritis with systemic onset, left shoulder |
| Rheumatoid Arthritis or Collagen Vascular Diseases | M08.219 | Juvenile rheumatoid arthritis with systemic onset, unspecified shoulder |
| Rheumatoid Arthritis or Collagen Vascular Diseases | M08.221 | Juvenile rheumatoid arthritis with systemic onset, right elbow |
| Rheumatoid Arthritis or Collagen Vascular Diseases | M08.222 | Juvenile rheumatoid arthritis with systemic onset, left elbow |
| Rheumatoid Arthritis or Collagen Vascular Diseases | M08.229 | Juvenile rheumatoid arthritis with systemic onset, unspecified elbow |
| Rheumatoid Arthritis or Collagen Vascular Diseases | M08.231 | Juvenile rheumatoid arthritis with systemic onset, right wrist |
| Rheumatoid Arthritis or Collagen Vascular Diseases | M08.232 | Juvenile rheumatoid arthritis with systemic onset, left wrist |
| Rheumatoid Arthritis or Collagen Vascular Diseases | M08.239 | Juvenile rheumatoid arthritis with systemic onset, unspecified wrist |
| Rheumatoid Arthritis or Collagen Vascular Diseases | M08.241 | Juvenile rheumatoid arthritis with systemic onset, right hand |
| Rheumatoid Arthritis or Collagen Vascular Diseases | M08.242 | Juvenile rheumatoid arthritis with systemic onset, left hand |
| Rheumatoid Arthritis or Collagen Vascular Diseases | M08.249 | Juvenile rheumatoid arthritis with systemic onset, unspecified hand |
| Rheumatoid Arthritis or Collagen Vascular Diseases | M08.251 | Juvenile rheumatoid arthritis with systemic onset, right hip |
| Rheumatoid Arthritis or Collagen Vascular Diseases | M08.252 | Juvenile rheumatoid arthritis with systemic onset, left hip |
| Rheumatoid Arthritis or Collagen Vascular Diseases | M08.259 | Juvenile rheumatoid arthritis with systemic onset, unspecified hip |
| Rheumatoid Arthritis or Collagen Vascular Diseases | M08.261 | Juvenile rheumatoid arthritis with systemic onset, right knee |
| Rheumatoid Arthritis or Collagen Vascular Diseases | M08.262 | Juvenile rheumatoid arthritis with systemic onset, left knee |
| Rheumatoid Arthritis or Collagen Vascular Diseases | M08.269 | Juvenile rheumatoid arthritis with systemic onset, unspecified knee |
| Rheumatoid Arthritis or Collagen Vascular Diseases | M08.271 | Juvenile rheumatoid arthritis with systemic onset, right ankle and foot |
| Rheumatoid Arthritis or Collagen Vascular Diseases | M08.272 | Juvenile rheumatoid arthritis with systemic onset, left ankle and foot |
| Rheumatoid Arthritis or Collagen Vascular Diseases | M08.279 | Juvenile rheumatoid arthritis with systemic onset, unspecified ankle and foot |
| Rheumatoid Arthritis or Collagen Vascular Diseases | M08.28 | Juvenile rheumatoid arthritis with systemic onset, vertebrae |
| Rheumatoid Arthritis or Collagen Vascular Diseases | M08.29 | Juvenile rheumatoid arthritis with systemic onset, multiple sites |
| Rheumatoid Arthritis or Collagen Vascular Diseases | M08.3 | Juvenile rheumatoid polyarthritis (seronegative) |
| Rheumatoid Arthritis or Collagen Vascular Diseases | M08.40 | Pauciarticular juvenile rheumatoid arthritis, unspecified site |
| Rheumatoid Arthritis or Collagen Vascular Diseases | M08.411 | Pauciarticular juvenile rheumatoid arthritis, right shoulder |
| Rheumatoid Arthritis or Collagen Vascular Diseases | M08.412 | Pauciarticular juvenile rheumatoid arthritis, left shoulder |
| Rheumatoid Arthritis or Collagen Vascular Diseases | M08.419 | Pauciarticular juvenile rheumatoid arthritis, unspecified shoulder |
| Rheumatoid Arthritis or Collagen Vascular Diseases | M08.421 | Pauciarticular juvenile rheumatoid arthritis, right elbow |
| Rheumatoid Arthritis or Collagen Vascular Diseases | M08.422 | Pauciarticular juvenile rheumatoid arthritis, left elbow |
| Rheumatoid Arthritis or Collagen Vascular Diseases | M08.429 | Pauciarticular juvenile rheumatoid arthritis, unspecified elbow |
| Rheumatoid Arthritis or Collagen Vascular Diseases | M08.431 | Pauciarticular juvenile rheumatoid arthritis, right wrist |
| Rheumatoid Arthritis or Collagen Vascular Diseases | M08.432 | Pauciarticular juvenile rheumatoid arthritis, left wrist |
| Rheumatoid Arthritis or Collagen Vascular Diseases | M08.439 | Pauciarticular juvenile rheumatoid arthritis, unspecified wrist |
| Rheumatoid Arthritis or Collagen Vascular Diseases | M08.441 | Pauciarticular juvenile rheumatoid arthritis, right hand |
| Rheumatoid Arthritis or Collagen Vascular Diseases | M08.442 | Pauciarticular juvenile rheumatoid arthritis, left hand |
| Rheumatoid Arthritis or Collagen Vascular Diseases | M08.449 | Pauciarticular juvenile rheumatoid arthritis, unspecified hand |
| Rheumatoid Arthritis or Collagen Vascular Diseases | M08.451 | Pauciarticular juvenile rheumatoid arthritis, right hip |
| Rheumatoid Arthritis or Collagen Vascular Diseases | M08.452 | Pauciarticular juvenile rheumatoid arthritis, left hip |
| Rheumatoid Arthritis or Collagen Vascular Diseases | M08.459 | Pauciarticular juvenile rheumatoid arthritis, unspecified hip |
| Rheumatoid Arthritis or Collagen Vascular Diseases | M08.461 | Pauciarticular juvenile rheumatoid arthritis, right knee |
| Rheumatoid Arthritis or Collagen Vascular Diseases | M08.462 | Pauciarticular juvenile rheumatoid arthritis, left knee |
| Rheumatoid Arthritis or Collagen Vascular Diseases | M08.469 | Pauciarticular juvenile rheumatoid arthritis, unspecified knee |
| Rheumatoid Arthritis or Collagen Vascular Diseases | M08.471 | Pauciarticular juvenile rheumatoid arthritis, right ankle and foot |
| Rheumatoid Arthritis or Collagen Vascular Diseases | M08.472 | Pauciarticular juvenile rheumatoid arthritis, left ankle and foot |
| Rheumatoid Arthritis or Collagen Vascular Diseases | M08.479 | Pauciarticular juvenile rheumatoid arthritis, unspecified ankle and foot |
| Rheumatoid Arthritis or Collagen Vascular Diseases | M08.48 | Pauciarticular juvenile rheumatoid arthritis, vertebrae |
| Rheumatoid Arthritis or Collagen Vascular Diseases | M08.80 | Other juvenile arthritis, unspecified site |
| Rheumatoid Arthritis or Collagen Vascular Diseases | M08.811 | Other juvenile arthritis, right shoulder |
| Rheumatoid Arthritis or Collagen Vascular Diseases | M08.812 | Other juvenile arthritis, left shoulder |
| Rheumatoid Arthritis or Collagen Vascular Diseases | M08.819 | Other juvenile arthritis, unspecified shoulder |
| Rheumatoid Arthritis or Collagen Vascular Diseases | M08.821 | Other juvenile arthritis, right elbow |
| Rheumatoid Arthritis or Collagen Vascular Diseases | M08.822 | Other juvenile arthritis, left elbow |
| Rheumatoid Arthritis or Collagen Vascular Diseases | M08.829 | Other juvenile arthritis, unspecified elbow |
| Rheumatoid Arthritis or Collagen Vascular Diseases | M08.831 | Other juvenile arthritis, right wrist |
| Rheumatoid Arthritis or Collagen Vascular Diseases | M08.832 | Other juvenile arthritis, left wrist |
| Rheumatoid Arthritis or Collagen Vascular Diseases | M08.839 | Other juvenile arthritis, unspecified wrist |
| Rheumatoid Arthritis or Collagen Vascular Diseases | M08.841 | Other juvenile arthritis, right hand |
| Rheumatoid Arthritis or Collagen Vascular Diseases | M08.842 | Other juvenile arthritis, left hand |
| Rheumatoid Arthritis or Collagen Vascular Diseases | M08.849 | Other juvenile arthritis, unspecified hand |
| Rheumatoid Arthritis or Collagen Vascular Diseases | M08.851 | Other juvenile arthritis, right hip |
| Rheumatoid Arthritis or Collagen Vascular Diseases | M08.852 | Other juvenile arthritis, left hip |
| Rheumatoid Arthritis or Collagen Vascular Diseases | M08.859 | Other juvenile arthritis, unspecified hip |
| Rheumatoid Arthritis or Collagen Vascular Diseases | M08.861 | Other juvenile arthritis, right knee |
| Rheumatoid Arthritis or Collagen Vascular Diseases | M08.862 | Other juvenile arthritis, left knee |
| Rheumatoid Arthritis or Collagen Vascular Diseases | M08.869 | Other juvenile arthritis, unspecified knee |
| Rheumatoid Arthritis or Collagen Vascular Diseases | M08.871 | Other juvenile arthritis, right ankle and foot |
| Rheumatoid Arthritis or Collagen Vascular Diseases | M08.872 | Other juvenile arthritis, left ankle and foot |
| Rheumatoid Arthritis or Collagen Vascular Diseases | M08.879 | Other juvenile arthritis, unspecified ankle and foot |
| Rheumatoid Arthritis or Collagen Vascular Diseases | M08.88 | Other juvenile arthritis, other specified site |
| Rheumatoid Arthritis or Collagen Vascular Diseases | M08.89 | Other juvenile arthritis, multiple sites |
| Rheumatoid Arthritis or Collagen Vascular Diseases | M08.90 | Juvenile arthritis, unspecified, unspecified site |
| Rheumatoid Arthritis or Collagen Vascular Diseases | M08.911 | Juvenile arthritis, unspecified, right shoulder |
| Rheumatoid Arthritis or Collagen Vascular Diseases | M08.912 | Juvenile arthritis, unspecified, left shoulder |
| Rheumatoid Arthritis or Collagen Vascular Diseases | M08.919 | Juvenile arthritis, unspecified, unspecified shoulder |
| Rheumatoid Arthritis or Collagen Vascular Diseases | M08.921 | Juvenile arthritis, unspecified, right elbow |
| Rheumatoid Arthritis or Collagen Vascular Diseases | M08.922 | Juvenile arthritis, unspecified, left elbow |
| Rheumatoid Arthritis or Collagen Vascular Diseases | M08.929 | Juvenile arthritis, unspecified, unspecified elbow |
| Rheumatoid Arthritis or Collagen Vascular Diseases | M08.931 | Juvenile arthritis, unspecified, right wrist |
| Rheumatoid Arthritis or Collagen Vascular Diseases | M08.932 | Juvenile arthritis, unspecified, left wrist |
| Rheumatoid Arthritis or Collagen Vascular Diseases | M08.939 | Juvenile arthritis, unspecified, unspecified wrist |
| Rheumatoid Arthritis or Collagen Vascular Diseases | M08.941 | Juvenile arthritis, unspecified, right hand |
| Rheumatoid Arthritis or Collagen Vascular Diseases | M08.942 | Juvenile arthritis, unspecified, left hand |
| Rheumatoid Arthritis or Collagen Vascular Diseases | M08.949 | Juvenile arthritis, unspecified, unspecified hand |
| Rheumatoid Arthritis or Collagen Vascular Diseases | M08.951 | Juvenile arthritis, unspecified, right hip |
| Rheumatoid Arthritis or Collagen Vascular Diseases | M08.952 | Juvenile arthritis, unspecified, left hip |
| Rheumatoid Arthritis or Collagen Vascular Diseases | M08.959 | Juvenile arthritis, unspecified, unspecified hip |
| Rheumatoid Arthritis or Collagen Vascular Diseases | M08.961 | Juvenile arthritis, unspecified, right knee |
| Rheumatoid Arthritis or Collagen Vascular Diseases | M08.962 | Juvenile arthritis, unspecified, left knee |
| Rheumatoid Arthritis or Collagen Vascular Diseases | M08.969 | Juvenile arthritis, unspecified, unspecified knee |
| Rheumatoid Arthritis or Collagen Vascular Diseases | M08.971 | Juvenile arthritis, unspecified, right ankle and foot |
| Rheumatoid Arthritis or Collagen Vascular Diseases | M08.972 | Juvenile arthritis, unspecified, left ankle and foot |
| Rheumatoid Arthritis or Collagen Vascular Diseases | M08.979 | Juvenile arthritis, unspecified, unspecified ankle and foot |
| Rheumatoid Arthritis or Collagen Vascular Diseases | M08.98 | Juvenile arthritis, unspecified, vertebrae |
| Rheumatoid Arthritis or Collagen Vascular Diseases | M08.99 | Juvenile arthritis, unspecified, multiple sites |
| Rheumatoid Arthritis or Collagen Vascular Diseases | M12.00 | Chronic postrheumatic arthropathy [Jaccoud], unspecified site |
| Rheumatoid Arthritis or Collagen Vascular Diseases | M12.011 | Chronic postrheumatic arthropathy [Jaccoud], right shoulder |
| Rheumatoid Arthritis or Collagen Vascular Diseases | M12.012 | Chronic postrheumatic arthropathy [Jaccoud], left shoulder |
| Rheumatoid Arthritis or Collagen Vascular Diseases | M12.019 | Chronic postrheumatic arthropathy [Jaccoud], unspecified shoulder |
| Rheumatoid Arthritis or Collagen Vascular Diseases | M12.021 | Chronic postrheumatic arthropathy [Jaccoud], right elbow |
| Rheumatoid Arthritis or Collagen Vascular Diseases | M12.022 | Chronic postrheumatic arthropathy [Jaccoud], left elbow |
| Rheumatoid Arthritis or Collagen Vascular Diseases | M12.029 | Chronic postrheumatic arthropathy [Jaccoud], unspecified elbow |
| Rheumatoid Arthritis or Collagen Vascular Diseases | M12.031 | Chronic postrheumatic arthropathy [Jaccoud], right wrist |
| Rheumatoid Arthritis or Collagen Vascular Diseases | M12.032 | Chronic postrheumatic arthropathy [Jaccoud], left wrist |
| Rheumatoid Arthritis or Collagen Vascular Diseases | M12.039 | Chronic postrheumatic arthropathy [Jaccoud], unspecified wrist |
| Rheumatoid Arthritis or Collagen Vascular Diseases | M12.041 | Chronic postrheumatic arthropathy [Jaccoud], right hand |
| Rheumatoid Arthritis or Collagen Vascular Diseases | M12.042 | Chronic postrheumatic arthropathy [Jaccoud], left hand |
| Rheumatoid Arthritis or Collagen Vascular Diseases | M12.049 | Chronic postrheumatic arthropathy [Jaccoud], unspecified hand |
| Rheumatoid Arthritis or Collagen Vascular Diseases | M12.051 | Chronic postrheumatic arthropathy [Jaccoud], right hip |
| Rheumatoid Arthritis or Collagen Vascular Diseases | M12.052 | Chronic postrheumatic arthropathy [Jaccoud], left hip |
| Rheumatoid Arthritis or Collagen Vascular Diseases | M12.059 | Chronic postrheumatic arthropathy [Jaccoud], unspecified hip |
| Rheumatoid Arthritis or Collagen Vascular Diseases | M12.061 | Chronic postrheumatic arthropathy [Jaccoud], right knee |
| Rheumatoid Arthritis or Collagen Vascular Diseases | M12.062 | Chronic postrheumatic arthropathy [Jaccoud], left knee |
| Rheumatoid Arthritis or Collagen Vascular Diseases | M12.069 | Chronic postrheumatic arthropathy [Jaccoud], unspecified knee |
| Rheumatoid Arthritis or Collagen Vascular Diseases | M12.071 | Chronic postrheumatic arthropathy [Jaccoud], right ankle and foot |
| Rheumatoid Arthritis or Collagen Vascular Diseases | M12.072 | Chronic postrheumatic arthropathy [Jaccoud], left ankle and foot |
| Rheumatoid Arthritis or Collagen Vascular Diseases | M12.079 | Chronic postrheumatic arthropathy [Jaccoud], unspecified ankle and foot |
| Rheumatoid Arthritis or Collagen Vascular Diseases | M12.08 | Chronic postrheumatic arthropathy [Jaccoud], other specified site |
| Rheumatoid Arthritis or Collagen Vascular Diseases | M12.09 | Chronic postrheumatic arthropathy [Jaccoud], multiple sites |
| Rheumatoid Arthritis or Collagen Vascular Diseases | M12.30 | Palindromic rheumatism, unspecified site |
| Rheumatoid Arthritis or Collagen Vascular Diseases | M12.311 | Palindromic rheumatism, right shoulder |
| Rheumatoid Arthritis or Collagen Vascular Diseases | M12.312 | Palindromic rheumatism, left shoulder |
| Rheumatoid Arthritis or Collagen Vascular Diseases | M12.319 | Palindromic rheumatism, unspecified shoulder |
| Rheumatoid Arthritis or Collagen Vascular Diseases | M12.321 | Palindromic rheumatism, right elbow |
| Rheumatoid Arthritis or Collagen Vascular Diseases | M12.322 | Palindromic rheumatism, left elbow |
| Rheumatoid Arthritis or Collagen Vascular Diseases | M12.329 | Palindromic rheumatism, unspecified elbow |
| Rheumatoid Arthritis or Collagen Vascular Diseases | M12.331 | Palindromic rheumatism, right wrist |
| Rheumatoid Arthritis or Collagen Vascular Diseases | M12.332 | Palindromic rheumatism, left wrist |
| Rheumatoid Arthritis or Collagen Vascular Diseases | M12.339 | Palindromic rheumatism, unspecified wrist |
| Rheumatoid Arthritis or Collagen Vascular Diseases | M12.341 | Palindromic rheumatism, right hand |
| Rheumatoid Arthritis or Collagen Vascular Diseases | M12.342 | Palindromic rheumatism, left hand |
| Rheumatoid Arthritis or Collagen Vascular Diseases | M12.349 | Palindromic rheumatism, unspecified hand |
| Rheumatoid Arthritis or Collagen Vascular Diseases | M12.351 | Palindromic rheumatism, right hip |
| Rheumatoid Arthritis or Collagen Vascular Diseases | M12.352 | Palindromic rheumatism, left hip |
| Rheumatoid Arthritis or Collagen Vascular Diseases | M12.359 | Palindromic rheumatism, unspecified hip |
| Rheumatoid Arthritis or Collagen Vascular Diseases | M12.361 | Palindromic rheumatism, right knee |
| Rheumatoid Arthritis or Collagen Vascular Diseases | M12.362 | Palindromic rheumatism, left knee |
| Rheumatoid Arthritis or Collagen Vascular Diseases | M12.369 | Palindromic rheumatism, unspecified knee |
| Rheumatoid Arthritis or Collagen Vascular Diseases | M12.371 | Palindromic rheumatism, right ankle and foot |
| Rheumatoid Arthritis or Collagen Vascular Diseases | M12.372 | Palindromic rheumatism, left ankle and foot |
| Rheumatoid Arthritis or Collagen Vascular Diseases | M12.379 | Palindromic rheumatism, unspecified ankle and foot |
| Rheumatoid Arthritis or Collagen Vascular Diseases | M12.38 | Palindromic rheumatism, other specified site |
| Rheumatoid Arthritis or Collagen Vascular Diseases | M12.39 | Palindromic rheumatism, multiple sites |
| Rheumatoid Arthritis or Collagen Vascular Diseases | M30.0 | Polyarteritis nodosa |
| Rheumatoid Arthritis or Collagen Vascular Diseases | M30.1 | Polyarteritis with lung involvement [Churg-Strauss] |
| Rheumatoid Arthritis or Collagen Vascular Diseases | M30.2 | Juvenile polyarteritis |
| Rheumatoid Arthritis or Collagen Vascular Diseases | M30.3 | Mucocutaneous lymph node syndrome [Kawasaki] |
| Rheumatoid Arthritis or Collagen Vascular Diseases | M30.8 | Other conditions related to polyarteritis nodosa |
| Rheumatoid Arthritis or Collagen Vascular Diseases | M31.0 | Hypersensitivity angiitis |
| Rheumatoid Arthritis or Collagen Vascular Diseases | M31.1 | Thrombotic microangiopathy |
| Rheumatoid Arthritis or Collagen Vascular Diseases | M31.2 | Lethal midline granuloma |
| Rheumatoid Arthritis or Collagen Vascular Diseases | M31.30 | Wegener's granulomatosis without renal involvement |
| Rheumatoid Arthritis or Collagen Vascular Diseases | M31.31 | Wegener's granulomatosis with renal involvement |
| Rheumatoid Arthritis or Collagen Vascular Diseases | M32.0 | Drug-induced systemic lupus erythematosus |
| Rheumatoid Arthritis or Collagen Vascular Diseases | M32.10 | Systemic lupus erythematosus, organ or system involvement unspecified |
| Rheumatoid Arthritis or Collagen Vascular Diseases | M32.11 | Endocarditis in systemic lupus erythematosus |
| Rheumatoid Arthritis or Collagen Vascular Diseases | M32.12 | Pericarditis in systemic lupus erythematosus |
| Rheumatoid Arthritis or Collagen Vascular Diseases | M32.13 | Lung involvement in systemic lupus erythematosus |
| Rheumatoid Arthritis or Collagen Vascular Diseases | M32.14 | Glomerular disease in systemic lupus erythematosus |
| Rheumatoid Arthritis or Collagen Vascular Diseases | M32.15 | Tubulo-interstitial nephropathy in systemic lupus erythematosus |
| Rheumatoid Arthritis or Collagen Vascular Diseases | M32.19 | Other organ or system involvement in systemic lupus erythematosus |
| Rheumatoid Arthritis or Collagen Vascular Diseases | M32.8 | Other forms of systemic lupus erythematosus |
| Rheumatoid Arthritis or Collagen Vascular Diseases | M32.9 | Systemic lupus erythematosus, unspecified |
| Rheumatoid Arthritis or Collagen Vascular Diseases | M33.00 | Juvenile dermatomyositis, organ involvement unspecified |
| Rheumatoid Arthritis or Collagen Vascular Diseases | M33.01 | Juvenile dermatomyositis with respiratory involvement |
| Rheumatoid Arthritis or Collagen Vascular Diseases | M33.02 | Juvenile dermatomyositis with myopathy |
| Rheumatoid Arthritis or Collagen Vascular Diseases | M33.09 | Juvenile dermatomyositis with other organ involvement |
| Rheumatoid Arthritis or Collagen Vascular Diseases | M33.10 | Other dermatomyositis, organ involvement unspecified |
| Rheumatoid Arthritis or Collagen Vascular Diseases | M33.11 | Other dermatomyositis with respiratory involvement |
| Rheumatoid Arthritis or Collagen Vascular Diseases | M33.12 | Other dermatomyositis with myopathy |
| Rheumatoid Arthritis or Collagen Vascular Diseases | M33.19 | Other dermatomyositis with other organ involvement |
| Rheumatoid Arthritis or Collagen Vascular Diseases | M33.20 | Polymyositis, organ involvement unspecified |
| Rheumatoid Arthritis or Collagen Vascular Diseases | M33.21 | Polymyositis with respiratory involvement |
| Rheumatoid Arthritis or Collagen Vascular Diseases | M33.22 | Polymyositis with myopathy |
| Rheumatoid Arthritis or Collagen Vascular Diseases | M33.29 | Polymyositis with other organ involvement |
| Rheumatoid Arthritis or Collagen Vascular Diseases | M33.90 | Dermatopolymyositis, unspecified, organ involvement unspecified |
| Rheumatoid Arthritis or Collagen Vascular Diseases | M33.91 | Dermatopolymyositis, unspecified with respiratory involvement |
| Rheumatoid Arthritis or Collagen Vascular Diseases | M33.92 | Dermatopolymyositis, unspecified with myopathy |
| Rheumatoid Arthritis or Collagen Vascular Diseases | M33.99 | Dermatopolymyositis, unspecified with other organ involvement |
| Rheumatoid Arthritis or Collagen Vascular Diseases | M34.0 | Progressive systemic sclerosis |
| Rheumatoid Arthritis or Collagen Vascular Diseases | M34.1 | CR(E)ST syndrome |
| Rheumatoid Arthritis or Collagen Vascular Diseases | M34.2 | Systemic sclerosis induced by drug and chemical |
| Rheumatoid Arthritis or Collagen Vascular Diseases | M34.81 | Systemic sclerosis with lung involvement |
| Rheumatoid Arthritis or Collagen Vascular Diseases | M34.82 | Systemic sclerosis with myopathy |
| Rheumatoid Arthritis or Collagen Vascular Diseases | M34.83 | Systemic sclerosis with polyneuropathy |
| Rheumatoid Arthritis or Collagen Vascular Diseases | M34.89 | Other systemic sclerosis |
| Rheumatoid Arthritis or Collagen Vascular Diseases | M34.9 | Systemic sclerosis, unspecified |
| Rheumatoid Arthritis or Collagen Vascular Diseases | M35.00 | Sjögren syndrome, unspecified |
| Rheumatoid Arthritis or Collagen Vascular Diseases | M35.01 | Sjögren syndrome with keratoconjunctivitis |
| Rheumatoid Arthritis or Collagen Vascular Diseases | M35.02 | Sjögren syndrome with lung involvement |
| Rheumatoid Arthritis or Collagen Vascular Diseases | M35.03 | Sjögren syndrome with myopathy |
| Rheumatoid Arthritis or Collagen Vascular Diseases | M35.04 | Sjögren syndrome with tubulo-interstitial nephropathy |
| Rheumatoid Arthritis or Collagen Vascular Diseases | M35.09 | Sjögren syndrome with other organ involvement |
| Rheumatoid Arthritis or Collagen Vascular Diseases | M35.1 | Other overlap syndromes |
| Rheumatoid Arthritis or Collagen Vascular Diseases | M35.2 | Behçet's disease |
| Rheumatoid Arthritis or Collagen Vascular Diseases | M35.3 | Polymyalgia rheumatica |
| Rheumatoid Arthritis or Collagen Vascular Diseases | M35.4 | Diffuse (eosinophilic) fasciitis |
| Rheumatoid Arthritis or Collagen Vascular Diseases | M35.5 | Multifocal fibrosclerosis |
| Rheumatoid Arthritis or Collagen Vascular Diseases | M35.6 | Relapsing panniculitis [Weber-Christian] |
| Rheumatoid Arthritis or Collagen Vascular Diseases | M35.7 | Hypermobility syndrome |
| Rheumatoid Arthritis or Collagen Vascular Diseases | M35.8 | Other specified systemic involvement of connective tissue |
| Rheumatoid Arthritis or Collagen Vascular Diseases | M35.9 | Systemic involvement of connective tissue, unspecified |
| Rheumatoid Arthritis or Collagen Vascular Diseases | M45.0 | Ankylosing spondylitis of multiple sites in spine |
| Rheumatoid Arthritis or Collagen Vascular Diseases | M45.1 | Ankylosing spondylitis of occipito-atlanto-axial region |
| Rheumatoid Arthritis or Collagen Vascular Diseases | M45.2 | Ankylosing spondylitis of cervical region |
| Rheumatoid Arthritis or Collagen Vascular Diseases | M45.3 | Ankylosing spondylitis of cervicothoracic region |
| Rheumatoid Arthritis or Collagen Vascular Diseases | M45.4 | Ankylosing spondylitis of thoracic region |
| Rheumatoid Arthritis or Collagen Vascular Diseases | M45.5 | Ankylosing spondylitis of thoracolumbar region |
| Rheumatoid Arthritis or Collagen Vascular Diseases | M45.6 | Ankylosing spondylitis lumbar region |
| Rheumatoid Arthritis or Collagen Vascular Diseases | M45.7 | Ankylosing spondylitis of lumbosacral region |
| Rheumatoid Arthritis or Collagen Vascular Diseases | M45.8 | Ankylosing spondylitis sacral and sacrococcygeal region |
| Rheumatoid Arthritis or Collagen Vascular Diseases | M45.9 | Ankylosing spondylitis of unspecified sites in spine |
| Rheumatoid Arthritis or Collagen Vascular Diseases | M46.1 | Sacroiliitis, not elsewhere classified |
| Rheumatoid Arthritis or Collagen Vascular Diseases | M46.80 | Other specified inflammatory spondylopathies, site unspecified |
| Rheumatoid Arthritis or Collagen Vascular Diseases | M46.81 | Other specified inflammatory spondylopathies, occipito-atlanto-axial region |
| Rheumatoid Arthritis or Collagen Vascular Diseases | M46.82 | Other specified inflammatory spondylopathies, cervical region |
| Rheumatoid Arthritis or Collagen Vascular Diseases | M46.83 | Other specified inflammatory spondylopathies, cervicothoracic region |
| Rheumatoid Arthritis or Collagen Vascular Diseases | M46.84 | Other specified inflammatory spondylopathies, thoracic region |
| Rheumatoid Arthritis or Collagen Vascular Diseases | M46.85 | Other specified inflammatory spondylopathies, thoracolumbar region |
| Rheumatoid Arthritis or Collagen Vascular Diseases | M46.86 | Other specified inflammatory spondylopathies, lumbar region |
| Rheumatoid Arthritis or Collagen Vascular Diseases | M46.87 | Other specified inflammatory spondylopathies, lumbosacral region |
| Rheumatoid Arthritis or Collagen Vascular Diseases | M46.88 | Other specified inflammatory spondylopathies, sacral and sacrococcygeal region |
| Rheumatoid Arthritis or Collagen Vascular Diseases | M46.89 | Other specified inflammatory spondylopathies, multiple sites in spine |
| Rheumatoid Arthritis or Collagen Vascular Diseases | M46.90 | Unspecified inflammatory spondylopathy, site unspecified |
| Rheumatoid Arthritis or Collagen Vascular Diseases | M46.91 | Unspecified inflammatory spondylopathy, occipito-atlanto-axial region |
| Rheumatoid Arthritis or Collagen Vascular Diseases | M46.92 | Unspecified inflammatory spondylopathy, cervical region |
| Rheumatoid Arthritis or Collagen Vascular Diseases | M46.93 | Unspecified inflammatory spondylopathy, cervicothoracic region |
| Rheumatoid Arthritis or Collagen Vascular Diseases | M46.94 | Unspecified inflammatory spondylopathy, thoracic region |
| Rheumatoid Arthritis or Collagen Vascular Diseases | M46.95 | Unspecified inflammatory spondylopathy, thoracolumbar region |
| Rheumatoid Arthritis or Collagen Vascular Diseases | M46.96 | Unspecified inflammatory spondylopathy, lumbar region |
| Rheumatoid Arthritis or Collagen Vascular Diseases | M46.97 | Unspecified inflammatory spondylopathy, lumbosacral region |
| Rheumatoid Arthritis or Collagen Vascular Diseases | M46.98 | Unspecified inflammatory spondylopathy, sacral and sacrococcygeal region |
| Rheumatoid Arthritis or Collagen Vascular Diseases | M46.99 | Unspecified inflammatory spondylopathy, multiple sites in spine |
| Solid Tumor Without Metastasis | C00.0 | Malignant neoplasm of external upper lip |
| Solid Tumor Without Metastasis | C00.1 | Malignant neoplasm of external lower lip |
| Solid Tumor Without Metastasis | C00.2 | Malignant neoplasm of external lip, unspecified |
| Solid Tumor Without Metastasis | C00.3 | Malignant neoplasm of upper lip, inner aspect |
| Solid Tumor Without Metastasis | C00.4 | Malignant neoplasm of lower lip, inner aspect |
| Solid Tumor Without Metastasis | C00.5 | Malignant neoplasm of lip, unspecified, inner aspect |
| Solid Tumor Without Metastasis | C00.6 | Malignant neoplasm of commissure of lip, unspecified |
| Solid Tumor Without Metastasis | C00.8 | Malignant neoplasm of overlapping sites of lip |
| Solid Tumor Without Metastasis | C00.9 | Malignant neoplasm of lip, unspecified |
| Solid Tumor Without Metastasis | C01 | Malignant neoplasm of base of tongue |
| Solid Tumor Without Metastasis | C02.0 | Malignant neoplasm of dorsal surface of tongue |
| Solid Tumor Without Metastasis | C02.1 | Malignant neoplasm of border of tongue |
| Solid Tumor Without Metastasis | C02.2 | Malignant neoplasm of ventral surface of tongue |
| Solid Tumor Without Metastasis | C02.3 | Malignant neoplasm of anterior two-thirds of tongue, part unspecified |
| Solid Tumor Without Metastasis | C02.4 | Malignant neoplasm of lingual tonsil |
| Solid Tumor Without Metastasis | C02.8 | Malignant neoplasm of overlapping sites of tongue |
| Solid Tumor Without Metastasis | C02.9 | Malignant neoplasm of tongue, unspecified |
| Solid Tumor Without Metastasis | C03.0 | Malignant neoplasm of upper gum |
| Solid Tumor Without Metastasis | C03.1 | Malignant neoplasm of lower gum |
| Solid Tumor Without Metastasis | C03.9 | Malignant neoplasm of gum, unspecified |
| Solid Tumor Without Metastasis | C04.0 | Malignant neoplasm of anterior floor of mouth |
| Solid Tumor Without Metastasis | C04.1 | Malignant neoplasm of lateral floor of mouth |
| Solid Tumor Without Metastasis | C04.8 | Malignant neoplasm of overlapping sites of floor of mouth |
| Solid Tumor Without Metastasis | C04.9 | Malignant neoplasm of floor of mouth, unspecified |
| Solid Tumor Without Metastasis | C05.0 | Malignant neoplasm of hard palate |
| Solid Tumor Without Metastasis | C05.1 | Malignant neoplasm of soft palate |
| Solid Tumor Without Metastasis | C05.2 | Malignant neoplasm of uvula |
| Solid Tumor Without Metastasis | C05.8 | Malignant neoplasm of overlapping sites of palate |
| Solid Tumor Without Metastasis | C05.9 | Malignant neoplasm of palate, unspecified |
| Solid Tumor Without Metastasis | C06.0 | Malignant neoplasm of cheek mucosa |
| Solid Tumor Without Metastasis | C06.1 | Malignant neoplasm of vestibule of mouth |
| Solid Tumor Without Metastasis | C06.2 | Malignant neoplasm of retromolar area |
| Solid Tumor Without Metastasis | C06.80 | Malignant neoplasm of overlapping sites of unspecified parts of mouth |
| Solid Tumor Without Metastasis | C06.89 | Malignant neoplasm of overlapping sites of other parts of mouth |
| Solid Tumor Without Metastasis | C06.9 | Malignant neoplasm of mouth, unspecified |
| Solid Tumor Without Metastasis | C07 | Malignant neoplasm of parotid gland |
| Solid Tumor Without Metastasis | C08.0 | Malignant neoplasm of submandibular gland |
| Solid Tumor Without Metastasis | C08.1 | Malignant neoplasm of sublingual gland |
| Solid Tumor Without Metastasis | C08.9 | Malignant neoplasm of major salivary gland, unspecified |
| Solid Tumor Without Metastasis | C09.0 | Malignant neoplasm of tonsillar fossa |
| Solid Tumor Without Metastasis | C09.1 | Malignant neoplasm of tonsillar pillar (anterior) (posterior) |
| Solid Tumor Without Metastasis | C09.8 | Malignant neoplasm of overlapping sites of tonsil |
| Solid Tumor Without Metastasis | C09.9 | Malignant neoplasm of tonsil, unspecified |
| Solid Tumor Without Metastasis | C10.0 | Malignant neoplasm of vallecula |
| Solid Tumor Without Metastasis | C10.1 | Malignant neoplasm of anterior surface of epiglottis |
| Solid Tumor Without Metastasis | C10.2 | Malignant neoplasm of lateral wall of oropharynx |
| Solid Tumor Without Metastasis | C10.3 | Malignant neoplasm of posterior wall of oropharynx |
| Solid Tumor Without Metastasis | C10.4 | Malignant neoplasm of branchial cleft |
| Solid Tumor Without Metastasis | C10.8 | Malignant neoplasm of overlapping sites of oropharynx |
| Solid Tumor Without Metastasis | C10.9 | Malignant neoplasm of oropharynx, unspecified |
| Solid Tumor Without Metastasis | C11.0 | Malignant neoplasm of superior wall of nasopharynx |
| Solid Tumor Without Metastasis | C11.1 | Malignant neoplasm of posterior wall of nasopharynx |
| Solid Tumor Without Metastasis | C11.2 | Malignant neoplasm of lateral wall of nasopharynx |
| Solid Tumor Without Metastasis | C11.3 | Malignant neoplasm of anterior wall of nasopharynx |
| Solid Tumor Without Metastasis | C11.8 | Malignant neoplasm of overlapping sites of nasopharynx |
| Solid Tumor Without Metastasis | C11.9 | Malignant neoplasm of nasopharynx, unspecified |
| Solid Tumor Without Metastasis | C12 | Malignant neoplasm of pyriform sinus |
| Solid Tumor Without Metastasis | C13.0 | Malignant neoplasm of postcricoid region |
| Solid Tumor Without Metastasis | C13.1 | Malignant neoplasm of aryepiglottic fold, hypopharyngeal aspect |
| Solid Tumor Without Metastasis | C13.2 | Malignant neoplasm of posterior wall of hypopharynx |
| Solid Tumor Without Metastasis | C13.8 | Malignant neoplasm of overlapping sites of hypopharynx |
| Solid Tumor Without Metastasis | C13.9 | Malignant neoplasm of hypopharynx, unspecified |
| Solid Tumor Without Metastasis | C14.0 | Malignant neoplasm of pharynx, unspecified |
| Solid Tumor Without Metastasis | C14.2 | Malignant neoplasm of Waldeyer's ring |
| Solid Tumor Without Metastasis | C14.8 | Malignant neoplasm of overlapping sites of lip, oral cavity and pharynx |
| Solid Tumor Without Metastasis | C15.3 | Malignant neoplasm of upper third of esophagus |
| Solid Tumor Without Metastasis | C15.4 | Malignant neoplasm of middle third of esophagus |
| Solid Tumor Without Metastasis | C15.5 | Malignant neoplasm of lower third of esophagus |
| Solid Tumor Without Metastasis | C15.8 | Malignant neoplasm of overlapping sites of esophagus |
| Solid Tumor Without Metastasis | C15.9 | Malignant neoplasm of esophagus, unspecified |
| Solid Tumor Without Metastasis | C16.0 | Malignant neoplasm of cardia |
| Solid Tumor Without Metastasis | C16.1 | Malignant neoplasm of fundus of stomach |
| Solid Tumor Without Metastasis | C16.2 | Malignant neoplasm of body of stomach |
| Solid Tumor Without Metastasis | C16.3 | Malignant neoplasm of pyloric antrum |
| Solid Tumor Without Metastasis | C16.4 | Malignant neoplasm of pylorus |
| Solid Tumor Without Metastasis | C16.5 | Malignant neoplasm of lesser curvature of stomach, unspecified |
| Solid Tumor Without Metastasis | C16.6 | Malignant neoplasm of greater curvature of stomach, unspecified |
| Solid Tumor Without Metastasis | C16.8 | Malignant neoplasm of overlapping sites of stomach |
| Solid Tumor Without Metastasis | C16.9 | Malignant neoplasm of stomach, unspecified |
| Solid Tumor Without Metastasis | C17.0 | Malignant neoplasm of duodenum |
| Solid Tumor Without Metastasis | C17.1 | Malignant neoplasm of jejunum |
| Solid Tumor Without Metastasis | C17.2 | Malignant neoplasm of ileum |
| Solid Tumor Without Metastasis | C17.3 | Meckel's diverticulum, malignant |
| Solid Tumor Without Metastasis | C17.8 | Malignant neoplasm of overlapping sites of small intestine |
| Solid Tumor Without Metastasis | C17.9 | Malignant neoplasm of small intestine, unspecified |
| Solid Tumor Without Metastasis | C18.0 | Malignant neoplasm of cecum |
| Solid Tumor Without Metastasis | C18.1 | Malignant neoplasm of appendix |
| Solid Tumor Without Metastasis | C18.2 | Malignant neoplasm of ascending colon |
| Solid Tumor Without Metastasis | C18.3 | Malignant neoplasm of hepatic flexure |
| Solid Tumor Without Metastasis | C18.4 | Malignant neoplasm of transverse colon |
| Solid Tumor Without Metastasis | C18.5 | Malignant neoplasm of splenic flexure |
| Solid Tumor Without Metastasis | C18.6 | Malignant neoplasm of descending colon |
| Solid Tumor Without Metastasis | C18.7 | Malignant neoplasm of sigmoid colon |
| Solid Tumor Without Metastasis | C18.8 | Malignant neoplasm of overlapping sites of colon |
| Solid Tumor Without Metastasis | C18.9 | Malignant neoplasm of colon, unspecified |
| Solid Tumor Without Metastasis | C19 | Malignant neoplasm of rectosigmoid junction |
| Solid Tumor Without Metastasis | C20 | Malignant neoplasm of rectum |
| Solid Tumor Without Metastasis | C21.0 | Malignant neoplasm of anus, unspecified |
| Solid Tumor Without Metastasis | C21.1 | Malignant neoplasm of anal canal |
| Solid Tumor Without Metastasis | C21.2 | Malignant neoplasm of cloacogenic zone |
| Solid Tumor Without Metastasis | C21.8 | Malignant neoplasm of overlapping sites of rectum, anus and anal canal |
| Solid Tumor Without Metastasis | C22.0 | Liver cell carcinoma |
| Solid Tumor Without Metastasis | C22.1 | Intrahepatic bile duct carcinoma |
| Solid Tumor Without Metastasis | C22.2 | Hepatoblastoma |
| Solid Tumor Without Metastasis | C22.3 | Angiosarcoma of liver |
| Solid Tumor Without Metastasis | C22.4 | Other sarcomas of liver |
| Solid Tumor Without Metastasis | C22.7 | Other specified carcinomas of liver |
| Solid Tumor Without Metastasis | C22.8 | Malignant neoplasm of liver, primary, unspecified as to type |
| Solid Tumor Without Metastasis | C22.9 | Malignant neoplasm of liver, not specified as primary or secondary |
| Solid Tumor Without Metastasis | C23 | Malignant neoplasm of gallbladder |
| Solid Tumor Without Metastasis | C24.0 | Malignant neoplasm of extrahepatic bile duct |
| Solid Tumor Without Metastasis | C24.1 | Malignant neoplasm of ampulla of Vater |
| Solid Tumor Without Metastasis | C24.8 | Malignant neoplasm of overlapping sites of biliary tract |
| Solid Tumor Without Metastasis | C24.9 | Malignant neoplasm of biliary tract, unspecified |
| Solid Tumor Without Metastasis | C25.0 | Malignant neoplasm of head of pancreas |
| Solid Tumor Without Metastasis | C25.1 | Malignant neoplasm of body of pancreas |
| Solid Tumor Without Metastasis | C25.2 | Malignant neoplasm of tail of pancreas |
| Solid Tumor Without Metastasis | C25.3 | Malignant neoplasm of pancreatic duct |
| Solid Tumor Without Metastasis | C25.4 | Malignant neoplasm of endocrine pancreas |
| Solid Tumor Without Metastasis | C25.7 | Malignant neoplasm of other parts of pancreas |
| Solid Tumor Without Metastasis | C25.8 | Malignant neoplasm of overlapping sites of pancreas |
| Solid Tumor Without Metastasis | C25.9 | Malignant neoplasm of pancreas, unspecified |
| Solid Tumor Without Metastasis | C26.0 | Malignant neoplasm of intestinal tract, part unspecified |
| Solid Tumor Without Metastasis | C26.1 | Malignant neoplasm of spleen |
| Solid Tumor Without Metastasis | C26.9 | Malignant neoplasm of ill-defined sites within the digestive system |
| Solid Tumor Without Metastasis | C30.0 | Malignant neoplasm of nasal cavity |
| Solid Tumor Without Metastasis | C30.1 | Malignant neoplasm of middle ear |
| Solid Tumor Without Metastasis | C31.0 | Malignant neoplasm of maxillary sinus |
| Solid Tumor Without Metastasis | C31.1 | Malignant neoplasm of ethmoidal sinus |
| Solid Tumor Without Metastasis | C31.2 | Malignant neoplasm of frontal sinus |
| Solid Tumor Without Metastasis | C31.3 | Malignant neoplasm of sphenoid sinus |
| Solid Tumor Without Metastasis | C31.8 | Malignant neoplasm of overlapping sites of accessory sinuses |
| Solid Tumor Without Metastasis | C31.9 | Malignant neoplasm of accessory sinus, unspecified |
| Solid Tumor Without Metastasis | C32.0 | Malignant neoplasm of glottis |
| Solid Tumor Without Metastasis | C32.1 | Malignant neoplasm of supraglottis |
| Solid Tumor Without Metastasis | C32.2 | Malignant neoplasm of subglottis |
| Solid Tumor Without Metastasis | C32.3 | Malignant neoplasm of laryngeal cartilage |
| Solid Tumor Without Metastasis | C32.8 | Malignant neoplasm of overlapping sites of larynx |
| Solid Tumor Without Metastasis | C32.9 | Malignant neoplasm of larynx, unspecified |
| Solid Tumor Without Metastasis | C33 | Malignant neoplasm of trachea |
| Solid Tumor Without Metastasis | C34.00 | Malignant neoplasm of unspecified main bronchus |
| Solid Tumor Without Metastasis | C34.01 | Malignant neoplasm of right main bronchus |
| Solid Tumor Without Metastasis | C34.02 | Malignant neoplasm of left main bronchus |
| Solid Tumor Without Metastasis | C34.10 | Malignant neoplasm of upper lobe, unspecified bronchus or lung |
| Solid Tumor Without Metastasis | C34.11 | Malignant neoplasm of upper lobe, right bronchus or lung |
| Solid Tumor Without Metastasis | C34.12 | Malignant neoplasm of upper lobe, left bronchus or lung |
| Solid Tumor Without Metastasis | C34.2 | Malignant neoplasm of middle lobe, bronchus or lung |
| Solid Tumor Without Metastasis | C34.30 | Malignant neoplasm of lower lobe, unspecified bronchus or lung |
| Solid Tumor Without Metastasis | C34.31 | Malignant neoplasm of lower lobe, right bronchus or lung |
| Solid Tumor Without Metastasis | C34.32 | Malignant neoplasm of lower lobe, left bronchus or lung |
| Solid Tumor Without Metastasis | C34.80 | Malignant neoplasm of overlapping sites of unspecified bronchus and lung |
| Solid Tumor Without Metastasis | C34.81 | Malignant neoplasm of overlapping sites of right bronchus and lung |
| Solid Tumor Without Metastasis | C34.82 | Malignant neoplasm of overlapping sites of left bronchus and lung |
| Solid Tumor Without Metastasis | C34.90 | Malignant neoplasm of unspecified part of unspecified bronchus or lung |
| Solid Tumor Without Metastasis | C34.91 | Malignant neoplasm of unspecified part of right bronchus or lung |
| Solid Tumor Without Metastasis | C34.92 | Malignant neoplasm of unspecified part of left bronchus or lung |
| Solid Tumor Without Metastasis | C37 | Malignant neoplasm of thymus |
| Solid Tumor Without Metastasis | C38.0 | Malignant neoplasm of heart |
| Solid Tumor Without Metastasis | C38.1 | Malignant neoplasm of anterior mediastinum |
| Solid Tumor Without Metastasis | C38.2 | Malignant neoplasm of posterior mediastinum |
| Solid Tumor Without Metastasis | C38.3 | Malignant neoplasm of mediastinum, part unspecified |
| Solid Tumor Without Metastasis | C38.4 | Malignant neoplasm of pleura |
| Solid Tumor Without Metastasis | C38.8 | Malignant neoplasm of overlapping sites of heart, mediastinum and pleura |
| Solid Tumor Without Metastasis | C39.0 | Malignant neoplasm of upper respiratory tract, part unspecified |
| Solid Tumor Without Metastasis | C39.9 | Malignant neoplasm of lower respiratory tract, part unspecified |
| Solid Tumor Without Metastasis | C40.00 | Malignant neoplasm of scapula and long bones of unspecified upper limb |
| Solid Tumor Without Metastasis | C40.01 | Malignant neoplasm of scapula and long bones of right upper limb |
| Solid Tumor Without Metastasis | C40.02 | Malignant neoplasm of scapula and long bones of left upper limb |
| Solid Tumor Without Metastasis | C40.10 | Malignant neoplasm of short bones of unspecified upper limb |
| Solid Tumor Without Metastasis | C40.11 | Malignant neoplasm of short bones of right upper limb |
| Solid Tumor Without Metastasis | C40.12 | Malignant neoplasm of short bones of left upper limb |
| Solid Tumor Without Metastasis | C40.20 | Malignant neoplasm of long bones of unspecified lower limb |
| Solid Tumor Without Metastasis | C40.21 | Malignant neoplasm of long bones of right lower limb |
| Solid Tumor Without Metastasis | C40.22 | Malignant neoplasm of long bones of left lower limb |
| Solid Tumor Without Metastasis | C40.30 | Malignant neoplasm of short bones of unspecified lower limb |
| Solid Tumor Without Metastasis | C40.31 | Malignant neoplasm of short bones of right lower limb |
| Solid Tumor Without Metastasis | C40.32 | Malignant neoplasm of short bones of left lower limb |
| Solid Tumor Without Metastasis | C40.80 | Malignant neoplasm of overlapping sites of bone and articular cartilage of unspecified limb |
| Solid Tumor Without Metastasis | C40.81 | Malignant neoplasm of overlapping sites of bone and articular cartilage of right limb |
| Solid Tumor Without Metastasis | C40.82 | Malignant neoplasm of overlapping sites of bone and articular cartilage of left limb |
| Solid Tumor Without Metastasis | C40.90 | Malignant neoplasm of unspecified bones and articular cartilage of unspecified limb |
| Solid Tumor Without Metastasis | C40.91 | Malignant neoplasm of unspecified bones and articular cartilage of right limb |
| Solid Tumor Without Metastasis | C40.92 | Malignant neoplasm of unspecified bones and articular cartilage of left limb |
| Solid Tumor Without Metastasis | C41.0 | Malignant neoplasm of bones of skull and face |
| Solid Tumor Without Metastasis | C41.1 | Malignant neoplasm of mandible |
| Solid Tumor Without Metastasis | C41.2 | Malignant neoplasm of vertebral column |
| Solid Tumor Without Metastasis | C41.3 | Malignant neoplasm of ribs, sternum and clavicle |
| Solid Tumor Without Metastasis | C41.4 | Malignant neoplasm of pelvic bones, sacrum and coccyx |
| Solid Tumor Without Metastasis | C41.9 | Malignant neoplasm of bone and articular cartilage, unspecified |
| Solid Tumor Without Metastasis | C43.0 | Malignant melanoma of lip |
| Solid Tumor Without Metastasis | C43.10 | Malignant melanoma of unspecified eyelid, including canthus |
| Solid Tumor Without Metastasis | C43.11 | Malignant melanoma of right eyelid, including canthus |
| Solid Tumor Without Metastasis | C43.12 | Malignant melanoma of left eyelid, including canthus |
| Solid Tumor Without Metastasis | C43.20 | Malignant melanoma of unspecified ear and external auricular canal |
| Solid Tumor Without Metastasis | C43.21 | Malignant melanoma of right ear and external auricular canal |
| Solid Tumor Without Metastasis | C43.22 | Malignant melanoma of left ear and external auricular canal |
| Solid Tumor Without Metastasis | C43.30 | Malignant melanoma of unspecified part of face |
| Solid Tumor Without Metastasis | C43.31 | Malignant melanoma of nose |
| Solid Tumor Without Metastasis | C43.39 | Malignant melanoma of other parts of face |
| Solid Tumor Without Metastasis | C43.4 | Malignant melanoma of scalp and neck |
| Solid Tumor Without Metastasis | C43.51 | Malignant melanoma of anal skin |
| Solid Tumor Without Metastasis | C43.52 | Malignant melanoma of skin of breast |
| Solid Tumor Without Metastasis | C43.59 | Malignant melanoma of other part of trunk |
| Solid Tumor Without Metastasis | C43.60 | Malignant melanoma of unspecified upper limb, including shoulder |
| Solid Tumor Without Metastasis | C43.61 | Malignant melanoma of right upper limb, including shoulder |
| Solid Tumor Without Metastasis | C43.62 | Malignant melanoma of left upper limb, including shoulder |
| Solid Tumor Without Metastasis | C43.70 | Malignant melanoma of unspecified lower limb, including hip |
| Solid Tumor Without Metastasis | C43.71 | Malignant melanoma of right lower limb, including hip |
| Solid Tumor Without Metastasis | C43.72 | Malignant melanoma of left lower limb, including hip |
| Solid Tumor Without Metastasis | C43.8 | Malignant melanoma of overlapping sites of skin |
| Solid Tumor Without Metastasis | C43.9 | Malignant melanoma of skin, unspecified |
| Solid Tumor Without Metastasis | C45.0 | Mesothelioma of pleura |
| Solid Tumor Without Metastasis | C45.1 | Mesothelioma of peritoneum |
| Solid Tumor Without Metastasis | C45.2 | Mesothelioma of pericardium |
| Solid Tumor Without Metastasis | C45.7 | Mesothelioma of other sites |
| Solid Tumor Without Metastasis | C45.9 | Mesothelioma, unspecified |
| Solid Tumor Without Metastasis | C46.0 | Kaposi's sarcoma of skin |
| Solid Tumor Without Metastasis | C46.1 | Kaposi's sarcoma of soft tissue |
| Solid Tumor Without Metastasis | C46.2 | Kaposi's sarcoma of palate |
| Solid Tumor Without Metastasis | C46.3 | Kaposi's sarcoma of lymph nodes |
| Solid Tumor Without Metastasis | C46.4 | Kaposi's sarcoma of gastrointestinal sites |
| Solid Tumor Without Metastasis | C46.50 | Kaposi's sarcoma of unspecified lung |
| Solid Tumor Without Metastasis | C46.51 | Kaposi's sarcoma of right lung |
| Solid Tumor Without Metastasis | C46.52 | Kaposi's sarcoma of left lung |
| Solid Tumor Without Metastasis | C46.7 | Kaposi's sarcoma of other sites |
| Solid Tumor Without Metastasis | C46.9 | Kaposi's sarcoma, unspecified |
| Solid Tumor Without Metastasis | C47.0 | Malignant neoplasm of peripheral nerves of head, face and neck |
| Solid Tumor Without Metastasis | C47.10 | Malignant neoplasm of peripheral nerves of unspecified upper limb, including shoulder |
| Solid Tumor Without Metastasis | C47.11 | Malignant neoplasm of peripheral nerves of right upper limb, including shoulder |
| Solid Tumor Without Metastasis | C47.12 | Malignant neoplasm of peripheral nerves of left upper limb, including shoulder |
| Solid Tumor Without Metastasis | C47.20 | Malignant neoplasm of peripheral nerves of unspecified lower limb, including hip |
| Solid Tumor Without Metastasis | C47.21 | Malignant neoplasm of peripheral nerves of right lower limb, including hip |
| Solid Tumor Without Metastasis | C47.22 | Malignant neoplasm of peripheral nerves of left lower limb, including hip |
| Solid Tumor Without Metastasis | C47.3 | Malignant neoplasm of peripheral nerves of thorax |
| Solid Tumor Without Metastasis | C47.4 | Malignant neoplasm of peripheral nerves of abdomen |
| Solid Tumor Without Metastasis | C47.5 | Malignant neoplasm of peripheral nerves of pelvis |
| Solid Tumor Without Metastasis | C47.6 | Malignant neoplasm of peripheral nerves of trunk, unspecified |
| Solid Tumor Without Metastasis | C47.8 | Malignant neoplasm of overlapping sites of peripheral nerves and autonomic nervous system |
| Solid Tumor Without Metastasis | C47.9 | Malignant neoplasm of peripheral nerves and autonomic nervous system, unspecified |
| Solid Tumor Without Metastasis | C48.0 | Malignant neoplasm of retroperitoneum |
| Solid Tumor Without Metastasis | C48.1 | Malignant neoplasm of specified parts of peritoneum |
| Solid Tumor Without Metastasis | C48.2 | Malignant neoplasm of peritoneum, unspecified |
| Solid Tumor Without Metastasis | C48.8 | Malignant neoplasm of overlapping sites of retroperitoneum and peritoneum |
| Solid Tumor Without Metastasis | C49.0 | Malignant neoplasm of connective and soft tissue of head, face and neck |
| Solid Tumor Without Metastasis | C49.10 | Malignant neoplasm of connective and soft tissue of unspecified upper limb, including shoulder |
| Solid Tumor Without Metastasis | C49.11 | Malignant neoplasm of connective and soft tissue of right upper limb, including shoulder |
| Solid Tumor Without Metastasis | C49.12 | Malignant neoplasm of connective and soft tissue of left upper limb, including shoulder |
| Solid Tumor Without Metastasis | C49.20 | Malignant neoplasm of connective and soft tissue of unspecified lower limb, including hip |
| Solid Tumor Without Metastasis | C49.21 | Malignant neoplasm of connective and soft tissue of right lower limb, including hip |
| Solid Tumor Without Metastasis | C49.22 | Malignant neoplasm of connective and soft tissue of left lower limb, including hip |
| Solid Tumor Without Metastasis | C49.3 | Malignant neoplasm of connective and soft tissue of thorax |
| Solid Tumor Without Metastasis | C49.4 | Malignant neoplasm of connective and soft tissue of abdomen |
| Solid Tumor Without Metastasis | C49.5 | Malignant neoplasm of connective and soft tissue of pelvis |
| Solid Tumor Without Metastasis | C49.6 | Malignant neoplasm of connective and soft tissue of trunk, unspecified |
| Solid Tumor Without Metastasis | C49.8 | Malignant neoplasm of overlapping sites of connective and soft tissue |
| Solid Tumor Without Metastasis | C49.9 | Malignant neoplasm of connective and soft tissue, unspecified |
| Solid Tumor Without Metastasis | C50.011 | Malignant neoplasm of nipple and areola, right female breast |
| Solid Tumor Without Metastasis | C50.012 | Malignant neoplasm of nipple and areola, left female breast |
| Solid Tumor Without Metastasis | C50.019 | Malignant neoplasm of nipple and areola, unspecified female breast |
| Solid Tumor Without Metastasis | C50.021 | Malignant neoplasm of nipple and areola, right male breast |
| Solid Tumor Without Metastasis | C50.022 | Malignant neoplasm of nipple and areola, left male breast |
| Solid Tumor Without Metastasis | C50.029 | Malignant neoplasm of nipple and areola, unspecified male breast |
| Solid Tumor Without Metastasis | C50.111 | Malignant neoplasm of central portion of right female breast |
| Solid Tumor Without Metastasis | C50.112 | Malignant neoplasm of central portion of left female breast |
| Solid Tumor Without Metastasis | C50.119 | Malignant neoplasm of central portion of unspecified female breast |
| Solid Tumor Without Metastasis | C50.121 | Malignant neoplasm of central portion of right male breast |
| Solid Tumor Without Metastasis | C50.122 | Malignant neoplasm of central portion of left male breast |
| Solid Tumor Without Metastasis | C50.129 | Malignant neoplasm of central portion of unspecified male breast |
| Solid Tumor Without Metastasis | C50.211 | Malignant neoplasm of upper-inner quadrant of right female breast |
| Solid Tumor Without Metastasis | C50.212 | Malignant neoplasm of upper-inner quadrant of left female breast |
| Solid Tumor Without Metastasis | C50.219 | Malignant neoplasm of upper-inner quadrant of unspecified female breast |
| Solid Tumor Without Metastasis | C50.221 | Malignant neoplasm of upper-inner quadrant of right male breast |
| Solid Tumor Without Metastasis | C50.222 | Malignant neoplasm of upper-inner quadrant of left male breast |
| Solid Tumor Without Metastasis | C50.229 | Malignant neoplasm of upper-inner quadrant of unspecified male breast |
| Solid Tumor Without Metastasis | C50.311 | Malignant neoplasm of lower-inner quadrant of right female breast |
| Solid Tumor Without Metastasis | C50.312 | Malignant neoplasm of lower-inner quadrant of left female breast |
| Solid Tumor Without Metastasis | C50.319 | Malignant neoplasm of lower-inner quadrant of unspecified female breast |
| Solid Tumor Without Metastasis | C50.321 | Malignant neoplasm of lower-inner quadrant of right male breast |
| Solid Tumor Without Metastasis | C50.322 | Malignant neoplasm of lower-inner quadrant of left male breast |
| Solid Tumor Without Metastasis | C50.329 | Malignant neoplasm of lower-inner quadrant of unspecified male breast |
| Solid Tumor Without Metastasis | C50.411 | Malignant neoplasm of upper-outer quadrant of right female breast |
| Solid Tumor Without Metastasis | C50.412 | Malignant neoplasm of upper-outer quadrant of left female breast |
| Solid Tumor Without Metastasis | C50.419 | Malignant neoplasm of upper-outer quadrant of unspecified female breast |
| Solid Tumor Without Metastasis | C50.421 | Malignant neoplasm of upper-outer quadrant of right male breast |
| Solid Tumor Without Metastasis | C50.422 | Malignant neoplasm of upper-outer quadrant of left male breast |
| Solid Tumor Without Metastasis | C50.429 | Malignant neoplasm of upper-outer quadrant of unspecified male breast |
| Solid Tumor Without Metastasis | C50.511 | Malignant neoplasm of lower-outer quadrant of right female breast |
| Solid Tumor Without Metastasis | C50.512 | Malignant neoplasm of lower-outer quadrant of left female breast |
| Solid Tumor Without Metastasis | C50.519 | Malignant neoplasm of lower-outer quadrant of unspecified female breast |
| Solid Tumor Without Metastasis | C50.521 | Malignant neoplasm of lower-outer quadrant of right male breast |
| Solid Tumor Without Metastasis | C50.522 | Malignant neoplasm of lower-outer quadrant of left male breast |
| Solid Tumor Without Metastasis | C50.529 | Malignant neoplasm of lower-outer quadrant of unspecified male breast |
| Solid Tumor Without Metastasis | C50.611 | Malignant neoplasm of axillary tail of right female breast |
| Solid Tumor Without Metastasis | C50.612 | Malignant neoplasm of axillary tail of left female breast |
| Solid Tumor Without Metastasis | C50.619 | Malignant neoplasm of axillary tail of unspecified female breast |
| Solid Tumor Without Metastasis | C50.621 | Malignant neoplasm of axillary tail of right male breast |
| Solid Tumor Without Metastasis | C50.622 | Malignant neoplasm of axillary tail of left male breast |
| Solid Tumor Without Metastasis | C50.629 | Malignant neoplasm of axillary tail of unspecified male breast |
| Solid Tumor Without Metastasis | C50.811 | Malignant neoplasm of overlapping sites of right female breast |
| Solid Tumor Without Metastasis | C50.812 | Malignant neoplasm of overlapping sites of left female breast |
| Solid Tumor Without Metastasis | C50.819 | Malignant neoplasm of overlapping sites of unspecified female breast |
| Solid Tumor Without Metastasis | C50.821 | Malignant neoplasm of overlapping sites of right male breast |
| Solid Tumor Without Metastasis | C50.822 | Malignant neoplasm of overlapping sites of left male breast |
| Solid Tumor Without Metastasis | C50.829 | Malignant neoplasm of overlapping sites of unspecified male breast |
| Solid Tumor Without Metastasis | C50.911 | Malignant neoplasm of unspecified site of right female breast |
| Solid Tumor Without Metastasis | C50.912 | Malignant neoplasm of unspecified site of left female breast |
| Solid Tumor Without Metastasis | C50.919 | Malignant neoplasm of unspecified site of unspecified female breast |
| Solid Tumor Without Metastasis | C50.921 | Malignant neoplasm of unspecified site of right male breast |
| Solid Tumor Without Metastasis | C50.922 | Malignant neoplasm of unspecified site of left male breast |
| Solid Tumor Without Metastasis | C50.929 | Malignant neoplasm of unspecified site of unspecified male breast |
| Solid Tumor Without Metastasis | C51.0 | Malignant neoplasm of labium majus |
| Solid Tumor Without Metastasis | C51.1 | Malignant neoplasm of labium minus |
| Solid Tumor Without Metastasis | C51.2 | Malignant neoplasm of clitoris |
| Solid Tumor Without Metastasis | C51.8 | Malignant neoplasm of overlapping sites of vulva |
| Solid Tumor Without Metastasis | C51.9 | Malignant neoplasm of vulva, unspecified |
| Solid Tumor Without Metastasis | C52 | Malignant neoplasm of vagina |
| Solid Tumor Without Metastasis | C53.0 | Malignant neoplasm of endocervix |
| Solid Tumor Without Metastasis | C53.1 | Malignant neoplasm of exocervix |
| Solid Tumor Without Metastasis | C53.8 | Malignant neoplasm of overlapping sites of cervix uteri |
| Solid Tumor Without Metastasis | C53.9 | Malignant neoplasm of cervix uteri, unspecified |
| Solid Tumor Without Metastasis | C54.0 | Malignant neoplasm of isthmus uteri |
| Solid Tumor Without Metastasis | C54.1 | Malignant neoplasm of endometrium |
| Solid Tumor Without Metastasis | C54.2 | Malignant neoplasm of myometrium |
| Solid Tumor Without Metastasis | C54.3 | Malignant neoplasm of fundus uteri |
| Solid Tumor Without Metastasis | C54.8 | Malignant neoplasm of overlapping sites of corpus uteri |
| Solid Tumor Without Metastasis | C54.9 | Malignant neoplasm of corpus uteri, unspecified |
| Solid Tumor Without Metastasis | C55 | Malignant neoplasm of uterus, part unspecified |
| Solid Tumor Without Metastasis | C56.1 | Malignant neoplasm of right ovary |
| Solid Tumor Without Metastasis | C56.2 | Malignant neoplasm of left ovary |
| Solid Tumor Without Metastasis | C56.9 | Malignant neoplasm of unspecified ovary |
| Solid Tumor Without Metastasis | C57.00 | Malignant neoplasm of unspecified fallopian tube |
| Solid Tumor Without Metastasis | C57.01 | Malignant neoplasm of right fallopian tube |
| Solid Tumor Without Metastasis | C57.02 | Malignant neoplasm of left fallopian tube |
| Solid Tumor Without Metastasis | C57.10 | Malignant neoplasm of unspecified broad ligament |
| Solid Tumor Without Metastasis | C57.11 | Malignant neoplasm of right broad ligament |
| Solid Tumor Without Metastasis | C57.12 | Malignant neoplasm of left broad ligament |
| Solid Tumor Without Metastasis | C57.20 | Malignant neoplasm of unspecified round ligament |
| Solid Tumor Without Metastasis | C57.21 | Malignant neoplasm of right round ligament |
| Solid Tumor Without Metastasis | C57.22 | Malignant neoplasm of left round ligament |
| Solid Tumor Without Metastasis | C57.3 | Malignant neoplasm of parametrium |
| Solid Tumor Without Metastasis | C57.4 | Malignant neoplasm of uterine adnexa, unspecified |
| Solid Tumor Without Metastasis | C57.7 | Malignant neoplasm of other specified female genital organs |
| Solid Tumor Without Metastasis | C57.8 | Malignant neoplasm of overlapping sites of female genital organs |
| Solid Tumor Without Metastasis | C57.9 | Malignant neoplasm of female genital organ, unspecified |
| Solid Tumor Without Metastasis | C58 | Malignant neoplasm of placenta |
| Solid Tumor Without Metastasis | C60.0 | Malignant neoplasm of prepuce |
| Solid Tumor Without Metastasis | C60.1 | Malignant neoplasm of glans penis |
| Solid Tumor Without Metastasis | C60.2 | Malignant neoplasm of body of penis |
| Solid Tumor Without Metastasis | C60.8 | Malignant neoplasm of overlapping sites of penis |
| Solid Tumor Without Metastasis | C60.9 | Malignant neoplasm of penis, unspecified |
| Solid Tumor Without Metastasis | C61 | Malignant neoplasm of prostate |
| Solid Tumor Without Metastasis | C62.00 | Malignant neoplasm of unspecified undescended testis |
| Solid Tumor Without Metastasis | C62.01 | Malignant neoplasm of undescended right testis |
| Solid Tumor Without Metastasis | C62.02 | Malignant neoplasm of undescended left testis |
| Solid Tumor Without Metastasis | C62.10 | Malignant neoplasm of unspecified descended testis |
| Solid Tumor Without Metastasis | C62.11 | Malignant neoplasm of descended right testis |
| Solid Tumor Without Metastasis | C62.12 | Malignant neoplasm of descended left testis |
| Solid Tumor Without Metastasis | C62.90 | Malignant neoplasm of unspecified testis, unspecified whether descended or undescended |
| Solid Tumor Without Metastasis | C62.91 | Malignant neoplasm of right testis, unspecified whether descended or undescended |
| Solid Tumor Without Metastasis | C62.92 | Malignant neoplasm of left testis, unspecified whether descended or undescended |
| Solid Tumor Without Metastasis | C63.00 | Malignant neoplasm of unspecified epididymis |
| Solid Tumor Without Metastasis | C63.01 | Malignant neoplasm of right epididymis |
| Solid Tumor Without Metastasis | C63.02 | Malignant neoplasm of left epididymis |
| Solid Tumor Without Metastasis | C63.10 | Malignant neoplasm of unspecified spermatic cord |
| Solid Tumor Without Metastasis | C63.11 | Malignant neoplasm of right spermatic cord |
| Solid Tumor Without Metastasis | C63.12 | Malignant neoplasm of left spermatic cord |
| Solid Tumor Without Metastasis | C63.2 | Malignant neoplasm of scrotum |
| Solid Tumor Without Metastasis | C63.7 | Malignant neoplasm of other specified male genital organs |
| Solid Tumor Without Metastasis | C63.8 | Malignant neoplasm of overlapping sites of male genital organs |
| Solid Tumor Without Metastasis | C63.9 | Malignant neoplasm of male genital organ, unspecified |
| Solid Tumor Without Metastasis | C64.1 | Malignant neoplasm of right kidney, except renal pelvis |
| Solid Tumor Without Metastasis | C64.2 | Malignant neoplasm of left kidney, except renal pelvis |
| Solid Tumor Without Metastasis | C64.9 | Malignant neoplasm of unspecified kidney, except renal pelvis |
| Solid Tumor Without Metastasis | C65.1 | Malignant neoplasm of right renal pelvis |
| Solid Tumor Without Metastasis | C65.2 | Malignant neoplasm of left renal pelvis |
| Solid Tumor Without Metastasis | C65.9 | Malignant neoplasm of unspecified renal pelvis |
| Solid Tumor Without Metastasis | C66.1 | Malignant neoplasm of right ureter |
| Solid Tumor Without Metastasis | C66.2 | Malignant neoplasm of left ureter |
| Solid Tumor Without Metastasis | C66.9 | Malignant neoplasm of unspecified ureter |
| Solid Tumor Without Metastasis | C67.0 | Malignant neoplasm of trigone of bladder |
| Solid Tumor Without Metastasis | C67.1 | Malignant neoplasm of dome of bladder |
| Solid Tumor Without Metastasis | C67.2 | Malignant neoplasm of lateral wall of bladder |
| Solid Tumor Without Metastasis | C67.3 | Malignant neoplasm of anterior wall of bladder |
| Solid Tumor Without Metastasis | C67.4 | Malignant neoplasm of posterior wall of bladder |
| Solid Tumor Without Metastasis | C67.5 | Malignant neoplasm of bladder neck |
| Solid Tumor Without Metastasis | C67.6 | Malignant neoplasm of ureteric orifice |
| Solid Tumor Without Metastasis | C67.7 | Malignant neoplasm of urachus |
| Solid Tumor Without Metastasis | C67.8 | Malignant neoplasm of overlapping sites of bladder |
| Solid Tumor Without Metastasis | C67.9 | Malignant neoplasm of bladder, unspecified |
| Solid Tumor Without Metastasis | C68.0 | Malignant neoplasm of urethra |
| Solid Tumor Without Metastasis | C68.1 | Malignant neoplasm of paraurethral glands |
| Solid Tumor Without Metastasis | C68.8 | Malignant neoplasm of overlapping sites of urinary organs |
| Solid Tumor Without Metastasis | C68.9 | Malignant neoplasm of urinary organ, unspecified |
| Solid Tumor Without Metastasis | C69.00 | Malignant neoplasm of unspecified conjunctiva |
| Solid Tumor Without Metastasis | C69.01 | Malignant neoplasm of right conjunctiva |
| Solid Tumor Without Metastasis | C69.02 | Malignant neoplasm of left conjunctiva |
| Solid Tumor Without Metastasis | C69.10 | Malignant neoplasm of unspecified cornea |
| Solid Tumor Without Metastasis | C69.11 | Malignant neoplasm of right cornea |
| Solid Tumor Without Metastasis | C69.12 | Malignant neoplasm of left cornea |
| Solid Tumor Without Metastasis | C69.20 | Malignant neoplasm of unspecified retina |
| Solid Tumor Without Metastasis | C69.21 | Malignant neoplasm of right retina |
| Solid Tumor Without Metastasis | C69.22 | Malignant neoplasm of left retina |
| Solid Tumor Without Metastasis | C69.30 | Malignant neoplasm of unspecified choroid |
| Solid Tumor Without Metastasis | C69.31 | Malignant neoplasm of right choroid |
| Solid Tumor Without Metastasis | C69.32 | Malignant neoplasm of left choroid |
| Solid Tumor Without Metastasis | C69.40 | Malignant neoplasm of unspecified ciliary body |
| Solid Tumor Without Metastasis | C69.41 | Malignant neoplasm of right ciliary body |
| Solid Tumor Without Metastasis | C69.42 | Malignant neoplasm of left ciliary body |
| Solid Tumor Without Metastasis | C69.50 | Malignant neoplasm of unspecified lacrimal gland and duct |
| Solid Tumor Without Metastasis | C69.51 | Malignant neoplasm of right lacrimal gland and duct |
| Solid Tumor Without Metastasis | C69.52 | Malignant neoplasm of left lacrimal gland and duct |
| Solid Tumor Without Metastasis | C69.60 | Malignant neoplasm of unspecified orbit |
| Solid Tumor Without Metastasis | C69.61 | Malignant neoplasm of right orbit |
| Solid Tumor Without Metastasis | C69.62 | Malignant neoplasm of left orbit |
| Solid Tumor Without Metastasis | C69.80 | Malignant neoplasm of overlapping sites of unspecified eye and adnexa |
| Solid Tumor Without Metastasis | C69.81 | Malignant neoplasm of overlapping sites of right eye and adnexa |
| Solid Tumor Without Metastasis | C69.82 | Malignant neoplasm of overlapping sites of left eye and adnexa |
| Solid Tumor Without Metastasis | C69.90 | Malignant neoplasm of unspecified site of unspecified eye |
| Solid Tumor Without Metastasis | C69.91 | Malignant neoplasm of unspecified site of right eye |
| Solid Tumor Without Metastasis | C69.92 | Malignant neoplasm of unspecified site of left eye |
| Solid Tumor Without Metastasis | C70.0 | Malignant neoplasm of cerebral meninges |
| Solid Tumor Without Metastasis | C70.1 | Malignant neoplasm of spinal meninges |
| Solid Tumor Without Metastasis | C70.9 | Malignant neoplasm of meninges, unspecified |
| Solid Tumor Without Metastasis | C71.0 | Malignant neoplasm of cerebrum, except lobes and ventricles |
| Solid Tumor Without Metastasis | C71.1 | Malignant neoplasm of frontal lobe |
| Solid Tumor Without Metastasis | C71.2 | Malignant neoplasm of temporal lobe |
| Solid Tumor Without Metastasis | C71.3 | Malignant neoplasm of parietal lobe |
| Solid Tumor Without Metastasis | C71.4 | Malignant neoplasm of occipital lobe |
| Solid Tumor Without Metastasis | C71.5 | Malignant neoplasm of cerebral ventricle |
| Solid Tumor Without Metastasis | C71.6 | Malignant neoplasm of cerebellum |
| Solid Tumor Without Metastasis | C71.7 | Malignant neoplasm of brain stem |
| Solid Tumor Without Metastasis | C71.8 | Malignant neoplasm of overlapping sites of brain |
| Solid Tumor Without Metastasis | C71.9 | Malignant neoplasm of brain, unspecified |
| Solid Tumor Without Metastasis | C72.0 | Malignant neoplasm of spinal cord |
| Solid Tumor Without Metastasis | C72.1 | Malignant neoplasm of cauda equina |
| Solid Tumor Without Metastasis | C72.20 | Malignant neoplasm of unspecified olfactory nerve |
| Solid Tumor Without Metastasis | C72.21 | Malignant neoplasm of right olfactory nerve |
| Solid Tumor Without Metastasis | C72.22 | Malignant neoplasm of left olfactory nerve |
| Solid Tumor Without Metastasis | C72.30 | Malignant neoplasm of unspecified optic nerve |
| Solid Tumor Without Metastasis | C72.31 | Malignant neoplasm of right optic nerve |
| Solid Tumor Without Metastasis | C72.32 | Malignant neoplasm of left optic nerve |
| Solid Tumor Without Metastasis | C72.40 | Malignant neoplasm of unspecified acoustic nerve |
| Solid Tumor Without Metastasis | C72.41 | Malignant neoplasm of right acoustic nerve |
| Solid Tumor Without Metastasis | C72.42 | Malignant neoplasm of left acoustic nerve |
| Solid Tumor Without Metastasis | C72.50 | Malignant neoplasm of unspecified cranial nerve |
| Solid Tumor Without Metastasis | C72.59 | Malignant neoplasm of other cranial nerves |
| Solid Tumor Without Metastasis | C72.9 | Malignant neoplasm of central nervous system, unspecified |
| Solid Tumor Without Metastasis | C73 | Malignant neoplasm of thyroid gland |
| Solid Tumor Without Metastasis | C74.00 | Malignant neoplasm of cortex of unspecified adrenal gland |
| Solid Tumor Without Metastasis | C74.01 | Malignant neoplasm of cortex of right adrenal gland |
| Solid Tumor Without Metastasis | C74.02 | Malignant neoplasm of cortex of left adrenal gland |
| Solid Tumor Without Metastasis | C74.10 | Malignant neoplasm of medulla of unspecified adrenal gland |
| Solid Tumor Without Metastasis | C74.11 | Malignant neoplasm of medulla of right adrenal gland |
| Solid Tumor Without Metastasis | C74.12 | Malignant neoplasm of medulla of left adrenal gland |
| Solid Tumor Without Metastasis | C74.90 | Malignant neoplasm of unspecified part of unspecified adrenal gland |
| Solid Tumor Without Metastasis | C74.91 | Malignant neoplasm of unspecified part of right adrenal gland |
| Solid Tumor Without Metastasis | C74.92 | Malignant neoplasm of unspecified part of left adrenal gland |
| Solid Tumor Without Metastasis | C75.0 | Malignant neoplasm of parathyroid gland |
| Solid Tumor Without Metastasis | C75.1 | Malignant neoplasm of pituitary gland |
| Solid Tumor Without Metastasis | C75.2 | Malignant neoplasm of craniopharyngeal duct |
| Solid Tumor Without Metastasis | C75.3 | Malignant neoplasm of pineal gland |
| Solid Tumor Without Metastasis | C75.4 | Malignant neoplasm of carotid body |
| Solid Tumor Without Metastasis | C75.5 | Malignant neoplasm of aortic body and other paraganglia |
| Solid Tumor Without Metastasis | C75.8 | Malignant neoplasm with pluriglandular involvement, unspecified |
| Solid Tumor Without Metastasis | C75.9 | Malignant neoplasm of endocrine gland, unspecified |
| Solid Tumor Without Metastasis | C76.0 | Malignant neoplasm of head, face and neck |
| Solid Tumor Without Metastasis | C76.1 | Malignant neoplasm of thorax |
| Solid Tumor Without Metastasis | C76.2 | Malignant neoplasm of abdomen |
| Solid Tumor Without Metastasis | C76.3 | Malignant neoplasm of pelvis |
| Solid Tumor Without Metastasis | C76.40 | Malignant neoplasm of unspecified upper limb |
| Solid Tumor Without Metastasis | C76.41 | Malignant neoplasm of right upper limb |
| Solid Tumor Without Metastasis | C76.42 | Malignant neoplasm of left upper limb |
| Solid Tumor Without Metastasis | C76.50 | Malignant neoplasm of unspecified lower limb |
| Solid Tumor Without Metastasis | C76.51 | Malignant neoplasm of right lower limb |
| Solid Tumor Without Metastasis | C76.52 | Malignant neoplasm of left lower limb |
| Solid Tumor Without Metastasis | C76.8 | Malignant neoplasm of other specified ill-defined sites |
| Valvular Disease | A52.00 | Cardiovascular syphilis, unspecified |
| Valvular Disease | A52.01 | Syphilitic aneurysm of aorta |
| Valvular Disease | A52.02 | Syphilitic aortitis |
| Valvular Disease | A52.03 | Syphilitic endocarditis |
| Valvular Disease | A52.04 | Syphilitic cerebral arteritis |
| Valvular Disease | A52.05 | Other cerebrovascular syphilis |
| Valvular Disease | A52.06 | Other syphilitic heart involvement |
| Valvular Disease | A52.09 | Other cardiovascular syphilis |
| Valvular Disease | I05.0 | Rheumatic mitral stenosis |
| Valvular Disease | I05.1 | Rheumatic mitral insufficiency |
| Valvular Disease | I05.2 | Rheumatic mitral stenosis with insufficiency |
| Valvular Disease | I05.8 | Other rheumatic mitral valve diseases |
| Valvular Disease | I05.9 | Rheumatic mitral valve disease, unspecified |
| Valvular Disease | I06.0 | Rheumatic aortic stenosis |
| Valvular Disease | I06.1 | Rheumatic aortic insufficiency |
| Valvular Disease | I06.2 | Rheumatic aortic stenosis with insufficiency |
| Valvular Disease | I06.8 | Other rheumatic aortic valve diseases |
| Valvular Disease | I06.9 | Rheumatic aortic valve disease, unspecified |
| Valvular Disease | I07.0 | Rheumatic tricuspid stenosis |
| Valvular Disease | I07.1 | Rheumatic tricuspid insufficiency |
| Valvular Disease | I07.2 | Rheumatic tricuspid stenosis and insufficiency |
| Valvular Disease | I07.8 | Other rheumatic tricuspid valve diseases |
| Valvular Disease | I07.9 | Rheumatic tricuspid valve disease, unspecified |
| Valvular Disease | I08.0 | Rheumatic disorders of both mitral and aortic valves |
| Valvular Disease | I08.1 | Rheumatic disorders of both mitral and tricuspid valves |
| Valvular Disease | I08.2 | Rheumatic disorders of both aortic and tricuspid valves |
| Valvular Disease | I08.3 | Combined rheumatic disorders of mitral, aortic and tricuspid valves |
| Valvular Disease | I08.8 | Other rheumatic multiple valve diseases |
| Valvular Disease | I08.9 | Rheumatic multiple valve disease, unspecified |
| Valvular Disease | I09.1 | Rheumatic diseases of endocardium, valve unspecified |
| Valvular Disease | I09.81 | Rheumatic heart failure |
| Valvular Disease | I09.89 | Other specified rheumatic heart diseases |
| Valvular Disease | I34.0 | Nonrheumatic mitral (valve) insufficiency |
| Valvular Disease | I34.1 | Nonrheumatic mitral (valve) prolapse |
| Valvular Disease | I34.2 | Nonrheumatic mitral (valve) stenosis |
| Valvular Disease | I34.8 | Other nonrheumatic mitral valve disorders |
| Valvular Disease | I34.9 | Nonrheumatic mitral valve disorder, unspecified |
| Valvular Disease | I35.0 | Nonrheumatic aortic (valve) stenosis |
| Valvular Disease | I35.1 | Nonrheumatic aortic (valve) insufficiency |
| Valvular Disease | I35.2 | Nonrheumatic aortic (valve) stenosis with insufficiency |
| Valvular Disease | I35.8 | Other nonrheumatic aortic valve disorders |
| Valvular Disease | I35.9 | Nonrheumatic aortic valve disorder, unspecified |
| Valvular Disease | I36.0 | Nonrheumatic tricuspid (valve) stenosis |
| Valvular Disease | I36.1 | Nonrheumatic tricuspid (valve) insufficiency |
| Valvular Disease | I36.2 | Nonrheumatic tricuspid (valve) stenosis with insufficiency |
| Valvular Disease | I36.8 | Other nonrheumatic tricuspid valve disorders |
| Valvular Disease | I36.9 | Nonrheumatic tricuspid valve disorder, unspecified |
| Valvular Disease | I37.0 | Nonrheumatic pulmonary valve stenosis |
| Valvular Disease | I37.1 | Nonrheumatic pulmonary valve insufficiency |
| Valvular Disease | I37.2 | Nonrheumatic pulmonary valve stenosis with insufficiency |
| Valvular Disease | I37.8 | Other nonrheumatic pulmonary valve disorders |
| Valvular Disease | I37.9 | Nonrheumatic pulmonary valve disorder, unspecified |
| Valvular Disease | I38 | Endocarditis, valve unspecified |
| Valvular Disease | I39 | Endocarditis and heart valve disorders in diseases classified elsewhere |
| Valvular Disease | Q23.0 | Congenital stenosis of aortic valve |
| Valvular Disease | Q23.1 | Congenital insufficiency of aortic valve |
| Valvular Disease | Q23.2 | Congenital mitral stenosis |
| Valvular Disease | Q23.3 | Congenital mitral insufficiency |
| Valvular Disease | Z95.2 | Presence of prosthetic heart valve |
| Valvular Disease | Z95.3 | Presence of xenogenic heart valve |
| Valvular Disease | Z95.4 | Presence of other heart-valve replacement |
| Weight Loss | E40 | Kwashiorkor |
| Weight Loss | E41 | Nutritional marasmus |
| Weight Loss | E42 | Marasmic kwashiorkor |
| Weight Loss | E43 | Unspecified severe protein-calorie malnutrition |
| Weight Loss | E44.0 | Moderate protein-calorie malnutrition |
| Weight Loss | E44.1 | Mild protein-calorie malnutrition |
| Weight Loss | E45 | Retarded development following protein-calorie malnutrition |
| Weight Loss | E46 | Unspecified protein-calorie malnutrition |
| Weight Loss | R63.4 | Abnormal weight loss |
| Weight Loss | R64 | Cachexia |

**Appendix E – Prolonged Ventilation**

| **Variable** | **Code** | **Description** |
| --- | --- | --- |
| Prolonged Ventilation | 5A09457 | Assistance with Respiratory Ventilation, 24-96 Consecutive Hours, Continuous Positive Airway Pressure |
| Prolonged Ventilation | 5A09458 | Assistance with Respiratory Ventilation, 24-96 Consecutive Hours, Intermittent Positive Airway Pressure |
| Prolonged Ventilation | 5A09459 | Assistance with Respiratory Ventilation, 24-96 Consecutive Hours, Continuous Negative Airway Pressure |
| Prolonged Ventilation | 5A0945B | Assistance with Respiratory Ventilation, 24-96 Consecutive Hours, Intermittent Negative Airway Pressure |
| Prolonged Ventilation | 5A0945Z | Assistance with Respiratory Ventilation, 24-96 Consecutive Hours |
| Prolonged Ventilation | 5A09557 | Assistance with Respiratory Ventilation, Greater than 96 Consecutive Hours, Continuous Positive Airway Pressure |
| Prolonged Ventilation | 5A09558 | Assistance with Respiratory Ventilation, Greater than 96 Consecutive Hours, Intermittent Positive Airway Pressure |
| Prolonged Ventilation | 5A09559 | Assistance with Respiratory Ventilation, Greater than 96 Consecutive Hours, Continuous Negative Airway Pressure |
| Prolonged Ventilation | 5A0955B | Assistance with Respiratory Ventilation, Greater than 96 Consecutive Hours, Intermittent Negative Airway Pressure |
| Prolonged Ventilation | 5A0955Z | Assistance with Respiratory Ventilation, Greater than 96 Consecutive Hours |
| Prolonged Ventilation | 5A1945Z | Respiratory Ventilation, 24-96 Consecutive Hours |
| Prolonged Ventilation | 5A1955Z | Respiratory Ventilation, Greater than 96 Consecutive Hours |
